# Metabolomic Atlas of Cardiovascular Diseases: Mapping Shared and Specific Signatures

**DOI:** 10.1016/j.jacadv.2026.102742

**Published:** 2026-04-17

**Authors:** Jingjing Yang, Wanshan Ning, Ruizhi Xu, Yaping Guo, Amei He, Jiajun Fan, Yanbo Wang, Xingyu Li, Qun Chen

**Affiliations:** a Department of Pulmonary and Critical Care Medicine, The First Affiliated Hospital of Xiamen University, School of Medicine, Xiamen University, Xiamen, Fujian, China; b Institute of Clinical Medical Research, The First Affiliated Hospital, School of Medicine, Xiamen University, Xiamen, Fujian, China; c Nanjing Drum Tower Hospital Center of Molecular Diagnostic and Therapy, Chinese Academy of Medical Sciences Research Unit of Extracellular RNA, State Key Laboratory of Pharmaceutical Biotechnology and Department of Physiology, Jiangsu Engineering Research Center for MicroRNA Biology and Biotechnology, NJU Advanced Institute of Life Sciences (NAILS), School of Life Sciences, Nanjing University, Nanjing, China; d Department of Pathophysiology, School of Basic Medical Sciences, Zhengzhou University, Zhengzhou, Henan, China; e Siebel School of Computing and Data Science, University of Illinois Urbana-Champaign, Champaign, Illinois, USA; f Department of Hepatopancreatobiliary Surgery, The Third Xiangya Hospital, Central South University, Changsha, Hunan, China; g Xiamen Cell Therapy Research Center, The First Affiliated Hospital of Xiamen University, School of Medicine, Xiamen University, Xiamen, China

**Keywords:** cardiovascular disease, cross-CVD signatures, machine learning, metabolic heterogeneity, UK Biobank

## Abstract

**Background:**

Cardiovascular disease (CVD) remains the leading global cause of death. While metabolic dysregulation is central to CVD pathogenesis, the extent to which distinct clinical subtypes exhibit unique or shared metabolic signatures remains unclear.

**Objectives:**

The purpose of this study was to systematically characterize metabolomic patterns across a broad spectrum of CVD subtypes and to delineate both shared and subtype-specific metabolic features.

**Methods:**

We analyzed nuclear magnetic resonance–based metabolomic data (325 metabolites) from 244,567 UK Biobank participants, including 27,950 with prevalent CVDs classified into 87 phenotypes (37 classes, 50 subclasses) using International Classification of Diseases-10th Revision codes. Logistic regression, random forest, and XGBoost models assessed cross-sectional metabolite-disease associations. SHapley Additive exPlanations analysis identified key discriminative features. Internal geographic validation used Scotland and Wales cohorts.

**Results:**

Metabolomic profiles demonstrated substantial heterogeneity across CVD subtypes. We identified 21 metabolites consistently associated with multiple conditions, including Intermediate-Density Lipoprotein cholesteryl esters, linoleic acid percentage, and small very low_density lipoprotein particles, reflecting shared alterations in lipoprotein metabolism and inflammatory pathways. Cross-sectional discrimination models achieved moderate-to-high performance for prevalent disease status (eg, chronic ischemic heart disease area under the curve = 0.876). Disease similarity clustering revealed reproducible organizational structures: ischemic entities formed tight clusters, hypertensive-renal diseases showed graded patterns, while rheumatic and pulmonary conditions remained distinct. These patterns were confirmed in geographic validation cohorts.

**Conclusions:**

This comprehensive metabolomic atlas reveals both shared and subtype-specific metabolic alterations across prevalent CVD. The identified metabolite set provides a hypothesis-generating framework for understanding cardiovascular metabolic heterogeneity. However, the cross-sectional design and inclusion of treated patients preclude causal or predictive inference, requiring validation in prospective, treatment-naive cohorts.

Cardiovascular diseases (CVDs) represent the leading cause of global mortality and morbidity, imposing an immense burden on health care systems and economies worldwide.^1^^,^^2^ Despite significant advances in prevention and treatment, CVDs continue to account for approximately 17.9 million deaths annually, with a substantial proportion occurring prematurely.^2^^,^^3^ This persistent challenge underscores the urgent need for more refined characterization of disease heterogeneity and deeper biological understanding of cardiovascular conditions, rather than relying solely on conventional clinical classifications.

Metabolic dysregulation is a well-established cornerstone in the pathogenesis of various CVDs, influencing processes ranging from lipid metabolism and inflammation to vascular function and energy homeostasis.^4^, ^5^, ^6^ Traditional metabolic markers, such as cholesterol and glucose levels, have long been integral to CVD risk assessment.^7^^,^^8^ However, the advent of high-throughput metabolomics technologies has revolutionized our ability to capture a comprehensive snapshot of the dynamic biochemical state of an individual.^9^, ^10^, ^11^ By simultaneously quantifying a wide array of small molecules, metabolomics offers unprecedented opportunities to systematically describe disease-associated metabolic alterations and to generate hypotheses regarding underlying biological mechanisms, as well as to gain deeper insights into the complex interplay between genetic, environmental, and lifestyle factors in CVD development.

Many studies have been conducted on relatively small cohorts, limiting statistical power and the ability to generalize findings across populations.^12^^,^^13^ Furthermore, research has typically focused on specific CVD subtypes (eg, acute myocardial infarction, heart failure) or a limited subset of metabolites, precluding a systematic analysis across the entire spectrum of cardiovascular conditions defined by standardized classifications such as International Classification of Diseases-10th Revision (ICD-10).^14^^,^^15^ As a result, it remains unclear which metabolic alterations are broadly shared across multiple CVD manifestations and which are specific to particular disease entities. Disentangling shared vs subtype-specific metabolic patterns is essential for advancing etiological understanding of CVD heterogeneity, yet a large-scale, systematic investigation across the entire CVD spectrum has been lacking.

The recent availability of large-scale population cohorts with comprehensive, high-throughput metabolic profiling offers an unprecedented opportunity to overcome the limitations of previous studies and systematically address the question of CVD metabolic heterogeneity. The UK Biobank (UKB), with its vast number of participants and detailed health data, including high-quality nuclear magnetic resonance (NMR) metabolomics, provides an ideal platform for such an investigation.^11^^,^^16^ Given the high dimensionality of metabolomic data, integrative statistical and computational approaches are required to summarize complex association patterns across disease categories. In this context, machine learning methods, particularly those equipped with model interpretation frameworks such as SHapley Additive exPlanations (SHAP), can facilitate the systematic evaluation of metabolites that contribute to cross-sectional differentiation among disease phenotypes.^17^

Against this background, our study aimed to conduct a comprehensive metabolomic atlas—a systematic cartography of cardiovascular metabolic heterogeneity, leveraging the UKB NMR metabolomics data within a cross-sectional, population-based framework (Supplemental Figure 1). Specifically, we sought to: 1) map detailed metabolomic profiles across the CVD spectrum; 2) explore the metabolic relevance and overlaps between different CVD categories, to understand which patterns are shared and which are specific to certain subtypes; 3) identify metabolites consistently associated with multiple CVD subtypes (“cross-CVD metabolic features”, ie, recurrent top contributors across diseases with stable directionality) as well as metabolites predominantly linked to specific disease categories (“subtype-linked metabolic patterns”); and 4) quantify the degree of metabolic distinctiveness as a descriptive measure of disease-associated signatures, rather than to perform prospective prediction.

## Methods

The overall workflow chart was illustrated in Supplemental Figure 1. The study leveraged UKB data to characterize metabolic associations with CVDs, classifying conditions according to ICD-10 codes into CVD classes and CVD subclasses. The metabolomic data (325 metabolites from NMR profiling) and comprehensive clinical data (demographics, biochemistry measurements, and blood parameters) were used. The analysis includes differential metabolite expression between CVD and non-CVD participants and machine learning (logistic regression [LR], random forest [RF], and XGBoost [Extreme Gradient Boosting]). The metabolite-linked CVD subtypes and cross-CVD metabolic features were identified. To further study the importance of cross-CVD metabolic features, these features were subsequently validated by reapplying machine learning methods and performing internal geographic hold-out validation using separate geographic cohorts from Scotland and Wales, confirming their robustness and generalizability across different populations.

### Study design and participants

For this study, we excluded participants who lacked NMR data (227,895 individuals) and those with confirmed cancer diagnoses (29,669 participants). This study employed a cross-sectional, case-control design using prevalent CVD diagnoses recorded prior to the baseline assessment date. CVD cases were defined using ICD-10 codes for circulatory system diseases (Supplemental Table 1). Conditions with fewer than 100 cases were excluded to ensure adequate sample sizes and mitigate the risk of extreme model overfitting. The final cohort comprised 244,567 participants (Supplemental Figure 2). As is characteristic of a prevalent disease cohort, participants in the CVD group (n = 27,950) reported substantially higher use of cardiometabolic medications compared to the non-CVD group (n = 216,617), including statins (36.26% vs 6.77%), antiplatelets (25.91% vs 2.25%), and beta-blockers (25.00% vs 3.30%) (see Supplemental Table 4 for full medication details). This polypharmacy is a critical confounding factor considered in the interpretation of our results.

### Metabolomic biomarker quantification and quality control

Metabolic biomarker measurements were conducted in 2 phases: phase 1, from June 2019 to April 2020, and phase 2, from April 2020 to June 2022, using a high-throughput NMR profiling platform (Nightingale Health). A total of 325 metabolic features (Supplemental Table 2), including lipoprotein lipids across different subclasses, fatty acids and their compositions, and various low-molecular-weight metabolites were measured in ethylenediaminetetraacetic acid (EDTA) plasma samples collected at baseline from a randomly selected subset of UKB participants.^16^ Further details on the Nightingale Health NMR biomarker platform and experimental procedures have been previously reported.^18^

### Machine learning methods

Candidate features included NMR metabolomics data consisting of 325 metabolic features, demographic variables (age, sex, body mass index, and hip circumference), biochemical markers (n = 31), routine blood test indicators (n = 30), and urine assay indicators (n = 8). After removing clinical variables with >20% missingness, 66 clinical features were retained (Supplemental Table 3) for the clinical-only and combined models. Metabolomics features were filtered separately using the same missingness threshold, yielding the final metabolite feature matrix used in metabolomics-only models. To align with our goal of mapping metabolic heterogeneity, rather than developing clinically deployable diagnostic models, we evaluated 3 parallel feature sets: 1) metabolites only; 2) clinical only (demographics, anthropometrics, routine biochemistry, hematology, and urinalysis; no NMR variables); and 3) combined (metabolites plus clinical covariates). This allowed us to quantify the cross-sectional discriminative performance of metabolites alone and their potential incremental contribution when added to clinical models. The chosen algorithms were logistic regression,^19^ random forest,^20^ and XGBoost.^21^ Random Forest and XGBoost were selected because their tree-based architecture does not assume normality of predictors and is naturally resilient to skewed distributions and extreme values—common characteristics of NMR-derived metabolomic biomarkers. All preprocessing (median imputation, z-score standardization, class-imbalance handling; detailed descriptions are provided in the Supplemental Methods) was confined to training folds and then applied to the corresponding validation data to avoid information leakage. The logistic regression and random forest models were implemented using the Scikit-learn library in the Python programming language.^22^ XGBoost was used through the xgboost package. All preprocessing was performed within each training fold and then applied to the corresponding validation data to avoid information leakage. For metabolites and clinical variables, features with >20% missing values were excluded from analysis. Missing values in the remaining features were imputed using the median value of the training fold. Given that most NMR-derived metabolic biomarkers exhibit skewed distributions, we applied *z*-score standardization (scaling to mean = 0, SD = 1) based on the training fold statistics to ensure all features were on a comparable scale for model input. Detailed descriptions of class-imbalance handling are provided in the Supplemental Methods.

### Model construction and validation

To characterize metabolic heterogeneity, we developed supervised models (LR, RF, and XGBoost) using a nested 5-fold cross-validation framework for robust hyperparameter optimization and performance estimation. The primary training and tuning were conducted on the England cohort, while participants from Scotland and Wales served as an independent geographic hold-out for validation. Model performance was quantified using receiver operating characteristic (ROC) area under the curve and a comprehensive suite of classification metrics, with 95% CIs derived from bootstrap resampling (1,000 iterations). Detailed descriptions of the algorithmic parameters, hyperparameter search spaces, geographic validation strategy, and performance metric definitions are provided in the Supplemental Materials.

### Model interpretation

To address the “black box” nature of machine learning models, we utilized the SHAP algorithm to perform feature importance analysis on each metabolite. SHAP values provide a way to explain the contribution of each feature to the model’s cross-sectional discrimination, which we used to characterize cross-disease metabolic patterns rather than to infer causality or to build diagnostic tools. For all network visualizations (including the Sankey diagram), we exclusively used SHAP outputs from the combined (metabolites + clinical) models; the metabolite-only and clinical-only pipelines were retained for stability/sensitivity analyses but were not used to construct the Sankey.^23^^,^^24^ This analysis was conducted using the SHAP Python package, which facilitates both global and local interpretability of model decisions.

The top 30 features with the highest SHAP values were selected for further analysis to evaluate their impact on model performance and relevance to CVD classifications. This approach provides insights into which metabolites and clinical features are most influential in distinguishing CVD from non-CVD cases.

### Cross-sectional discriminative performance of cross-CVD metabolic features

To evaluate the cross-sectional discriminative ability of our cross-CVD metabolite features, we constructed cross-sectional discrimination models using only these 21 selected metabolites as input features and evaluated their performance across different CVD classes and subclasses. The models were initially developed and tested on the England cohort, followed by validation on the internal geographic hold-out cohorts from Scotland and Wales to assess generalizability across different populations. These analyses were used to assess the parsimony and stability of cross-CVD signals over and above clinical information, not to develop deployable diagnostic tools.

### Ethics approval and consent

This study analyzed data from the UKB (application number 347405) in accordance with the principles of the Declaration of Helsinki. The UKB protocol was approved by the North West Multi-centre Research Ethics Committee, and all participants provided written informed consent.

## Results

### Study cohorts

In this study, we stratified the UKB cohort into individuals diagnosed with non-CVD participants vs CVD participants according to ICD-10 codes (Methods; Supplemental Table 1). The cohort assembly and exclusions are shown in Supplemental Figure 1. We included a total of 502,131 participants from the UKB data set, recruited between 2006 and 2010. After excluding 227,895 individuals without metabolite data and an additional 29,669 with a history of cancer, the final analysis cohort consisted of 244,567 participants (Supplemental Figure 1). Of these, 128,649 were female and 115,918 were male (Supplemental Figure 2A), and a total of 27,950 participants were diagnosed with prevalent CVDs, while 216,617 individuals were free of CVDs. The sex and age distribution of the cohort are detailed in the Supplemental Material.

### Metabolomic profiling and machine learning classification of CVDs

**Figure 1:**
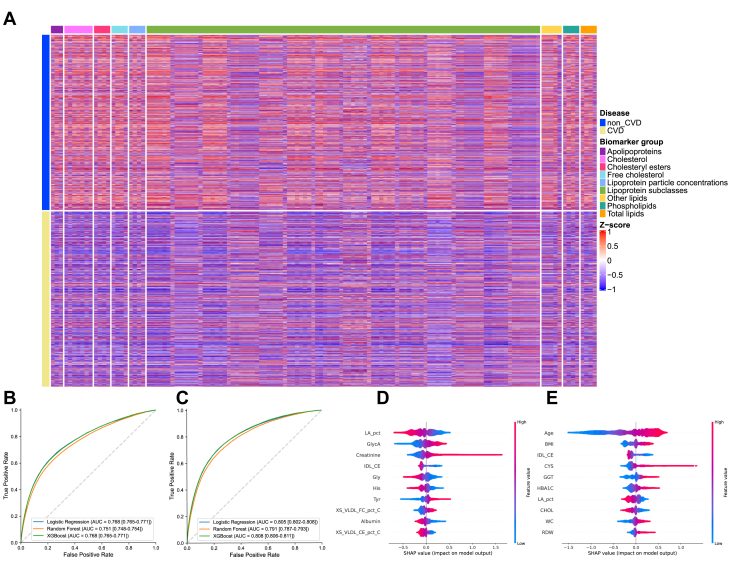
**Metabolomic Profiling and Machine Learning Classification of Cardiovascular Diseases in UK Biobank** (A) Distributional differences of 9 major metabolite groups between non-cardiovascular disease (blue) and cardiovascular disease (yellow) populations, with red indicating up-regulation and blue indicating downregulation. (B and C) Performance comparison of 3 machine learning algorithms (Logistic Regression, Random Forest, and XGBoost) in classifying cardiovascular disease vs non-cardiovascular disease using metabolomics data alone and in combination with clinical parameters. The study included 27,950 cardiovascular disease patients and 216,617 non-cardiovascular disease controls. (D and E) SHapley Additive exPlanations analysis revealing key metabolites and clinical factors driving model performance. AUC = area under the curve; CVD = cardiovascular disease; SHAP = SHapley Additive exPlanations.

To explore metabolomic profiles in non-CVD and CVD participants, the differential patterns of NMR metabolomics between non-CVD (blue) and CVD (yellow) populations across 9 major metabolite groups, including apolipoproteins, cholesterol, cholesteryl esters, free cholesterol, lipoprotein particle concentrations, lipoprotein subclasses, other lipids, phospholipids, and total lipids, were shown in Figure 1B. Red indicates up-regulation, while blue indicates downregulation, with broad shifts across lipid-related measures in CVD cohorts, confirming clear cross-sectional differences in individuals with CVD.

We constructed metabolomics-based discrimination models using 2 approaches: one using metabolic data alone (Figure 1C) and another combining metabolic data with clinical features (Figure 1D). Three machine learning algorithms—LR, RF, and XGBoost—were applied to analyze these data sets. Using metabolomic data alone, the models achieved AUC values of 0.768, 0.751, and 0.768 for LR, RF, and XGBoost on the England cohort, respectively (Figure 1C). Incorporating 65 clinical parameters substantially improved model performance, with the XGBoost model demonstrating the highest AUC of 0.7835 (95% CI: 0.7817-0.7854) (Figure 1D; full performance metrics are detailed in Supplemental Table 6).

The generalizability and robustness of these metabolic patterns were further confirmed through independent validation in geographic hold-out cohorts from Scotland and Wales, with comprehensive performance metrics across all models detailed in the Supplemental Material (Supplemental Tables 7 and 8).

**Figure 2:**
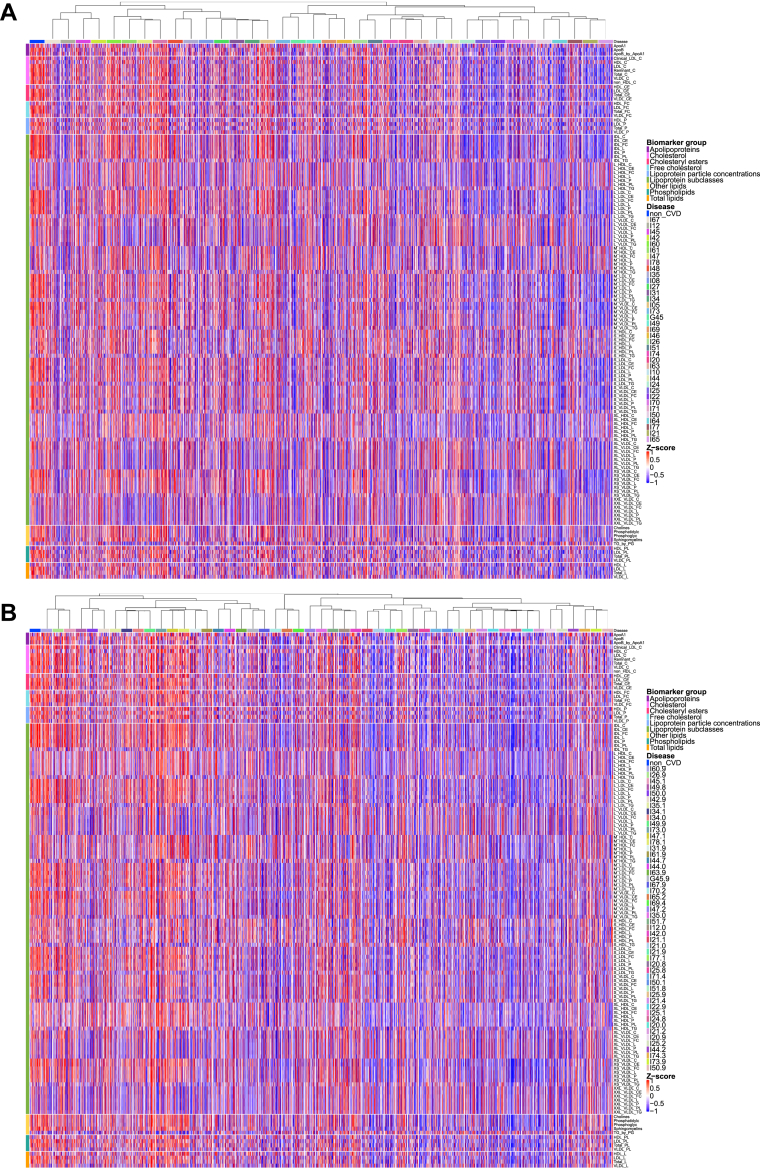
**Global Metabolic Landscape and Interdisease Similarity Heatmaps** (A) Classes. Heatmap of disease-wise mean *z*-scores (cardiovascular disease vs non-cardiovascular disease) for 325 nuclear magnetic resonance features across cardiovascular disease classes. Columns (diseases) are ordered by hierarchical agglomerative clustering (complete linkage) of a composite interdisease similarity S=ωJ+(1−ω)R, where J is the Jaccard overlap of significantly altered metabolites (|meanZ| >0.5) and R is the Spearman rank correlation of disease-specific mean *z*-score profiles rescaled to [0,1]; ω=0.5 in the primary analysis. The top dendrogram summarizes interdisease similarity. Rows are individual features, annotated by biomarker group (right bar). Colors encode mean *z*-scores (red = higher, blue = lower vs non-cardiovascular disease). (B) Subclasses. Same display as panel A but for cardiovascular disease subclasses. Columns (subclasses) are ordered by the same composite similarity *S*. Compared with classes, the subclass map reveals finer-grained heterogeneity, with tight ischemic and hypertensive-renal microclusters and more distinct rheumatic/valvular patterns. For visualization purposes only, participants were randomly sampled for each group (classes, subclasses, and non-cardiovascular disease), with a maximum of 100 individuals per group; all statistical analyses were performed using the full available sample. Abbreviation as in Figure 1.

To identify critical metabolites contributing to model performance, we conducted SHAP analysis as shown in Figures 2E and 2F. Key metabolites contributing to model discrimination included LA_pct (linoleic acid percentage), GlycA, creatinine, IDL_CE (cholesteryl esters in intermediate-density lipoprotein), Gly, His, Tyr, XS_VLDL_FC_pct_C (free cholesterol to cholesterol in very small very low_density lipoprotein (VLDL) percentage), albumin, and XS_VLDL_CE_pct_C (Cholesteryl Esters to Cholesterol in Very Small VLDL percentage). When baseline clinical data were incorporated, age, body mass index, IDL_CE, CYS (cystatin C), GGT (gamma‑glutamyl transferase), HbA1c (glycated hemoglobin), LA_pt, CHOL (cholesterol), WC (waist circumference), and RDW (red cell distribution width) emerged as the most influential factors. Building on the characterization of the study cohort and the identification of key metabolites associated with CVD status, we next aimed to explore the inter-relationships between different CVD entities based on their metabolomic profiles. To this end, we delineated disease-wise metabolomic profiles and organized cardiovascular entities in a classification-independent metabolic similarity space to characterize shared vs disease-specific patterns (Figures 2A and 2B).

### Global metabolomic landscape and interdisease similarity

**Figure 3:**
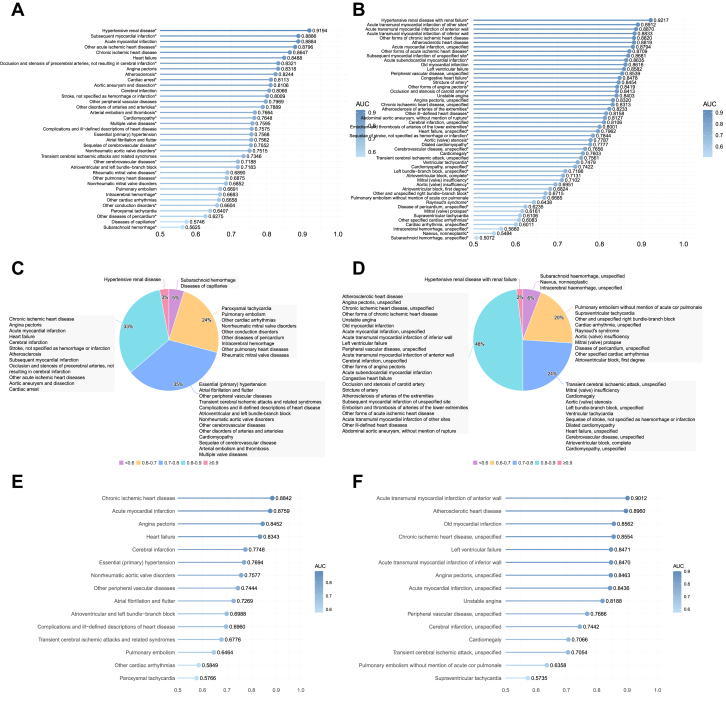
**Metabolomics-Only Discrimination Across Cardiovascular Disease Classes and Subclasses With Internal Geographic Hold-Out Validation** (A) Classes—ranking. Area under the curve values for cross-sectional discrimination of individual cardiovascular disease classes vs non-cardiovascular disease using metabolomics data alone, ranked in descending order (XGBoost; England/discovery). (B) Classes—distribution. Proportion of cardiovascular disease classes across area under the curve ranges for metabolomics-only models (<0.6, 0.6-0.7, 0.7-0.8, 0.8-0.9, >0.9). (C) Classes—internal geographic hold-out validation. Area under the curve values for the same metabolomics-only models evaluated in Scotland + Wales (independent validation cohort, n = 21,961). (D) Subclasses—ranking. Area under the curve values for cross-sectional discrimination of individual CVD subclasses vs non-cardiovascular disease using metabolomics only, ranked in descending order (XGBoost; England/discovery). (E) Subclasses—distribution. Proportion of cardiovascular disease subclasses across area under the curve ranges for metabolomics-only models. (F) Subclasses—internal geographic hold-out validation. Area under the curve values for the same metabolomics-only subclass models in Scotland + Wales. Abbreviation as in Figure 1.

The class-level heatmap ordered by inter-disease similarity reveals clear and reproducible structures shown in Figure 3A. Ischemic entities—acute and subsequent myocardial infarction, chronic ischemic heart disease, angina pectoris, and atherosclerotic disease—form a tight cluster characterized by concordant shifts across intermediate‑density lipoprotein (IDL)/low‑density lipoprotein (LDL)‑related lipid fractions and fatty-acid composition indices. Hypertensive and hypertensive-renal diseases adjoin this block, sharing the lipid-centered background with additional features consistent with cardiorenal coupling. Cerebrovascular diseases (including transient cerebral ischemic attacks) lie proximal to the ischemic cluster but display a less strongly lipid-dominant profile, in keeping with a shared atherosclerotic–inflammatory milieu. In contrast, rheumatic/valvular classes segregate on a separate branch with lower overlap to lipid-driven patterns, while pulmonary embolism is distinctly isolated relative to the other disease classes in the similarity space. Together, these diagnosis-independent structures provide a global scaffold for summarizing shared vs disease-specific metabolic alterations across CVD classes.

At finer granularity, the subclass map preserves and refines the class-level architecture (Figure 2B). Acute myocardial infarction subclasses—acute transmural MI of the anterior and inferior walls and other sites, together with acute MI unspecified and old MI—assemble into a compact microcluster, again marked by coherent changes in lipoprotein composition and fatty-acid fractions. Chronic ischemic heart disease (unspecified) and angina pectoris (unspecified) reside adjacent to this block with similar directions of change but reduced contrast relative to acute entities. Along the hypertensive–renal axis, hypertensive renal disease with renal failure occupies the extreme of the gradient, combining marked lipid/lipoprotein alterations with creatinine-linked signals; other hypertensive subclasses align along the same arm with progressively milder patterns. Heart failure subclasses (eg, left ventricular failure and congestive heart failure) lie near the ischemic block, sharing the lipid-centered background while exhibiting additional differences in selected small-molecule and protein measures. Cerebrovascular subclasses—notably cerebral infarction and occlusion/stenosis of cerebral arteries—cluster together close to the ischemic axis but with a less pronounced lipid signature. Rheumatic/valvular subclasses form a distinct branch with limited overlap to lipid-driven patterns, and pulmonary embolism remains relatively isolated. These subclass-level patterns expose stable ischemic microclusters, a graded hypertensive–renal arm, and distinct rheumatic/valvular trajectories, refining the cardiovascular metabolic landscape established at the class level.

To assess potential confounding by statin therapy, we repeated hierarchical clustering on statin-adjusted and unadjusted residuals and quantified concordance using the Adjusted Rand Index (ARI); concordance was high … (ARI = 0.9138), indicating that the global clustering structure is robust to statin adjustment (Methods).

### Mapping metabolic heterogeneity via machine learning

A systematic characterization of metabolomic signatures across 87 CVD phenotypes, including 37 classes and 50 subclasses, was performed using supervised machine learning algorithms. The discriminative performance varied significantly across the disease spectrum; notably, highly prevalent phenotypes with robust statistical power, such as chronic ischemic heart disease and angina pectoris, demonstrated high and reproducible metabolic separation from non-CVD controls. In contrast, exceptionally high AUC values observed in certain rare, low-event per variable (EPV) conditions were interpreted with caution as potential indicators of model overfitting rather than intrinsic biological distinctiveness, as detailed in the statistical power summaries (Supplemental Table 5). These metabolic patterns were further substantiated through independent geographic hold-out validation using cohorts from Scotland and Wales, which confirmed the generalizability and robustness of the signatures across different populations.

**Figure 4:**
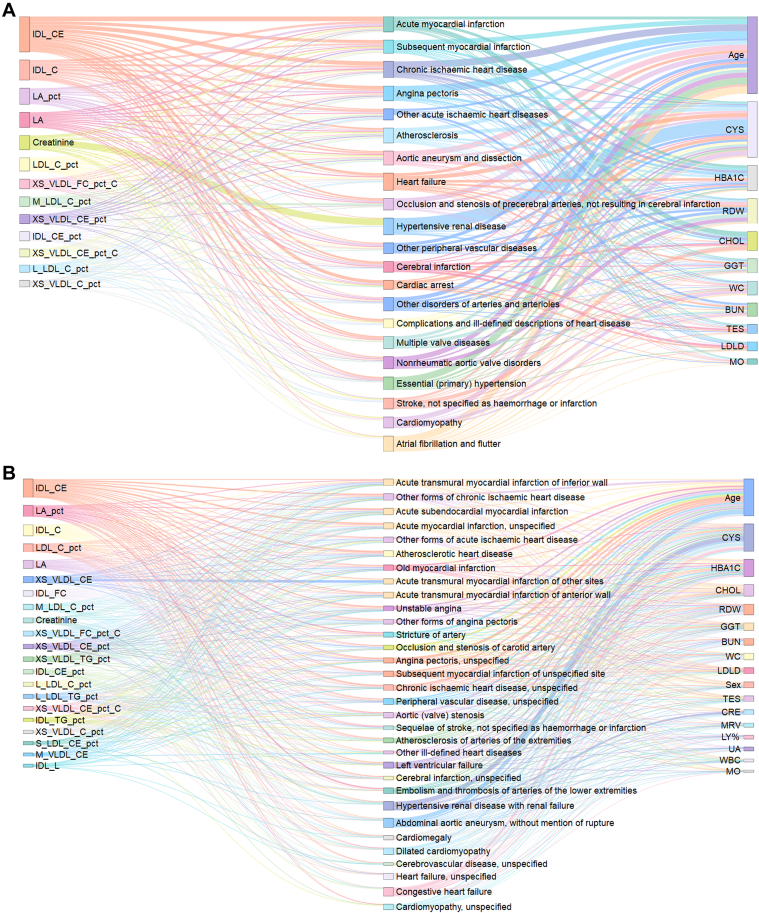
**Sankey Diagram Visualization of Relationships Between Key Metabolites, Clinical Parameters, and Cardiovascular Diseases** (A) Sankey diagram depicting the associations between top-ranked metabolites and clinical parameters with major cardiovascular disease classes. The width of the connecting flows represents the magnitude of SHapley Additive exPlanations values, indicating the strength of the relationship between each feature and disease category. (B) Detailed Sankey diagram showing the connections between key metabolites and clinical parameters with specific cardiovascular disease subclasses. In both diagrams, metabolites are shown in blue, clinical parameters in red, and disease categories in green.

Model performance remained consistent across XGBoost, Logistic Regression, and Random Forest algorithms, with the integration of clinical covariates providing complementary discriminative information to the metabolomics-only models (Supplemental Figures 4 to 10, Supplemental Table 8). SHAP-based feature importance analyses identified a parsimonious set of 21 influential markers consistently associated with multiple CVD subtypes, predominantly centered on lipoprotein subfractions (eg, IDL_CE), fatty acid composition (eg, linoleic acid percentage), and markers of systemic inflammation or hepatic function. Detailed performance metrics, EPV summaries, and comprehensive AUC distributions for all 87 evaluated phenotypes are relocated to the Supplemental Results and illustrated in Figure 4.

### Identification of cross-CVD metabolite features and metabolite-linked CVD subtypes

To investigate the metabolic commonalities and differences across CVDs, we employed a systematic approach focusing on CVD classifications with pronounced metabolic alterations (AUC ≥0.8). Differential expression analysis confirmed significant metabolic alterations across disease states, with consistent patterns of metabolic dysregulation across multiple CVD classifications demonstrating shared metabolic perturbations (Figure 3B, Supplemental Figure 6). The complex relationships between identified metabolic features and CVD categories were visualized through Sankey diagram analysis (Figure 4).

Our comprehensive analysis identified 2 novel conceptual frameworks: cross-CVD metabolite features: we identified 21 key metabolites that consistently demonstrated high discriminative importance across multiple cardiovascular conditions, predominantly associated with lipoprotein metabolism. Metabolite-linked CVD subtypes: We characterized specific cardiovascular conditions exhibiting particularly strong associations with distinct metabolic signatures, suggesting they possess distinctive metabolic fingerprints (Figures 4A and 4C). This framework provides a basis for comparing subtype-specific patterns and understanding metabolic heterogeneity within the CVD spectrum.

### Classifications of cross-CVD metabolite features

To evaluate the discriminative capacity of our identified cross-CVD metabolite features, we constructed models using only these selected metabolites as input features. Supplemental Figure 11 presents the metabolic signature distinctiveness for the top 15 CVD classes and subclasses in the England cohort. For CVD classes (Supplemental Figure 11A), the models demonstrated particularly pronounced metabolic alterations for chronic ischemic heart disease (AUC = 0.8762), acute myocardial infarction (AUC = 0.8739), heart failure (AUC = 0.8346), and angina pectoris (AUC = 0.8329). At the subclass level (Supplemental Figure 11B), the most distinct metabolic signatures were observed for acute transmural myocardial infarction of anterior wall (AUC = 0.8931), atherosclerotic heart disease (AUC = 0.8861), acute myocardial infarction, unspecified (AUC = 0.8597), and left ventricular failure (AUC = 0.8517). Performance details are provided in Supplemental Tables 9 and 10.

Analysis of the metabolic signature distribution across all disease categories (Supplemental Figures 11C and 11D) revealed that 33% of CVD classes and 48% of CVD subclasses showed highly distinct metabolic profiles (AUC >0.8), again noting the contribution of low-EPV, overfit models to this percentage.

**Figure 5:**
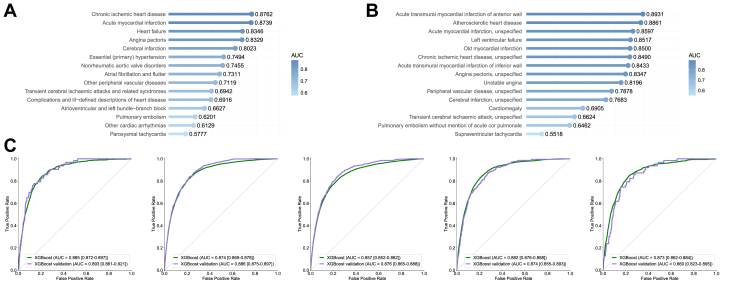
**Evaluation of Cross-Sectional Discriminative Patterns Using Selected Metabolites Across Cardiovascular Disease Classes and Subclasses** (A) Area under the curve values for the top 15 cardiovascular disease classes in the England cohort (n = 222,606), ranked from highest to lowest performance. (B) Area under the curve values for the top 15 cardiovascular disease subclasses in the England cohort. (C) Comprehensive area under the curve values for all cardiovascular disease classes in the internal geographic hold-out validation cohort (Scotland and Wales), demonstrating robust generalizability of metabolite-based cross-sectional discrimination. Abbreviation as in Figure 1.

Crucially, these findings from the parsimonious top-30 feature model were comparable to our comprehensive models using all available features. This strongly suggests that the essential metabolic information distinguishing prevalent CVD from non-CVD is highly concentrated within this core set of features, primarily those related to lipoprotein metabolism. Validation on the internal geographic hold-out cohorts (Figures 5A and 5B) demonstrated consistent metabolic patterns. While hypertensive renal disease again showed a high AUC (0.9078), this finding is considered unreliable due to the small validation sample size (n = 37). More robustly, subsequent myocardial infarction (AUC = 0.8923) and acute myocardial infarction (AUC = 0.8823) showing the most pronounced metabolic alterations among CVD classes.

Direct comparison between development and internal geographic hold-out cohorts for the top 5 diseases (Figure 5C) demonstrated remarkable consistency in metabolic signature patterns across populations, with only minor variations. These findings are collectively consistent with the 21 cross-CVD metabolite features and underscore their value as a compact and biologically coherent feature set capturing shared metabolic perturbations across diverse cardiovascular phenotypes.

## Discussion

**Figure 6:**
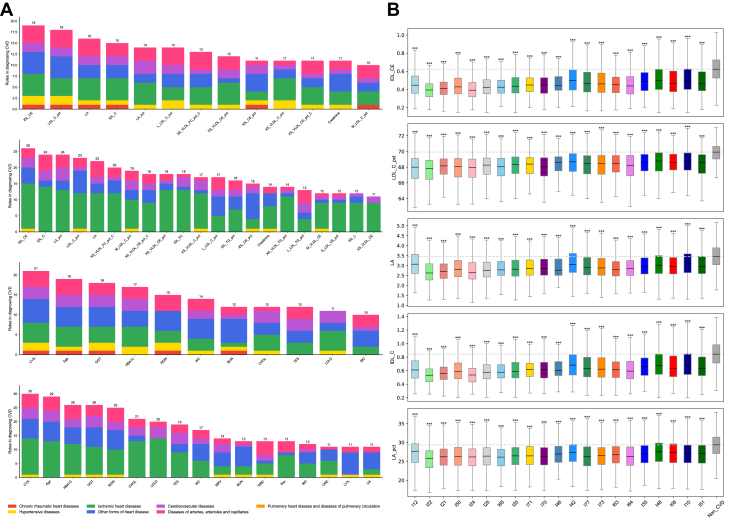
**Identification and Validation of Consistent Metabolic and Clinical Predictors Across Cardiovascular Disease Spectrum** (A) Frequency distribution of top-ranked features identified by SHAP analysis in at least 10 different cardiovascular conditions, stratified by feature type (metabolites or clinical parameters) and disease cross-sectional discrimination level (cardiovascular disease classes or subclasses). Color coding represents different cardiovascular disease categories: chronic rheumatic heart diseases (dark red), hypertensive diseases (yellow), ischemic heart diseases (green), other forms of heart disease (blue), cerebrovascular diseases (purple), diseases of arteries, arterioles and capillaries (magenta), and pulmonary heart disease and diseases of pulmonary circulation (orange). (B) Box plots showing the distribution of the 5 most frequently recurrent metabolites (IDL_CE, IDL_C, LA_pct, LDL_C_pct, and LA) across different cardiovascular disease classes compared to non-cardiovascular disease controls. (B) Statistical comparisons between each cardiovascular disease subclass and the non-cardiovascular disease control group were performed using the 2-sided Mann-Whitney *U* test. The resulting *P* values were adjusted for multiple comparisons using the Benjamini-Hochberg false discovery rate procedure. Asterisks indicate the level of significance based on these adjusted *P* values: ∗*P* < 0.05, ∗∗*P* < 0.01, and ∗∗∗*P* < 0.001. Abbreviation as in Figure 1.

In this UKB analysis, we mapped metabolomic variation across the CVD spectrum to characterize shared vs disease-specific patterns (Figure 1, Figure 2, Figure 3, Figure 4, Figure 6). While the prominence of atherogenic lipoproteins in coronary disease is well-established, the core contribution of this work lies in the systematic organization of 87 CVD phenotypes. This “metabolomic cartography” reveals reproducible clustering structures that transcend traditional clinical classifications. Furthermore, our parsimonious 21-feature set provides an organizational scaffold, demonstrating that the vast majority of shared cardiovascular metabolic signals are concentrated within a limited number of biologically coherent pathways.

First, disease-wise similarity maps revealed reproducible organization. Ischemic entities—acute and subsequent myocardial infarction, chronic ischemic heart disease, angina, and atherosclerotic disease—clustered together and were marked by concordant shifts in IDL/LDL-related fractions and fatty-acid composition indices (Figures 2A and 2B), consistent with established atherogenic biology and endothelial dysfunction pathways.^25^, ^26^, ^27^, ^28^, ^29^ Hypertensive and hypertensive-renal diseases aligned adjacent to this block with additional creatinine-linked signals, consistent with heart–kidney interactions (Figure 2B).^30^, ^31^, ^32^ Hypertensive renal disease with renal failure occupied the extreme of this gradient, (Figure 2B) in line with its strong (though likely influenced by limited statistical power) distinctiveness in single-disease analyses (Figures 3A and 3D),^33^ a point we address further below. Cerebrovascular diseases lay proximal to the ischemic cluster yet showed a less lipid-dominant profile (Figures 2A and 2B), concordant with shared atherothrombotic processes across vascular beds.^34^^,^^35^ By contrast, rheumatic/valvular diseases and pulmonary embolism were comparatively separated (Figures 2A and 2B), aligning with mechanisms less dependent on systemic dysmetabolism.^36^^,^^37^ The global structure persisted after statin adjustment (Adjusted Rand Index 0.9138), indicating robustness to this specific medication class, while not excluding confounding from other commonly prescribed cardiovascular therapies (Methods; Figures 2A and 2B).^38^^,^^39^

Second, the degree of metabolic distinctiveness varied substantially across CVD classes and subclasses, which we interpreted through the lens of per-disease AUCs. Our analysis distinguished between signals from rare conditions with limited statistical power and those from high-prevalence phenotypes with robust support. Crucially, patterns observed in common, high-EPV phenotypes were stable across internal geographical hold-out testing and were preserved in parsimonious models using only the key metabolite features, suggesting these associations may represent reproducible cross-sectional metabolic patterns rather than methodological artifacts or model overfitting.

Third, feature-importance analyses consistently highlighted a lipid-centered and fatty-acid–related signal, accompanied by inflammatory, glycemic, renal, and hepatic markers. Recurrent robust features included IDL_CE, IDL_C, LDL_C_pct, small-VLDL ratios (eg, XS_VLDL_FC_pct/CE_pct), LA_pct, GlycA, HbA1c, creatinine, and GGT (Figures 1E and 1F, Supplemental Figures 6C and 6F). The prominence of LA-related metrics accords with links to macrophage cholesterol handling, mitochondrial energetics, and inflammatory signaling, while small, dense apoB-containing lipoproteins provide a plausible basis for the metabolic proximity of acute and chronic coronary entities.^26^^,^^28^^,^^29^^,^^40^, ^41^, ^42^, ^43^, ^44^, ^45^ Directional differences relative to non-CVD were coherent for the top metabolites (Figures 4B and 4C). The importance of this core metabolic feature set was further supported by our parsimonious model analysis (Figure 5). Here, “cross-CVD metabolite features” refer to the 21 metabolites that recurrently ranked among the top contributors across multiple CVD classes and subclasses, showed consistent directionality, and remained stable across modeling strategies and geographic validation. A compact model using only these 21 metabolites (the “cross-CVD” set) preserved the vast majority of the cross-sectional discriminative performance observed in the full 325-feature model. This finding suggests that shared metabolic signals across CVDs are concentrated within a limited number of biologically coherent pathways, notably atherogenic lipoprotein metabolism and inflammation.

These observations refine an atlas of metabolomic heterogeneity. Heart failure subclasses neighbored ischemic entities yet showed additional small molecule and protein differences; cerebrovascular subclasses lay near—but not within—the lipid-dominant core; rheumatic/valvular diseases and pulmonary embolism remained more metabolically independent on this NMR panel (Figures 2 and 3).^34^, ^35^, ^36^, ^37^ Taken together, the results highlight convergent lipid/fatty-acid perturbations for coronary phenotypes, a graded hypertensive–renal arm with creatinine-linked features, and entities with weaker systemic metabolic signatures on this platform.

Methodologically, we summarized disease–disease proximity with a composite similarity metric to move beyond qualitative comparisons; alternative normalizations or distances could modify local topology.^46^^,^^47^ The dominant patterns were consistent across standard classifiers and regions, and adding clinical variables contributed complementary—not redundant—information (Figures 1D, 3C, 3F, Central Illustration, Supplemental Figures 6A to 6E). All analyses are descriptive of metabolic covariation and should not be interpreted as evidence of causality, and extensions to other omics and disease domains should be tested empirically.^48^^,^^49^

**Central Illustration:**
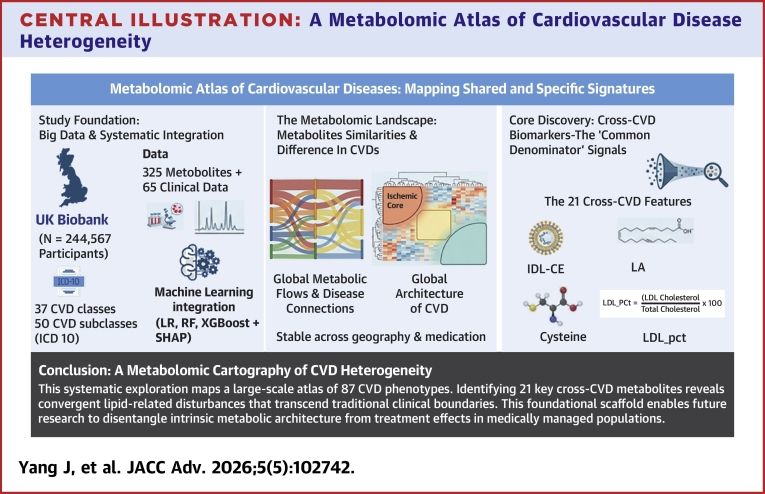
**A Metabolomic Atlas of Cardiovascular Disease Heterogeneity** This systematic exploration leverages the UK Biobank (N = 244,567) and machine learning to map the metabolic architecture across 87 cardiovascular disease phenotypes. The resulting “metabolomic cartography” identifies 21 core “cross-cardiovascular disease” features—primarily convergent lipid-related disturbances—that remain robust across independent geographic cohorts and major medication classes. By uncovering diagnosis-independent structures like the “Ischemic Core,” this foundational scaffold provides a reproducible landscape to disentangle intrinsic metabolic signals from treatment effects in medically managed populations. ICD-10 = International Classification of Diseases-10th Revision; IDL_CE = cholesteryl esters in intermediate-density lipoprotein; LA = linoleic acid; SHAP = SHapley Additive exPlanations; other abbreviation as in Figure 1.

A central challenge in cross-sectional analyses of prevalent CVD is confounding by medication use, as the observed metabolomic profiles reflect a composite of disease biology and long-term pharmacological exposure. Cardiovascular populations are frequently exposed to lipid-lowering, antihypertensive, antidiabetic, and antithrombotic therapies, all of which are known to substantially modify circulating metabolite levels, particularly lipoprotein subfractions and fatty-acid measures. To mitigate this, we incorporated statin use into multivariable models and conducted dedicated sensitivity analyses, including statin-adjusted residual analyses and restriction to statin-naïve subsets. Key lipid- and fatty-acid–related patterns remained qualitatively consistent after statin adjustment, supporting the robustness of the major structures identified.

Nevertheless, complete disentanglement of disease-related and treatment-related metabolic effects is not feasible in observational cohorts of treated patients. Accordingly, the metabolic signatures described here are best interpreted as reflecting the composite metabolic phenotype of contemporary, real-world, medically managed CVD populations, rather than intrinsic disease biology in treatment-naïve states. This perspective is clinically relevant, as it mirrors the metabolic landscape encountered in routine practice, but caution is warranted when extrapolating these findings to early or untreated disease stages.

Given the scale of this analysis, multiple testing represents an important methodological consideration. Hundreds of metabolites were evaluated across dozens of CVD classes and subclasses, resulting in a high-dimensional testing framework. False discovery rate control was applied across all metabolite–disease comparisons, but statistically significant findings may still include false positives, particularly for less prevalent disease categories with limited effective sample size.

To reduce overinterpretation, our primary biological inferences were intentionally restricted to metabolic features that demonstrated reproducibility across multiple dimensions, including consistency across CVD subtypes, stability across different modeling approaches, and replication in internal geographic hold-out cohorts. Findings specific to rare subtypes or low-EPV models should therefore be regarded as exploratory and hypothesis-generating, rather than definitive.

### Study Limitations

Several major limitations must be emphasized as they frame the interpretation of this study. First, the UKB participants are predominantly of European ancestry, which may limit the direct generalizability of our metabolic patterns to other ethnic groups. In addition, UKB participants are generally healthier and better educated than the general population, which may influence both disease spectrum and metabolic profiles. The use of prevalent CVD cases further introduces survivor bias, as individuals with severe or rapidly fatal disease are less likely to be represented at baseline. Consequently, the observed metabolic signatures may preferentially reflect CVD phenotypes compatible with longer survival and study participation, rather than the full spectrum of disease severity present in the general population. Second, residual confounding from unmeasured comorbidities, lifestyle factors, or environmental exposures cannot be excluded. Third, the cross-sectional design precludes causal inference. The metabolic profiles identified here likely reflect a composite of disease processes, physiological adaptation, and long-term treatment exposure, rather than early disease drivers. Finally, while the NMR platform provides broad coverage of lipid-related metabolism, it does not capture the full metabolome; future longitudinal studies integrating mass spectrometry–based metabolomics and other omics layers will be important to further resolve dynamic and causal relationships. Additionally, although we employed robust methods like median imputation and tree-based models to handle missing data and non-normal distributions, we cannot entirely rule out residual bias from these preprocessing steps; sensitivity analyses exploring different imputation strategies or model assumptions could further validate the robustness of the observed patterns.

## Conclusions

Our study establishes a large-scale metabolomic atlas—a systematic cartography rather than mechanistic dissection—of prevalent CVD. We systematically mapped both shared and subtype-specific metabolic alterations, identifying a convergent set of metabolic disturbances particularly in lipid-related pathways. While the interpretive scope is shaped by the cross-sectional design and inclusion of treated patients, the findings provide a foundational resource for generating new biological hypotheses. Future studies in prospective, treatment-naive cohorts are required to further distill the intrinsic metabolic architecture of CVDs and to distinguish pathophysiological drivers from treatment effects.

## Funding support and author disclosures

This work was supported by the 10.13039/501100012166National Key R&D Program of China (2022YFC2704300 and 2021ZD0201300), the 10.13039/100014717National Natural Science Foundation of China (32400532), the Fujian Science and Technology Program Guiding Project (2025D022), the 10.13039/501100017686Fujian Provincial Health Technology Project (2024GGB18), and the 10.13039/501100003392Natural Science Foundation of Fujian Province, China (Grant No. 2025J08313). The funders had no role in the design and conduct of the study; collection, management, analysis, and interpretation of the data; preparation, review, or approval of the manuscript; and decision to submit the manuscript for publication. The authors have reported that they have no relationships relevant to the contents of this paper to disclose.

## References

[1] Joseph P., Lanas F., Roth G. (2025). Cardiovascular disease in the Americas: the epidemiology of cardiovascular disease and its risk factors. Lancet Reg Health Am.

[2] World Health Organization (2024).

[3] Zhu C., Li L., Zhao M. (2025). Risk of premature cardiovascular disease and all-cause mortality in young adults, association with risk factor prevalence early in life. BMC Cardiovasc Disord.

[4] Cao X., Wang T., Mu G. (2025). Dysregulated homocysteine metabolism and cardiovascular disease and clinical treatments. Mol Cell Biochem.

[5] Karlstaedt A., Moslehi J., de Boer R.A. (2022). Cardio-onco-metabolism: metabolic remodelling in cardiovascular disease and cancer. Nat Rev Cardiol.

[6] Back M., Yurdagul A., Tabas I., Oorni K., Kovanen P.T. (2019). Inflammation and its resolution in atherosclerosis: mediators and therapeutic opportunities. Nat Rev Cardiol.

[7] Hong K.N., Fuster V., Rosenson R.S., Rosendorff C., Bhatt D.L. (2017). How low to Go with glucose, cholesterol, and blood pressure in primary prevention of CVD. J Am Coll Cardiol.

[8] Arsenault B.J., Pibarot P., Despres J.P. (2009). The quest for the optimal assessment of global cardiovascular risk: are traditional risk factors and metabolic syndrome partners in crime?. Cardiology.

[9] Clendenen N., D'Alessandro A. (2018). High throughput metabolomics in clinical studies: review and new applications to remote ischemic preconditioning. Curr Top Med Chem.

[10] Newgard C.B. (2017). Metabolomics and metabolic diseases: where do we stand?. Cell Metab.

[11] Buergel T., Steinfeldt J., Ruyoga G. (2022). Metabolomic profiles predict individual multidisease outcomes. Nature Med.

[12] Witkowski M., Nemet I., Li X.S. (2024). Xylitol is prothrombotic and associated with cardiovascular risk. Eur Heart J.

[13] Zhang L., Wei T.T., Li Y. (2018). Functional metabolomics characterizes a key role for N-Acetylneuraminic acid in coronary artery diseases. Circulation.

[14] Kronenberg F., Mora S., Stroes E.S.G. (2022). Lipoprotein(a) in atherosclerotic cardiovascular disease and aortic stenosis: a European atherosclerosis society consensus statement. Eur Heart J.

[15] Vignoli A., Tenori L., Giusti B. (2019). NMR-based metabolomics identifies patients at high risk of death within two years after acute myocardial infarction in the AMI-Florence II cohort. BMC Med.

[16] UK Biobank (2024).

[17] Wang Z., Gu Y., Huang L. (2024). Construction of machine learning diagnostic models for cardiovascular pan-disease based on blood routine and biochemical detection data. Cardiovasc Diabetol.

[18] Julkunen H., Cichonska A., Tiainen M. (2023). Atlas of plasma NMR biomarkers for health and disease in 118,461 individuals from the UK Biobank. Nat Commun.

[19] Vetter T.R., Schober P. (2018). Regression: the apple does not fall far from the tree. Anesth Analg.

[20] Hu J., Szymczak S. (2023). A review on longitudinal data analysis with random forest. Brief Bioinform.

[21] Yu Y., Tran H. (2022).

[22] Pedregosa F., Varoquaux G., Gramfort A. (2011). Scikit-learn: Machine Learning in Python. J Mach Learn Res.

[23] Crombe A., Kataoka M. (2024). Breast cancer molecular subtype prediction: improving interpretability of complex machine-learning models based on multiparametric-MRI features using SHapley Additive exPlanations (SHAP) methodology. Diagn Interv Imaging.

[24] Zhang Y., Xiang T., Wang Y. (2024). Explainable machine learning for predicting 30-day readmission in acute heart failure patients. iScience.

[25] Cheng S., Rhee E.P., Larson M.G. (2012). Metabolite profiling identifies pathways associated with metabolic risk in humans. Circulation.

[26] Ikon N., Ryan R.O. (2017). Cardiolipin and mitochondrial cristae organization. Biochim Biophys Acta Biomembr.

[27] Edfeldt K., Swedenborg J., Hansson G.K., Yan Z.Q. (2002). Expression of toll-like receptors in human atherosclerotic lesions: a possible pathway for plaque activation. Circulation.

[28] Tabas I. (2010). The role of endoplasmic reticulum stress in the progression of atherosclerosis. Circulation Res.

[29] Tabas I. (2005). Consequences and therapeutic implications of macrophage apoptosis in atherosclerosis: the importance of lesion stage and phagocytic efficiency. Arterioscler Thromb Vasc Biol.

[30] Hotamisligil G.S. (2017). Inflammation, metaflammation and immunometabolic disorders. Nature.

[31] Connaughton R.M., McMorrow A.M., McGillicuddy F.C., Lithander F.E., Roche H.M. (2016). Impact of anti-inflammatory nutrients on obesity-associated metabolic-inflammation from childhood through to adulthood. Proc Nutr Soc.

[32] Akinkuolie A.O., Buring J.E., Ridker P.M., Mora S. (2014). A novel protein glycan biomarker and future cardiovascular disease events. J Am Heart Assoc.

[33] Duprez D.A., Otvos J., Sanchez O.A., Mackey R.H., Tracy R., Jacobs D.R. (2016). Comparison of the predictive value of GlycA and other biomarkers of inflammation for total death, incident cardiovascular events, noncardiovascular and noncancer inflammatory-related events, and total cancer events. Clin Chem.

[34] Marklund M., Wu J.H.Y., Imamura F. (2019). Biomarkers of dietary Omega-6 fatty acids and incident cardiovascular disease and mortality. Circulation.

[35] Harris W.S., Mozaffarian D., Rimm E. (2009). Omega-6 fatty acids and risk for cardiovascular disease: a science advisory from the American Heart Association Nutrition Subcommittee of the Council on Nutrition, Physical Activity, and Metabolism; Council on Cardiovascular Nursing; and Council on Epidemiology and Prevention. Circulation.

[36] Ronco C., Haapio M., House A.A., Anavekar N., Bellomo R. (2008). Cardiorenal syndrome. J Am Coll Cardiol.

[37] House A.A., Wanner C., Sarnak M.J. (2019). Heart failure in chronic kidney disease: conclusions from a Kidney Disease: Improving Global Outcomes (KDIGO) controversies conference. Kidney Int.

[38] Valderas J.M., Starfield B., Sibbald B., Salisbury C., Roland M. (2009). Defining comorbidity: implications for understanding health and health services. Ann Fam Med.

[39] Tinetti M.E., Fried T.R., Boyd C.M. (2012). Designing health care for the most common chronic condition--multimorbidity. JAMA.

[40] Moore K.J., Sheedy F.J., Fisher E.A. (2013). Macrophages in atherosclerosis: a dynamic balance. Nat Rev Immunol.

[41] Tabas I., García-Cardeña G., Owens G.K. (2015). Recent insights into the cellular biology of atherosclerosis. J Cell Biol.

[42] Claypool S.M., Koehler C.M. (2012). The complexity of cardiolipin in health and disease. Trends Biochem Sci.

[43] Michelsen K.S., Wong M.H., Shah P.K. (2004). Lack of toll-like receptor 4 or myeloid differentiation factor 88 reduces atherosclerosis and alters plaque phenotype in mice deficient in apolipoprotein E. Proc Natl Acad Sci U S A.

[44] Rhee E.P., Gerszten R.E. (2012). Metabolomics and cardiovascular biomarker discovery. Clin Chem.

[45] Feinstein A.R. (1970). The pre-therapeutic classification OF CO-morbidity in chronic disease. J Chronic Dis.

[46] Boehme A.K., Esenwa C., Elkind M.S. (2017). Stroke risk factors, genetics, and prevention. Circ Res.

[47] O'Donnell M.J., Chin S.L., Rangarajan S. (2016). Global and regional effects of potentially modifiable risk factors associated with acute stroke in 32 countries (INTERSTROKE): a case-control study. Lancet (London, England).

[48] Würtz P., Mäkinen V.P., Soininen P. (2012). Metabolic signatures of insulin resistance in 7,098 young adults. Diabetes.

[49] Sliz E., Kettunen J., Holmes M.V. (2018). Metabolomic consequences of genetic inhibition of PCSK9 compared with statin treatment. Circulation.

