# Supplementary material for: Metabolomic Atlas of Cardiovascular Diseases: Mapping Shared and Specific Signatures
Source: JACC Adv. 2026 Apr 17;5(5):102742. doi: 10.1016/j.jacadv.2026.102742 (PMC13098572; doi:10.1016/j.jacadv.2026.102742)
Supplement: Supplemental Material [file mmc1.pdf]

Metabolomic Atlas of Cardiovascular Diseases: Mapping Shared and Specific Signatures

Jingjing Yang MD[2†], Wanshan Ning PhD[1†], Ruizhi Xu MD[1,4], Yaping Guo PhD[5], Amei He MD[1], Jiajun Fan MD[6], Yanbo Wang PhD[4], Xingyu Li PhD[1*], Qun Chen PhD[1,3*]

[1]Institute of Clinical Medical Research, the First Affiliated Hospital, School of Medicine, Xiamen University, Xiamen, Fujian 361003, China.

[2]Department of Pulmonary and Critical Care Medicine, the First Affiliated Hospital of Xiamen University, School of Medicine, Xiamen University, Xiamen, Fujian 361003, China.

[3]Xiamen Cell Therapy Research Center, The First Affiliated Hospital of Xiamen University, School of Medicine, Xiamen University, Xiamen, China.

[4]Nanjing Drum Tower Hospital Center of Molecular Diagnostic and Therapy, Chinese Academy of Medical Sciences Research Unit of Extracellular RNA, State Key Laboratory of Pharmaceutical Biotechnology and Department of Physiology, Jiangsu Engineering Research Center for MicroRNA Biology and Biotechnology, NJU Ad vanced Institute of Life Sciences (NAILS), School of Life Sciences, Nanjing University, 163 Xianlin Avenue, Nanjing 210023, Chia.

[5]Department of Pathophysiology, School of Basic Medical Sciences, Zhengzhou University, Zhengzhou, Henan 450001, China.

[6]Siebel School of Computing and Data Science, University of Illinois Urbana-Champaign.

[7]Department of Hepatopancreatobiliary Surgery, the Third Xiangya Hospital, Central South University, Changsha, 410013, Hunan, China.

[†] Jingjing Yang and Wanshan Ning, contributed equally to this work.

[*]Correspondence: Xingyu Li, The First Affiliated Hospital of Xiamen University, 55 Zhenhai Road, Xiamen, Fujian, China 361003, E-mail:xingyuli22@yeah.net, Fax：0592-2137189; Qun Chen, The First Affiliated Hospital of Xiamen University, 55 Zhenhai Road, Xiamen, Fujian, China 361003,E-mail:chenquns@xmu.edu.cn, Fax：0592-2137189.

Supplementary Materials

Methods

Study Design and Participants

The UKB is a large-scale, prospective study that enrolled over 500,000 UK residents aged 37 to 73 years from 2006 to 2010, with follow-up assessments conducted thereafter. The methodology of the study has been described in detail in previous reports(1,2). The UKB study has received ethical approval from the National Information Governance Board for Health and Social Care and the NHS North West Multicentre Research Ethics Committee (11/NW/0382), and all participants provided written informed consent prior to participation. Additional project-specific ethical approval is not required.

Metabolomic Biomarker Quantification and Quality Control

All metabolite concentrations were standardized using the z-score normalization (StandardScaler function from scikit-learn). This process scaled the data to have a mean of zero and a standard deviation of one, ensuring that all features were on a comparable scale(3). Features with more than 20% missing values were excluded from the analysis. Remaining missing values were imputed using the median value of each feature, as it is less sensitive to outliers compared to mean imputation(4). All preprocessing steps (median imputation and standardization) were performed within training folds only and subsequently applied to validation data to avoid information leakage.

Class-Imbalance Handling

To address the class-imbalance across CVD subtypes, we adopted a fold-aware, two-step strategy. First, the Synthetic Minority Over-sampling Technique (SMOTE, k_neighbors = 5, sampling_strategy='auto', random_state=42) was applied within each training fold only to

generate synthetic samples for minority subtypes by linear interpolation among k nearest minority neighbours. Second, algorithm-specific class weighting was enabled during model fitting.For XGBoost we set $\text{scale\_pos\_weight} = \frac{N\text{negative}}{N\text{positive}}$ (computed per fold), where *N*negative and *N*positive denote the numbers of negative (non-CVD) and positive (CVD or specific subtype) samples, respectively. This weighting scheme increases the minority class's contribution to the objective function, thereby directing the algorithm to prioritise its accurate classification. For Logistic Regression and Random Forest, we enabled class_weight='balanced', which automatically assigns class weights inversely proportional to class frequencies in the training data. In practice, minority classes receive higher weights, while majority classes are down-weighted, preventing the model from being dominated by the abundant non-CVD samples and improving minority-class recognition.

Together, SMOTE and class-weighting reduced misclassification of rare subtypes while preserving a leakage-free evaluation protocol.

Drug-Effect Adjustment Procedure

Given the high prevalence of polypharmacy in the CVD cohort (Table S4), we conducted a specific sensitivity analysis to account for confounding by statin therapy, one of the most common treatments. To account for potential confounding by statin therapy, we adopted a three-step adjustment strategy. First, metabolite concentrations were regressed on age, sex, BMI, and statin use; residuals from this model served as statin-adjusted metabolite values. This procedure was used as a sensitivity analysis to assess robustness of clustering and discrimination patterns, not to estimate causal treatment effects. A parallel regression on

age, sex, and BMI alone generated residuals in which statin effects were retained. Both residual matrices were subsequently Z-score standardised to ensure comparability. Second, to assess whether statin adjustment altered the sample clustering structure, we applied agglomerative hierarchical clustering (identical distance metric and linkage across specifications) to each residual matrix, cut the dendrograms to yield the same number of clusters k, and quantified partition concordance using the Adjusted Rand Index (ARI) from sklearn.metrics.adjusted_rand_score. cl_full_for_ari and cl_nostatin_for_ari denote the cluster-label vectors from the statin-adjusted and unadjusted analyses, respectively; the ARI was computed as adjusted_rand_score, which measures agreement corrected for chance (range −1 to 1). Third, to evaluate model performance in a cohort with reduced pharmacological confounding, individuals reporting statin use were excluded from the training set and all downstream analyses were repeated on this statin-naïve subset. We acknowledge that this analysis only formally adjusts for statins, while other prevalent medications (e.g., antihypertensives, antidiabetics, as shown in Table S4) represent significant unadjusted confounders, a limitation that is addressed in the Discussion.

Differential Metabolite Analysis

Because metabolite distributions deviated from normality, we compared each disease category with the non-CVD group using two-sided Mann–Whitney U tests. For every metabolite-by-comparison test, we reported distributional summaries (median and IQR) and effect sizes (Hodges–Lehmann median difference with 95% CI and/or Cliff's delta). To control multiplicity across all metabolite × disease comparisons, p-values were adjusted using

Benjamini–Hochberg false discovery rate (FDR); unless otherwise specified, "significance" refers to FDR-adjusted p-values (q-values). Box plots were annotated using q-values with the conventional star notation: *** $q < 0.001$, ** $q < 0.01$, * $q < 0.05$. This approach helped validate the SHAP-selected metabolites and their relevance in distinguishing CVD subtypes from non-CVD cases.

Disease–Disease Metabolic Similarity Analysis

To quantify inter-disease similarity in metabolomic perturbations, we combined two complementary measures derived from disease-specific differential profiles. First, for each CVD entity we defined a set of significantly altered metabolites as those with an absolute mean Z-score > 0.5 (computed on standardised metabolite values). The Jaccard index between two diseases,$J = |A \cap B|/|A \cup B|$, captured overlap in which metabolites change. Second, we computed the Spearman rank correlation between the vectors of disease-specific mean Z-scores (ranked within disease), capturing concordance in how metabolites change (i.e., the pattern of up-/down-regulation). The correlation was linearly rescaled to [0,1] as $R=(\rho+1)/2$ prior to aggregation.

We defined a composite similarity as a convex combination of the two measures, $S = \omega J + (1 - \omega)R$, S is a convex combination balancing set overlap and pattern concordance; ω tunes their relative influence while preserving boundedness and comparability across disease pairs. In the primary analysis we set $\omega=0.5$; sensitivity analyses varying ω from 0.25 to 0.75 yielded qualitatively similar structures. The corresponding dissimilarity was defined as D=1-S and subjected to hierarchical

agglomerative clustering (complete linkage) to generate dendrograms and heatmaps that organise diseases by shared metabolic patterns.

Machine Learning Methods

Rather than building deployable diagnostic tools, supervised learners were used to prioritise and stabilise features and to quantify how clinical covariates modulate metabolite–disease associations across subtypes. The supervised comparison was constructed using prevalent CVD diagnoses recorded prior to the baseline date. Individuals were considered to have prevalent CVDs if they had been diagnosed with the specific condition prior to the baseline, and each model contrasted a given CVD subtype against non-CVD to facilitate feature selection and heterogeneity mapping (i.e., to assess metabolic distinctiveness, not for clinical classification per se).

Model Construction and Validation

Each machine learning algorithm had specific hyperparameters that influenced its discriminative performance. To minimise optimisation bias, we used nested cross-validation (outer 5-fold CV for performance/stability estimation; inner 5-fold CV for hyperparameter tuning). Within the inner loop we employed a combination of grid search, random search, and manual fine-tuning: Logistic regression was optimized for regularization strength (C) and solver type (e.g., 'liblinear' for small datasets, 'saga' for larger datasets with L1/L2 regularization); Random forest was fine-tuned for number of trees (n_estimators), tree depth (max_depth), minimum samples per leaf (min_samples_leaf), and feature selection criteria (max_features) to balance bias-variance tradeoff. XGBoost was adjusted for learning rate

(learning_rate), maximum tree depth (max_depth), subsample ratio (subsample), and column sampling (colsample_bytree) to enhance model efficiency and reduce overfitting. Early stopping was applied for XGBoost using a stratified validation split within the inner loop.

Hyperparameter tuning was conducted iteratively, starting with random search to explore a wide range of parameter values, followed by grid search for fine-tuning within a narrowed search space. The optimal parameter combination was then selected based on cross-validation performance, considering area under the curve (AUC), F1-score, and accuracy.

To ensure robustness and generalizability, model performance was evaluated using 5-fold cross-validation, where the training dataset was split into five equal-sized subsets. In each iteration, four subsets were used for training, while the remaining subset served as the validation set. This process was repeated five times, ensuring that each subset functioned as the validation set once, and the final performance was computed as the average of all iterations.

Participants recruited from the 22 assessment centers (11012, 11021, 11011, 11008, 11009,11024, 11020, 11018, 11010, 11016, 11001, 11017, 11013, 11002, 11007, 11014, 10003, 11006, 11025, 11026, 11027, 11028) in England (n = 222606) were included in the training dataset, while participants recruited from the 5 assessment centers (11004, 11005,11003, 11022, 11023) in Scotland and Wales (n = 21,961) were included in an internal geographic hold-out dataset. All tuning and threshold choices were made within the England dataset; no refitting or threshold optimisation was performed on the internal geographic

hold-out cohort.

Performance Metrics

Model performance was assessed using both classification and curve-based metrics. Classification metrics included accuracy (acc), which measures overall correctness; sensitivity (Sn, recall), the proportion of actual positive cases correctly identified; specificity (sp), the proportion of actual negative cases correctly classified; positive predictive value (PPV, precision), the likelihood that a predicted positive case is truly positive; negative predictive value (NPV), the likelihood that a predicted negative case is truly negative; and F1 score, the harmonic mean of precision and recall, balancing false positives and false negatives(5,6). Curve-based metrics included the Receiver Operating Characteristic (ROC) curve, which assesses the tradeoff between sensitivity and specificity, and the AUC, which quantifies overall classification performance, with AUC = 1.0 representing a perfect classifier. To further assess the stability and reliability of model performance, bootstrap resampling (n=1,000 iterations) was applied to compute 95% confidence intervals (CI) for all metrics, providing a more robust estimate of model variance and performance uncertainty.

Visualization of Metabolite-Disease Relationships

The CVDs included in this study were categorized into seven major groups, based on the official ICD-10 code classification: Chronic rheumatic heart disease, ischemic heart disease, cerebrovascular disease, transient cerebral ischemic attacks and related syndromes, hypertensive diseases, other forms of heart disease, and diseases of arteries, arterioles, and capillaries.

To visually represent the distribution of these important features across different CVD types, we used stacked histograms to illustrate the proportion of each disease category in relation to the overall frequency of the top 30 SHAP features. The selected diseases for analysis were those that had an AUC value greater than 0.8 in the XGBOOST models, which were built using both metabolomics data and clinical features. We acknowledge that for rare disease subtypes with low Events Per Variable (EPV), a high AUC may be an indicator of model overfitting; therefore, these results are interpreted with caution as exploratory. Features recurring in a minimum of five disease categories within the top 30 SHAP rankings were selected to highlight associations across the CVD spectrum.. This approach provided an intuitive visual representation of how specific features are associated with different CVD types.

To visualize the relationships between metabolites, diseases, and clinical features, we employed a Sankey diagram. This diagram was created using Python, providing an intuitive way to illustrate the flow and connections between the different categories. The Sankey diagram effectively visualizes how metabolites are associated with specific CVD diseases and clinical characteristics, highlighting the contributions of each feature to the overall disease classification. The thickness of the connecting lines is proportional to the recurrence of the top 30 SHAP-ranked features across diseases.

The feature importance for the Sankey diagram was calculated as:

$$\text{Feature importance}(j) = \frac{1}{\mathrm{N}}\sum_{i=1}^{N} |\o i, j|$$

Where N is the number of samples, φ_i,j is the SHAP value for feature j for sample i, and $|\varphi\_i,j|$ represents the absolute value of the SHAP value for feature j for sample i, which indicates the contribution of this feature to the model's prediction. Thicker lines indicate stronger contributions, reflecting the importance of each feature in the model-based discrimination of prevalent CVD phenotypes(7).

Cross-sectional Discriminative Performance of Cross-CVD Metabolic Features

To evaluate the cross-sectional discriminative ability of our Cross-CVD Metabolite Features, we constructed cross-sectional discrimination models using only these 21 selected metabolites as input features and evaluated their performance across different cardiovascular disease classes and subclasses. The models were initially developed and tested on the England cohort, followed by validation on the internal geographic hold-out cohorts from Scotland and Wales to assess generalizability across different populations. These analyses were used to assess the parsimony and stability of cross-CVD signals over and above clinical information, not to develop deployable diagnostic tools.

Results

Study Cohorts

The sex distribution revealed a female predominance in the overall cohort. However, within the CVD group, the majority were male (n = 16,409), compared to females (n = 11,541), indicating a higher burden of CVDs in men despite the larger number of women in the cohort. Conversely, among participants without CVDs, females outnumbered males (117,108 vs. 99,509) as shown in Figure S3A.

The age of participants ranged from 37 to 73 years at recruitment. Age-stratified analysis showed that CVD prevalence increased with age (Supplement Figure 3B). The majority of CVD cases were found in the 56–65 age group (n = 14,705), followed by those older than 65 years (n = 7,490) (Supplement Figure 3B). This trend is consistent with the known age-related rise in cardiovascular risk. In contrast, the highest number of Non_CVD individuals were also in the 56–65 age group (n = 90,033), followed by the 46–55 group (n = 68,766), reflecting the age composition of the UKB cohort.

Metabolomic Profiling and Machine Learning Classification of CVDs

To further evaluate the generalizability of our models, we performed validation using the internal geographic hold-out dataset from Scotland and Wales (Table S7). Consistent with internal validation results, all three machine learning algorithms demonstrated robust performance. Using metabolomic data alone, AUC values were 0.7594, 0.7512, and 0.7557 for LR, RF, and XGBoost, respectively. Upon integrating clinical variables, the XGBoost model again yielded the best performance, achieving an AUC of 0.7910 (95% CI: 0.7806–0.8016)(full metrics detailed in Table S7). These results highlight the robustness of metabolic patterns for cross-sectional discrimination of prevalent CVD status across independent populations within the UKB.

Global metabolomic landscape and inter-disease similarity

The class-level heatmap ordered by inter-disease similarity reveals clear and reproducible structures shown in Figure 2A. Ischaemic entities—acute and subsequent myocardial infarction, chronic ischaemic heart disease, angina pectoris and atherosclerotic

disease—form a tight cluster characterised by concordant shifts across IDL/LDL-related lipid fractions and fatty-acid composition indices. Hypertensive and hypertensive-renal diseases adjoin this block, sharing the lipid-centred background with additional features consistent with cardio-renal coupling. Cerebrovascular diseases (including transient cerebral ischaemic attacks) lie proximal to the ischaemic cluster but display a less strongly lipid-dominant profile, in keeping with a shared atherosclerotic–inflammatory milieu. In contrast, rheumatic/valvular classes segregate on a separate branch with lower overlap to lipid-driven patterns, while pulmonary embolism is distinctly isolated relative to the other disease classes in the similarity space. Together, these diagnosis-independent structures provide a global scaffold for summarizing shared versus disease-specific metabolic alterations across CVD classes.

At finer granularity, the subclass map preserves and refines the class-level architecture (Figure 2B). Acute myocardial infarction subclasses—acute transmural MI of the anterior and inferior walls and other sites, together with acute MI unspecified and old MI—assemble into a compact micro-cluster, again marked by coherent changes in lipoprotein composition and fatty-acid fractions. Chronic ischaemic heart disease (unspecified) and angina pectoris (unspecified) reside adjacent to this block with similar directions of change but reduced contrast relative to acute entities. Along the hypertensive–renal axis, hypertensive renal disease with renal failure occupies the extreme of the gradient, combining marked lipid/lipoprotein alterations with creatinine-linked signals; other hypertensive subclasses align along the same arm with progressively milder patterns. Heart-failure subclasses (e.g., left

ventricular failure, congestive heart failure) lie near the ischaemic block, sharing the lipid-centred background while exhibiting additional differences in selected small-molecule and protein measures. Cerebrovascular subclasses—notably cerebral infarction and occlusion/stenosis of cerebral arteries—cluster together close to the ischaemic axis but with a less pronounced lipid signature. Rheumatic/valvular subclasses form a distinct branch with limited overlap to lipid-driven patterns, and pulmonary embolism remains relatively isolated. These subclass-level patterns expose stable ischaemic micro-clusters, a graded hypertensive–renal arm, and distinct rheumatic/valvular trajectories, refining the cardiovascular metabolic landscape established at the class level.

To assess potential confounding by statin therapy, we repeated hierarchical clustering on statin-adjusted and unadjusted residuals and quantified concordance using the Adjusted Rand Index; concordance was high at the class level (ARI = 0.9138), indicating that the global clustering structure is robust to statin adjustment (Methods).

Comprehensive Exploration of Metabolic Signatures Across CVD Classes and Subclasses Using Machine Learning

The same pattern of metabolic signature distinctiveness was observed when using Logistic Regression (Supplementary Figure S9) and Random Forest (Supplementary Figure S10) algorithms. Integration of clinical data with metabolomics enhanced the ability to detect metabolic differences, with detailed results shown in Supplementary Figures S5 and S9 for subclass analysis. SHAP analysis identified key metabolic features including Age, IDL_CE, CHOL, Sex, LA, CYS, IDL_C, Total_CE, and GGT as consistently important across multiple

CVD conditions (Supplementary Figure S10).

To assess the robustness of our findings across different machine learning approaches, we evaluated the top five prevalent CVD classes (Chronic ischaemic heart disease, Acute myocardial infarction, Angina pectoris, Heart failure, and Cerebral infarction) using XGBoost, Logistic Regression, and Random Forest algorithms (Supplementary Figure 6A). ROC curve analysis demonstrated comparable performance across all three methods on the England discovery dataset, with XGBoost showing slightly superior performance. Validation on the internal geographic hold-out cohort (Supplementary Figure 6B) yielded AUC values that closely matched those of the primary dataset, supporting the robustness and generalizability of our models across different populations and algorithmic approaches. Similarly, we evaluated the top five prevalent CVD subclasses (Acute transmural myocardial infarction of anterior wall, Atherosclerotic heart disease, Chronic ischaemic heart disease unspecified, Angina pectoris unspecified, and Old myocardial infarction) across all three machine learning algorithms (Supplementary Figure 6D). All algorithms demonstrated consistent performance on both the primary England dataset and internal geographic hold-out cohort (Supplementary Figure 6E), corroborating the generalizability and algorithmic independence of our discrimination approach. SHAP (SHapley Additive exPlanations) analysis identified key metabolic features driving CVD discrimination and provided mechanistic insights into the underlying biological processes. For CVD class analysis (Supplementary Figure 6C), the most important discriminative features included IDL_CE (cholesteryl esters in intermediate-density lipoproteins), CHOL (total cholesterol), LA (linoleic acid), CYS

(cysteine), IDL_C (total cholesterol in intermediate-density lipoproteins), Total_CE (total cholesteryl esters), and GGT (gamma-glutamyl transferase), alongside demographic factors (Age, Sex). SHAP analysis for CVD subclasses (Supplementary Figure 6F) revealed consistent patterns, with the same metabolic features maintaining high importance across different levels of classification granularity. This consistency underscores the fundamental role of lipid metabolism (particularly lipoprotein subfractions and cholesteryl esters), amino acid metabolism (cysteine), and hepatic function markers (GGT) in distinguishing various CVD conditions.

This comprehensive metabolomics-based analysis demonstrates that different CVD conditions exhibit distinct metabolic signatures with varying degrees of distinctiveness. The consistency of results across algorithms and successful internal geographic hold-out validation for common, high-EPV diseases supports the robustness of metabolomics-based cross-sectional discrimination of prevalent CVD status. Integration with clinical data further enhanced performance as detailed in the supplementary analyses (Supplementary Figures 5A-5F).

The performance outcomes of the Logistic Regression and Random Forest models are detailed in Figure S9 and S10, respectively. These models exhibited classification patterns similar to those observed with the XGBoost model (Table S8).

Mapping Metabolic Heterogeneity via Machine Learning

To comprehensively explore the metabolic alterations and signatures associated with different cardiovascular diseases, we developed machine learning models for 37 CVD classes

and 50 CVD subclasses using XGBoost as the primary method, with metabolomics data as the sole input features. This approach allowed us to systematically characterize distinct metabolic patterns and commonalities across various CVD conditions. The performance metrics for all machine learning models are detailed in Table S6, while the statistical power (EPV) for each model is detailed in Table S5 .

As shown in Figure 3A, the distinctiveness of metabolic signatures varied considerably across CVD conditions. A critical finding emerged when contrasting model performance against statistical power (Table S5). For rare conditions with extremely low statistical power, such as Hypertensive renal disease (n=322, EPV $\approx$ 0.99), the model produced the highest AUC (0.9194). This result is a classic signature of model overfitting and is considered a statistical artifact. By comparison, highly prevalent conditions with strong statistical power also showed high metabolic distinctiveness. For example, Chronic ischaemic heart disease (n=7,244, EPV $\approx$ 22.3) and Angina pectoris (n= 5,562, EPV $\approx$ 17.1) yielded robustly high AUCs (0.8647 and 0.8452, respectively).In contrast, conditions such as Rheumatic mitral valve diseases (n=90, EPV $\approx$ 0.3) showed more subtle metabolic alterations.The distribution of metabolic signature distinctiveness across different performance ranges is presented in Figure 3B. Among all CVD classes analyzed, the 3% that showed highly distinct metabolic profiles (AUC >0.9) corresponds to the aforementioned overfit model. A more reliable 33% demonstrated moderately distinct signatures (AUC 0.8-0.9) representing common diseases with sufficient statistical power. 35% showed subtle but detectable metabolic differences (AUC 0.7-0.8), 24% exhibited minimal metabolic alterations (AUC

0.6-0.7), and 5% showed metabolic profiles similar to healthy controls (AUC <0.6).

To validate the consistency of metabolic signature patterns across different populations, we utilized the internal geographic hold-out dataset from Scotland and Wales regions, focusing on 15 CVD classes with sufficient case numbers (Figure 3C). The hold-out validation demonstrated consistent metabolic patterns across geographical regions. Key conditions such as Chronic ischaemic heart disease (AUC=0.8842), Acute myocardial infarction (AUC=0.8759), Angina pectoris (AUC=0.8452), Heart failure (AUC=0.8343), and Cerebral infarction (AUC=0.7748) maintained similar degrees of metabolic alterations in the hold-out validation set, supporting the reproducibility of metabolic signatures across different populations.

We further refined our analysis by examining metabolic signatures in distinct CVD subclasses based on detailed ICD-10 classifications. Our analysis of CVD subclasses identified several highly distinct metabolic signatures (Figure 3D), even among some rarer conditions with limited statistical power (low EPV, Table S5). The distribution of metabolic signature distinctiveness for CVD subclasses (Figure 3E) reveals that 50% of conditions exhibited pronounced metabolic alterations (AUC >0.8). Notably, the patterns for subtypes such as Hypertensive renal disease with renal failure (AUC=0.9217) and acute transmural myocardial infarction (AUCs 0.8833–0.8912) were consistently replicated in the internal geographical hold-out dataset (Figure 3F). These robust yet preliminary findings highlight specific subtypes worthy of deeper investigation and will be followed by further analyses to confirm their biological significance.

The same pattern of metabolic signature distinctiveness was observed when using Logistic Regression (Supplementary Figure S9) and Random Forest (Supplementary Figure S10) algorithms. Integration of clinical data with metabolomics enhanced the ability to detect metabolic differences, with detailed results shown in Supplementary Figures S5 and S9 for subclass analysis. SHAP analysis identified key metabolic features including Age, IDL_CE, CHOL, Sex, LA, CYS, IDL_C, Total_CE, and GGT as consistently important across multiple CVD conditions (Supplementary Figure S10).

To assess the robustness of our findings across different machine learning approaches, we evaluated the top five prevalent CVD classes (Chronic ischaemic heart disease, Acute myocardial infarction, Angina pectoris, Heart failure, and Cerebral infarction) using XGBoost, Logistic Regression, and Random Forest algorithms (Supplementary Figure 6A). ROC curve analysis demonstrated comparable performance across all three methods on the England discovery dataset, with XGBoost showing slightly superior performance. Validation on the internal geographic hold-out cohort (Supplementary Figure 6B) yielded AUC values that closely matched those of the primary dataset, supporting the robustness and generalizability of our models across different populations and algorithmic approaches. Similarly, we evaluated the top five prevalent CVD subclasses (Acute transmural myocardial infarction of anterior wall, Atherosclerotic heart disease, Chronic ischaemic heart disease unspecified, Angina pectoris unspecified, and Old myocardial infarction) across all three machine learning algorithms (Supplementary Figure 6D). All algorithms demonstrated consistent performance on both the primary England dataset and internal geographic hold-out cohort (Supplementary

Figure 6E), corroborating the generalizability and algorithmic independence of our discrimination approach. SHAP (SHapley Additive exPlanations) analysis identified key metabolic features driving CVD discrimination and provided mechanistic insights into the underlying biological processes. For CVD class analysis (Supplementary Figure 6C), the most important discriminative features included IDL_CE (cholesteryl esters in intermediate-density lipoproteins), CHOL (total cholesterol), LA (linoleic acid), CYS (cysteine), IDL_C (total cholesterol in intermediate-density lipoproteins), Total_CE (total cholesteryl esters), and GGT (gamma-glutamyl transferase), alongside demographic factors (Age, Sex). SHAP analysis for CVD subclasses (Supplementary Figure 6F) revealed consistent patterns, with the same metabolic features maintaining high importance across different levels of classification granularity. This consistency underscores the fundamental role of lipid metabolism (particularly lipoprotein subfractions and cholesteryl esters), amino acid metabolism (cysteine), and hepatic function markers (GGT) in distinguishing various CVD conditions.

This comprehensive metabolomics-based analysis demonstrates that different CVD conditions exhibit distinct metabolic signatures with varying degrees of distinctiveness. The consistency of results across algorithms and successful internal geographic hold-out validation for common, high-EPV diseases supports the robustness of metabolomics-based cross-sectional discrimination of prevalent CVD status. Integration with clinical data further enhanced performance as detailed in the supplementary analyses (Supplementary Figures 5A-5F).

The performance outcomes of the Logistic Regression and Random Forest models are detailed in Figure S9 and S10, respectively. These models exhibited classification patterns similar to those observed with the XGBoost model (Table S8).

References

1. Collins R. What makes UK Biobank special? Lancet (London, England) 2012;379:1173-4.
2. Bycroft C, Freeman C, Petkova D et al. The UK Biobank resource with deep phenotyping and genomic data. Nature 2018;562:203-209.
3. Saeed MH, Hama JI. Cardiac disease prediction using AI algorithms with SelectKBest. Medical & biological engineering & computing 2023;61:3397-3408.
4. Ritchie SC, Surendran P, Karthikeyan S et al. Quality control and removal of technical variation of NMR metabolic biomarker data in ~120,000 UK Biobank participants. Sci Data 2023;10:64.
5. Liu M, Zhou J, Xi Q et al. A computational framework of routine test data for the cost-effective chronic disease prediction. Briefings in bioinformatics 2023;24.
6. Ben-Haim Y, Dacso CC. Interpreting PPV and NPV of Diagnostic Tests with Uncertain Prevalence. Rambam Maimonides medical journal 2024;15.
7. Lee SMLaS-I. A Unified Approach to Interpreting Model Predictions. Advances in neural information processing systems: Curran Associates, Inc, 2017:4765--4774.

Supplementary figures

Supplementary Figure 1. Schematic of the study workflow. The schematic illustrates the workflow of our UKB-based study. After applying exclusion criteria (participants without NMR data and those with cancer), the final cohort comprised 244,567 participants with electronic health record (EHR) linkage (ICD-10 and GP). We classified cardiovascular diseases into 37 CVD classes and 50 subclasses based on ICD-10 codes. The data includes metabolomic data (325 metabolites measured via Nightingale Health NMR platform) and clinical data (blood routine parameters, biochemistry measurements, and demographic data including age, sex, and BMI).

Metabolite differences across CVD classes were analyzed, and three machine learning algorithms (Logistic Regression, Random Forest, and XGBoost), with SHAP values used for model interpretation. The metabolite-linked CVD subtypes and cross-CVD metabolite features were identified. These cross-CVD metabolite features were subsequently validated using participants from different UK regions (England cohort for training, n=222,606; Scotland and Wales cohorts for internal geographic hold-out validation, n=21,961) to confirm their robustness and generalizability.

Supplementary Figure 2. Study design and modeling workflow. From 502,131 UK Biobank participants enrolled in 2006–2010, we excluded 227,895 without metabolomics and 29,669 with a cancer history, yielding 244,567 eligible individuals. The England subset (n=222,606) was used for model development with 5-fold cross-validation across three algorithms—logistic regression (LR), random forest (RF), and XGBoost—to build CVD metabolic association models. An independent cohort from Scotland and Wales (n=21,961) served for internal geographic hold-out validation. Performance evaluation included receiver-operating-characteristic (ROC) analysis and summary metrics (ACC, SN, SP, PPV, NPV, F1). Model interpretability relied on SHAP (Shapley Additive exPlanations), and the best-performing models were selected based on these assessments.

Supplementary Figure 3. Distribution of CVD status by sex and age group. (A) Bar plot showing the number of individuals stratified by sex and CVD status. The cohort includes 16,409 male CVD participants, 11,541 female CVD participants, 99,509 male Non-CVD participants controls, and 117,108 female Non-CVD participants controls. (B) Bar plot

displaying the count of individuals with and without CVDs across different age groups. The distribution is shown for age groups 35-45 (n=1,026 CVDs; n=31,884 Non-CVD participants), 46-55 (n=4,729 CVDs; n=68,766 Non-CVD participants), 56-65 (n=14,705 CVDs; n=90,033 Non-CVD participants), and >65 (n=7,490 CVDs; n=25,934 Non-CVD participants).

Supplementary Figure 4. Distribution of core metabolic features across cardiovascular disease subclasses. Box plots illustrating the distribution of the five most frequently recurrent metabolites—IDL_CE (cholesteryl esters in intermediate-density lipoproteins), IDL_C (total cholesterol in intermediate-density lipoproteins), LA_pct (linoleic acid percentage), LDL_C_pct (cholesteryl esters in low-density lipoproteins percentage), and LA (linoleic acid)—across individual CVD subclasses compared with non-CVD controls. These features represent the core "Cross-CVD" signature identified through SHAP-based frequency ranking. Statistical comparisons between each CVD subclass and the non-CVD group were performed using the two-sided Mann–Whitney U test. P-values were adjusted for multiple testing across all metabolite-disease comparisons using the Benjamini–Hochberg false discovery rate (FDR) procedure. The resulting significance levels (q-values) are indicated by asterisks: * $q < 0.05$, ** $q < 0.01$, and *** $q < 0.001$. These subclass-level distributions refine the metabolic landscape by confirming that shared perturbations in lipoprotein and fatty acid metabolism are consistently preserved at finer-grained levels of disease classification.

Supplementary Figure 5. Combined (metabolomics + clinical) models: classes and subclasses. A) Classes—AUC ranking for combined models (metabolomics + clinical). B)

Classes-internal geographic hold-out validation (combined). (C) Classes - AUC distribution across ranges (combined). (D) Subclasses-AUC ranking for combined models. (E) Subclasses - (combined). (F) Subclasses - AUC distribution across ranges (combined). Table S1 details the participant counts (n) for each CVD class and subclass.

Supplementary Figure 6. ROC and SHAP details for top entities and alternative algorithms. A–C correspond to the five top CVD classes: I25 (chronic ischaemic heart disease), I21 (acute myocardial infarction), I20 (angina pectoris), I50 (heart failure), and I63 (cerebral infarction). (A) ROC curves in the England development cohort (n=222,606) for each class, comparing logistic regression (LR), random forest (RF), and XGBoost; legends report AUCs for each algorithm. (B) ROC curves for the same five classes in the external Scotland/Wales cohort (n=21,961). (C) SHAP beeswarm summaries for the combined (metabolomics + clinical) models of these classes; the x-axis shows SHAP values (feature impact on log-odds), and point colour encodes feature values (pink = higher, blue = lower). D-F correspond to the five top CVD subclasses: I21.0 (acute transmural myocardial infarction of anterior wall), I25.1 (atherosclerotic heart disease), I25.9 (chronic ischaemic heart disease, unspecified), I20.9 (angina pectoris, unspecified), and I25.2 (old myocardial infarction). (D) ROC curves in the England development cohort across LR/RF/XGBoost. (E) ROC curves for the same subclasses in the external Scotland/Wales cohort. (F) SHAP beeswarm summaries for the combined models of these subclasses (axes/colouring as in panel C). The grey diagonal in ROC plots denotes random discrimination. All models were trained in England with 5-fold cross-validation and evaluated on the independent external

cohort; full metrics are provided in Tables S7–S8.

Supplementary Figure 7. Logistic regression-based discrimination of specific cardiovascular disease subclasses. A) AUC values for discrimination of individual CVD subclasses versus non-CVD using metabolomics data alone, ranked in descending order. B) AUC values for discrimination using combined metabolomics and clinical data. C-D) Distribution of model performance across different cardiovascular condition subclasses for metabolomics-only models (C) and combined metabolomics-clinical models (D). E-F) Proportion of CVD subclasses achieving different AUC value ranges for metabolomics models (E) and combined metabolomics-clinical models (F). Table S1 details the participant counts (n) for each CVD class and subclass.

Supplementary Figure 8. Random forest-based discrimination of cardiovascular disease classes. A) AUC values for discrimination of individual CVD classes versus non-CVD using metabolomics data alone, ranked in descending order. B) AUC values for discrimination using combined metabolomics and clinical data. C-D) Distribution of model performance across different cardiovascular conditions for metabolomics-only models (C) and combined metabolomics-clinical models (D). E-F) Proportion of CVD classes achieving different AUC value ranges for metabolomics models (E) and combined metabolomics-clinical models (F). Table S1 details the participant counts (n) for each CVD class and subclass.

Supplementary Figure 9. Logistic regression discrimination of specific cardiovascular disease subclasses. A) AUC values for discrimination of individual CVD subclasses versus non-CVD using metabolomics data alone, ranked in descending order. B) AUC values for

discrimination using combined metabolomics and clinical data. C-D) Distribution of model performance across different cardiovascular condition subclasses for metabolomics-only models (C) and combined metabolomics-clinical models (D). E-F) Proportion of CVD subclasses achieving different AUC value ranges for metabolomics models (E) and combined metabolomics-clinical models (F). Table S1 details the participant counts (n) for each CVD class and subclass.

Supplementary Figure 10. Random forest-based discrimination of specific cardiovascular disease subclasses. A) AUC values for discrimination of individual CVD subclasses versus non-CVD using metabolomics data alone, ranked in descending order. B) AUC values for discrimination using combined metabolomics and clinical data. C-D) Distribution of model performance across different cardiovascular condition subclasses for metabolomics-only models (C) and combined metabolomics-clinical models (D). E-F) Proportion of CVD subclasses achieving different AUC value ranges for metabolomics models (E) and combined metabolomics-clinical models (F). Table S1 details the participant counts (n) for each CVD class and subclass.

Supplementary Figure 11. Performance of parsimonious models using Cross-CVD Metabolite Features. A) Classes - ranking. AUC values for cross-sectional discrimination of the top 15 CVD classes in the England cohort (n=222,606) using only the identified Cross-CVD Metabolite Features, ranked from highest to lowest performance. B) Subclasses - ranking. AUC values for the top 15 CVD subclasses in the England cohort using the same parsimonious 21-metabolite set. C) Classes - geographic validation. Comprehensive AUC

values for all CVD classes in the internal geographic hold-out validation cohort (Scotland and Wales, n=21,961), demonstrating the robust generalizability of metabolite-based discrimination across different populations. D) Subclasses - geographic validation. AUC values for all CVD subclasses in the internal geographic hold-out validation cohort. These analyses confirm that a compact set of biologically coherent features preserves high discriminative performance across the cardiovascular spectrum, even when applied to independent geographic regions.

Supplementary tables

Table S1. Distribution of CVD cases stratified by class and subclass in the study cohort

Table S2. List of metabolites and their abbreviations

Table S3. Clinical data and abbreviations

Table S4. Prevalence of cardio-metabolic medication use in CVD and non-CVD cohorts

Table S5. Statistical power summary (Events Per Variable, EPV) for all CVD models

Table S6. Discrimination performance of machine learning models based on metabolomic and clinical data integration

Table S7. internal geographic hold-out validation of XGBoost CVD class-level Discrimination

Table S8. internal geographic hold-out validation of XGBoost model for CVD subclass-level

Table S9. Discrimination performance of XGBoost using selected metabolites for CVD class-level

Table S10. Discrimination performance of XGBoost using selected metabolites for CVD subclass-level

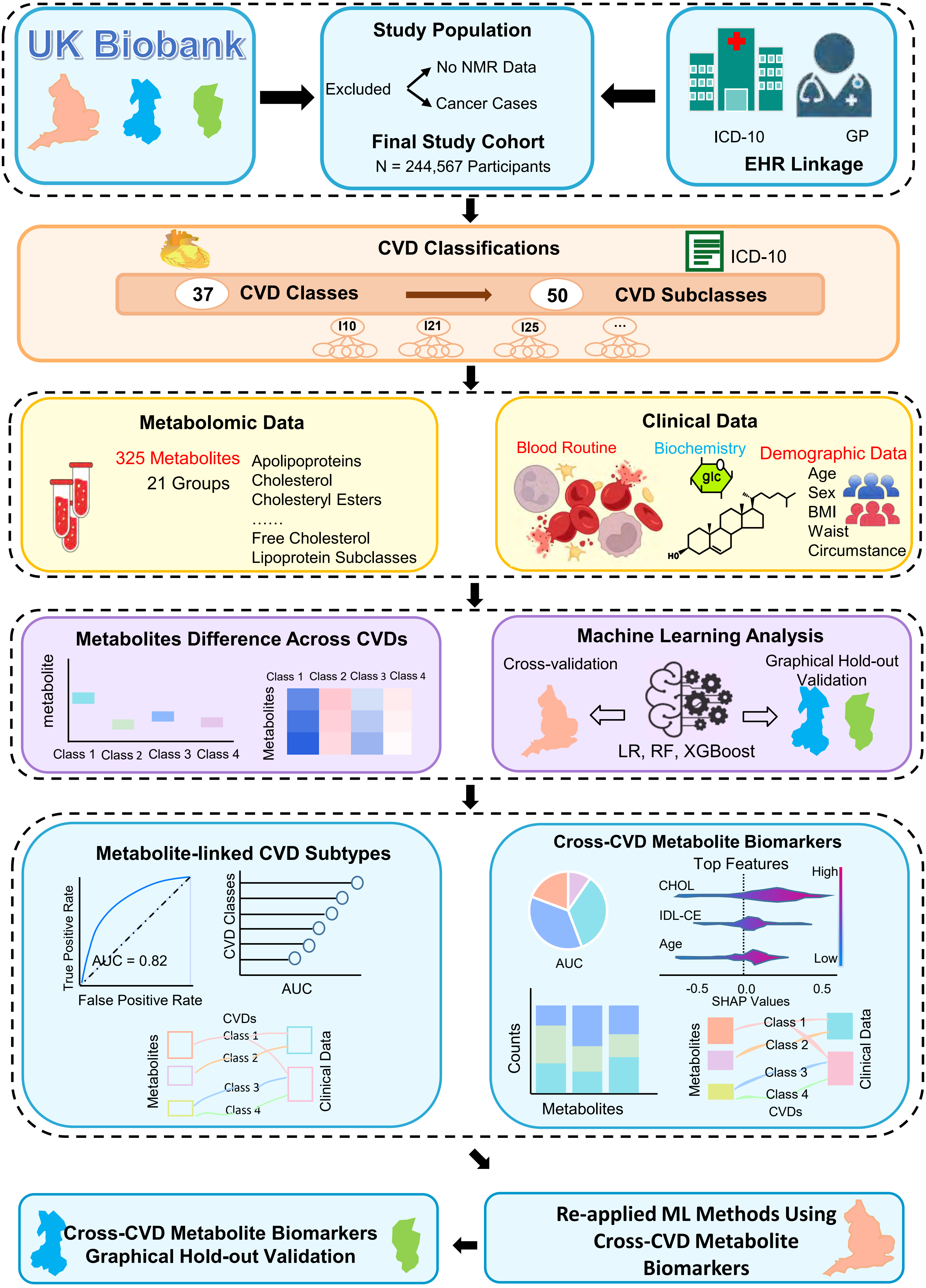
UK Biobank
Study Population
Excluded
No NMR Data
Cancer Cases
Final Study Cohort
N = 244,567 Participants
ICD-10
GP
EHR Linkage
CVD Classifications
ICD-10
37 CVD Classes
50 CVD Subclasses
I10
I21
I25
...
Metabolomic Data
325 Metabolites
21 Groups
Apolipoproteins
Cholesterol
Cholesteryl Esters
......
Free Cholesterol
Lipoprotein Subclasses
Clinical Data
Blood Routine
Biochemistry
Demographic Data
glc
Age
Sex
BMI
Waist
Circumstance
Metabolites Difference Across CVDs
metabolite
Class 1 Class 2 Class 3 Class 4
Metabolites
Class 1 Class 2 Class 3 Class 4
Machine Learning Analysis
Cross-validation
Graphical Hold-out Validation
LR, RF, XGBoost
Metabolite-linked CVD Subtypes
True Positive Rate
AUC = 0.82
False Positive Rate
CVD Classes
AUC
CVDs
Class 1
Class 2
Class 3
Class 4
Metabolites
Clinical Data
Cross-CVD Metabolite Biomarkers
Top Features
High
CHOL
IDL-CE
Age
Low
AUC
-0.5 0.0 0.5
SHAP Values
Counts
Metabolites
Class 1
Class 2
Class 3
Class 4
CVDs
Cross-CVD Metabolite Biomarkers Graphical Hold-out Validation
Re-applied ML Methods Using Cross-CVD Metabolite Biomarkers

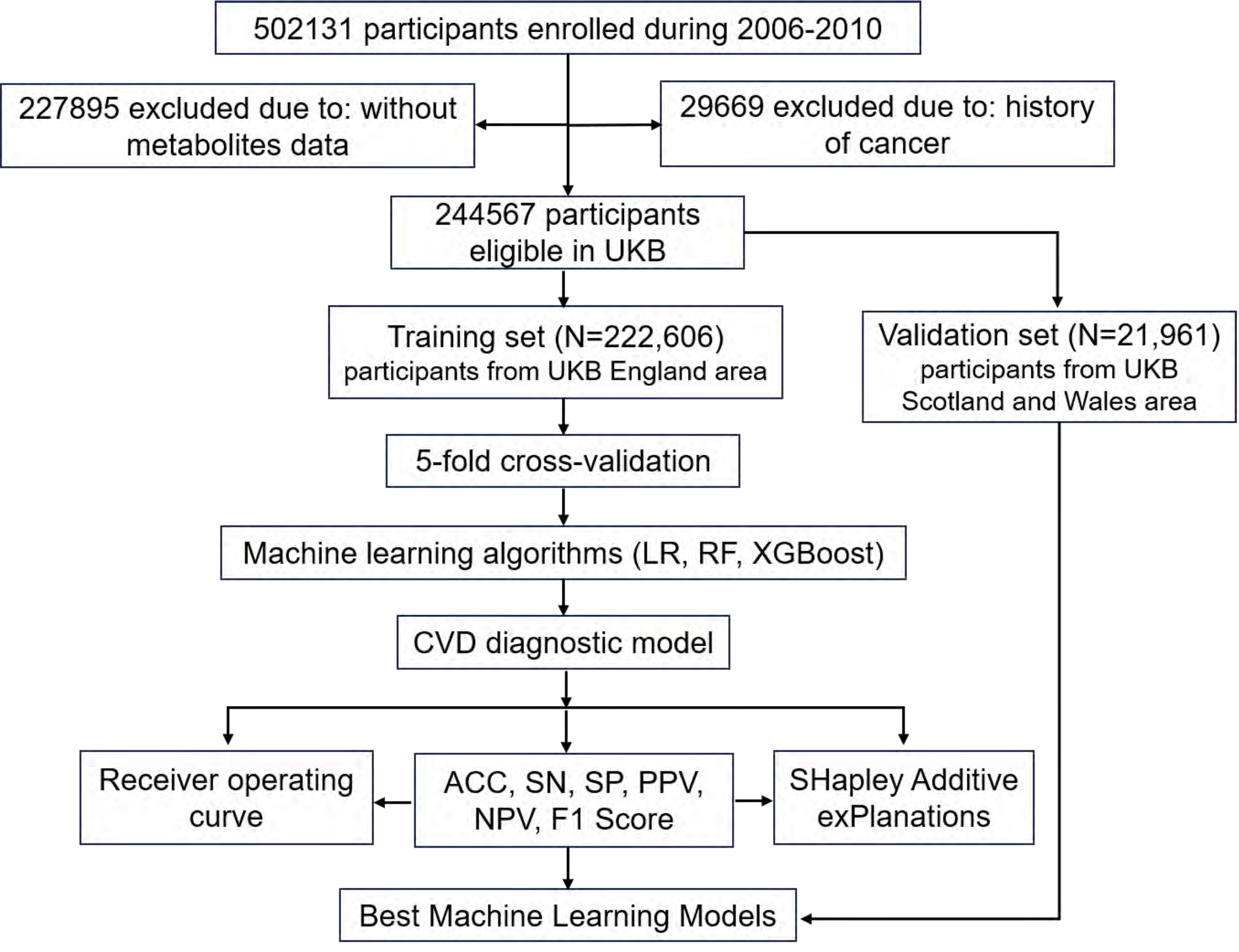

502131 participants enrolled during 2006-2010
227895 excluded due to: without metabolites data
29669 excluded due to: history of cancer
244567 participants eligible in UKB
Training set (N=222,606) participants from UKB England area
Validation set (N=21,961) participants from UKB Scotland and Wales area
5-fold cross-validation
Machine learning algorithms (LR, RF, XGBoost)
CVD diagnostic model
Receiver operating curve
ACC, SN, SP, PPV, NPV, F1 Score
SHapley Additive exPlanations
Best Machine Learning Models

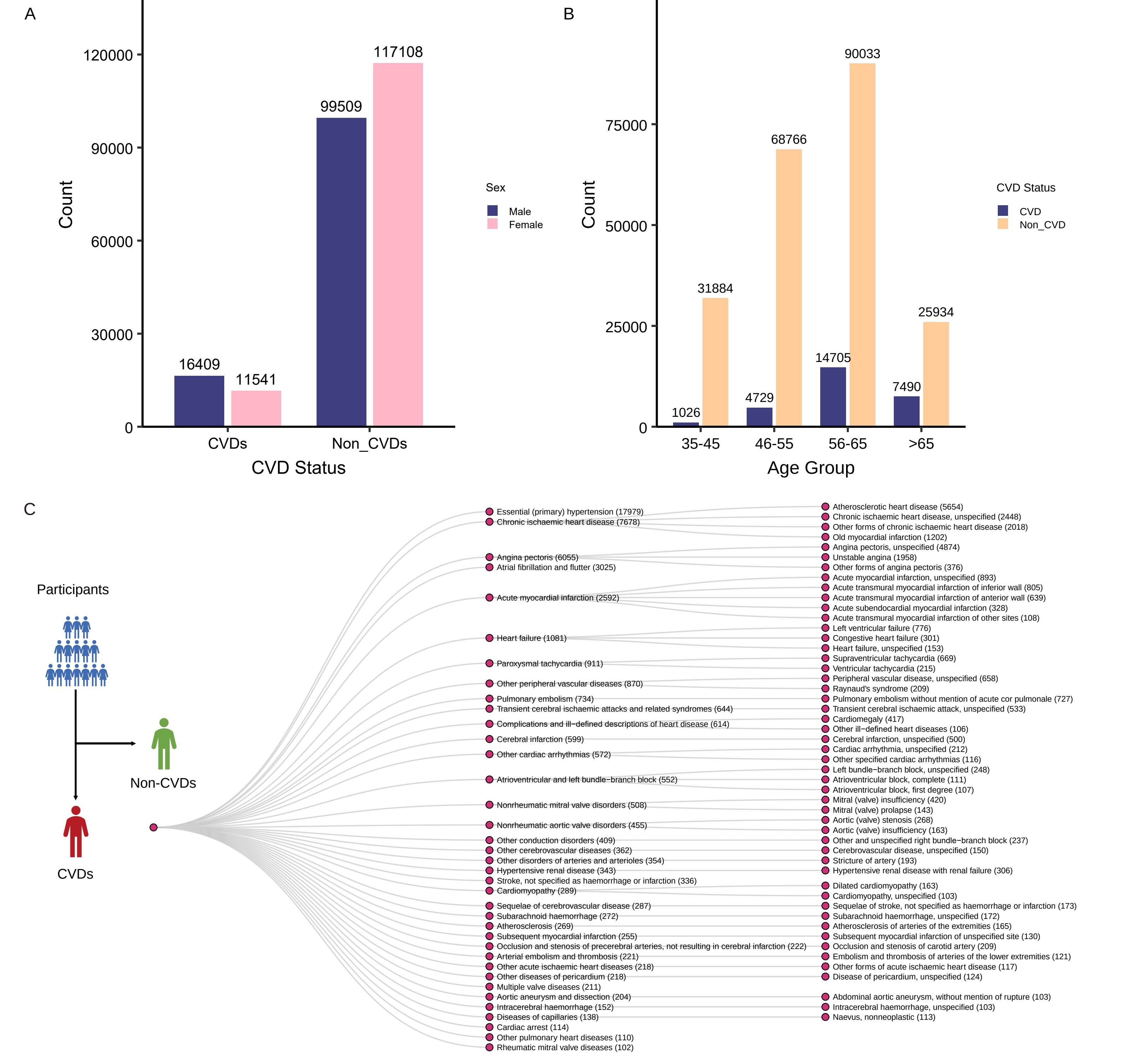
A
Count
120000
90000
60000
30000
0
16409
11541
99509
117108
CVDs
Non_CVDs
CVD Status
Sex
Male
Female
B
Count
75000
50000
25000
0
1026
31884
4729
68766
14705
90033
7490
25934
35-45
46-55
56-65
>65
Age Group
CVD Status
CVD
Non_CVD
C
Participants
Non-CVDs
CVDs
Essential (primary) hypertension (17979)
Chronic ischaemic heart disease (7678)
Angina pectoris (6055)
Atrial fibrillation and flutter (3025)
Acute myocardial infarction (2592)
Heart failure (1081)
Paroxysmal tachycardia (911)
Other peripheral vascular diseases (870)
Pulmonary embolism (734)
Transient cerebral ischaemic attacks and related syndromes (644)
Complications and ill−defined descriptions of heart disease (614)
Cerebral infarction (599)
Other cardiac arrhythmias (572)
Atrioventricular and left bundle−branch block (552)
Nonrheumatic mitral valve disorders (508)
Nonrheumatic aortic valve disorders (455)
Other conduction disorders (409)
Other cerebrovascular diseases (362)
Other disorders of arteries and arterioles (354)
Hypertensive renal disease (343)
Stroke, not specified as haemorrhage or infarction (336)
Cardiomyopathy (289)
Sequelae of cerebrovascular disease (287)
Subarachnoid haemorrhage (272)
Atherosclerosis (269)
Subsequent myocardial infarction (255)
Occlusion and stenosis of precerebral arteries, not resulting in cerebral infarction (222)
Arterial embolism and thrombosis (221)
Other acute ischaemic heart diseases (218)
Other diseases of pericardium (218)
Multiple valve diseases (211)
Aortic aneurysm and dissection (204)
Intracerebral haemorrhage (152)
Diseases of capillaries (138)
Cardiac arrest (114)
Other pulmonary heart diseases (110)
Rheumatic mitral valve diseases (102)
Atherosclerotic heart disease (5654)
Chronic ischaemic heart disease, unspecified (2448)
Other forms of chronic ischaemic heart disease (2018)
Old myocardial infarction (1202)
Angina pectoris, unspecified (4874)
Unstable angina (1958)
Other forms of angina pectoris (376)
Acute myocardial infarction, unspecified (893)
Acute transmural myocardial infarction of inferior wall (805)
Acute transmural myocardial infarction of anterior wall (639)
Acute subendocardial myocardial infarction (328)
Acute transmural myocardial infarction of other sites (108)
Left ventricular failure (776)
Congestive heart failure (301)
Heart failure, unspecified (153)
Supraventricular tachycardia (669)
Ventricular tachycardia (215)
Peripheral vascular disease, unspecified (658)
Raynaud's syndrome (209)
Pulmonary embolism without mention of acute cor pulmonale (727)
Transient cerebral ischaemic attack, unspecified (533)
Cardiomegaly (417)
Other ill−defined heart diseases (106)
Cerebral infarction, unspecified (500)
Cardiac arrhythmia, unspecified (212)
Other specified cardiac arrhythmias (116)
Left bundle−branch block, unspecified (248)
Atrioventricular block, complete (111)
Atrioventricular block, first degree (107)
Mitral (valve) insufficiency (420)
Mitral (valve) prolapse (143)
Aortic (valve) stenosis (268)
Aortic (valve) insufficiency (163)
Other and unspecified right bundle−branch block (237)
Cerebrovascular disease, unspecified (150)
Stricture of artery (193)
Hypertensive renal disease with renal failure (306)
Dilated cardiomyopathy (163)
Cardiomyopathy, unspecified (103)
Sequelae of stroke, not specified as haemorrhage or infarction (173)
Subarachnoid haemorrhage, unspecified (172)
Atherosclerosis of arteries of the extremities (165)
Subsequent myocardial infarction of unspecified site (130)
Occlusion and stenosis of carotid artery (209)
Embolism and thrombosis of arteries of the lower extremities (121)
Other forms of acute ischaemic heart disease (117)
Disease of pericardium, unspecified (124)
Abdominal aortic aneurysm, without mention of rupture (103)
Intracerebral haemorrhage, unspecified (103)
Naevus, nonneoplastic (113)

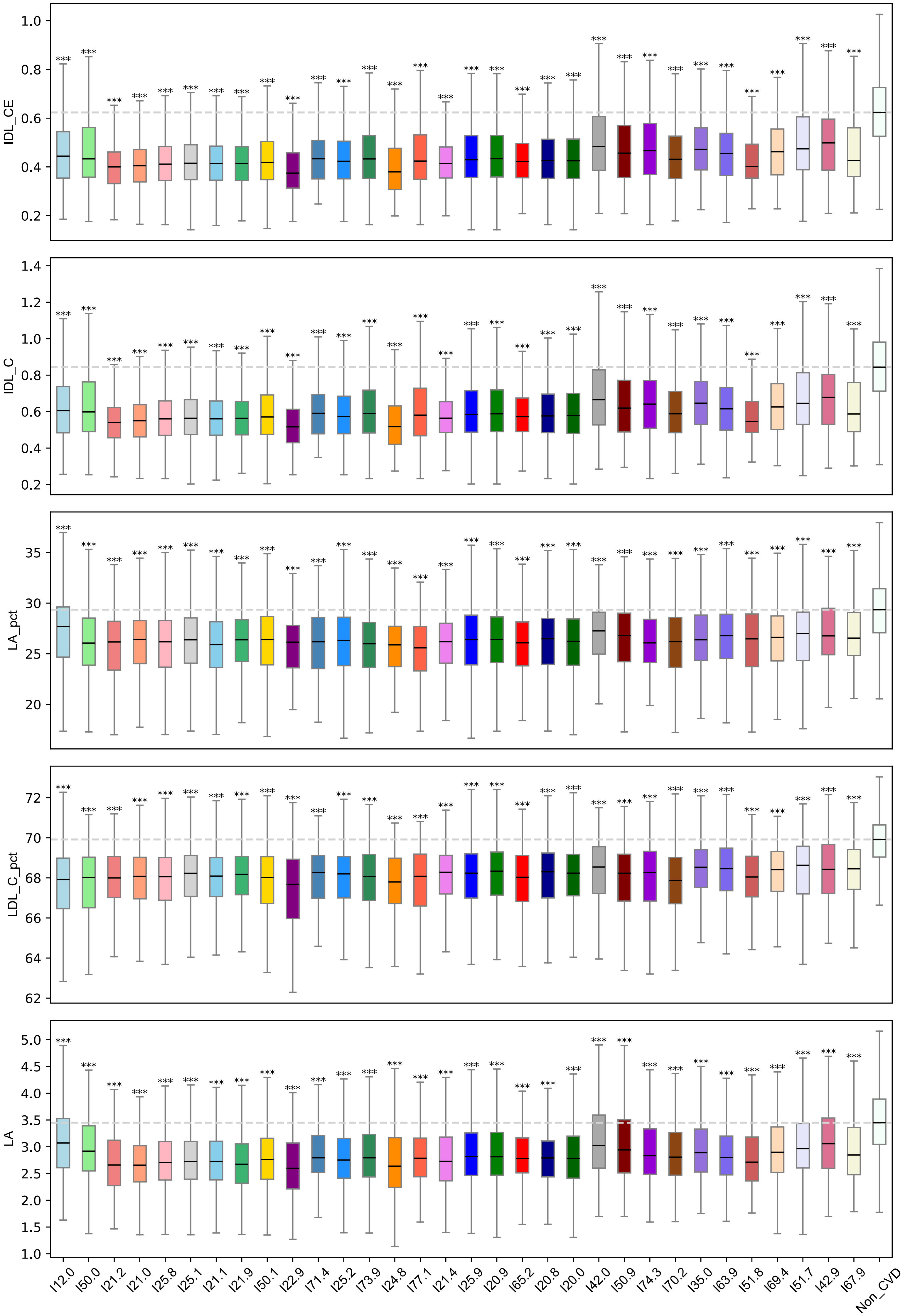
IDL_CE
IDL_C
LA_pct
LDL_C_pct
LA
I12.0
I50.0
I21.2
I21.0
I25.8
I25.1
I21.1
I21.9
I50.1
I22.9
I71.4
I25.2
I73.9
I24.8
I77.1
I21.4
I25.9
I20.9
I65.2
I20.8
I20.0
I42.0
I50.9
I74.3
I70.2
I35.0
I63.9
I51.8
I69.4
I51.7
I42.9
I67.9
Non_CVD

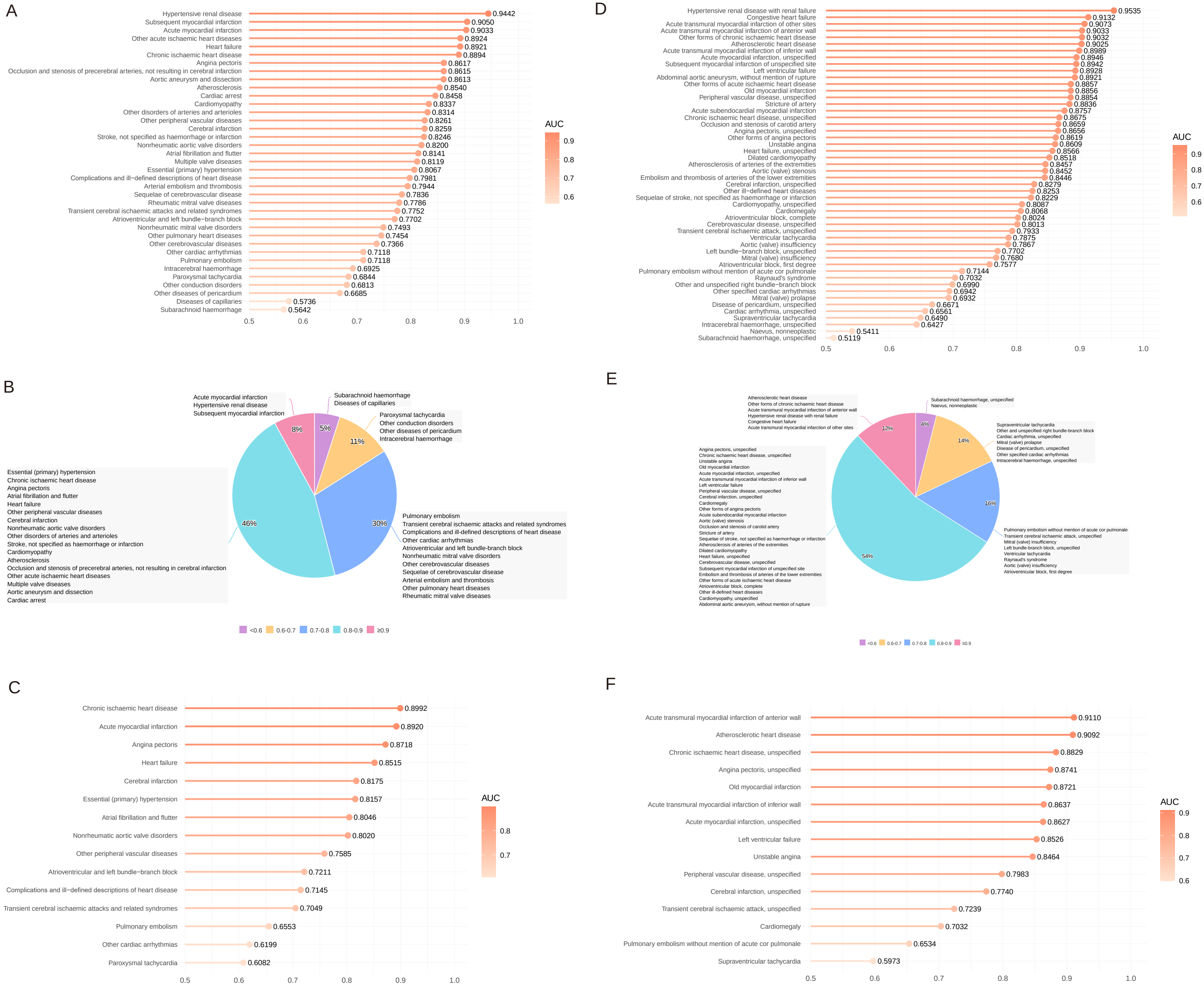
A
Hypertensive renal disease 0.9442
Subsequent myocardial infarction 0.9050
Acute myocardial infarction 0.9033
Other acute ischaemic heart diseases 0.8924
Heart failure 0.8921
Chronic ischaemic heart disease 0.8894
Angina pectoris 0.8617
Occlusion and stenosis of precerebral arteries, not resulting in cerebral infarction 0.8615
Aortic aneurysm and dissection 0.8613
Atherosclerosis 0.8540
Cardiac arrest 0.8458
Cardiomyopathy 0.8337
Other disorders of arteries and arterioles 0.8314
Other peripheral vascular diseases 0.8261
Cerebral infarction 0.8259
Stroke, not specified as haemorrhage or infarction 0.8246
Nonrheumatic aortic valve disorders 0.8200
Atrial fibrillation and flutter 0.8141
Multiple valve diseases 0.8119
Essential (primary) hypertension 0.8067
Complications and ill−defined descriptions of heart disease 0.7981
Arterial embolism and thrombosis 0.7944
Sequelae of cerebrovascular disease 0.7836
Rheumatic mitral valve diseases 0.7786
Transient cerebral ischaemic attacks and related syndromes 0.7752
Atrioventricular and left bundle−branch block 0.7702
Nonrheumatic mitral valve disorders 0.7493
Other pulmonary heart diseases 0.7454
Other cerebrovascular diseases 0.7366
Other cardiac arrhythmias 0.7118
Pulmonary embolism 0.7118
Intracerebral haemorrhage 0.6925
Paroxysmal tachycardia 0.6844
Other conduction disorders 0.6813
Other diseases of pericardium 0.6685
Diseases of capillaries 0.5736
Subarachnoid haemorrhage 0.5642
0.5 0.6 0.7 0.8 0.9 1.0
AUC 0.9 0.8 0.7 0.6
D
Hypertensive renal disease with renal failure 0.9535
Congestive heart failure 0.9132
Acute transmural myocardial infarction of other sites 0.9073
Acute transmural myocardial infarction of anterior wall 0.9033
Other forms of chronic ischaemic heart disease 0.9032
Atherosclerotic heart disease 0.9025
Acute transmural myocardial infarction of inferior wall 0.8989
Acute myocardial infarction, unspecified 0.8946
Subsequent myocardial infarction of unspecified site 0.8942
Left ventricular failure 0.8928
Abdominal aortic aneurysm, without mention of rupture 0.8921
Other forms of acute ischaemic heart disease 0.8857
Old myocardial infarction 0.8856
Peripheral vascular disease, unspecified 0.8854
Stricture of artery 0.8836
Acute subendocardial myocardial infarction 0.8757
Chronic ischaemic heart disease, unspecified 0.8675
Occlusion and stenosis of carotid artery 0.8659
Angina pectoris, unspecified 0.8656
Other forms of angina pectoris 0.8619
Unstable angina 0.8609
Heart failure, unspecified 0.8566
Dilated cardiomyopathy 0.8518
Atherosclerosis of arteries of the extremities 0.8457
Aortic (valve) stenosis 0.8452
Embolism and thrombosis of arteries of the lower extremities 0.8446
Cerebral infarction, unspecified 0.8279
Other ill−defined heart diseases 0.8253
Sequelae of stroke, not specified as haemorrhage or infarction 0.8229
Cardiomyopathy, unspecified 0.8087
Cardiomegaly 0.8068
Atrioventricular block, complete 0.8024
Cerebrovascular disease, unspecified 0.8013
Transient cerebral ischaemic attack, unspecified 0.7933
Ventricular tachycardia 0.7875
Aortic (valve) insufficiency 0.7867
Left bundle−branch block, unspecified 0.7702
Mitral (valve) insufficiency 0.7680
Atrioventricular block, first degree 0.7577
Pulmonary embolism without mention of acute cor pulmonale 0.7144
Raynaud's syndrome 0.7032
Other and unspecified right bundle−branch block 0.6990
Other specified cardiac arrhythmias 0.6942
Mitral (valve) prolapse 0.6932
Disease of pericardium, unspecified 0.6671
Cardiac arrhythmia, unspecified 0.6561
Supraventricular tachycardia 0.6490
Intracerebral haemorrhage, unspecified 0.6427
Naevus, nonneoplastic 0.5411
Subarachnoid haemorrhage, unspecified 0.5119
0.5 0.6 0.7 0.8 0.9 1.0
AUC 0.9 0.8 0.7 0.6
B
Acute myocardial infarction
Hypertensive renal disease
Subsequent myocardial infarction
Subarachnoid haemorrhage
Diseases of capillaries
Paroxysmal tachycardia
Other conduction disorders
Other diseases of pericardium
Intracerebral haemorrhage
8%
5%
11%
46%
30%
Essential (primary) hypertension
Chronic ischaemic heart disease
Angina pectoris
Atrial fibrillation and flutter
Heart failure
Other peripheral vascular diseases
Cerebral infarction
Nonrheumatic aortic valve disorders
Other disorders of arteries and arterioles
Stroke, not specified as haemorrhage or infarction
Cardiomyopathy
Atherosclerosis
Occlusion and stenosis of precerebral arteries, not resulting in cerebral infarction
Other acute ischaemic heart diseases
Multiple valve diseases
Aortic aneurysm and dissection
Cardiac arrest
Pulmonary embolism
Transient cerebral ischaemic attacks and related syndromes
Complications and ill−defined descriptions of heart disease
Other cardiac arrhythmias
Atrioventricular and left bundle−branch block
Nonrheumatic mitral valve disorders
Other cerebrovascular diseases
Sequelae of cerebrovascular disease
Arterial embolism and thrombosis
Other pulmonary heart diseases
Rheumatic mitral valve diseases
<0.6 0.6-0.7 0.7-0.8 0.8-0.9 ≥0.9
E
Atherosclerotic heart disease
Other forms of chronic ischaemic heart disease
Acute transmural myocardial infarction of anterior wall
Hypertensive renal disease with renal failure
Congestive heart failure
Acute transmural myocardial infarction of other sites
Subarachnoid haemorrhage, unspecified
Naevus, nonneoplastic
12%
4%
14%
16%
54%
Supraventricular tachycardia
Other and unspecified right bundle-branch block
Cardiac arrhythmia, unspecified
Mitral (valve) prolapse
Disease of pericardium, unspecified
Other specified cardiac arrhythmias
Intracerebral haemorrhage, unspecified
Angina pectoris, unspecified
Chronic ischaemic heart disease, unspecified
Unstable angina
Old myocardial infarction
Acute myocardial infarction, unspecified
Acute transmural myocardial infarction of inferior wall
Left ventricular failure
Peripheral vascular disease, unspecified
Cerebral infarction, unspecified
Cardiomegaly
Other forms of angina pectoris
Acute subendocardial myocardial infarction
Aortic (valve) stenosis
Occlusion and stenosis of carotid artery
Stricture of artery
Sequelae of stroke, not specified as haemorrhage or infarction
Atherosclerosis of arteries of the extremities
Dilated cardiomyopathy
Heart failure, unspecified
Cerebrovascular disease, unspecified
Subsequent myocardial infarction of unspecified site
Embolism and thrombosis of arteries of the lower extremities
Other forms of acute ischaemic heart disease
Atrioventricular block, complete
Other ill-defined heart diseases
Cardiomyopathy, unspecified
Abdominal aortic aneurysm, without mention of rupture
Pulmonary embolism without mention of acute cor pulmonale
Transient cerebral ischaemic attack, unspecified
Mitral (valve) insufficiency
Left bundle-branch block, unspecified
Ventricular tachycardia
Raynaud's syndrome
Aortic (valve) insufficiency
Atrioventricular block, first degree
<0.6 0.6-0.7 0.7-0.8 0.8-0.9 ≥0.9
C
Chronic ischaemic heart disease 0.8992
Acute myocardial infarction 0.8920
Angina pectoris 0.8718
Heart failure 0.8515
Cerebral infarction 0.8175
Essential (primary) hypertension 0.8157
Atrial fibrillation and flutter 0.8046
Nonrheumatic aortic valve disorders 0.8020
Other peripheral vascular diseases 0.7585
Atrioventricular and left bundle−branch block 0.7211
Complications and ill−defined descriptions of heart disease 0.7145
Transient cerebral ischaemic attacks and related syndromes 0.7049
Pulmonary embolism 0.6553
Other cardiac arrhythmias 0.6199
Paroxysmal tachycardia 0.6082
0.5 0.6 0.7 0.8 0.9 1.0
AUC 0.8 0.7
F
Acute transmural myocardial infarction of anterior wall 0.9110
Atherosclerotic heart disease 0.9092
Chronic ischaemic heart disease, unspecified 0.8829
Angina pectoris, unspecified 0.8741
Old myocardial infarction 0.8721
Acute transmural myocardial infarction of inferior wall 0.8637
Acute myocardial infarction, unspecified 0.8627
Left ventricular failure 0.8526
Unstable angina 0.8464
Peripheral vascular disease, unspecified 0.7983
Cerebral infarction, unspecified 0.7740
Transient cerebral ischaemic attack, unspecified 0.7239
Cardiomegaly 0.7032
Pulmonary embolism without mention of acute cor pulmonale 0.6534
Supraventricular tachycardia 0.5973
0.5 0.6 0.7 0.8 0.9 1.0
AUC 0.9 0.8 0.7 0.6

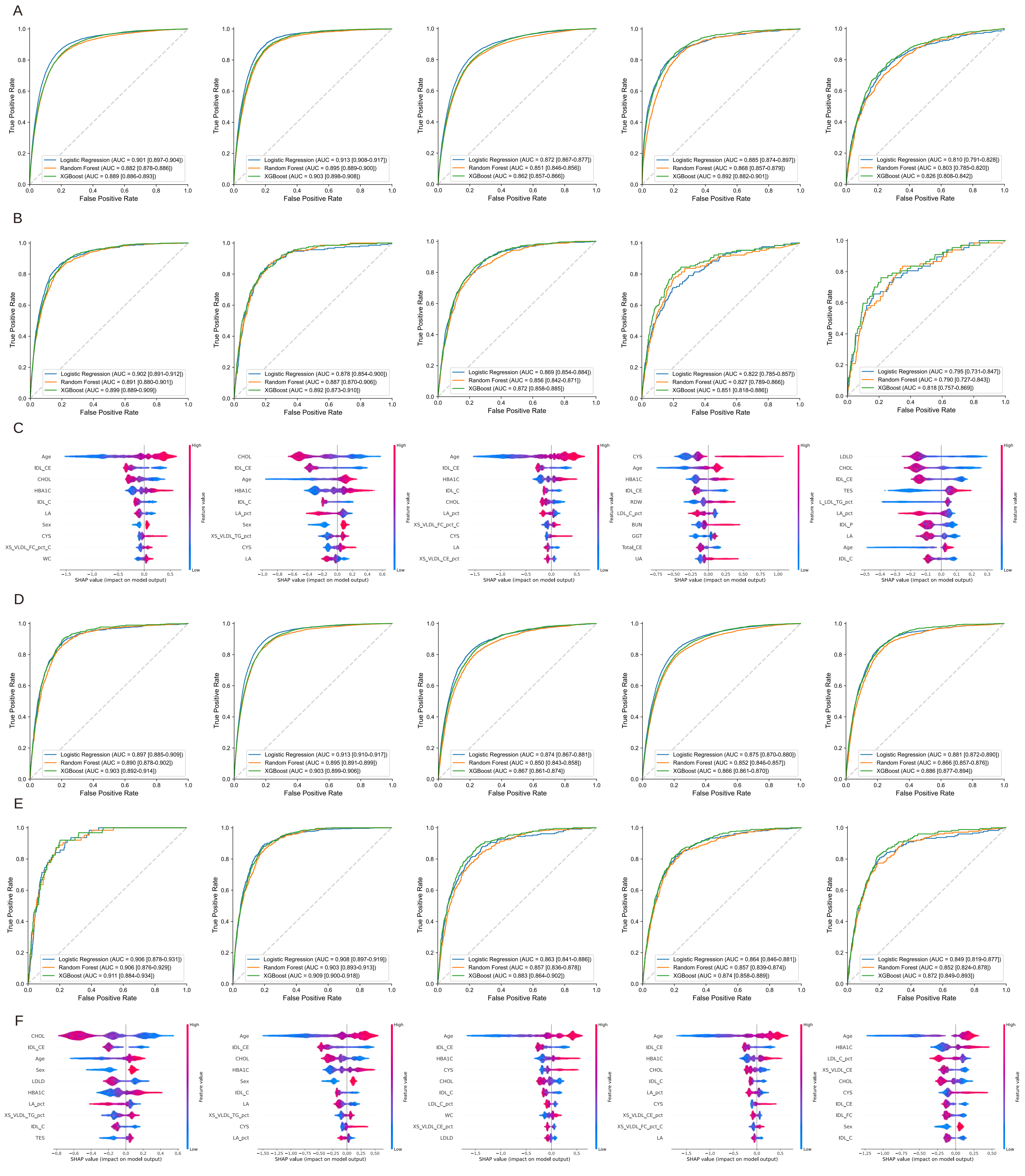
A
Logistic Regression (AUC = 0.901 [0.897-0.904])
Random Forest (AUC = 0.882 [0.878-0.886])
XGBoost (AUC = 0.889 [0.886-0.893])
Logistic Regression (AUC = 0.913 [0.908-0.917])
Random Forest (AUC = 0.895 [0.889-0.900])
XGBoost (AUC = 0.903 [0.898-0.908])
Logistic Regression (AUC = 0.872 [0.867-0.877])
Random Forest (AUC = 0.851 [0.846-0.856])
XGBoost (AUC = 0.862 [0.857-0.866])
Logistic Regression (AUC = 0.885 [0.874-0.897])
Random Forest (AUC = 0.868 [0.857-0.879])
XGBoost (AUC = 0.892 [0.882-0.901])
Logistic Regression (AUC = 0.810 [0.791-0.828])
Random Forest (AUC = 0.803 [0.785-0.820])
XGBoost (AUC = 0.826 [0.808-0.842])
B
Logistic Regression (AUC = 0.902 [0.891-0.912])
Random Forest (AUC = 0.891 [0.880-0.901])
XGBoost (AUC = 0.899 [0.889-0.909])
Logistic Regression (AUC = 0.878 [0.854-0.900])
Random Forest (AUC = 0.887 [0.870-0.906])
XGBoost (AUC = 0.892 [0.873-0.910])
Logistic Regression (AUC = 0.869 [0.854-0.884])
Random Forest (AUC = 0.856 [0.842-0.871])
XGBoost (AUC = 0.872 [0.858-0.885])
Logistic Regression (AUC = 0.822 [0.785-0.857])
Random Forest (AUC = 0.827 [0.789-0.866])
XGBoost (AUC = 0.851 [0.818-0.886])
Logistic Regression (AUC = 0.795 [0.731-0.847])
Random Forest (AUC = 0.790 [0.727-0.843])
XGBoost (AUC = 0.818 [0.757-0.869])
C
Age, IDL_CE, CHOL, HBA1C, IDL_C, LA, Sex, CYS, XS_VLDL_FC_pct_C, WC
CHOL, IDL_CE, Age, HBA1C, IDL_C, LA_pct, Sex, XS_VLDL_TG_pct, CYS, LA
Age, IDL_CE, HBA1C, IDL_C, CHOL, LA_pct, XS_VLDL_FC_pct_C, CYS, LA, XS_VLDL_CE_pct
CYS, Age, HBA1C, IDL_CE, RDW, LDL_C_pct, BUN, GGT, Total_CE, UA
LDLD, CHOL, IDL_CE, TES, L_LDL_TG_pct, LA_pct, IDL_P, LA, Age, IDL_C
D
Logistic Regression (AUC = 0.897 [0.885-0.909])
Random Forest (AUC = 0.890 [0.878-0.902])
XGBoost (AUC = 0.903 [0.892-0.914])
Logistic Regression (AUC = 0.913 [0.910-0.917])
Random Forest (AUC = 0.895 [0.891-0.899])
XGBoost (AUC = 0.903 [0.899-0.906])
Logistic Regression (AUC = 0.874 [0.867-0.881])
Random Forest (AUC = 0.850 [0.843-0.858])
XGBoost (AUC = 0.867 [0.861-0.874])
Logistic Regression (AUC = 0.875 [0.870-0.880])
Random Forest (AUC = 0.852 [0.846-0.857])
XGBoost (AUC = 0.866 [0.861-0.870])
Logistic Regression (AUC = 0.881 [0.872-0.890])
Random Forest (AUC = 0.866 [0.857-0.876])
XGBoost (AUC = 0.886 [0.877-0.894])
E
Logistic Regression (AUC = 0.906 [0.878-0.931])
Random Forest (AUC = 0.906 [0.876-0.929])
XGBoost (AUC = 0.911 [0.884-0.934])
Logistic Regression (AUC = 0.908 [0.897-0.919])
Random Forest (AUC = 0.903 [0.893-0.913])
XGBoost (AUC = 0.909 [0.900-0.918])
Logistic Regression (AUC = 0.863 [0.841-0.886])
Random Forest (AUC = 0.857 [0.836-0.878])
XGBoost (AUC = 0.883 [0.864-0.902])
Logistic Regression (AUC = 0.864 [0.846-0.881])
Random Forest (AUC = 0.857 [0.839-0.874])
XGBoost (AUC = 0.874 [0.858-0.889])
Logistic Regression (AUC = 0.849 [0.819-0.877])
Random Forest (AUC = 0.852 [0.824-0.878])
XGBoost (AUC = 0.872 [0.849-0.893])
F
CHOL, IDL_CE, Age, Sex, LDLD, HBA1C, LA_pct, XS_VLDL_TG_pct, IDL_C, TES
Age, IDL_CE, CHOL, HBA1C, Sex, IDL_C, LA, XS_VLDL_TG_pct, CYS, LA_pct
Age, IDL_CE, HBA1C, CYS, CHOL, IDL_C, LDL_C_pct, WC, XS_VLDL_CE_pct, LDLD
Age, IDL_CE, HBA1C, CHOL, IDL_C, LA_pct, CYS, XS_VLDL_CE_pct, XS_VLDL_FC_pct_C, LA
Age, HBA1C, LDL_C_pct, XS_VLDL_CE, CHOL, CYS, IDL_CE, IDL_FC, Sex, IDL_C
True Positive Rate
False Positive Rate
SHAP value (impact on model output)
Feature value
High
Low

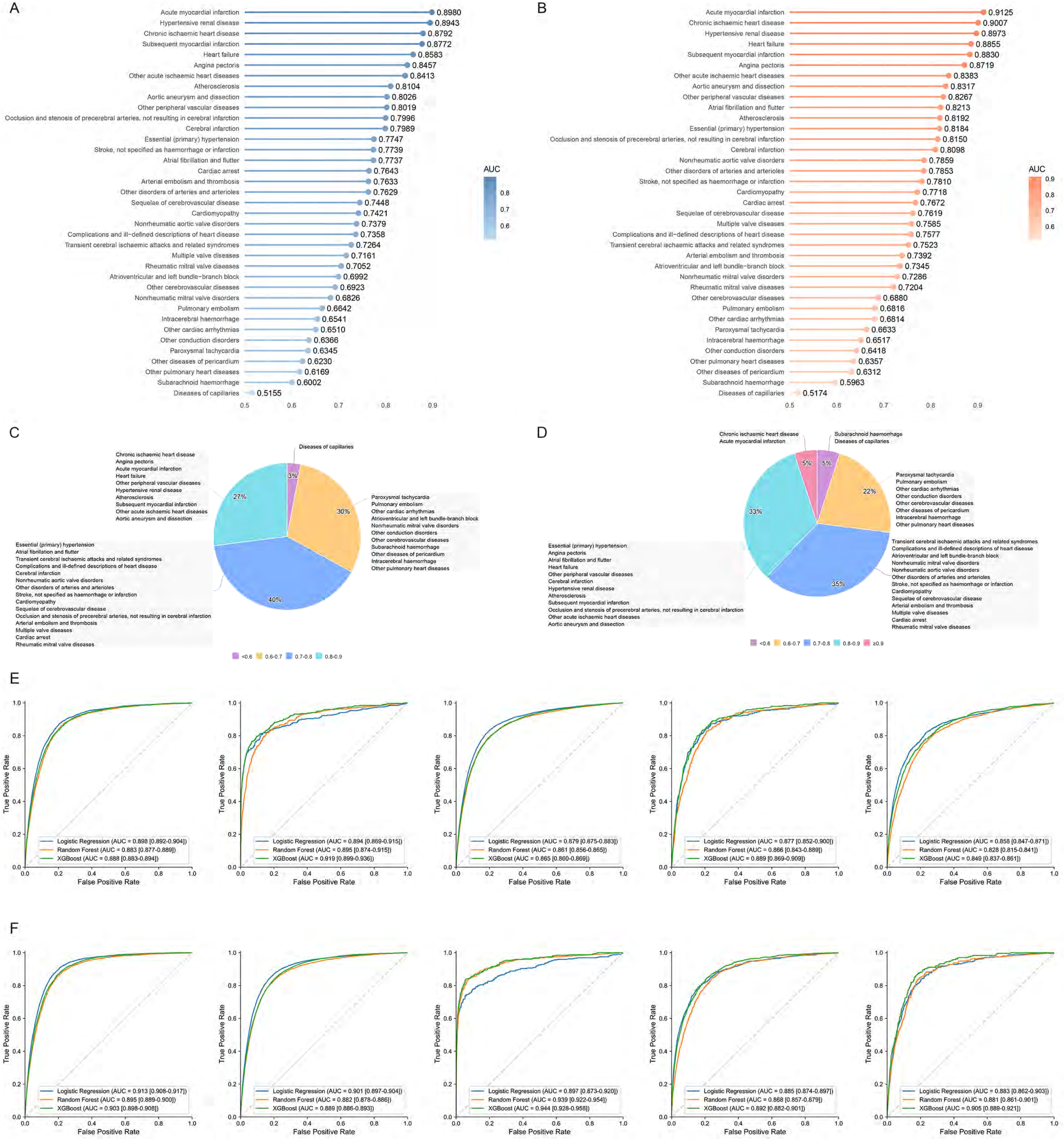
A
Acute myocardial infarction 0.8980
Hypertensive renal disease 0.8943
Chronic ischaemic heart disease 0.8792
Subsequent myocardial infarction 0.8772
Heart failure 0.8583
Angina pectoris 0.8457
Other acute ischaemic heart diseases 0.8413
Atherosclerosis 0.8104
Aortic aneurysm and dissection 0.8026
Other peripheral vascular diseases 0.8019
Occlusion and stenosis of precerebral arteries, not resulting in cerebral infarction 0.7996
Cerebral infarction 0.7989
Essential (primary) hypertension 0.7747
Stroke, not specified as haemorrhage or infarction 0.7739
Atrial fibrillation and flutter 0.7737
Cardiac arrest 0.7643
Arterial embolism and thrombosis 0.7633
Other disorders of arteries and arterioles 0.7629
Sequelae of cerebrovascular disease 0.7448
Cardiomyopathy 0.7421
Nonrheumatic aortic valve disorders 0.7379
Complications and ill-defined descriptions of heart disease 0.7358
Transient cerebral ischaemic attacks and related syndromes 0.7264
Multiple valve diseases 0.7161
Rheumatic mitral valve diseases 0.7052
Atrioventricular and left bundle-branch block 0.6992
Other cerebrovascular diseases 0.6923
Nonrheumatic mitral valve disorders 0.6826
Pulmonary embolism 0.6642
Intracerebral haemorrhage 0.6541
Other cardiac arrhythmias 0.6510
Other conduction disorders 0.6366
Paroxysmal tachycardia 0.6345
Other diseases of pericardium 0.6230
Other pulmonary heart diseases 0.6169
Subarachnoid haemorrhage 0.6002
Diseases of capillaries 0.5155
0.5 0.6 0.7 0.8 0.9
AUC
0.8 0.7 0.6
B
Acute myocardial infarction 0.9125
Chronic ischaemic heart disease 0.9007
Hypertensive renal disease 0.8973
Heart failure 0.8855
Subsequent myocardial infarction 0.8830
Angina pectoris 0.8719
Other acute ischaemic heart diseases 0.8383
Aortic aneurysm and dissection 0.8317
Other peripheral vascular diseases 0.8267
Atrial fibrillation and flutter 0.8213
Atherosclerosis 0.8192
Essential (primary) hypertension 0.8184
Occlusion and stenosis of precerebral arteries, not resulting in cerebral infarction 0.8150
Cerebral infarction 0.8098
Nonrheumatic aortic valve disorders 0.7859
Other disorders of arteries and arterioles 0.7853
Stroke, not specified as haemorrhage or infarction 0.7810
Cardiomyopathy 0.7718
Cardiac arrest 0.7672
Sequelae of cerebrovascular disease 0.7619
Multiple valve diseases 0.7585
Complications and ill-defined descriptions of heart disease 0.7577
Transient cerebral ischaemic attacks and related syndromes 0.7523
Arterial embolism and thrombosis 0.7392
Atrioventricular and left bundle-branch block 0.7345
Nonrheumatic mitral valve disorders 0.7286
Rheumatic mitral valve diseases 0.7204
Other cerebrovascular diseases 0.6880
Pulmonary embolism 0.6816
Other cardiac arrhythmias 0.6814
Paroxysmal tachycardia 0.6633
Intracerebral haemorrhage 0.6517
Other conduction disorders 0.6418
Other pulmonary heart diseases 0.6357
Other diseases of pericardium 0.6312
Subarachnoid haemorrhage 0.5963
Diseases of capillaries 0.5174
0.5 0.6 0.7 0.8 0.9
AUC
0.9 0.8 0.7 0.6
C
Diseases of capillaries
3%
Chronic ischaemic heart disease
Angina pectoris
Acute myocardial infarction
Heart failure
Other peripheral vascular diseases
Hypertensive renal disease
Atherosclerosis
Subsequent myocardial infarction
Other acute ischaemic heart diseases
Aortic aneurysm and dissection
27%
30%
Paroxysmal tachycardia
Pulmonary embolism
Other cardiac arrhythmias
Atrioventricular and left bundle-branch block
Nonrheumatic mitral valve disorders
Other conduction disorders
Other cerebrovascular diseases
Subarachnoid haemorrhage
Other diseases of pericardium
Intracerebral haemorrhage
Other pulmonary heart diseases
40%
Essential (primary) hypertension
Atrial fibrillation and flutter
Transient cerebral ischaemic attacks and related syndromes
Complications and ill-defined descriptions of heart disease
Cerebral infarction
Nonrheumatic aortic valve disorders
Other disorders of arteries and arterioles
Stroke, not specified as haemorrhage or infarction
Cardiomyopathy
Sequelae of cerebrovascular disease
Occlusion and stenosis of precerebral arteries, not resulting in cerebral infarction
Arterial embolism and thrombosis
Multiple valve diseases
Cardiac arrest
Rheumatic mitral valve diseases
<0.6 0.6-0.7 0.7-0.8 0.8-0.9
D
Chronic ischaemic heart disease
Acute myocardial infarction
Subarachnoid haemorrhage
Diseases of capillaries
5% 5%
22%
33%
35%
Paroxysmal tachycardia
Pulmonary embolism
Other cardiac arrhythmias
Other conduction disorders
Other cerebrovascular diseases
Other diseases of pericardium
Intracerebral haemorrhage
Other pulmonary heart diseases
Transient cerebral ischaemic attacks and related syndromes
Complications and ill-defined descriptions of heart disease
Atrioventricular and left bundle-branch block
Nonrheumatic mitral valve disorders
Nonrheumatic aortic valve disorders
Other disorders of arteries and arterioles
Stroke, not specified as haemorrhage or infarction
Cardiomyopathy
Sequelae of cerebrovascular disease
Arterial embolism and thrombosis
Multiple valve diseases
Cardiac arrest
Rheumatic mitral valve diseases
Essential (primary) hypertension
Angina pectoris
Atrial fibrillation and flutter
Heart failure
Other peripheral vascular diseases
Cerebral infarction
Hypertensive renal disease
Atherosclerosis
Subsequent myocardial infarction
Occlusion and stenosis of precerebral arteries, not resulting in cerebral infarction
Other acute ischaemic heart diseases
Aortic aneurysm and dissection
<0.6 0.6-0.7 0.7-0.8 0.8-0.9 ≥0.9
E
True Positive Rate
False Positive Rate
Logistic Regression (AUC = 0.898 [0.892-0.904])
Random Forest (AUC = 0.883 [0.877-0.889])
XGBoost (AUC = 0.888 [0.883-0.894])
Logistic Regression (AUC = 0.894 [0.869-0.915])
Random Forest (AUC = 0.895 [0.874-0.915])
XGBoost (AUC = 0.919 [0.899-0.936])
Logistic Regression (AUC = 0.879 [0.875-0.883])
Random Forest (AUC = 0.861 [0.856-0.865])
XGBoost (AUC = 0.865 [0.860-0.869])
Logistic Regression (AUC = 0.877 [0.852-0.900])
Random Forest (AUC = 0.866 [0.843-0.889])
XGBoost (AUC = 0.889 [0.869-0.909])
Logistic Regression (AUC = 0.858 [0.847-0.871])
Random Forest (AUC = 0.828 [0.815-0.841])
XGBoost (AUC = 0.849 [0.837-0.861])
F
True Positive Rate
False Positive Rate
Logistic Regression (AUC = 0.913 [0.908-0.917])
Random Forest (AUC = 0.895 [0.889-0.900])
XGBoost (AUC = 0.903 [0.898-0.908])
Logistic Regression (AUC = 0.901 [0.897-0.904])
Random Forest (AUC = 0.882 [0.878-0.886])
XGBoost (AUC = 0.889 [0.886-0.893])
Logistic Regression (AUC = 0.897 [0.873-0.920])
Random Forest (AUC = 0.939 [0.922-0.954])
XGBoost (AUC = 0.944 [0.928-0.958])
Logistic Regression (AUC = 0.885 [0.874-0.897])
Random Forest (AUC = 0.868 [0.857-0.879])
XGBoost (AUC = 0.892 [0.882-0.901])
Logistic Regression (AUC = 0.883 [0.862-0.903])
Random Forest (AUC = 0.881 [0.861-0.901])
XGBoost (AUC = 0.905 [0.889-0.921])

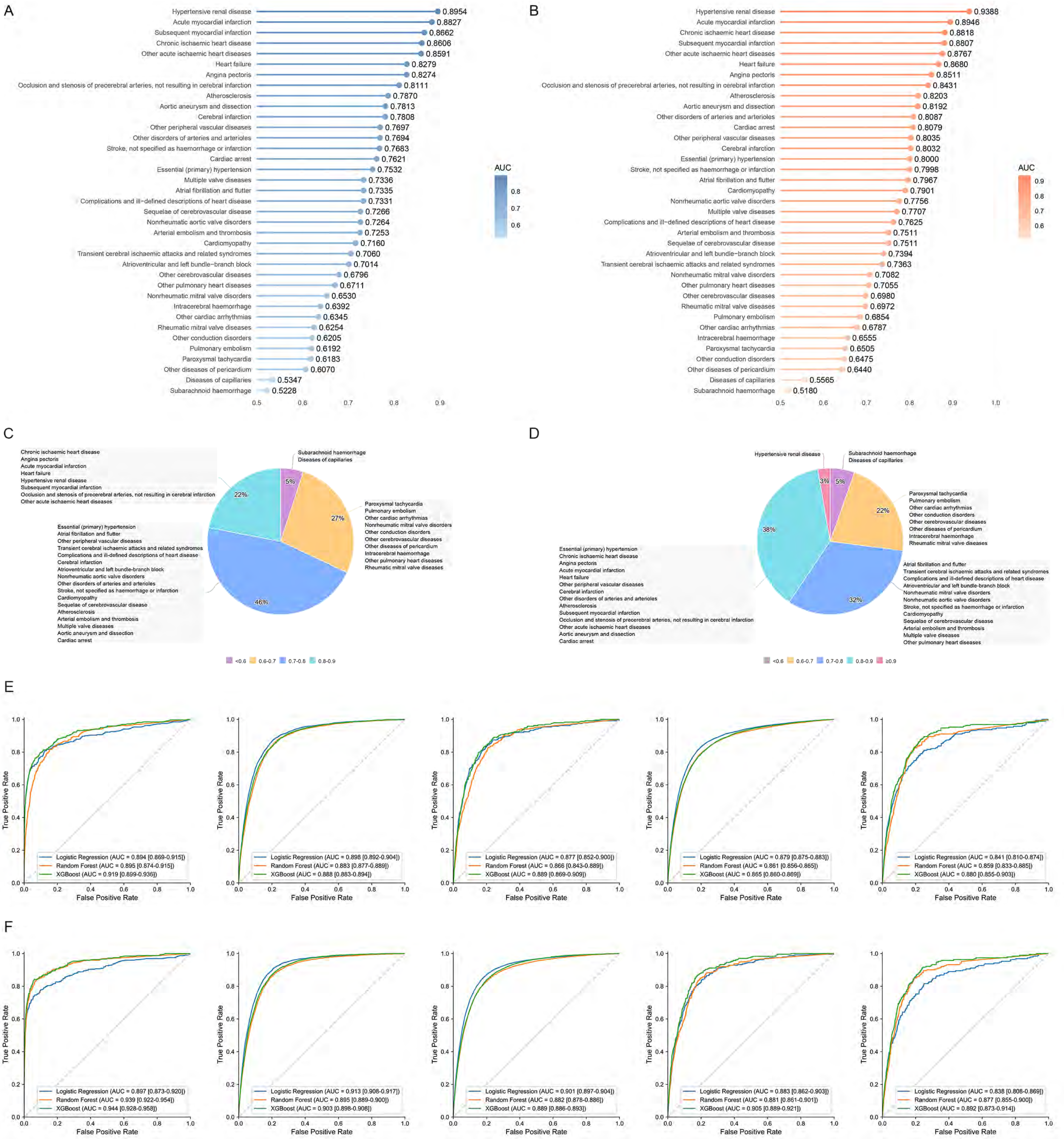
A
Hypertensive renal disease 0.8954
Acute myocardial infarction 0.8827
Subsequent myocardial infarction 0.8662
Chronic ischaemic heart disease 0.8606
Other acute ischaemic heart diseases 0.8591
Heart failure 0.8279
Angina pectoris 0.8274
Occlusion and stenosis of precerebral arteries, not resulting in cerebral infarction 0.8111
Atherosclerosis 0.7870
Aortic aneurysm and dissection 0.7813
Cerebral infarction 0.7808
Other peripheral vascular diseases 0.7697
Other disorders of arteries and arterioles 0.7694
Stroke, not specified as haemorrhage or infarction 0.7683
Cardiac arrest 0.7621
Essential (primary) hypertension 0.7532
Multiple valve diseases 0.7336
Atrial fibrillation and flutter 0.7335
Complications and ill-defined descriptions of heart disease 0.7331
Sequelae of cerebrovascular disease 0.7266
Nonrheumatic aortic valve disorders 0.7264
Arterial embolism and thrombosis 0.7253
Cardiomyopathy 0.7160
Transient cerebral ischaemic attacks and related syndromes 0.7060
Atrioventricular and left bundle-branch block 0.7014
Other cerebrovascular diseases 0.6796
Other pulmonary heart diseases 0.6711
Nonrheumatic mitral valve disorders 0.6530
Intracerebral haemorrhage 0.6392
Other cardiac arrhythmias 0.6345
Rheumatic mitral valve diseases 0.6254
Other conduction disorders 0.6205
Pulmonary embolism 0.6192
Paroxysmal tachycardia 0.6183
Other diseases of pericardium 0.6070
Diseases of capillaries 0.5347
Subarachnoid haemorrhage 0.5228
AUC
B
Hypertensive renal disease 0.9388
Acute myocardial infarction 0.8946
Chronic ischaemic heart disease 0.8818
Subsequent myocardial infarction 0.8807
Other acute ischaemic heart diseases 0.8767
Heart failure 0.8680
Angina pectoris 0.8511
Occlusion and stenosis of precerebral arteries, not resulting in cerebral infarction 0.8431
Atherosclerosis 0.8203
Aortic aneurysm and dissection 0.8192
Other disorders of arteries and arterioles 0.8087
Cardiac arrest 0.8079
Other peripheral vascular diseases 0.8035
Cerebral infarction 0.8032
Essential (primary) hypertension 0.8000
Stroke, not specified as haemorrhage or infarction 0.7998
Atrial fibrillation and flutter 0.7967
Cardiomyopathy 0.7901
Nonrheumatic aortic valve disorders 0.7756
Multiple valve diseases 0.7707
Complications and ill-defined descriptions of heart disease 0.7625
Arterial embolism and thrombosis 0.7511
Sequelae of cerebrovascular disease 0.7511
Atrioventricular and left bundle-branch block 0.7394
Transient cerebral ischaemic attacks and related syndromes 0.7363
Nonrheumatic mitral valve disorders 0.7082
Other pulmonary heart diseases 0.7055
Other cerebrovascular diseases 0.6980
Rheumatic mitral valve diseases 0.6972
Pulmonary embolism 0.6854
Other cardiac arrhythmias 0.6787
Intracerebral haemorrhage 0.6555
Paroxysmal tachycardia 0.6505
Other conduction disorders 0.6475
Other diseases of pericardium 0.6440
Diseases of capillaries 0.5565
Subarachnoid haemorrhage 0.5180
AUC
C
5%
27%
46%
22%
<0.6 0.6-0.7 0.7-0.8 0.8-0.9
D
3%
5%
22%
32%
38%
<0.6 0.6-0.7 0.7-0.8 0.8-0.9 ≥0.9
E
Logistic Regression (AUC = 0.894 [0.869-0.915])
Random Forest (AUC = 0.895 [0.874-0.915])
XGBoost (AUC = 0.919 [0.899-0.936])
Logistic Regression (AUC = 0.898 [0.892-0.904])
Random Forest (AUC = 0.883 [0.877-0.889])
XGBoost (AUC = 0.888 [0.883-0.894])
Logistic Regression (AUC = 0.877 [0.852-0.900])
Random Forest (AUC = 0.866 [0.843-0.889])
XGBoost (AUC = 0.889 [0.869-0.909])
Logistic Regression (AUC = 0.879 [0.875-0.883])
Random Forest (AUC = 0.861 [0.856-0.865])
XGBoost (AUC = 0.865 [0.860-0.869])
Logistic Regression (AUC = 0.841 [0.810-0.874])
Random Forest (AUC = 0.859 [0.833-0.885])
XGBoost (AUC = 0.880 [0.855-0.903])
F
Logistic Regression (AUC = 0.897 [0.873-0.920])
Random Forest (AUC = 0.939 [0.922-0.954])
XGBoost (AUC = 0.944 [0.928-0.958])
Logistic Regression (AUC = 0.913 [0.908-0.917])
Random Forest (AUC = 0.895 [0.889-0.900])
XGBoost (AUC = 0.903 [0.898-0.908])
Logistic Regression (AUC = 0.901 [0.897-0.904])
Random Forest (AUC = 0.882 [0.878-0.886])
XGBoost (AUC = 0.889 [0.886-0.893])
Logistic Regression (AUC = 0.883 [0.862-0.903])
Random Forest (AUC = 0.881 [0.861-0.901])
XGBoost (AUC = 0.905 [0.889-0.921])
Logistic Regression (AUC = 0.838 [0.808-0.869])
Random Forest (AUC = 0.877 [0.855-0.900])
XGBoost (AUC = 0.892 [0.873-0.914])
True Positive Rate
False Positive Rate

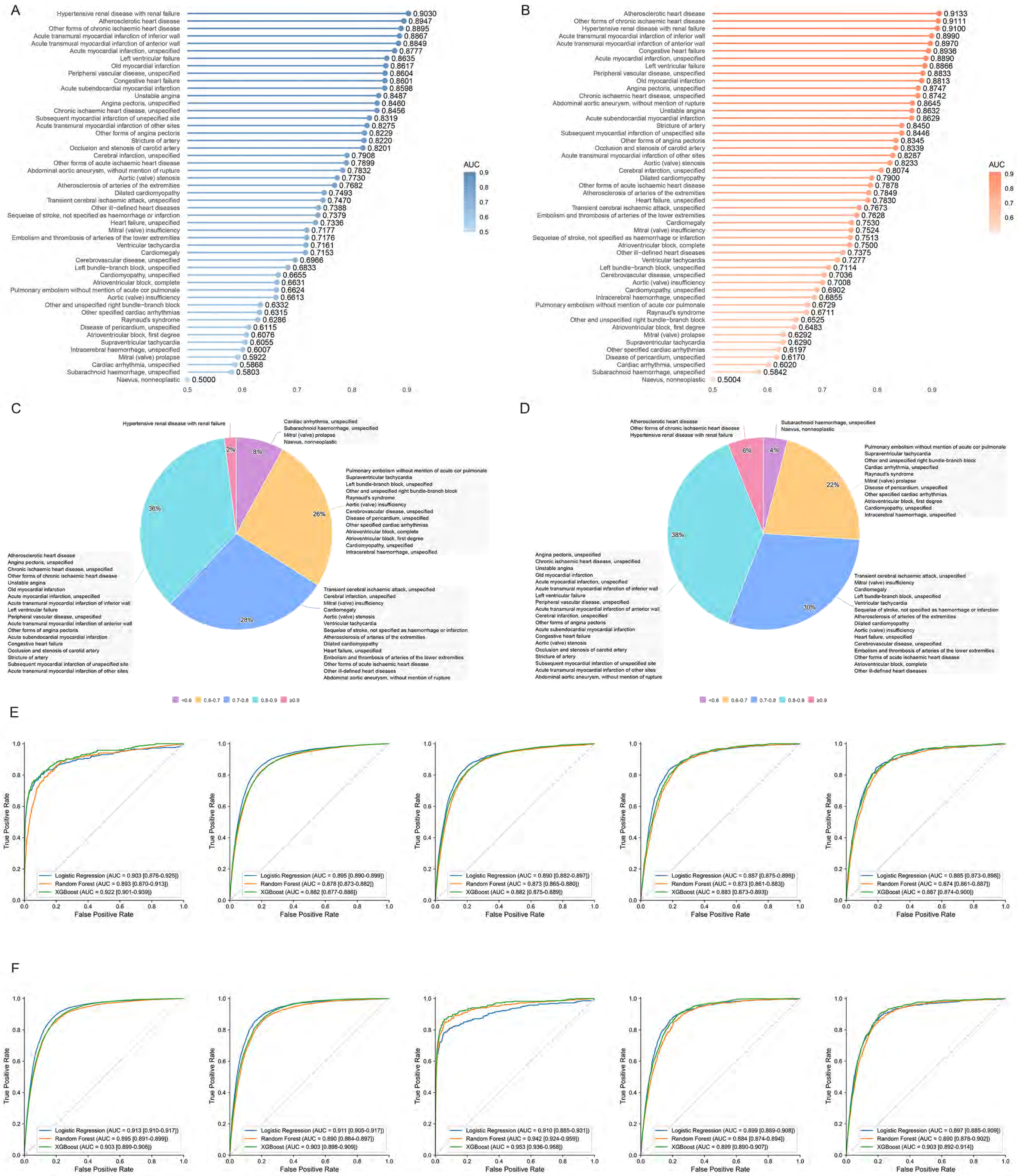
A
Hypertensive renal disease with renal failure 0.9030
Atherosclerotic heart disease 0.8947
Other forms of chronic ischaemic heart disease 0.8895
Acute transmural myocardial infarction of inferior wall 0.8867
Acute transmural myocardial infarction of anterior wall 0.8849
Acute myocardial infarction, unspecified 0.8777
Left ventricular failure 0.8635
Old myocardial infarction 0.8617
Peripheral vascular disease, unspecified 0.8604
Congestive heart failure 0.8601
Acute subendocardial myocardial infarction 0.8598
Unstable angina 0.8487
Angina pectoris, unspecified 0.8460
Chronic ischaemic heart disease, unspecified 0.8456
Subsequent myocardial infarction of unspecified site 0.8319
Acute transmural myocardial infarction of other sites 0.8275
Other forms of angina pectoris 0.8229
Stricture of artery 0.8220
Occlusion and stenosis of carotid artery 0.8201
Cerebral infarction, unspecified 0.7908
Other forms of acute ischaemic heart disease 0.7899
Abdominal aortic aneurysm, without mention of rupture 0.7832
Aortic (valve) stenosis 0.7730
Atherosclerosis of arteries of the extremities 0.7682
Dilated cardiomyopathy 0.7493
Transient cerebral ischaemic attack, unspecified 0.7470
Other ill-defined heart diseases 0.7388
Sequelae of stroke, not specified as haemorrhage or infarction 0.7379
Heart failure, unspecified 0.7336
Mitral (valve) insufficiency 0.7177
Embolism and thrombosis of arteries of the lower extremities 0.7176
Ventricular tachycardia 0.7161
Cardiomegaly 0.7153
Cerebrovascular disease, unspecified 0.6966
Left bundle-branch block, unspecified 0.6833
Cardiomyopathy, unspecified 0.6655
Atrioventricular block, complete 0.6631
Pulmonary embolism without mention of acute cor pulmonale 0.6624
Aortic (valve) insufficiency 0.6613
Other and unspecified right bundle-branch block 0.6332
Other specified cardiac arrhythmias 0.6315
Raynaud's syndrome 0.6286
Disease of pericardium, unspecified 0.6115
Atrioventricular block, first degree 0.6076
Supraventricular tachycardia 0.6055
Intracerebral haemorrhage, unspecified 0.6007
Mitral (valve) prolapse 0.5922
Cardiac arrhythmia, unspecified 0.5868
Subarachnoid haemorrhage, unspecified 0.5803
Naevus, nonneoplastic 0.5000
0.5 0.6 0.7 0.8 0.9
AUC
0.9 0.8 0.7 0.6 0.5
B
Atherosclerotic heart disease 0.9133
Other forms of chronic ischaemic heart disease 0.9111
Hypertensive renal disease with renal failure 0.9100
Acute transmural myocardial infarction of inferior wall 0.8990
Acute transmural myocardial infarction of anterior wall 0.8970
Congestive heart failure 0.8936
Acute myocardial infarction, unspecified 0.8890
Left ventricular failure 0.8866
Peripheral vascular disease, unspecified 0.8833
Old myocardial infarction 0.8813
Angina pectoris, unspecified 0.8747
Chronic ischaemic heart disease, unspecified 0.8742
Abdominal aortic aneurysm, without mention of rupture 0.8645
Unstable angina 0.8632
Acute subendocardial myocardial infarction 0.8629
Stricture of artery 0.8450
Subsequent myocardial infarction of unspecified site 0.8446
Other forms of angina pectoris 0.8345
Occlusion and stenosis of carotid artery 0.8339
Acute transmural myocardial infarction of other sites 0.8287
Aortic (valve) stenosis 0.8233
Cerebral infarction, unspecified 0.8074
Dilated cardiomyopathy 0.7900
Other forms of acute ischaemic heart disease 0.7878
Atherosclerosis of arteries of the extremities 0.7849
Heart failure, unspecified 0.7830
Transient cerebral ischaemic attack, unspecified 0.7673
Embolism and thrombosis of arteries of the lower extremities 0.7628
Cardiomegaly 0.7530
Mitral (valve) insufficiency 0.7524
Sequelae of stroke, not specified as haemorrhage or infarction 0.7513
Atrioventricular block, complete 0.7500
Other ill-defined heart diseases 0.7375
Ventricular tachycardia 0.7277
Left bundle-branch block, unspecified 0.7114
Cerebrovascular disease, unspecified 0.7036
Aortic (valve) insufficiency 0.7008
Cardiomyopathy, unspecified 0.6902
Intracerebral haemorrhage, unspecified 0.6855
Pulmonary embolism without mention of acute cor pulmonale 0.6729
Raynaud's syndrome 0.6711
Other and unspecified right bundle-branch block 0.6525
Atrioventricular block, first degree 0.6483
Mitral (valve) prolapse 0.6292
Supraventricular tachycardia 0.6290
Other specified cardiac arrhythmias 0.6197
Disease of pericardium, unspecified 0.6170
Cardiac arrhythmia, unspecified 0.6020
Subarachnoid haemorrhage, unspecified 0.5842
Naevus, nonneoplastic 0.5004
0.5 0.6 0.7 0.8 0.9
AUC
0.9 0.8 0.7 0.6
C
Hypertensive renal disease with renal failure
2%
Cardiac arrhythmia, unspecified
Subarachnoid haemorrhage, unspecified
Mitral (valve) prolapse
Naevus, nonneoplastic
8%
Pulmonary embolism without mention of acute cor pulmonale
Supraventricular tachycardia
Left bundle-branch block, unspecified
Other and unspecified right bundle-branch block
Raynaud's syndrome
Aortic (valve) insufficiency
Cerebrovascular disease, unspecified
Disease of pericardium, unspecified
Other specified cardiac arrhythmias
Atrioventricular block, complete
Atrioventricular block, first degree
Cardiomyopathy, unspecified
Intracerebral haemorrhage, unspecified
26%
Transient cerebral ischaemic attack, unspecified
Cerebral infarction, unspecified
Mitral (valve) insufficiency
Cardiomegaly
Aortic (valve) stenosis
Ventricular tachycardia
Sequelae of stroke, not specified as haemorrhage or infarction
Atherosclerosis of arteries of the extremities
Dilated cardiomyopathy
Heart failure, unspecified
Embolism and thrombosis of arteries of the lower extremities
Other forms of acute ischaemic heart disease
Other ill-defined heart diseases
Abdominal aortic aneurysm, without mention of rupture
28%
36%
Atherosclerotic heart disease
Angina pectoris, unspecified
Chronic ischaemic heart disease, unspecified
Other forms of chronic ischaemic heart disease
Unstable angina
Old myocardial infarction
Acute myocardial infarction, unspecified
Acute transmural myocardial infarction of inferior wall
Left ventricular failure
Peripheral vascular disease, unspecified
Acute transmural myocardial infarction of anterior wall
Other forms of angina pectoris
Acute subendocardial myocardial infarction
Congestive heart failure
Occlusion and stenosis of carotid artery
Stricture of artery
Subsequent myocardial infarction of unspecified site
Acute transmural myocardial infarction of other sites
<0.6 0.6-0.7 0.7-0.8 0.8-0.9 ≥0.9
D
Atherosclerotic heart disease
Other forms of chronic ischaemic heart disease
Hypertensive renal disease with renal failure
6%
Subarachnoid haemorrhage, unspecified
Naevus, nonneoplastic
4%
Pulmonary embolism without mention of acute cor pulmonale
Supraventricular tachycardia
Other and unspecified right bundle-branch block
Cardiac arrhythmia, unspecified
Raynaud's syndrome
Mitral (valve) prolapse
Disease of pericardium, unspecified
Other specified cardiac arrhythmias
Atrioventricular block, first degree
Cardiomyopathy, unspecified
Intracerebral haemorrhage, unspecified
22%
38%
Angina pectoris, unspecified
Chronic ischaemic heart disease, unspecified
Unstable angina
Old myocardial infarction
Acute myocardial infarction, unspecified
Acute transmural myocardial infarction of inferior wall
Left ventricular failure
Peripheral vascular disease, unspecified
Acute transmural myocardial infarction of anterior wall
Cerebral infarction, unspecified
Other forms of angina pectoris
Acute subendocardial myocardial infarction
Congestive heart failure
Aortic (valve) stenosis
Occlusion and stenosis of carotid artery
Stricture of artery
Subsequent myocardial infarction of unspecified site
Acute transmural myocardial infarction of other sites
Abdominal aortic aneurysm, without mention of rupture
30%
Transient cerebral ischaemic attack, unspecified
Mitral (valve) insufficiency
Cardiomegaly
Left bundle-branch block, unspecified
Ventricular tachycardia
Sequelae of stroke, not specified as haemorrhage or infarction
Atherosclerosis of arteries of the extremities
Dilated cardiomyopathy
Aortic (valve) insufficiency
Heart failure, unspecified
Cerebrovascular disease, unspecified
Embolism and thrombosis of arteries of the lower extremities
Other forms of acute ischaemic heart disease
Atrioventricular block, complete
Other ill-defined heart diseases
<0.6 0.6-0.7 0.7-0.8 0.8-0.9 ≥0.9
E
True Positive Rate
False Positive Rate
Logistic Regression (AUC = 0.903 [0.876-0.925])
Random Forest (AUC = 0.893 [0.870-0.913])
XGBoost (AUC = 0.922 [0.901-0.939])
Logistic Regression (AUC = 0.895 [0.890-0.899])
Random Forest (AUC = 0.878 [0.873-0.882])
XGBoost (AUC = 0.882 [0.877-0.886])
Logistic Regression (AUC = 0.890 [0.882-0.897])
Random Forest (AUC = 0.873 [0.865-0.880])
XGBoost (AUC = 0.882 [0.875-0.889])
Logistic Regression (AUC = 0.887 [0.875-0.898])
Random Forest (AUC = 0.873 [0.861-0.883])
XGBoost (AUC = 0.883 [0.873-0.893])
Logistic Regression (AUC = 0.885 [0.873-0.898])
Random Forest (AUC = 0.874 [0.861-0.887])
XGBoost (AUC = 0.887 [0.874-0.900])
F
True Positive Rate
False Positive Rate
Logistic Regression (AUC = 0.913 [0.910-0.917])
Random Forest (AUC = 0.895 [0.891-0.899])
XGBoost (AUC = 0.903 [0.899-0.906])
Logistic Regression (AUC = 0.911 [0.905-0.917])
Random Forest (AUC = 0.890 [0.884-0.897])
XGBoost (AUC = 0.903 [0.898-0.909])
Logistic Regression (AUC = 0.910 [0.885-0.931])
Random Forest (AUC = 0.942 [0.924-0.959])
XGBoost (AUC = 0.953 [0.936-0.968])
Logistic Regression (AUC = 0.899 [0.889-0.908])
Random Forest (AUC = 0.884 [0.874-0.894])
XGBoost (AUC = 0.899 [0.890-0.907])
Logistic Regression (AUC = 0.897 [0.885-0.909])
Random Forest (AUC = 0.890 [0.878-0.902])
XGBoost (AUC = 0.903 [0.892-0.914])

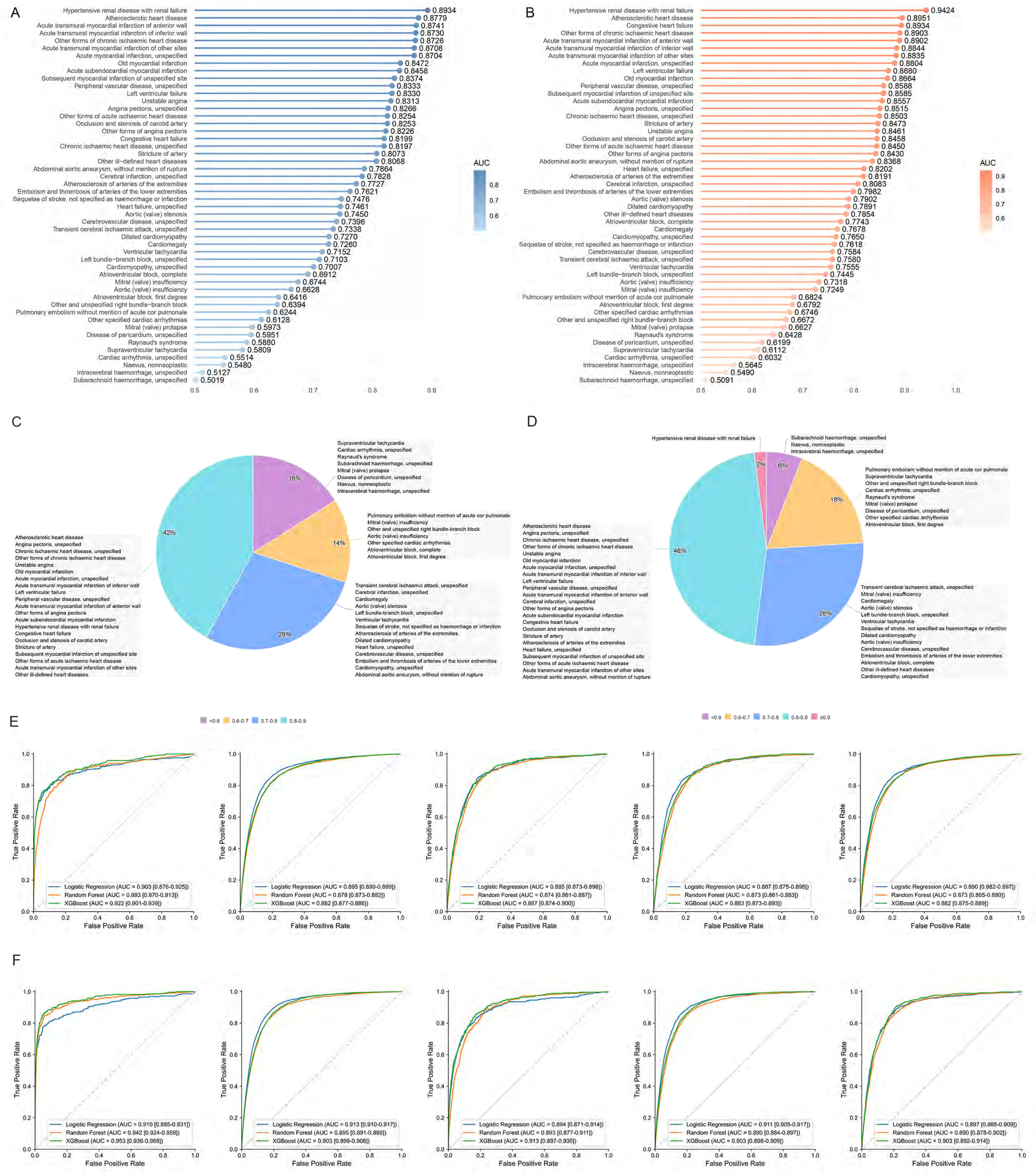

A
Hypertensive renal disease with renal failure 0.8934
Atherosclerotic heart disease 0.8779
Acute transmural myocardial infarction of anterior wall 0.8741
Acute transmural myocardial infarction of inferior wall 0.8730
Other forms of chronic ischaemic heart disease 0.8726
Acute transmural myocardial infarction of other sites 0.8708
Acute myocardial infarction, unspecified 0.8704
Old myocardial infarction 0.8472
Acute subendocardial myocardial infarction 0.8458
Subsequent myocardial infarction of unspecified site 0.8374
Peripheral vascular disease, unspecified 0.8333
Left ventricular failure 0.8330
Unstable angina 0.8313
Angina pectoris, unspecified 0.8266
Other forms of acute ischaemic heart disease 0.8254
Occlusion and stenosis of carotid artery 0.8253
Other forms of angina pectoris 0.8226
Congestive heart failure 0.8199
Chronic ischaemic heart disease, unspecified 0.8197
Stricture of artery 0.8073
Other ill-defined heart diseases 0.8068
Abdominal aortic aneurysm, without mention of rupture 0.7864
Cerebral infarction, unspecified 0.7828
Atherosclerosis of arteries of the extremities 0.7727
Embolism and thrombosis of arteries of the lower extremities 0.7621
Sequelae of stroke, not specified as haemorrhage or infarction 0.7476
Heart failure, unspecified 0.7461
Aortic (valve) stenosis 0.7450
Cerebrovascular disease, unspecified 0.7396
Transient cerebral ischaemic attack, unspecified 0.7338
Dilated cardiomyopathy 0.7270
Cardiomegaly 0.7260
Ventricular tachycardia 0.7152
Left bundle-branch block, unspecified 0.7103
Cardiomyopathy, unspecified 0.7007
Atrioventricular block, complete 0.6912
Mitral (valve) insufficiency 0.6744
Aortic (valve) insufficiency 0.6628
Atrioventricular block, first degree 0.6416
Other and unspecified right bundle-branch block 0.6394
Pulmonary embolism without mention of acute cor pulmonale 0.6244
Other specified cardiac arrhythmias 0.6128
Mitral (valve) prolapse 0.5973
Disease of pericardium, unspecified 0.5951
Raynaud's syndrome 0.5880
Supraventricular tachycardia 0.5809
Cardiac arrhythmia, unspecified 0.5514
Naevus, nonneoplastic 0.5480
Intracerebral haemorrhage, unspecified 0.5127
Subarachnoid haemorrhage, unspecified 0.5019
0.5 0.6 0.7 0.8 0.9
AUC
0.8
0.7
0.6
B
Hypertensive renal disease with renal failure 0.9424
Atherosclerotic heart disease 0.8951
Congestive heart failure 0.8934
Other forms of chronic ischaemic heart disease 0.8903
Acute transmural myocardial infarction of anterior wall 0.8902
Acute transmural myocardial infarction of inferior wall 0.8844
Acute transmural myocardial infarction of other sites 0.8835
Acute myocardial infarction, unspecified 0.8804
Left ventricular failure 0.8680
Old myocardial infarction 0.8664
Peripheral vascular disease, unspecified 0.8588
Subsequent myocardial infarction of unspecified site 0.8585
Acute subendocardial myocardial infarction 0.8557
Angina pectoris, unspecified 0.8515
Chronic ischaemic heart disease, unspecified 0.8503
Stricture of artery 0.8473
Unstable angina 0.8461
Occlusion and stenosis of carotid artery 0.8458
Other forms of acute ischaemic heart disease 0.8450
Other forms of angina pectoris 0.8430
Abdominal aortic aneurysm, without mention of rupture 0.8368
Heart failure, unspecified 0.8202
Atherosclerosis of arteries of the extremities 0.8191
Cerebral infarction, unspecified 0.8083
Embolism and thrombosis of arteries of the lower extremities 0.7982
Aortic (valve) stenosis 0.7902
Dilated cardiomyopathy 0.7891
Other ill-defined heart diseases 0.7854
Atrioventricular block, complete 0.7743
Cardiomegaly 0.7678
Cardiomyopathy, unspecified 0.7650
Sequelae of stroke, not specified as haemorrhage or infarction 0.7618
Cerebrovascular disease, unspecified 0.7584
Transient cerebral ischaemic attack, unspecified 0.7580
Ventricular tachycardia 0.7555
Left bundle-branch block, unspecified 0.7445
Aortic (valve) insufficiency 0.7318
Mitral (valve) insufficiency 0.7249
Pulmonary embolism without mention of acute cor pulmonale 0.6824
Atrioventricular block, first degree 0.6792
Other specified cardiac arrhythmias 0.6746
Other and unspecified right bundle-branch block 0.6672
Mitral (valve) prolapse 0.6627
Raynaud's syndrome 0.6428
Disease of pericardium, unspecified 0.6199
Supraventricular tachycardia 0.6112
Cardiac arrhythmia, unspecified 0.6032
Intracerebral haemorrhage, unspecified 0.5645
Naevus, nonneoplastic 0.5490
Subarachnoid haemorrhage, unspecified 0.5091
0.5 0.6 0.7 0.8 0.9 1.0
AUC
0.9
0.8
0.7
0.6
C
16%
14%
28%
42%
Supraventricular tachycardia
Cardiac arrhythmia, unspecified
Raynaud's syndrome
Subarachnoid haemorrhage, unspecified
Mitral (valve) prolapse
Disease of pericardium, unspecified
Naevus, nonneoplastic
Intracerebral haemorrhage, unspecified
Pulmonary embolism without mention of acute cor pulmonale
Mitral (valve) insufficiency
Other and unspecified right bundle-branch block
Aortic (valve) insufficiency
Other specified cardiac arrhythmias
Atrioventricular block, complete
Atrioventricular block, first degree
Transient cerebral ischaemic attack, unspecified
Cerebral infarction, unspecified
Cardiomegaly
Aortic (valve) stenosis
Left bundle-branch block, unspecified
Ventricular tachycardia
Sequelae of stroke, not specified as haemorrhage or infarction
Atherosclerosis of arteries of the extremities
Dilated cardiomyopathy
Heart failure, unspecified
Cerebrovascular disease, unspecified
Embolism and thrombosis of arteries of the lower extremities
Cardiomyopathy, unspecified
Abdominal aortic aneurysm, without mention of rupture
Atherosclerotic heart disease
Angina pectoris, unspecified
Chronic ischaemic heart disease, unspecified
Other forms of chronic ischaemic heart disease
Unstable angina
Old myocardial infarction
Acute myocardial infarction, unspecified
Acute transmural myocardial infarction of inferior wall
Left ventricular failure
Peripheral vascular disease, unspecified
Acute transmural myocardial infarction of anterior wall
Other forms of angina pectoris
Acute subendocardial myocardial infarction
Hypertensive renal disease with renal failure
Congestive heart failure
Occlusion and stenosis of carotid artery
Stricture of artery
Subsequent myocardial infarction of unspecified site
Other forms of acute ischaemic heart disease
Acute transmural myocardial infarction of other sites
Other ill-defined heart diseases
D
Hypertensive renal disease with renal failure
2%
6%
18%
28%
46%
Subarachnoid haemorrhage, unspecified
Naevus, nonneoplastic
Intracerebral haemorrhage, unspecified
Pulmonary embolism without mention of acute cor pulmonale
Supraventricular tachycardia
Other and unspecified right bundle-branch block
Cardiac arrhythmia, unspecified
Raynaud's syndrome
Mitral (valve) prolapse
Disease of pericardium, unspecified
Other specified cardiac arrhythmias
Atrioventricular block, first degree
Atherosclerotic heart disease
Angina pectoris, unspecified
Chronic ischaemic heart disease, unspecified
Other forms of chronic ischaemic heart disease
Unstable angina
Old myocardial infarction
Acute myocardial infarction, unspecified
Acute transmural myocardial infarction of inferior wall
Left ventricular failure
Peripheral vascular disease, unspecified
Acute transmural myocardial infarction of anterior wall
Cerebral infarction, unspecified
Other forms of angina pectoris
Acute subendocardial myocardial infarction
Congestive heart failure
Occlusion and stenosis of carotid artery
Stricture of artery
Atherosclerosis of arteries of the extremities
Heart failure, unspecified
Subsequent myocardial infarction of unspecified site
Other forms of acute ischaemic heart disease
Acute transmural myocardial infarction of other sites
Abdominal aortic aneurysm, without mention of rupture
Transient cerebral ischaemic attack, unspecified
Mitral (valve) insufficiency
Cardiomegaly
Aortic (valve) stenosis
Left bundle-branch block, unspecified
Ventricular tachycardia
Sequelae of stroke, not specified as haemorrhage or infarction
Dilated cardiomyopathy
Aortic (valve) insufficiency
Cerebrovascular disease, unspecified
Embolism and thrombosis of arteries of the lower extremities
Atrioventricular block, complete
Other ill-defined heart diseases
Cardiomyopathy, unspecified
<0.6 0.6-0.7 0.7-0.8 0.8-0.9
<0.6 0.6-0.7 0.7-0.8 0.8-0.9 ≥0.9
E
True Positive Rate
False Positive Rate
Logistic Regression (AUC = 0.903 [0.876-0.925])
Random Forest (AUC = 0.893 [0.870-0.913])
XGBoost (AUC = 0.922 [0.901-0.939])
Logistic Regression (AUC = 0.895 [0.890-0.899])
Random Forest (AUC = 0.878 [0.873-0.882])
XGBoost (AUC = 0.882 [0.877-0.886])
Logistic Regression (AUC = 0.885 [0.873-0.898])
Random Forest (AUC = 0.874 [0.861-0.887])
XGBoost (AUC = 0.887 [0.874-0.900])
Logistic Regression (AUC = 0.887 [0.875-0.898])
Random Forest (AUC = 0.873 [0.861-0.883])
XGBoost (AUC = 0.883 [0.873-0.893])
Logistic Regression (AUC = 0.890 [0.882-0.897])
Random Forest (AUC = 0.873 [0.865-0.880])
XGBoost (AUC = 0.882 [0.875-0.889])
F
Logistic Regression (AUC = 0.910 [0.885-0.931])
Random Forest (AUC = 0.942 [0.924-0.959])
XGBoost (AUC = 0.953 [0.936-0.968])
Logistic Regression (AUC = 0.913 [0.910-0.917])
Random Forest (AUC = 0.895 [0.891-0.899])
XGBoost (AUC = 0.903 [0.899-0.906])
Logistic Regression (AUC = 0.894 [0.871-0.914])
Random Forest (AUC = 0.893 [0.877-0.911])
XGBoost (AUC = 0.913 [0.897-0.930])
Logistic Regression (AUC = 0.911 [0.905-0.917])
Random Forest (AUC = 0.890 [0.884-0.897])
XGBoost (AUC = 0.903 [0.898-0.909])
Logistic Regression (AUC = 0.897 [0.885-0.909])
Random Forest (AUC = 0.890 [0.878-0.902])
XGBoost (AUC = 0.903 [0.892-0.914])

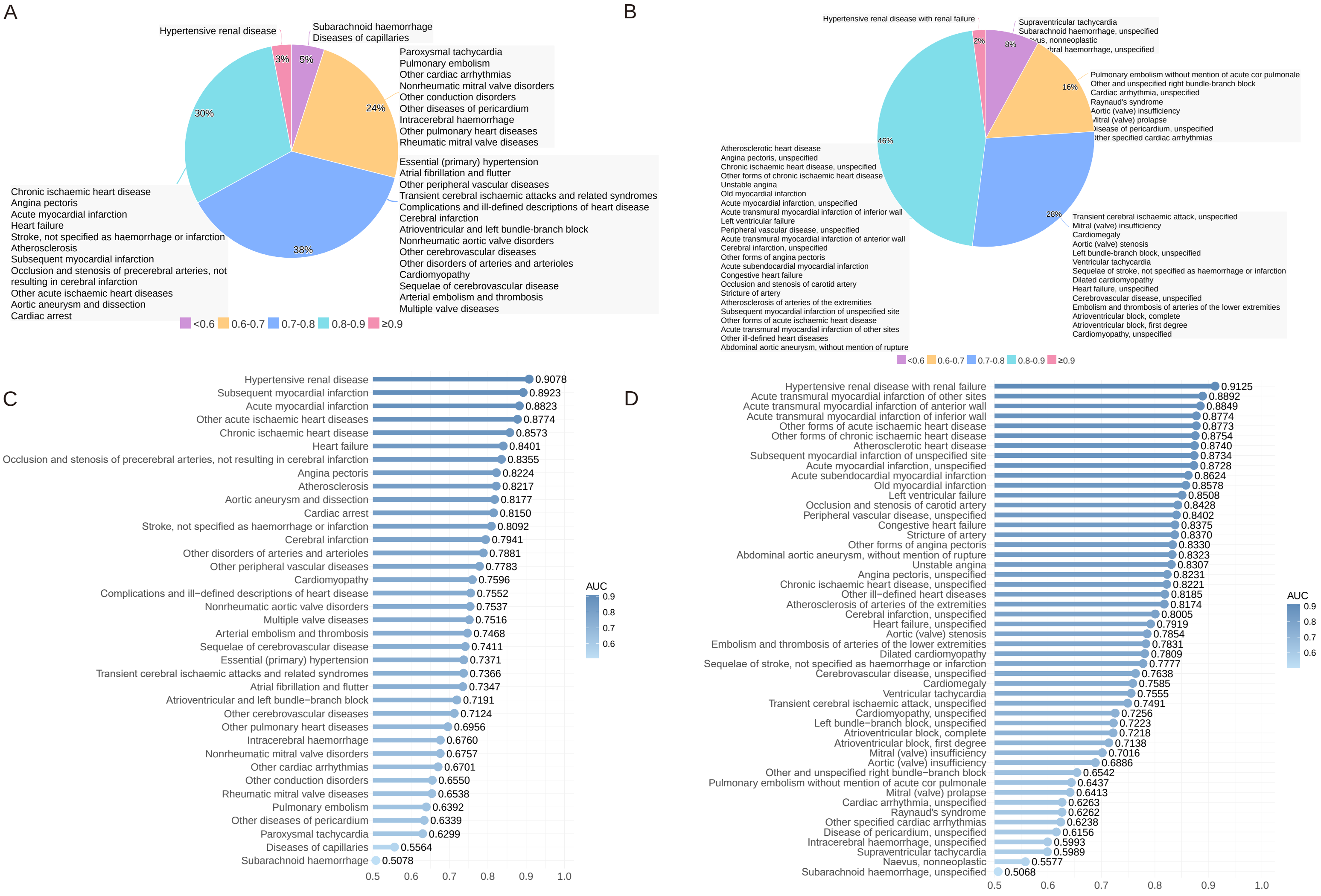

A
Hypertensive renal disease
Subarachnoid haemorrhage
Diseases of capillaries
3%
5%
24%
30%
38%
Paroxysmal tachycardia
Pulmonary embolism
Other cardiac arrhythmias
Nonrheumatic mitral valve disorders
Other conduction disorders
Other diseases of pericardium
Intracerebral haemorrhage
Other pulmonary heart diseases
Rheumatic mitral valve diseases
Essential (primary) hypertension
Atrial fibrillation and flutter
Other peripheral vascular diseases
Transient cerebral ischaemic attacks and related syndromes
Complications and ill-defined descriptions of heart disease
Cerebral infarction
Atrioventricular and left bundle-branch block
Nonrheumatic aortic valve disorders
Other cerebrovascular diseases
Other disorders of arteries and arterioles
Cardiomyopathy
Sequelae of cerebrovascular disease
Arterial embolism and thrombosis
Multiple valve diseases
Chronic ischaemic heart disease
Angina pectoris
Acute myocardial infarction
Heart failure
Stroke, not specified as haemorrhage or infarction
Atherosclerosis
Subsequent myocardial infarction
Occlusion and stenosis of precerebral arteries, not resulting in cerebral infarction
Other acute ischaemic heart diseases
Aortic aneurysm and dissection
Cardiac arrest
<0.6 0.6-0.7 0.7-0.8 0.8-0.9 ≥0.9
B
Hypertensive renal disease with renal failure
Supraventricular tachycardia
Subarachnoid haemorrhage, unspecified
2%
8%
16%
46%
28%
Pulmonary embolism without mention of acute cor pulmonale
Other and unspecified right bundle-branch block
Cardiac arrhythmia, unspecified
Raynaud's syndrome
Aortic (valve) insufficiency
Atherosclerotic heart disease
Angina pectoris, unspecified
Chronic ischaemic heart disease, unspecified
Other forms of chronic ischaemic heart disease
Unstable angina
Old myocardial infarction
Acute myocardial infarction, unspecified
Acute transmural myocardial infarction of inferior wall
Left ventricular failure
Peripheral vascular disease, unspecified
Acute transmural myocardial infarction of anterior wall
Cerebral infarction, unspecified
Other forms of angina pectoris
Acute subendocardial myocardial infarction
Congestive heart failure
Occlusion and stenosis of carotid artery
Stricture of artery
Atherosclerosis of arteries of the extremities
Subsequent myocardial infarction of unspecified site
Other forms of acute ischaemic heart disease
Acute transmural myocardial infarction of other sites
Other ill-defined heart diseases
Abdominal aortic aneurysm, without mention of rupture
Transient cerebral ischaemic attack, unspecified
Mitral (valve) insufficiency
Cardiomegaly
Aortic (valve) stenosis
Left bundle-branch block, unspecified
Ventricular tachycardia
Sequelae of stroke, not specified as haemorrhage or infarction
Dilated cardiomyopathy
Heart failure, unspecified
Cerebrovascular disease, unspecified
Embolism and thrombosis of arteries of the lower extremities
Atrioventricular block, complete
Atrioventricular block, first degree
Cardiomyopathy, unspecified
<0.6 0.6-0.7 0.7-0.8 0.8-0.9 ≥0.9
C
Hypertensive renal disease 0.9078
Subsequent myocardial infarction 0.8923
Acute myocardial infarction 0.8823
Other acute ischaemic heart diseases 0.8774
Chronic ischaemic heart disease 0.8573
Heart failure 0.8401
Occlusion and stenosis of precerebral arteries, not resulting in cerebral infarction 0.8355
Angina pectoris 0.8224
Atherosclerosis 0.8217
Aortic aneurysm and dissection 0.8177
Cardiac arrest 0.8150
Stroke, not specified as haemorrhage or infarction 0.8092
Cerebral infarction 0.7941
Other disorders of arteries and arterioles 0.7881
Other peripheral vascular diseases 0.7783
Cardiomyopathy 0.7596
Complications and ill−defined descriptions of heart disease 0.7552
Nonrheumatic aortic valve disorders 0.7537
Multiple valve diseases 0.7516
Arterial embolism and thrombosis 0.7468
Sequelae of cerebrovascular disease 0.7411
Essential (primary) hypertension 0.7371
Transient cerebral ischaemic attacks and related syndromes 0.7366
Atrial fibrillation and flutter 0.7347
Atrioventricular and left bundle−branch block 0.7191
Other cerebrovascular diseases 0.7124
Other pulmonary heart diseases 0.6956
Intracerebral haemorrhage 0.6760
Nonrheumatic mitral valve disorders 0.6757
Other cardiac arrhythmias 0.6701
Other conduction disorders 0.6550
Rheumatic mitral valve diseases 0.6538
Pulmonary embolism 0.6392
Other diseases of pericardium 0.6339
Paroxysmal tachycardia 0.6299
Diseases of capillaries 0.5564
Subarachnoid haemorrhage 0.5078
0.5 0.6 0.7 0.8 0.9 1.0
AUC
0.9
0.8
0.7
0.6
D
Hypertensive renal disease with renal failure 0.9125
Acute transmural myocardial infarction of other sites 0.8892
Acute transmural myocardial infarction of anterior wall 0.8849
Acute transmural myocardial infarction of inferior wall 0.8774
Other forms of acute ischaemic heart disease 0.8773
Other forms of chronic ischaemic heart disease 0.8754
Atherosclerotic heart disease 0.8740
Subsequent myocardial infarction of unspecified site 0.8734
Acute myocardial infarction, unspecified 0.8728
Acute subendocardial myocardial infarction 0.8624
Old myocardial infarction 0.8578
Left ventricular failure 0.8508
Occlusion and stenosis of carotid artery 0.8428
Peripheral vascular disease, unspecified 0.8402
Congestive heart failure 0.8375
Stricture of artery 0.8370
Other forms of angina pectoris 0.8330
Abdominal aortic aneurysm, without mention of rupture 0.8323
Unstable angina 0.8307
Angina pectoris, unspecified 0.8231
Chronic ischaemic heart disease, unspecified 0.8221
Other ill−defined heart diseases 0.8185
Atherosclerosis of arteries of the extremities 0.8174
Cerebral infarction, unspecified 0.8005
Heart failure, unspecified 0.7919
Aortic (valve) stenosis 0.7854
Embolism and thrombosis of arteries of the lower extremities 0.7831
Dilated cardiomyopathy 0.7809
Sequelae of stroke, not specified as haemorrhage or infarction 0.7777
Cerebrovascular disease, unspecified 0.7638
Cardiomegaly 0.7585
Ventricular tachycardia 0.7555
Transient cerebral ischaemic attack, unspecified 0.7491
Cardiomyopathy, unspecified 0.7256
Left bundle−branch block, unspecified 0.7223
Atrioventricular block, complete 0.7218
Atrioventricular block, first degree 0.7138
Mitral (valve) insufficiency 0.7016
Aortic (valve) insufficiency 0.6886
Other and unspecified right bundle−branch block 0.6542
Pulmonary embolism without mention of acute cor pulmonale 0.6437
Mitral (valve) prolapse 0.6413
Cardiac arrhythmia, unspecified 0.6263
Raynaud's syndrome 0.6262
Other specified cardiac arrhythmias 0.6238
Disease of pericardium, unspecified 0.6156
Intracerebral haemorrhage, unspecified 0.5993
Supraventricular tachycardia 0.5989
Naevus, nonneoplastic 0.5577
Subarachnoid haemorrhage, unspecified 0.5068
0.5 0.6 0.7 0.8 0.9 1.0
AUC
0.9
0.8
0.7
0.6

Table S1. Distribution of CVD cases stratified by class and subclass in the study cohort

ICD-10 disease code	Disease	Count
G45	Transient cerebral ischaemic attacks and related syndromes	644
G45.9	Transient cerebral ischaemic attack, unspecified	533
I05	Rheumatic mitral valve diseases	102
I08	Multiple valve diseases	211
I10	Essential (primary) hypertension	17979
I12	Hypertensive renal disease	343
I12.0	Hypertensive renal disease with renal failure	306
I20	Angina pectoris	6055
I20.0	Unstable angina	1958
I20.8	Other forms of angina pectoris	376
I20.9	Angina pectoris, unspecified	4874
I21	Acute myocardial infarction	2592
I21.0	Acute transmural myocardial infarction of anterior wall	639
I21.1	Acute transmural myocardial infarction of inferior wall	805
I21.2	Acute transmural myocardial infarction of other sites	108
I21.4	Acute subendocardial myocardial infarction	328
I21.9	Acute myocardial infarction, unspecified	893
I22	Subsequent myocardial infarction	255
I22.9	Subsequent myocardial infarction of unspecified site	130
I24	Other acute ischaemic heart diseases	218
I24.8	Other forms of acute ischaemic heart disease	117
I25	Chronic ischaemic heart disease	7678
I25.1	Atherosclerotic heart disease	5654
I25.2	Old myocardial infarction	1202
I25.8	Other forms of chronic ischaemic heart disease	2018
I25.9	Chronic ischaemic heart disease, unspecified	2448
I26	Pulmonary embolism	734
I26.9	Pulmonary embolism without mention of acute cor pulmonale	727
I27	Other pulmonary heart diseases	110
I31	Other diseases of pericardium	218
I31.9	Disease of pericardium, unspecified	124
I34	Nonrheumatic mitral valve disorders	508
I34.0	Mitral (valve) insufficiency	420
I34.1	Mitral (valve) prolapse	143
I35	Nonrheumatic aortic valve disorders	455
I35.0	Aortic (valve) stenosis	268
I35.1	Aortic (valve) insufficiency	163
I42	Cardiomyopathy	289
I42.0	Dilated cardiomyopathy	163
I42.9	Cardiomyopathy, unspecified	103
I44	Atrioventricular and left bundle-branch block	552
I44.0	Atrioventricular block, first degree	107
I44.2	Atrioventricular block, complete	111
I44.7	Left bundle-branch block, unspecified	248
I45	Other conduction disorders	409
I45.1	Other and unspecified right bundle-branch block	237
I46	Cardiac arrest	114
I47	Paroxysmal tachycardia	911
I47.1	Supraventricular tachycardia	669
I47.2	Ventricular tachycardia	215
I48	Atrial fibrillation and flutter	3025
I49	Other cardiac arrhythmias	572
I49.8	Other specified cardiac arrhythmias	116

I49.9	Cardiac arrhythmia, unspecified	212
I50	Heart failure	1081
I50.0	Congestive heart failure	301
I50.1	Left ventricular failure	776
I50.9	Heart failure, unspecified	153
I51	Complications and ill-defined descriptions of heart disease	614
I51.7	Cardiomegaly	417
I51.8	Other ill-defined heart diseases	106
I60	Subarachnoid haemorrhage	272
I60.9	Subarachnoid haemorrhage, unspecified	172
I61	Intracerebral haemorrhage	152
I61.9	Intracerebral haemorrhage, unspecified	103
I63	Cerebral infarction	599
I63.9	Cerebral infarction, unspecified	500
I64	Stroke, not specified as haemorrhage or infarction	336
I65	Occlusion and stenosis of precerebral arteries, not resulting in cerebral infarction	222
I65.2	Occlusion and stenosis of carotid artery	209
I67	Other cerebrovascular diseases	362
I67.9	Cerebrovascular disease, unspecified	150
I69	Sequelae of cerebrovascular disease	287
I69.4	Sequelae of stroke, not specified as haemorrhage or infarction	173
I70	Atherosclerosis	269
I70.2	Atherosclerosis of arteries of the extremities	165
I71	Aortic aneurysm and dissection	204
I71.4	Abdominal aortic aneurysm, without mention of rupture	103
I73	Other peripheral vascular diseases	870
I73.0	Raynaud's syndrome	209
I73.9	Peripheral vascular disease, unspecified	658
I74	Arterial embolism and thrombosis	221
I74.3	Embolism and thrombosis of arteries of the lower extremities	121
I77	Other disorders of arteries and arterioles	354
I77.1	Stricture of artery	193
I78	Diseases of capillaries	138
I78.1	Naevus, nonneoplastic	113

Table S2. List of metabolites and their abbreviations

Metabolites	Description	Units	Group	Sub-group	Type	UKB Field ID	QC Flag Field ID
Total_C	Total Cholesterol	mmol/L	Cholesterol		Composite	23400	23700
non_HDL_C	Total Cholesterol Minus HDL-C	mmol/L	Cholesterol		Composite	23401	23701
Remnant_C	Remnant Cholesterol (Non-HDL, Non-LDL -Cholesterol)	mmol/L	Cholesterol		Composite	23402	23702
VLDL_C	VLDL Cholesterol	mmol/L	Cholesterol		Composite	23403	23703
Clinical_LDL_C	Clinical LDL Cholesterol	mmol/L	Cholesterol		Non-derived	23404	23704
LDL_C	LDL Cholesterol	mmol/L	Cholesterol		Composite	23405	23705
HDL_C	HDL Cholesterol	mmol/L	Cholesterol		Composite	23406	23706
Total_TG	Total Triglycerides	mmol/L	Triglycerides		Composite	23407	23707
VLDL_TG	Triglycerides in VLDL	mmol/L	Triglycerides		Composite	23408	23708
LDL_TG	Triglycerides in LDL	mmol/L	Triglycerides		Composite	23409	23709
HDL_TG	Triglycerides in HDL	mmol/L	Triglycerides		Composite	23410	23710
Total_PL	Total Phospholipids in Lipoprotein Particles	mmol/L	Phospholipids		Composite	23411	23711
VLDL_PL	Phospholipids in VLDL	mmol/L	Phospholipids		Composite	23412	23712
LDL_PL	Phospholipids in LDL	mmol/L	Phospholipids		Composite	23413	23713
HDL_PL	Phospholipids in HDL	mmol/L	Phospholipids		Composite	23414	23714
Total_CE	Total Esterified Cholesterol	mmol/L	Cholesteryl esters		Composite	23415	23715
VLDL_CE	Cholesteryl Esters in VLDL	mmol/L	Cholesteryl esters		Composite	23416	23716
LDL_CE	Cholesteryl Esters in LDL	mmol/L	Cholesteryl esters		Composite	23417	23717
HDL_CE	Cholesteryl Esters in HDL	mmol/L	Cholesteryl esters		Composite	23418	23718
Total_FC	Total Free Cholesterol	mmol/L	Free cholesterol		Composite	23419	23719
VLDL_FC	Free Cholesterol in VLDL	mmol/L	Free cholesterol		Composite	23420	23720
LDL_FC	Free Cholesterol in LDL	mmol/L	Free cholesterol		Composite	23421	23721
HDL_FC	Free Cholesterol in HDL	mmol/L	Free cholesterol		Composite	23422	23722
Total_L	Total Lipids in Lipoprotein Particles	mmol/L	Total lipids		Composite	23423	23723
VLDL_L	Total Lipids in VLDL	mmol/L	Total lipids		Composite	23424	23724
LDL_L	Total Lipids in LDL	mmol/L	Total lipids		Composite	23425	23725
HDL_L	Total Lipids in HDL	mmol/L	Total lipids		Composite	23426	23726

Total_P	Total Concentration of Lipoprotein Particles	mmol/L	Lipoprotein particle concentrations		Composite	23427	23727
VLDL_P	Concentration of VLDL Particles	mmol/L	Lipoprotein particle concentrations		Composite	23428	23728
LDL_P	Concentration of LDL Particles	mmol/L	Lipoprotein particle concentrations		Composite	23429	23729
HDL_P	Concentration of HDL Particles	mmol/L	Lipoprotein particle concentrations		Composite	23430	23730
VLDL_size	Average Diameter for VLDL Particles	nm	Lipoprotein particle sizes		Non-derived	23431	23731
LDL_size	Average Diameter for LDL Particles	nm	Lipoprotein particle sizes		Non-derived	23432	23732
HDL_size	Average Diameter for HDL Particles	nm	Lipoprotein particle sizes		Non-derived	23433	23733
Phosphoglyc	Phosphoglycerides	mmol/L	Other lipids		Non-derived	23434	23734
TG_by_PG	Triglycerides to Phosphoglycerides ratio	ratio	Other lipids		Ratio	23435	23735
Cholines	Total Cholines	mmol/L	Other lipids		Non-derived	23436	23736
Phosphatidylc	Phosphatidylcholines	mmol/L	Other lipids		Non-derived	23437	23737
Sphingomyelins	Sphingomyelins	mmol/L	Other lipids		Non-derived	23438	23738
ApoB	Apolipoprotein B	g/l	Apolipoproteins		Non-derived	23439	23739
ApoA1	Apolipoprotein A1	g/l	Apolipoproteins		Non-derived	23440	23740
ApoB_by_ApoA1	Apolipoprotein B to Apolipoprotein A1 ratio	ratio	Apolipoproteins		Ratio	23441	23741
Total_FA	Total Fatty Acids	mmol/L	Fatty acids		Composite	23442	23742
Unsaturation	Degree of Unsaturation	degree	Fatty acids		Non-derived	23443	23743
Omega_3	Omega-3 Fatty Acids	mmol/L	Fatty acids		Non-derived	23444	23744
Omega_6	Omega-6 Fatty Acids	mmol/L	Fatty acids		Non-derived	23445	23745
PUFA	Polyunsaturated Fatty Acids	mmol/L	Fatty acids		Composite	23446	23746
MUFA	Monounsaturated Fatty Acids	mmol/L	Fatty acids		Non-derived	23447	23747
SFA	Saturated Fatty Acids	mmol/L	Fatty acids		Non-derived	23448	23748
LA	Linoleic Acid	mmol/L	Fatty acids		Non-derived	23449	23749
DHA	Docosahexaenoic Acid	mmol/L	Fatty acids		Non-derived	23450	23750
Omega_3_pct	Omega-3 Fatty Acids to Total Fatty Acids percentage	%	Fatty acids	Fatty acid ratios	Percentage	23451	23751
Omega_6_pct	Omega-6 Fatty Acids to Total Fatty Acids percentage	%	Fatty acids	Fatty acid ratios	Percentage	23452	23752
PUFA_pct	Polyunsaturated Fatty Acids to Total Fatty Acids percentage	%	Fatty acids	Fatty acid ratios	Percentage	23453	23753

MUFA_pct	Monounsaturated Fatty Acids to Total Fatty Acids percentage	%	Fatty acids	Fatty acid ratios	Percentage	23454	23754
SFA_pct	Saturated Fatty Acids to Total Fatty Acids percentage	%	Fatty acids	Fatty acid ratios	Percentage	23455	23755
LA_pct	Linoleic Acid to Total Fatty Acids percentage	%	Fatty acids	Fatty acid ratios	Percentage	23456	23756
DHA_pct	Docosahexaenoic Acid to Total Fatty Acids percentage	%	Fatty acids	Fatty acid ratios	Percentage	23457	23757
PUFA_by_MUFA	Polyunsaturated Fatty Acids to Monounsaturated Fatty Acids ratio	ratio	Fatty acids	Fatty acid ratios	Ratio	23458	23758
Omega_6_by_Omega_3	Omega-6 Fatty Acids to Omega-3 Fatty Acids ratio	ratio	Fatty acids	Fatty acid ratios	Ratio	23459	23759
Ala	Alanine	mmol/L	Amino acids		Non-derived	23460	23760
Gln	Glutamine	mmol/L	Amino acids		Non-derived	23461	23761
Gly	Glycine	mmol/L	Amino acids		Non-derived	23462	23762
His	Histidine	mmol/L	Amino acids		Non-derived	23463	23763
Total_BCAA	Total Concentration of Branched-Chain Amino Acids (Leucine + Isoleucine + Valine)	mmol/L	Amino acids	Branched-chain amino acids	Composite	23464	23764
Ile	Isoleucine	mmol/L	Amino acids	Branched-chain amino acids	Non-derived	23465	23765
Leu	Leucine	mmol/L	Amino acids	Branched-chain amino acids	Non-derived	23466	23766
Val	Valine	mmol/L	Amino acids	Branched-chain amino acids	Non-derived	23467	23767
Phe	Phenylalanine	mmol/L	Amino acids	Aromatic amino acids	Non-derived	23468	23768
Tyr	Tyrosine	mmol/L	Amino acids	Aromatic amino acids	Non-derived	23469	23769
Glucose	Glucose	mmol/L	Glycolysis related metabolites		Non-derived	23470	23770
Lactate	Lactate	mmol/L	Glycolysis related metabolites		Non-derived	23471	23771
Pyruvate	Pyruvate	mmol/L	Glycolysis related metabolites		Non-derived	23472	23772
Citrate	Citrate	mmol/L	Glycolysis related metabolites		Non-derived	23473	23773
bOHbutyrate	3-Hydroxybutyrate	mmol/L	Ketone bodies		Non-derived	23474	23774
Acetate	Acetate	mmol/L	Ketone bodies		Non-derived	23475	23775
Acetoacetate	Acetoacetate	mmol/L	Ketone bodies		Non-derived	23476	23776
Acetone	Acetone	mmol/L	Ketone bodies		Non-derived	23477	23777

Creatinine	Creatinine	mmol/L	Fluid balance		Non-derived	23478	23778
Albumin	Albumin	g/l	Fluid balance		Non-derived	23479	23779
GlycA	Glycoprotein Acetyls	mmol/L	Inflammation		Non-derived	23480	23780
XXL_VLDL_P	Concentration of Chylomicrons and Extremely Large VLDL Particles	mmol/L	Lipoprotein subclasses	Chylomicrons and extremely large VLDL (particle diameters from 75 nm upwards)	Non-derived	23481	23781
XXL_VLDL_L	Total Lipids in Chylomicrons and Extremely Large VLDL	mmol/L	Lipoprotein subclasses	Chylomicrons and extremely large VLDL (particle diameters from 75 nm upwards)	Composite	23482	23782
XXL_VLDL_PL	Phospholipids in Chylomicrons and Extremely Large VLDL	mmol/L	Lipoprotein subclasses	Chylomicrons and extremely large VLDL (particle diameters from 75 nm upwards)	Non-derived	23483	23783
XXL_VLDL_C	Cholesterol in Chylomicrons and Extremely Large VLDL	mmol/L	Lipoprotein subclasses	Chylomicrons and extremely large VLDL (particle diameters from 75 nm upwards)	Composite	23484	23784
XXL_VLDL_CE	Cholesteryl Esters in Chylomicrons and Extremely Large VLDL	mmol/L	Lipoprotein subclasses	Chylomicrons and extremely large VLDL (particle diameters from 75 nm upwards)	Non-derived	23485	23785
XXL_VLDL_FC	Free Cholesterol in Chylomicrons and Extremely Large VLDL	mmol/L	Lipoprotein subclasses	Chylomicrons and extremely large VLDL (particle diameters from 75 nm upwards)	Non-derived	23486	23786
XXL_VLDL_TG	Triglycerides in Chylomicrons and Extremely Large VLDL	mmol/L	Lipoprotein subclasses	Chylomicrons and extremely large VLDL (particle diameters from 75 nm upwards)	Non-derived	23487	23787
XL_VLDL_P	Concentration of Very Large VLDL Particles	mmol/L	Lipoprotein subclasses	Very large VLDL (average diameter 64 nm)	Non-derived	23488	23788
XL_VLDL_L	Total Lipids in Very Large VLDL	mmol/L	Lipoprotein subclasses	Very large VLDL (average diameter 64 nm)	Composite	23489	23789
XL_VLDL_PL	Phospholipids in Very Large VLDL	mmol/L	Lipoprotein subclasses	Very large VLDL (average diameter 64 nm)	Non-derived	23490	23790
XL_VLDL_C	Cholesterol in Very Large VLDL	mmol/L	Lipoprotein subclasses	Very large VLDL (average diameter 64 nm)	Composite	23491	23791
XL_VLDL_CE	Cholesteryl Esters in Very Large VLDL	mmol/L	Lipoprotein subclasses	Very large VLDL (average diameter 64 nm)	Non-derived	23492	23792

XL_VLDL_FC	Free Cholesterol in Very Large VLDL	mmol/L	Lipoprotein subclasses	Very large VLDL (average diameter 64 nm)	Non-derived	23493	23793
XL_VLDL_TG	Triglycerides in Very Large VLDL	mmol/L	Lipoprotein subclasses	Very large VLDL (average diameter 64 nm)	Non-derived	23494	23794
L_VLDL_P	Concentration of Large VLDL Particles	mmol/L	Lipoprotein subclasses	Large VLDL (average diameter 53.6 nm)	Non-derived	23495	23795
L_VLDL_L	Total Lipids in Large VLDL	mmol/L	Lipoprotein subclasses	Large VLDL (average diameter 53.6 nm)	Composite	23496	23796
L_VLDL_PL	Phospholipids in Large VLDL	mmol/L	Lipoprotein subclasses	Large VLDL (average diameter 53.6 nm)	Non-derived	23497	23797
L_VLDL_C	Cholesterol in Large VLDL	mmol/L	Lipoprotein subclasses	Large VLDL (average diameter 53.6 nm)	Composite	23498	23798
L_VLDL_CE	Cholesteryl Esters in Large VLDL	mmol/L	Lipoprotein subclasses	Large VLDL (average diameter 53.6 nm)	Non-derived	23499	23799
L_VLDL_FC	Free Cholesterol in Large VLDL	mmol/L	Lipoprotein subclasses	Large VLDL (average diameter 53.6 nm)	Non-derived	23500	23800
L_VLDL_TG	Triglycerides in Large VLDL	mmol/L	Lipoprotein subclasses	Large VLDL (average diameter 53.6 nm)	Non-derived	23501	23801
M_VLDL_P	Concentration of Medium VLDL Particles	mmol/L	Lipoprotein subclasses	Medium VLDL (average diameter 44.5 nm)	Non-derived	23502	23802
M_VLDL_L	Total Lipids in Medium VLDL	mmol/L	Lipoprotein subclasses	Medium VLDL (average diameter 44.5 nm)	Composite	23503	23803
M_VLDL_PL	Phospholipids in Medium VLDL	mmol/L	Lipoprotein subclasses	Medium VLDL (average diameter 44.5 nm)	Non-derived	23504	23804
M_VLDL_C	Cholesterol in Medium VLDL	mmol/L	Lipoprotein subclasses	Medium VLDL (average diameter 44.5 nm)	Composite	23505	23805
M_VLDL_CE	Cholesteryl Esters in Medium VLDL	mmol/L	Lipoprotein subclasses	Medium VLDL (average diameter 44.5 nm)	Non-derived	23506	23806
M_VLDL_FC	Free Cholesterol in Medium VLDL	mmol/L	Lipoprotein subclasses	Medium VLDL (average diameter 44.5 nm)	Non-derived	23507	23807
M_VLDL_TG	Triglycerides in Medium VLDL	mmol/L	Lipoprotein subclasses	Medium VLDL (average diameter 44.5 nm)	Non-derived	23508	23808
S_VLDL_P	Concentration of Small VLDL Particles	mmol/L	Lipoprotein subclasses	Small VLDL (average diameter 36.8 nm)	Non-derived	23509	23809
S_VLDL_L	Total Lipids in Small VLDL	mmol/L	Lipoprotein subclasses	Small VLDL (average diameter 36.8 nm)	Composite	23510	23810
S_VLDL_PL	Phospholipids in Small VLDL	mmol/L	Lipoprotein subclasses	Small VLDL (average diameter 36.8 nm)	Non-derived	23511	23811
S_VLDL_C	Cholesterol in Small VLDL	mmol/L	Lipoprotein subclasses	Small VLDL (average diameter 36.8 nm)	Composite	23512	23812
S_VLDL_CE	Cholesteryl Esters in Small VLDL	mmol/L	Lipoprotein subclasses	Small VLDL (average diameter 36.8 nm)	Non-derived	23513	23813

S_VLDL_FC	Free Cholesterol in Small VLDL	mmol/L	Lipoprotein subclasses	Small VLDL (average diameter 36.8 nm)	Non-derived	23514	23814
S_VLDL_TG	Triglycerides in Small VLDL	mmol/L	Lipoprotein subclasses	Small VLDL (average diameter 36.8 nm)	Non-derived	23515	23815
XS_VLDL_P	Concentration of Very Small VLDL Particles	mmol/L	Lipoprotein subclasses	Very small VLDL (average diameter 31.3 nm)	Non-derived	23516	23816
XS_VLDL_L	Total Lipids in Very Small VLDL	mmol/L	Lipoprotein subclasses	Very small VLDL (average diameter 31.3 nm)	Composite	23517	23817
XS_VLDL_PL	Phospholipids in Very Small VLDL	mmol/L	Lipoprotein subclasses	Very small VLDL (average diameter 31.3 nm)	Non-derived	23518	23818
XS_VLDL_C	Cholesterol in Very Small VLDL	mmol/L	Lipoprotein subclasses	Very small VLDL (average diameter 31.3 nm)	Composite	23519	23819
XS_VLDL_CE	Cholesteryl Esters in Very Small VLDL	mmol/L	Lipoprotein subclasses	Very small VLDL (average diameter 31.3 nm)	Non-derived	23520	23820
XS_VLDL_FC	Free Cholesterol in Very Small VLDL	mmol/L	Lipoprotein subclasses	Very small VLDL (average diameter 31.3 nm)	Non-derived	23521	23821
XS_VLDL_TG	Triglycerides in Very Small VLDL	mmol/L	Lipoprotein subclasses	Very small VLDL (average diameter 31.3 nm)	Non-derived	23522	23822
IDL_P	Concentration of IDL Particles	mmol/L	Lipoprotein subclasses	IDL (average diameter 28.6 nm)	Non-derived	23523	23823
IDL_L	Total Lipids in IDL	mmol/L	Lipoprotein subclasses	IDL (average diameter 28.6 nm)	Composite	23524	23824
IDL_PL	Phospholipids in IDL	mmol/L	Lipoprotein subclasses	IDL (average diameter 28.6 nm)	Non-derived	23525	23825
IDL_C	Cholesterol in IDL	mmol/L	Lipoprotein subclasses	IDL (average diameter 28.6 nm)	Composite	23526	23826
IDL_CE	Cholesteryl Esters in IDL	mmol/L	Lipoprotein subclasses	IDL (average diameter 28.6 nm)	Non-derived	23527	23827
IDL_FC	Free Cholesterol in IDL	mmol/L	Lipoprotein subclasses	IDL (average diameter 28.6 nm)	Non-derived	23528	23828
IDL_TG	Triglycerides in IDL	mmol/L	Lipoprotein subclasses	IDL (average diameter 28.6 nm)	Non-derived	23529	23829
L_LDL_P	Concentration of Large LDL Particles	mmol/L	Lipoprotein subclasses	Large LDL (average diameter 25.5 nm)	Non-derived	23530	23830
L_LDL_L	Total Lipids in Large LDL	mmol/L	Lipoprotein subclasses	Large LDL (average diameter 25.5 nm)	Composite	23531	23831
L_LDL_PL	Phospholipids in Large LDL	mmol/L	Lipoprotein subclasses	Large LDL (average diameter 25.5 nm)	Non-derived	23532	23832
L_LDL_C	Cholesterol in Large LDL	mmol/L	Lipoprotein subclasses	Large LDL (average diameter 25.5 nm)	Composite	23533	23833
L_LDL_CE	Cholesteryl Esters in Large LDL	mmol/L	Lipoprotein subclasses	Large LDL (average diameter 25.5 nm)	Non-derived	23534	23834

L_LDL_FC	Free Cholesterol in Large LDL	mmol/L	Lipoprotein subclasses	Large LDL (average diameter 25.5 nm)	Non-derived	23535	23835
L_LDL_TG	Triglycerides in Large LDL	mmol/L	Lipoprotein subclasses	Large LDL (average diameter 25.5 nm)	Non-derived	23536	23836
M_LDL_P	Concentration of Medium LDL Particles	mmol/L	Lipoprotein subclasses	Medium LDL (average diameter 23 nm)	Non-derived	23537	23837
M_LDL_L	Total Lipids in Medium LDL	mmol/L	Lipoprotein subclasses	Medium LDL (average diameter 23 nm)	Composite	23538	23838
M_LDL_PL	Phospholipids in Medium LDL	mmol/L	Lipoprotein subclasses	Medium LDL (average diameter 23 nm)	Non-derived	23539	23839
M_LDL_C	Cholesterol in Medium LDL	mmol/L	Lipoprotein subclasses	Medium LDL (average diameter 23 nm)	Composite	23540	23840
M_LDL_CE	Cholesteryl Esters in Medium LDL	mmol/L	Lipoprotein subclasses	Medium LDL (average diameter 23 nm)	Non-derived	23541	23841
M_LDL_FC	Free Cholesterol in Medium LDL	mmol/L	Lipoprotein subclasses	Medium LDL (average diameter 23 nm)	Non-derived	23542	23842
M_LDL_TG	Triglycerides in Medium LDL	mmol/L	Lipoprotein subclasses	Medium LDL (average diameter 23 nm)	Non-derived	23543	23843
S_LDL_P	Concentration of Small LDL Particles	mmol/L	Lipoprotein subclasses	Small LDL (average diameter 18.7 nm)	Non-derived	23544	23844
S_LDL_L	Total Lipids in Small LDL	mmol/L	Lipoprotein subclasses	Small LDL (average diameter 18.7 nm)	Composite	23545	23845
S_LDL_PL	Phospholipids in Small LDL	mmol/L	Lipoprotein subclasses	Small LDL (average diameter 18.7 nm)	Non-derived	23546	23846
S_LDL_C	Cholesterol in Small LDL	mmol/L	Lipoprotein subclasses	Small LDL (average diameter 18.7 nm)	Composite	23547	23847
S_LDL_CE	Cholesteryl Esters in Small LDL	mmol/L	Lipoprotein subclasses	Small LDL (average diameter 18.7 nm)	Non-derived	23548	23848
S_LDL_FC	Free Cholesterol in Small LDL	mmol/L	Lipoprotein subclasses	Small LDL (average diameter 18.7 nm)	Non-derived	23549	23849
S_LDL_TG	Triglycerides in Small LDL	mmol/L	Lipoprotein subclasses	Small LDL (average diameter 18.7 nm)	Non-derived	23550	23850
XL_HDL_P	Concentration of Very Large HDL Particles	mmol/L	Lipoprotein subclasses	Very large HDL (average diameter 14.3 nm)	Non-derived	23551	23851
XL_HDL_L	Total Lipids in Very Large HDL	mmol/L	Lipoprotein subclasses	Very large HDL (average diameter 14.3 nm)	Composite	23552	23852
XL_HDL_PL	Phospholipids in Very Large HDL	mmol/L	Lipoprotein subclasses	Very large HDL (average diameter 14.3 nm)	Non-derived	23553	23853
XL_HDL_C	Cholesterol in Very Large HDL	mmol/L	Lipoprotein subclasses	Very large HDL (average diameter 14.3 nm)	Composite	23554	23854
XL_HDL_CE	Cholesteryl Esters in Very Large HDL	mmol/L	Lipoprotein subclasses	Very large HDL (average diameter 14.3 nm)	Non-derived	23555	23855

XL_HDL_FC	Free Cholesterol in Very Large HDL	mmol/L	Lipoprotein subclasses	Very large HDL (average diameter 14.3 nm)	Non-derived	23556	23856
XL_HDL_TG	Triglycerides in Very Large HDL	mmol/L	Lipoprotein subclasses	Very large HDL (average diameter 14.3 nm)	Non-derived	23557	23857
L_HDL_P	Concentration of Large HDL Particles	mmol/L	Lipoprotein subclasses	Large HDL (average diameter 12.1 nm)	Non-derived	23558	23858
L_HDL_L	Total Lipids in Large HDL	mmol/L	Lipoprotein subclasses	Large HDL (average diameter 12.1 nm)	Composite	23559	23859
L_HDL_PL	Phospholipids in Large HDL	mmol/L	Lipoprotein subclasses	Large HDL (average diameter 12.1 nm)	Non-derived	23560	23860
L_HDL_C	Cholesterol in Large HDL	mmol/L	Lipoprotein subclasses	Large HDL (average diameter 12.1 nm)	Composite	23561	23861
L_HDL_CE	Cholesteryl Esters in Large HDL	mmol/L	Lipoprotein subclasses	Large HDL (average diameter 12.1 nm)	Non-derived	23562	23862
L_HDL_FC	Free Cholesterol in Large HDL	mmol/L	Lipoprotein subclasses	Large HDL (average diameter 12.1 nm)	Non-derived	23563	23863
L_HDL_TG	Triglycerides in Large HDL	mmol/L	Lipoprotein subclasses	Large HDL (average diameter 12.1 nm)	Non-derived	23564	23864
M_HDL_P	Concentration of Medium HDL Particles	mmol/L	Lipoprotein subclasses	Medium HDL (average diameter 10.9 nm)	Non-derived	23565	23865
M_HDL_L	Total Lipids in Medium HDL	mmol/L	Lipoprotein subclasses	Medium HDL (average diameter 10.9 nm)	Composite	23566	23866
M_HDL_PL	Phospholipids in Medium HDL	mmol/L	Lipoprotein subclasses	Medium HDL (average diameter 10.9 nm)	Non-derived	23567	23867
M_HDL_C	Cholesterol in Medium HDL	mmol/L	Lipoprotein subclasses	Medium HDL (average diameter 10.9 nm)	Composite	23568	23868
M_HDL_CE	Cholesteryl Esters in Medium HDL	mmol/L	Lipoprotein subclasses	Medium HDL (average diameter 10.9 nm)	Non-derived	23569	23869
M_HDL_FC	Free Cholesterol in Medium HDL	mmol/L	Lipoprotein subclasses	Medium HDL (average diameter 10.9 nm)	Non-derived	23570	23870
M_HDL_TG	Triglycerides in Medium HDL	mmol/L	Lipoprotein subclasses	Medium HDL (average diameter 10.9 nm)	Non-derived	23571	23871
S_HDL_P	Concentration of Small HDL Particles	mmol/L	Lipoprotein subclasses	Small HDL (average diameter 8.7 nm)	Non-derived	23572	23872
S_HDL_L	Total Lipids in Small HDL	mmol/L	Lipoprotein subclasses	Small HDL (average diameter 8.7 nm)	Composite	23573	23873
S_HDL_PL	Phospholipids in Small HDL	mmol/L	Lipoprotein subclasses	Small HDL (average diameter 8.7 nm)	Non-derived	23574	23874
S_HDL_C	Cholesterol in Small HDL	mmol/L	Lipoprotein subclasses	Small HDL (average diameter 8.7 nm)	Composite	23575	23875
S_HDL_CE	Cholesteryl Esters in Small HDL	mmol/L	Lipoprotein subclasses	Small HDL (average diameter 8.7 nm)	Non-derived	23576	23876

S_HDL_FC	Free Cholesterol in Small HDL	mmol/L	Lipoprotein subclasses	Small HDL (average diameter 8.7 nm)	Non-derived	23577	23877
S_HDL_TG	Triglycerides in Small HDL	mmol/L	Lipoprotein subclasses	Small HDL (average diameter 8.7 nm)	Non-derived	23578	23878
XXL_VLDL_PL_pct	Phospholipids to Total Lipids in Chylomicrons and Extremely Large VLDL percentage	%	Relative lipoprotein lipid concentrations	Chylomicrons and extremely large VLDL ratios	Percentage	23579	23879
XXL_VLDL_C_pct	Cholesterol to Total Lipids in Chylomicrons and Extremely Large VLDL percentage	%	Relative lipoprotein lipid concentrations	Chylomicrons and extremely large VLDL ratios	Percentage	23580	23880
XXL_VLDL_CE_pct	Cholesteryl Esters to Total Lipids in Chylomicrons and Extremely Large VLDL percentage	%	Relative lipoprotein lipid concentrations	Chylomicrons and extremely large VLDL ratios	Percentage	23581	23881
XXL_VLDL_FC_pct	Free Cholesterol to Total Lipids in Chylomicrons and Extremely Large VLDL percentage	%	Relative lipoprotein lipid concentrations	Chylomicrons and extremely large VLDL ratios	Percentage	23582	23882
XXL_VLDL_TG_pct	Triglycerides to Total Lipids in Chylomicrons and Extremely Large VLDL percentage	%	Relative lipoprotein lipid concentrations	Chylomicrons and extremely large VLDL ratios	Percentage	23583	23883
XL_VLDL_PL_pct	Phospholipids to Total Lipids in Very Large VLDL percentage	%	Relative lipoprotein lipid concentrations	Very large VLDL ratios	Percentage	23584	23884
XL_VLDL_C_pct	Cholesterol to Total Lipids in Very Large VLDL percentage	%	Relative lipoprotein lipid concentrations	Very large VLDL ratios	Percentage	23585	23885
XL_VLDL_CE_pct	Cholesteryl Esters to Total Lipids in Very Large VLDL percentage	%	Relative lipoprotein lipid concentrations	Very large VLDL ratios	Percentage	23586	23886
XL_VLDL_FC_pct	Free Cholesterol to Total Lipids in Very Large VLDL percentage	%	Relative lipoprotein lipid concentrations	Very large VLDL ratios	Percentage	23587	23887
XL_VLDL_TG_pct	Triglycerides to Total Lipids in Very Large VLDL percentage	%	Relative lipoprotein lipid concentrations	Very large VLDL ratios	Percentage	23588	23888
L_VLDL_PL_pct	Phospholipids to Total Lipids in Large VLDL percentage	%	Relative lipoprotein lipid concentrations	Large VLDL ratios	Percentage	23589	23889
L_VLDL_C_pct	Cholesterol to Total Lipids in Large VLDL percentage	%	Relative lipoprotein lipid concentrations	Large VLDL ratios	Percentage	23590	23890
L_VLDL_CE_pct	Cholesteryl Esters to Total Lipids in Large VLDL percentage	%	Relative lipoprotein lipid concentrations	Large VLDL ratios	Percentage	23591	23891
L_VLDL_FC_pct	Free Cholesterol to Total Lipids in Large VLDL percentage	%	Relative lipoprotein lipid concentrations	Large VLDL ratios	Percentage	23592	23892
L_VLDL_TG_pct	Triglycerides to Total Lipids in Large VLDL percentage	%	Relative lipoprotein lipid concentrations	Large VLDL ratios	Percentage	23593	23893
M_VLDL_PL_pct	Phospholipids to Total Lipids in Medium VLDL percentage	%	Relative lipoprotein lipid concentrations	Medium VLDL ratios	Percentage	23594	23894
M_VLDL_C_pct	Cholesterol to Total Lipids in Medium VLDL percentage	%	Relative lipoprotein lipid concentrations	Medium VLDL ratios	Percentage	23595	23895

M_VLDL_CE_pct	Cholesteryl Esters to Total Lipids in Medium VLDL percentage	%	Relative lipoprotein lipid concentrations	Medium VLDL ratios	Percentage	23596	23896
M_VLDL_FC_pct	Free Cholesterol to Total Lipids in Medium VLDL percentage	%	Relative lipoprotein lipid concentrations	Medium VLDL ratios	Percentage	23597	23897
M_VLDL_TG_pct	Triglycerides to Total Lipids in Medium VLDL percentage	%	Relative lipoprotein lipid concentrations	Medium VLDL ratios	Percentage	23598	23898
S_VLDL_PL_pct	Phospholipids to Total Lipids in Small VLDL percentage	%	Relative lipoprotein lipid concentrations	Small VLDL ratios	Percentage	23599	23899
S_VLDL_C_pct	Cholesterol to Total Lipids in Small VLDL percentage	%	Relative lipoprotein lipid concentrations	Small VLDL ratios	Percentage	23600	23900
S_VLDL_CE_pct	Cholesteryl Esters to Total Lipids in Small VLDL percentage	%	Relative lipoprotein lipid concentrations	Small VLDL ratios	Percentage	23601	23901
S_VLDL_FC_pct	Free Cholesterol to Total Lipids in Small VLDL percentage	%	Relative lipoprotein lipid concentrations	Small VLDL ratios	Percentage	23602	23902
S_VLDL_TG_pct	Triglycerides to Total Lipids in Small VLDL percentage	%	Relative lipoprotein lipid concentrations	Small VLDL ratios	Percentage	23603	23903
XS_VLDL_PL_pct	Phospholipids to Total Lipids in Very Small VLDL percentage	%	Relative lipoprotein lipid concentrations	Very small VLDL ratios	Percentage	23604	23904
XS_VLDL_C_pct	Cholesterol to Total Lipids in Very Small VLDL percentage	%	Relative lipoprotein lipid concentrations	Very small VLDL ratios	Percentage	23605	23905
XS_VLDL_CE_pct	Cholesteryl Esters to Total Lipids in Very Small VLDL percentage	%	Relative lipoprotein lipid concentrations	Very small VLDL ratios	Percentage	23606	23906
XS_VLDL_FC_pct	Free Cholesterol to Total Lipids in Very Small VLDL percentage	%	Relative lipoprotein lipid concentrations	Very small VLDL ratios	Percentage	23607	23907
XS_VLDL_TG_pct	Triglycerides to Total Lipids in Very Small VLDL percentage	%	Relative lipoprotein lipid concentrations	Very small VLDL ratios	Percentage	23608	23908
IDL_PL_pct	Phospholipids to Total Lipids in IDL percentage	%	Relative lipoprotein lipid concentrations	IDL ratios	Percentage	23609	23909
IDL_C_pct	Cholesterol to Total Lipids in IDL percentage	%	Relative lipoprotein lipid concentrations	IDL ratios	Percentage	23610	23910
IDL_CE_pct	Cholesteryl Esters to Total Lipids in IDL percentage	%	Relative lipoprotein lipid concentrations	IDL ratios	Percentage	23611	23911
IDL_FC_pct	Free Cholesterol to Total Lipids in IDL percentage	%	Relative lipoprotein lipid concentrations	IDL ratios	Percentage	23612	23912
IDL_TG_pct	Triglycerides to Total Lipids in IDL percentage	%	Relative lipoprotein lipid concentrations	IDL ratios	Percentage	23613	23913
L_LDL_PL_pct	Phospholipids to Total Lipids in Large LDL percentage	%	Relative lipoprotein lipid concentrations	Large LDL ratios	Percentage	23614	23914
L_LDL_C_pct	Cholesterol to Total Lipids in Large LDL percentage	%	Relative lipoprotein lipid concentrations	Large LDL ratios	Percentage	23615	23915
L_LDL_CE_pct	Cholesteryl Esters to Total Lipids in Large LDL percentage	%	Relative lipoprotein lipid concentrations	Large LDL ratios	Percentage	23616	23916

L_LDL_FC_pct	Free Cholesterol to Total Lipids in Large LDL percentage	%	Relative lipoprotein lipid concentrations	Large LDL ratios	Percentage	23617	23917
L_LDL_TG_pct	Triglycerides to Total Lipids in Large LDL percentage	%	Relative lipoprotein lipid concentrations	Large LDL ratios	Percentage	23618	23918
M_LDL_PL_pct	Phospholipids to Total Lipids in Medium LDL percentage	%	Relative lipoprotein lipid concentrations	Medium LDL ratios	Percentage	23619	23919
M_LDL_C_pct	Cholesterol to Total Lipids in Medium LDL percentage	%	Relative lipoprotein lipid concentrations	Medium LDL ratios	Percentage	23620	23920
M_LDL_CE_pct	Cholesteryl Esters to Total Lipids in Medium LDL percentage	%	Relative lipoprotein lipid concentrations	Medium LDL ratios	Percentage	23621	23921
M_LDL_FC_pct	Free Cholesterol to Total Lipids in Medium LDL percentage	%	Relative lipoprotein lipid concentrations	Medium LDL ratios	Percentage	23622	23922
M_LDL_TG_pct	Triglycerides to Total Lipids in Medium LDL percentage	%	Relative lipoprotein lipid concentrations	Medium LDL ratios	Percentage	23623	23923
S_LDL_PL_pct	Phospholipids to Total Lipids in Small LDL percentage	%	Relative lipoprotein lipid concentrations	Small LDL ratios	Percentage	23624	23924
S_LDL_C_pct	Cholesterol to Total Lipids in Small LDL percentage	%	Relative lipoprotein lipid concentrations	Small LDL ratios	Percentage	23625	23925
S_LDL_CE_pct	Cholesteryl Esters to Total Lipids in Small LDL percentage	%	Relative lipoprotein lipid concentrations	Small LDL ratios	Percentage	23626	23926
S_LDL_FC_pct	Free Cholesterol to Total Lipids in Small LDL percentage	%	Relative lipoprotein lipid concentrations	Small LDL ratios	Percentage	23627	23927
S_LDL_TG_pct	Triglycerides to Total Lipids in Small LDL percentage	%	Relative lipoprotein lipid concentrations	Small LDL ratios	Percentage	23628	23928
XL_HDL_PL_pct	Phospholipids to Total Lipids in Very Large HDL percentage	%	Relative lipoprotein lipid concentrations	Very large HDL ratios	Percentage	23629	23929
XL_HDL_C_pct	Cholesterol to Total Lipids in Very Large HDL percentage	%	Relative lipoprotein lipid concentrations	Very large HDL ratios	Percentage	23630	23930
XL_HDL_CE_pct	Cholesteryl Esters to Total Lipids in Very Large HDL percentage	%	Relative lipoprotein lipid concentrations	Very large HDL ratios	Percentage	23631	23931
XL_HDL_FC_pct	Free Cholesterol to Total Lipids in Very Large HDL percentage	%	Relative lipoprotein lipid concentrations	Very large HDL ratios	Percentage	23632	23932
XL_HDL_TG_pct	Triglycerides to Total Lipids in Very Large HDL percentage	%	Relative lipoprotein lipid concentrations	Very large HDL ratios	Percentage	23633	23933
L_HDL_PL_pct	Phospholipids to Total Lipids in Large HDL percentage	%	Relative lipoprotein lipid concentrations	Large HDL ratios	Percentage	23634	23934
L_HDL_C_pct	Cholesterol to Total Lipids in Large HDL percentage	%	Relative lipoprotein lipid concentrations	Large HDL ratios	Percentage	23635	23935
L_HDL_CE_pct	Cholesteryl Esters to Total Lipids in Large HDL percentage	%	Relative lipoprotein lipid concentrations	Large HDL ratios	Percentage	23636	23936
L_HDL_FC_pct	Free Cholesterol to Total Lipids in Large HDL percentage	%	Relative lipoprotein lipid concentrations	Large HDL ratios	Percentage	23637	23937

L_HDL_TG_pct	Triglycerides to Total Lipids in Large HDL percentage	%	Relative lipoprotein lipid concentrations	Large HDL ratios	Percentage	23638	23938
M_HDL_PL_pct	Phospholipids to Total Lipids in Medium HDL percentage	%	Relative lipoprotein lipid concentrations	Medium HDL ratios	Percentage	23639	23939
M_HDL_C_pct	Cholesterol to Total Lipids in Medium HDL percentage	%	Relative lipoprotein lipid concentrations	Medium HDL ratios	Percentage	23640	23940
M_HDL_CE_pct	Cholesteryl Esters to Total Lipids in Medium HDL percentage	%	Relative lipoprotein lipid concentrations	Medium HDL ratios	Percentage	23641	23941
M_HDL_FC_pct	Free Cholesterol to Total Lipids in Medium HDL percentage	%	Relative lipoprotein lipid concentrations	Medium HDL ratios	Percentage	23642	23942
M_HDL_TG_pct	Triglycerides to Total Lipids in Medium HDL percentage	%	Relative lipoprotein lipid concentrations	Medium HDL ratios	Percentage	23643	23943
S_HDL_PL_pct	Phospholipids to Total Lipids in Small HDL percentage	%	Relative lipoprotein lipid concentrations	Small HDL ratios	Percentage	23644	23944
S_HDL_C_pct	Cholesterol to Total Lipids in Small HDL percentage	%	Relative lipoprotein lipid concentrations	Small HDL ratios	Percentage	23645	23945
S_HDL_CE_pct	Cholesteryl Esters to Total Lipids in Small HDL percentage	%	Relative lipoprotein lipid concentrations	Small HDL ratios	Percentage	23646	23946
S_HDL_FC_pct	Free Cholesterol to Total Lipids in Small HDL percentage	%	Relative lipoprotein lipid concentrations	Small HDL ratios	Percentage	23647	23947
S_HDL_TG_pct	Triglycerides to Total Lipids in Small HDL percentage	%	Relative lipoprotein lipid concentrations	Small HDL ratios	Percentage	23648	23948
HDL_C_pct	Cholesterol to Total Lipids in HDL percentage	%	Relative lipid concentrations		Percentage		
HDL_CE_pct	Cholesteryl Esters to Total Lipids in HDL percentage	%	Relative lipid concentrations		Percentage		
HDL_CE_pct_C	Cholesteryl Esters to Cholesterol in HDL percentage	%	Relative cholesterol concentrations		Percentage		
HDL_FC_by_CE	Free Cholesterol to Cholesteryl Esters in HDL percentage	ratio	Relative lipid concentrations		Ratio		
HDL_FC_pct	Free Cholesterol to Total Lipids in HDL percentage	%	Relative lipid concentrations		Percentage		
HDL_FC_pct_C	Free Cholesterol to Cholesterol in HDL percentage	%	Relative cholesterol concentrations		Percentage		
HDL_PL_pct	Phospholipids to Total Lipids in HDL percentage	%	Relative lipid concentrations		Percentage		
HDL_TG_pct	Triglycerides to Total Lipids in HDL percentage	%	Relative lipid concentrations		Percentage		
IDL_CE_pct_C	Cholesteryl Esters to Cholesterol in IDL percentage	%	Relative lipoprotein cholesterol concentrations	IDL ratios	Percentage		
IDL_FC_by_CE	Free Cholesterol to Cholesteryl Esters in IDL percentage	ratio	Relative lipoprotein lipid concentrations	IDL ratios	Ratio		

IDL_FC_pct_C	Free Cholesterol to Cholesterol in IDL percentage	%	Relative lipoprotein cholesterol concentrations	IDL ratios	Percentage
L_HDL_CE_pct_C	Cholesteryl Esters to Cholesterol in Large HDL percentage	%	Relative lipoprotein cholesterol concentrations	Large HDL ratios	Percentage
L_HDL_FC_by_CE	Free Cholesterol to Cholesteryl Esters in Large HDL ratio	ratio	Relative lipoprotein lipid concentrations	Large HDL ratios	Ratio
L_HDL_FC_pct_C	Free Cholesterol to Cholesterol in Large HDL percentage	%	Relative lipoprotein cholesterol concentrations	Large HDL ratios	Percentage
L_LDL_CE_pct_C	Cholesteryl Esters to Cholesterol in Large LDL percentage	%	Relative lipoprotein cholesterol concentrations	Large LDL ratios	Percentage
L_LDL_FC_by_CE	Free Cholesterol to Cholesteryl Esters in Large LDL ratio	ratio	Relative lipoprotein lipid concentrations	Large LDL ratios	Ratio
L_LDL_FC_pct_C	Free Cholesterol to Cholesterol in Large LDL percentage	%	Relative lipoprotein cholesterol concentrations	Large LDL ratios	Percentage
L_VLDL_CE_pct_C	Cholesteryl Esters to Cholesterol in Large VLDL percentage	%	Relative lipoprotein cholesterol concentrations	Large VLDL ratios	Percentage
L_VLDL_FC_by_CE	Free Cholesterol to Cholesteryl Esters in Large VLDL ratio	ratio	Relative lipoprotein lipid concentrations	Large VLDL ratios	Ratio
L_VLDL_FC_pct_C	Free Cholesterol to Cholesterol in Large VLDL percentage	%	Relative lipoprotein cholesterol concentrations	Large VLDL ratios	Percentage
LDL_C_pct	Cholesterol to Total Lipids in LDL percentage	%	Relative lipid concentrations		Percentage
LDL_CE_pct	Cholesteryl Esters to Total Lipids in LDL percentage	%	Relative lipid concentrations		Percentage
LDL_CE_pct_C	Cholesteryl Esters to Cholesterol in LDL percentage	%	Relative cholesterol concentrations		Percentage
LDL_FC_by_CE	Free Cholesterol to Cholesteryl Esters in LDL percentage	ratio	Relative lipid concentrations		Ratio
LDL_FC_pct	Free Cholesterol to Total Lipids in LDL percentage	%	Relative lipid concentrations		Percentage
LDL_FC_pct_C	Free Cholesterol to Cholesterol in LDL percentage	%	Relative cholesterol concentrations		Percentage
LDL_PL_pct	Phospholipids to Total Lipids in LDL percentage	%	Relative lipid concentrations		Percentage
LDL_TG_pct	Triglycerides to Total Lipids in LDL percentage	%	Relative lipid concentrations		Percentage

M_HDL_CE_pct_C	Cholesteryl Esters to Cholesterol in Medium HDL percentage	%	Relative lipoprotein cholesterol concentrations	Medium HDL ratios	Percentage
M_HDL_FC_by_CE	Free Cholesterol to Cholesteryl Esters in Medium HDL ratio	ratio	Relative lipoprotein lipid concentrations	Medium HDL ratios	Ratio
M_HDL_FC_pct_C	Free Cholesterol to Cholesterol in Medium HDL percentage	%	Relative lipoprotein cholesterol concentrations	Medium HDL ratios	Percentage
M_LDL_CE_pct_C	Cholesteryl Esters to Cholesterol in Medium LDL percentage	%	Relative lipoprotein cholesterol concentrations	Medium LDL ratios	Percentage
M_LDL_FC_by_CE	Free Cholesterol to Cholesteryl Esters in Medium LDL ratio	ratio	Relative lipoprotein lipid concentrations	Medium LDL ratios	Ratio
M_LDL_FC_pct_C	Free Cholesterol to Cholesterol in Medium LDL percentage	%	Relative lipoprotein cholesterol concentrations	Medium LDL ratios	Percentage
M_VLDL_CE_pct_C	Cholesteryl Esters to Cholesterol in Medium VLDL percentage	%	Relative lipoprotein cholesterol concentrations	Medium VLDL ratios	Percentage
M_VLDL_FC_by_CE	Free Cholesterol to Cholesteryl Esters in Medium VLDL ratio	ratio	Relative lipoprotein lipid concentrations	Medium VLDL ratios	Ratio
M_VLDL_FC_pct_C	Free Cholesterol to Cholesterol in Medium VLDL percentage	%	Relative lipoprotein cholesterol concentrations	Medium VLDL ratios	Percentage
Omega_3_pct_PUFA	Omega-3 Fatty Acids to Polyunsaturated Fatty Acids percentage	%	Fatty acids	Fatty acid ratios	Percentage
Omega_6_pct_PUFA	Omega-6 Fatty Acids to Polyunsaturated Fatty Acids percentage	%	Fatty acids	Fatty acid ratios	Percentage
S_HDL_CE_pct_C	Cholesteryl Esters to Cholesterol in Small HDL percentage	%	Relative lipoprotein cholesterol concentrations	Small HDL ratios	Percentage
S_HDL_FC_by_CE	Free Cholesterol to Cholesteryl Esters in Small HDL ratio	ratio	Relative lipoprotein lipid concentrations	Small HDL ratios	Ratio
S_HDL_FC_pct_C	Free Cholesterol to Cholesterol in Small HDL percentage	%	Relative lipoprotein cholesterol concentrations	Small HDL ratios	Percentage
S_LDL_CE_pct_C	Cholesteryl Esters to Cholesterol in Small LDL percentage	%	Relative lipoprotein cholesterol concentrations	Small LDL ratios	Percentage
S_LDL_FC_by_CE	Free Cholesterol to Cholesteryl Esters in Small LDL ratio	ratio	Relative lipoprotein lipid concentrations	Small LDL ratios	Ratio

S_LDL_FC_pct_C	Free Cholesterol to Cholesterol in Small LDL percentage	%	Relative lipoprotein cholesterol concentrations	Small LDL ratios	Percentage
S_VLDL_CE_pct_C	Cholesteryl Esters to Cholesterol in Small VLDL percentage	%	Relative lipoprotein cholesterol concentrations	Small VLDL ratios	Percentage
S_VLDL_FC_by_CE	Free Cholesterol to Cholesteryl Esters in Small VLDL ratio	ratio	Relative lipoprotein lipid concentrations	Small VLDL ratios	Ratio
S_VLDL_FC_pct_C	Free Cholesterol to Cholesterol in Small VLDL percentage	%	Relative lipoprotein cholesterol concentrations	Small VLDL ratios	Percentage
Total_C_pct	Total Cholesterol to Total Lipids percentage	%	Relative lipid concentrations		Percentage
Total_CE_pct	Total Esterified Cholesterol to Total Lipids percentage	%	Relative lipid concentrations		Percentage
Total_CE_pct_C	Total Esterified Cholesterol to Total Cholesterol percentage	%	Relative cholesterol concentrations		Percentage
Total_FC_by_CE	Total Free Cholesterol to Total Esterified Cholesterol ratio	ratio	Relative lipid concentrations		Ratio
Total_FC_pct	Total Free Cholesterol to Total Lipids percentage	%	Relative lipid concentrations		Percentage
Total_FC_pct_C	Total Free Cholesterol to Total Cholesterol percentage	%	Relative cholesterol concentrations		Percentage
Total_PL_pct	Total Phospholipids to Total Lipids percentage	%	Relative lipid concentrations		Percentage
Total_TG_pct	Total Triglycerides to Total Lipids percentage	%	Relative lipid concentrations		Percentage
VLDL_C_pct	Cholesterol to Total Lipids in VLDL percentage	%	Relative lipid concentrations		Percentage
VLDL_CE_pct	Cholesteryl Esters to Total Lipids in VLDL percentage	%	Relative lipid concentrations		Percentage
VLDL_CE_pct_C	Cholesteryl Esters to Cholesterol in VLDL percentage	%	Relative cholesterol concentrations		Percentage
VLDL_FC_by_CE	Free Cholesterol to Cholesteryl Esters in VLDL percentage	ratio	Relative lipid concentrations		Ratio
VLDL_FC_pct	Free Cholesterol to Total Lipids in VLDL percentage	%	Relative lipid concentrations		Percentage
VLDL_FC_pct_C	Free Cholesterol to Cholesterol in VLDL percentage	%	Relative cholesterol concentrations		Percentage
VLDL_PL_pct	Phospholipids to Total Lipids in VLDL percentage	%	Relative lipid concentrations		Percentage
VLDL_TG_pct	Triglycerides to Total Lipids in VLDL percentage	%	Relative lipid concentrations		Percentage

XL_HDL_CE_pct_C	Cholesteryl Esters to Cholesterol in Very Large HDL percentage	%	Relative lipoprotein cholesterol concentrations	Very large HDL ratios	Percentage
XL_HDL_FC_by_CE	Free Cholesterol to Cholesteryl Esters in Very Large HDL ratio	ratio	Relative lipoprotein lipid concentrations	Very large HDL ratios	Ratio
XL_HDL_FC_pct_C	Free Cholesterol to Cholesterol in Very Large HDL percentage	%	Relative lipoprotein cholesterol concentrations	Very large HDL ratios	Percentage
XL_VLDL_CE_pct_C	Cholesteryl Esters to Cholesterol in Very Large VLDL percentage	%	Relative lipoprotein cholesterol concentrations	Very large VLDL ratios	Percentage
XL_VLDL_FC_by_CE	Free Cholesterol to Cholesteryl Esters in Very Large VLDL ratio	ratio	Relative lipoprotein lipid concentrations	Very large VLDL ratios	Ratio
XL_VLDL_FC_pct_C	Free Cholesterol to Cholesterol in Very Large VLDL percentage	%	Relative lipoprotein cholesterol concentrations	Very large VLDL ratios	Percentage
XS_VLDL_CE_pct_C	Cholesteryl Esters to Cholesterol in Very Small VLDL percentage	%	Relative lipoprotein cholesterol concentrations	Very small VLDL ratios	Percentage
XS_VLDL_FC_by_CE	Free Cholesterol to Cholesteryl Esters in Very Small VLDL ratio	ratio	Relative lipoprotein lipid concentrations	Very small VLDL ratios	Ratio
XS_VLDL_FC_pct_C	Free Cholesterol to Cholesterol in Very Small VLDL percentage	%	Relative lipoprotein cholesterol concentrations	Very small VLDL ratios	Percentage
XXL_VLDL_CE_pct_C	Cholesteryl Esters to Cholesterol in Chylomicrons and Extremely Large VLDL percentage	%	Relative lipoprotein cholesterol concentrations	Chylomicrons and extremely large VLDL ratios	Percentage
XXL_VLDL_FC_by_CE	Free Cholesterol to Cholesteryl Esters in Chylomicrons and Extremely Large VLDL ratio	ratio	Relative lipoprotein lipid concentrations	Chylomicrons and extremely large VLDL ratios	Ratio
XXL_VLDL_FC_pct_C	Free Cholesterol to Cholesterol in Chylomicrons and Extremely Large VLDL percentage	%	Relative lipoprotein cholesterol concentrations	Chylomicrons and extremely large VLDL ratios	Percentage

Table S3. Clinical data and abbreviations

Name	Abbreviation
Age at recruitment	Age
Sex	Sex
Waist circumference	WC
Body mass index (BMI)	BMI
Basophill count	BA
Basophill percentage	BA%
Eosinophill count	EO
Eosinophill percentage	EO%
Haematocrit percentage	HCT%
Haemoglobin concentration	HGB
High light scatter reticulocyte count	HLR
High light scatter reticulocyte percentage	HLR%
Immature reticulocyte fraction	IRF
Lymphocyte count	LY
Lymphocyte percentage	LY%
Mean corpuscular haemoglobin	MCH
Mean corpuscular haemoglobin concentration	MCHC
Mean corpuscular volume	MCV
Mean platelet (thrombocyte) volume	MPV
Mean reticulocyte volume	MRV
Mean sphered cell volume	MSCV
Monocyte count	MO
Monocyte percentage	MO%
Neutrophill count	NE
Neutrophill percentage	NE%
Nucleated red blood cell count	NRBC
Nucleated red blood cell percentage	NRBC%
Platelet count	PLT
Platelet crit	PCT
Platelet distribution width	PDW
Red blood cell (erythrocyte) count	RBC
Red blood cell (erythrocyte) distribution width	RDW
Reticulocyte count	RET
Reticulocyte percentage	RET%
White blood cell (leukocyte) count	WBC
Alanine aminotransferase	ALT
Albumin_2	ALB
Alkaline phosphatase	ALP
Apolipoprotein A	APOA
Apolipoprotein B_2	APOB
Aspartate aminotransferase	AST
C-reactive protein	CRP
Calcium	CA
Cholesterol	CHOL
Creatinine_2	CRE
Cystatin C	CYS
Direct bilirubin	BILD
Gamma glutamyltransferase	GGT
Glucose_2	GLU
Glycated haemoglobin (HbA1c)	HBA1C
HDL cholesterol	HDL
IGF-1	IGF-1
LDL direct	LDLD

Phosphate	PHOS
SHBG	SHBG
Testosterone	TES
Total bilirubin	TBIL
Total protein	TP
Triglycerides	TRIG
Urate	UA
Urea	BUN
Vitamin D	VITD
Creatinine (enzymatic) in urine	UCr
Potassium in urine	UK
Sodium in urine	UNa
Hip circumference	HC

Table S4. Prevalence of cardio-metabolic medication use in CVD and non-CVD cohorts

Metabolites	Description	Units	Group	Sub-group	Type	UKB Field ID	QC Flag Field ID
Total_C	Total Cholesterol	mmol/L	Cholesterol		Composite	23400	23700
non_HDL_C	Total Cholesterol Minus HDL-C	mmol/L	Cholesterol		Composite	23401	23701
Remnant_C	Remnant Cholesterol (Non-HDL, Non-LDL -Cholesterol)	mmol/L	Cholesterol		Composite	23402	23702
VLDL_C	VLDL Cholesterol	mmol/L	Cholesterol		Composite	23403	23703
Clinical_LDL_C	Clinical LDL Cholesterol	mmol/L	Cholesterol		Non-derived	23404	23704
LDL_C	LDL Cholesterol	mmol/L	Cholesterol		Composite	23405	23705
HDL_C	HDL Cholesterol	mmol/L	Cholesterol		Composite	23406	23706
Total_TG	Total Triglycerides	mmol/L	Triglycerides		Composite	23407	23707
VLDL_TG	Triglycerides in VLDL	mmol/L	Triglycerides		Composite	23408	23708
LDL_TG	Triglycerides in LDL	mmol/L	Triglycerides		Composite	23409	23709
HDL_TG	Triglycerides in HDL	mmol/L	Triglycerides		Composite	23410	23710
Total_PL	Total Phospholipids in Lipoprotein Particles	mmol/L	Phospholipids		Composite	23411	23711
VLDL_PL	Phospholipids in VLDL	mmol/L	Phospholipids		Composite	23412	23712
LDL_PL	Phospholipids in LDL	mmol/L	Phospholipids		Composite	23413	23713
HDL_PL	Phospholipids in HDL	mmol/L	Phospholipids		Composite	23414	23714
Total_CE	Total Esterified Cholesterol	mmol/L	Cholesteryl esters		Composite	23415	23715
VLDL_CE	Cholesteryl Esters in VLDL	mmol/L	Cholesteryl esters		Composite	23416	23716
LDL_CE	Cholesteryl Esters in LDL	mmol/L	Cholesteryl esters		Composite	23417	23717
HDL_CE	Cholesteryl Esters in HDL	mmol/L	Cholesteryl esters		Composite	23418	23718

Total_FC	Total Free Cholesterol	mmol/L	Free cholesterol	Composite	23419	23719
VLDL_FC	Free Cholesterol in VLDL	mmol/L	Free cholesterol	Composite	23420	23720
LDL_FC	Free Cholesterol in LDL	mmol/L	Free cholesterol	Composite	23421	23721
HDL_FC	Free Cholesterol in HDL	mmol/L	Free cholesterol	Composite	23422	23722
Total_L	Total Lipids in Lipoprotein Particles	mmol/L	Total lipids	Composite	23423	23723
VLDL_L	Total Lipids in VLDL	mmol/L	Total lipids	Composite	23424	23724
LDL_L	Total Lipids in LDL	mmol/L	Total lipids	Composite	23425	23725
HDL_L	Total Lipids in HDL	mmol/L	Total lipids	Composite	23426	23726
Total_P	Total Concentration of Lipoprotein Particles	mmol/L	Lipoprotein particle concentrations	Composite	23427	23727
VLDL_P	Concentration of VLDL Particles	mmol/L	Lipoprotein particle concentrations	Composite	23428	23728
LDL_P	Concentration of LDL Particles	mmol/L	Lipoprotein particle concentrations	Composite	23429	23729
HDL_P	Concentration of HDL Particles	mmol/L	Lipoprotein particle concentrations	Composite	23430	23730
VLDL_size	Average Diameter for VLDL Particles	nm	Lipoprotein particle sizes	Non-derived	23431	23731
LDL_size	Average Diameter for LDL Particles	nm	Lipoprotein particle sizes	Non-derived	23432	23732
HDL_size	Average Diameter for HDL Particles	nm	Lipoprotein particle sizes	Non-derived	23433	23733
Phosphoglyc	Phosphoglycerides	mmol/L	Other lipids	Non-derived	23434	23734
TG_by_PG	Triglycerides to Phosphoglycerides ratio	ratio	Other lipids	Ratio	23435	23735
Cholines	Total Cholines	mmol/L	Other lipids	Non-derived	23436	23736
Phosphatidylc	Phosphatidylcholines	mmol/L	Other lipids	Non-derived	23437	23737
Sphingomyelins	Sphingomyelins	mmol/L	Other lipids	Non-derived	23438	23738
ApoB	Apolipoprotein B	g/l	Apolipoproteins	Non-derived	23439	23739
ApoA1	Apolipoprotein A1	g/l	Apolipoproteins	Non-derived	23440	23740

ApoB_by_ApoA1	Apolipoprotein B to Apolipoprotein A1 ratio	ratio	Apolipoproteins		Ratio	23441	23741
Total_FA	Total Fatty Acids	mmol/L	Fatty acids		Composite	23442	23742
Unsaturation	Degree of Unsaturation	degree	Fatty acids		Non-derived	23443	23743
Omega_3	Omega-3 Fatty Acids	mmol/L	Fatty acids		Non-derived	23444	23744
Omega_6	Omega-6 Fatty Acids	mmol/L	Fatty acids		Non-derived	23445	23745
PUFA	Polyunsaturated Fatty Acids	mmol/L	Fatty acids		Composite	23446	23746
MUFA	Monounsaturated Fatty Acids	mmol/L	Fatty acids		Non-derived	23447	23747
SFA	Saturated Fatty Acids	mmol/L	Fatty acids		Non-derived	23448	23748
LA	Linoleic Acid	mmol/L	Fatty acids		Non-derived	23449	23749
DHA	Docosahexaenoic Acid	mmol/L	Fatty acids		Non-derived	23450	23750
Omega_3_pct	Omega-3 Fatty Acids to Total Fatty Acids percentage	%	Fatty acids	Fatty acid ratios	Percentage	23451	23751
Omega_6_pct	Omega-6 Fatty Acids to Total Fatty Acids percentage	%	Fatty acids	Fatty acid ratios	Percentage	23452	23752
PUFA_pct	Polyunsaturated Fatty Acids to Total Fatty Acids percentage	%	Fatty acids	Fatty acid ratios	Percentage	23453	23753
MUFA_pct	Monounsaturated Fatty Acids to Total Fatty Acids percentage	%	Fatty acids	Fatty acid ratios	Percentage	23454	23754
SFA_pct	Saturated Fatty Acids to Total Fatty Acids percentage	%	Fatty acids	Fatty acid ratios	Percentage	23455	23755
LA_pct	Linoleic Acid to Total Fatty Acids percentage	%	Fatty acids	Fatty acid ratios	Percentage	23456	23756
DHA_pct	Docosahexaenoic Acid to Total Fatty Acids percentage	%	Fatty acids	Fatty acid ratios	Percentage	23457	23757
PUFA_by_MUFA	Polyunsaturated Fatty Acids to Monounsaturated Fatty Acids ratio	ratio	Fatty acids	Fatty acid ratios	Ratio	23458	23758
Omega_6_by_Omega_3	Omega-6 Fatty Acids to Omega-3 Fatty Acids ratio	ratio	Fatty acids	Fatty acid ratios	Ratio	23459	23759
Ala	Alanine	mmol/L	Amino acids		Non-derived	23460	23760
Gln	Glutamine	mmol/L	Amino acids		Non-derived	23461	23761
Gly	Glycine	mmol/L	Amino acids		Non-derived	23462	23762

His	Histidine	mmol/L	Amino acids		Non-derived	23463	23763
Total_BCAA	Total Concentration of Branched-Chain Amino Acids (Leucine + Isoleucine + Valine)	mmol/L	Amino acids	Branched-chain amino acids	Composite	23464	23764
Ile	Isoleucine	mmol/L	Amino acids	Branched-chain amino acids	Non-derived	23465	23765
Leu	Leucine	mmol/L	Amino acids	Branched-chain amino acids	Non-derived	23466	23766
Val	Valine	mmol/L	Amino acids	Branched-chain amino acids	Non-derived	23467	23767
Phe	Phenylalanine	mmol/L	Amino acids	Aromatic amino acids	Non-derived	23468	23768
Tyr	Tyrosine	mmol/L	Amino acids	Aromatic amino acids	Non-derived	23469	23769
Glucose	Glucose	mmol/L	Glycolysis related metabolites		Non-derived	23470	23770
Lactate	Lactate	mmol/L	Glycolysis related metabolites		Non-derived	23471	23771
Pyruvate	Pyruvate	mmol/L	Glycolysis related metabolites		Non-derived	23472	23772
Citrate	Citrate	mmol/L	Glycolysis related metabolites		Non-derived	23473	23773
bOHbutyrate	3-Hydroxybutyrate	mmol/L	Ketone bodies		Non-derived	23474	23774
Acetate	Acetate	mmol/L	Ketone bodies		Non-derived	23475	23775
Acetoacetate	Acetoacetate	mmol/L	Ketone bodies		Non-derived	23476	23776
Acetone	Acetone	mmol/L	Ketone bodies		Non-derived	23477	23777
Creatinine	Creatinine	mmol/L	Fluid balance		Non-derived	23478	23778
Albumin	Albumin	g/l	Fluid balance		Non-derived	23479	23779
GlycA	Glycoprotein Acetyls	mmol/L	Inflammation		Non-derived	23480	23780
XXL_VLDL_P	Concentration of Chylomicrons and Extremely Large VLDL Particles	mmol/L	Lipoprotein subclasses	Chylomicrons and extremely large VLDL (particle diameters from 75 nm upwards)	Non-derived	23481	23781
XXL_VLDL_L	Total Lipids in Chylomicrons and Extremely Large VLDL	mmol/L	Lipoprotein subclasses	Chylomicrons and extremely large VLDL (particle diameters from 75 nm upwards)	Composite	23482	23782

XXL_VLDL_PL	Phospholipids in Chylomicrons and Extremely Large VLDL	mmol/L	Lipoprotein subclasses	Chylomicrons and extremely large VLDL (particle diameters from 75 nm upwards)	Non-derived	23483	23783
XXL_VLDL_C	Cholesterol in Chylomicrons and Extremely Large VLDL	mmol/L	Lipoprotein subclasses	Chylomicrons and extremely large VLDL (particle diameters from 75 nm upwards)	Composite	23484	23784
XXL_VLDL_CE	Cholesteryl Esters in Chylomicrons and Extremely Large VLDL	mmol/L	Lipoprotein subclasses	Chylomicrons and extremely large VLDL (particle diameters from 75 nm upwards)	Non-derived	23485	23785
XXL_VLDL_FC	Free Cholesterol in Chylomicrons and Extremely Large VLDL	mmol/L	Lipoprotein subclasses	Chylomicrons and extremely large VLDL (particle diameters from 75 nm upwards)	Non-derived	23486	23786
XXL_VLDL_TG	Triglycerides in Chylomicrons and Extremely Large VLDL	mmol/L	Lipoprotein subclasses	Chylomicrons and extremely large VLDL (particle diameters from 75 nm upwards)	Non-derived	23487	23787
XL_VLDL_P	Concentration of Very Large VLDL Particles	mmol/L	Lipoprotein subclasses	Very large VLDL (average diameter 64 nm)	Non-derived	23488	23788
XL_VLDL_L	Total Lipids in Very Large VLDL	mmol/L	Lipoprotein subclasses	Very large VLDL (average diameter 64 nm)	Composite	23489	23789
XL_VLDL_PL	Phospholipids in Very Large VLDL	mmol/L	Lipoprotein subclasses	Very large VLDL (average diameter 64 nm)	Non-derived	23490	23790
XL_VLDL_C	Cholesterol in Very Large VLDL	mmol/L	Lipoprotein subclasses	Very large VLDL (average diameter 64 nm)	Composite	23491	23791
XL_VLDL_CE	Cholesteryl Esters in Very Large VLDL	mmol/L	Lipoprotein subclasses	Very large VLDL (average diameter 64 nm)	Non-derived	23492	23792
XL_VLDL_FC	Free Cholesterol in Very Large VLDL	mmol/L	Lipoprotein subclasses	Very large VLDL (average diameter 64 nm)	Non-derived	23493	23793
XL_VLDL_TG	Triglycerides in Very Large VLDL	mmol/L	Lipoprotein subclasses	Very large VLDL (average diameter 64 nm)	Non-derived	23494	23794
L_VLDL_P	Concentration of Large VLDL Particles	mmol/L	Lipoprotein subclasses	Large VLDL (average diameter 53.6 nm)	Non-derived	23495	23795
L_VLDL_L	Total Lipids in Large VLDL	mmol/L	Lipoprotein subclasses	Large VLDL (average diameter 53.6 nm)	Composite	23496	23796
L_VLDL_PL	Phospholipids in Large VLDL	mmol/L	Lipoprotein subclasses	Large VLDL (average diameter 53.6 nm)	Non-derived	23497	23797
L_VLDL_C	Cholesterol in Large VLDL	mmol/L	Lipoprotein subclasses	Large VLDL (average diameter 53.6 nm)	Composite	23498	23798
L_VLDL_CE	Cholesteryl Esters in Large VLDL	mmol/L	Lipoprotein subclasses	Large VLDL (average diameter 53.6 nm)	Non-derived	23499	23799
L_VLDL_FC	Free Cholesterol in Large VLDL	mmol/L	Lipoprotein subclasses	Large VLDL (average diameter 53.6 nm)	Non-derived	23500	23800
L_VLDL_TG	Triglycerides in Large VLDL	mmol/L	Lipoprotein subclasses	Large VLDL (average diameter 53.6 nm)	Non-derived	23501	23801

M_VLDL_P	Concentration of Medium VLDL Particles	mmol/L	Lipoprotein subclasses	Medium VLDL (average diameter 44.5 nm)	Non-derived	23502	23802
M_VLDL_L	Total Lipids in Medium VLDL	mmol/L	Lipoprotein subclasses	Medium VLDL (average diameter 44.5 nm)	Composite	23503	23803
M_VLDL_PL	Phospholipids in Medium VLDL	mmol/L	Lipoprotein subclasses	Medium VLDL (average diameter 44.5 nm)	Non-derived	23504	23804
M_VLDL_C	Cholesterol in Medium VLDL	mmol/L	Lipoprotein subclasses	Medium VLDL (average diameter 44.5 nm)	Composite	23505	23805
M_VLDL_CE	Cholesteryl Esters in Medium VLDL	mmol/L	Lipoprotein subclasses	Medium VLDL (average diameter 44.5 nm)	Non-derived	23506	23806
M_VLDL_FC	Free Cholesterol in Medium VLDL	mmol/L	Lipoprotein subclasses	Medium VLDL (average diameter 44.5 nm)	Non-derived	23507	23807
M_VLDL_TG	Triglycerides in Medium VLDL	mmol/L	Lipoprotein subclasses	Medium VLDL (average diameter 44.5 nm)	Non-derived	23508	23808
S_VLDL_P	Concentration of Small VLDL Particles	mmol/L	Lipoprotein subclasses	Small VLDL (average diameter 36.8 nm)	Non-derived	23509	23809
S_VLDL_L	Total Lipids in Small VLDL	mmol/L	Lipoprotein subclasses	Small VLDL (average diameter 36.8 nm)	Composite	23510	23810
S_VLDL_PL	Phospholipids in Small VLDL	mmol/L	Lipoprotein subclasses	Small VLDL (average diameter 36.8 nm)	Non-derived	23511	23811
S_VLDL_C	Cholesterol in Small VLDL	mmol/L	Lipoprotein subclasses	Small VLDL (average diameter 36.8 nm)	Composite	23512	23812
S_VLDL_CE	Cholesteryl Esters in Small VLDL	mmol/L	Lipoprotein subclasses	Small VLDL (average diameter 36.8 nm)	Non-derived	23513	23813
S_VLDL_FC	Free Cholesterol in Small VLDL	mmol/L	Lipoprotein subclasses	Small VLDL (average diameter 36.8 nm)	Non-derived	23514	23814
S_VLDL_TG	Triglycerides in Small VLDL	mmol/L	Lipoprotein subclasses	Small VLDL (average diameter 36.8 nm)	Non-derived	23515	23815
XS_VLDL_P	Concentration of Very Small VLDL Particles	mmol/L	Lipoprotein subclasses	Very small VLDL (average diameter 31.3 nm)	Non-derived	23516	23816
XS_VLDL_L	Total Lipids in Very Small VLDL	mmol/L	Lipoprotein subclasses	Very small VLDL (average diameter 31.3 nm)	Composite	23517	23817
XS_VLDL_PL	Phospholipids in Very Small VLDL	mmol/L	Lipoprotein subclasses	Very small VLDL (average diameter 31.3 nm)	Non-derived	23518	23818
XS_VLDL_C	Cholesterol in Very Small VLDL	mmol/L	Lipoprotein subclasses	Very small VLDL (average diameter 31.3 nm)	Composite	23519	23819
XS_VLDL_CE	Cholesteryl Esters in Very Small VLDL	mmol/L	Lipoprotein subclasses	Very small VLDL (average diameter 31.3 nm)	Non-derived	23520	23820
XS_VLDL_FC	Free Cholesterol in Very Small VLDL	mmol/L	Lipoprotein subclasses	Very small VLDL (average diameter 31.3 nm)	Non-derived	23521	23821
XS_VLDL_TG	Triglycerides in Very Small VLDL	mmol/L	Lipoprotein subclasses	Very small VLDL (average diameter 31.3 nm)	Non-derived	23522	23822

IDL_P	Concentration of IDL Particles	mmol/L	Lipoprotein subclasses	IDL (average diameter 28.6 nm)	Non-derived	23523	23823
IDL_L	Total Lipids in IDL	mmol/L	Lipoprotein subclasses	IDL (average diameter 28.6 nm)	Composite	23524	23824
IDL_PL	Phospholipids in IDL	mmol/L	Lipoprotein subclasses	IDL (average diameter 28.6 nm)	Non-derived	23525	23825
IDL_C	Cholesterol in IDL	mmol/L	Lipoprotein subclasses	IDL (average diameter 28.6 nm)	Composite	23526	23826
IDL_CE	Cholesteryl Esters in IDL	mmol/L	Lipoprotein subclasses	IDL (average diameter 28.6 nm)	Non-derived	23527	23827
IDL_FC	Free Cholesterol in IDL	mmol/L	Lipoprotein subclasses	IDL (average diameter 28.6 nm)	Non-derived	23528	23828
IDL_TG	Triglycerides in IDL	mmol/L	Lipoprotein subclasses	IDL (average diameter 28.6 nm)	Non-derived	23529	23829
L_LDL_P	Concentration of Large LDL Particles	mmol/L	Lipoprotein subclasses	Large LDL (average diameter 25.5 nm)	Non-derived	23530	23830
L_LDL_L	Total Lipids in Large LDL	mmol/L	Lipoprotein subclasses	Large LDL (average diameter 25.5 nm)	Composite	23531	23831
L_LDL_PL	Phospholipids in Large LDL	mmol/L	Lipoprotein subclasses	Large LDL (average diameter 25.5 nm)	Non-derived	23532	23832
L_LDL_C	Cholesterol in Large LDL	mmol/L	Lipoprotein subclasses	Large LDL (average diameter 25.5 nm)	Composite	23533	23833
L_LDL_CE	Cholesteryl Esters in Large LDL	mmol/L	Lipoprotein subclasses	Large LDL (average diameter 25.5 nm)	Non-derived	23534	23834
L_LDL_FC	Free Cholesterol in Large LDL	mmol/L	Lipoprotein subclasses	Large LDL (average diameter 25.5 nm)	Non-derived	23535	23835
L_LDL_TG	Triglycerides in Large LDL	mmol/L	Lipoprotein subclasses	Large LDL (average diameter 25.5 nm)	Non-derived	23536	23836
M_LDL_P	Concentration of Medium LDL Particles	mmol/L	Lipoprotein subclasses	Medium LDL (average diameter 23 nm)	Non-derived	23537	23837
M_LDL_L	Total Lipids in Medium LDL	mmol/L	Lipoprotein subclasses	Medium LDL (average diameter 23 nm)	Composite	23538	23838
M_LDL_PL	Phospholipids in Medium LDL	mmol/L	Lipoprotein subclasses	Medium LDL (average diameter 23 nm)	Non-derived	23539	23839
M_LDL_C	Cholesterol in Medium LDL	mmol/L	Lipoprotein subclasses	Medium LDL (average diameter 23 nm)	Composite	23540	23840
M_LDL_CE	Cholesteryl Esters in Medium LDL	mmol/L	Lipoprotein subclasses	Medium LDL (average diameter 23 nm)	Non-derived	23541	23841
M_LDL_FC	Free Cholesterol in Medium LDL	mmol/L	Lipoprotein subclasses	Medium LDL (average diameter 23 nm)	Non-derived	23542	23842
M_LDL_TG	Triglycerides in Medium LDL	mmol/L	Lipoprotein subclasses	Medium LDL (average diameter 23 nm)	Non-derived	23543	23843

S_LDL_P	Concentration of Small LDL Particles	mmol/L	Lipoprotein subclasses	Small LDL (average diameter 18.7 nm)	Non-derived	23544	23844
S_LDL_L	Total Lipids in Small LDL	mmol/L	Lipoprotein subclasses	Small LDL (average diameter 18.7 nm)	Composite	23545	23845
S_LDL_PL	Phospholipids in Small LDL	mmol/L	Lipoprotein subclasses	Small LDL (average diameter 18.7 nm)	Non-derived	23546	23846
S_LDL_C	Cholesterol in Small LDL	mmol/L	Lipoprotein subclasses	Small LDL (average diameter 18.7 nm)	Composite	23547	23847
S_LDL_CE	Cholesteryl Esters in Small LDL	mmol/L	Lipoprotein subclasses	Small LDL (average diameter 18.7 nm)	Non-derived	23548	23848
S_LDL_FC	Free Cholesterol in Small LDL	mmol/L	Lipoprotein subclasses	Small LDL (average diameter 18.7 nm)	Non-derived	23549	23849
S_LDL_TG	Triglycerides in Small LDL	mmol/L	Lipoprotein subclasses	Small LDL (average diameter 18.7 nm)	Non-derived	23550	23850
XL_HDL_P	Concentration of Very Large HDL Particles	mmol/L	Lipoprotein subclasses	Very large HDL (average diameter 14.3 nm)	Non-derived	23551	23851
XL_HDL_L	Total Lipids in Very Large HDL	mmol/L	Lipoprotein subclasses	Very large HDL (average diameter 14.3 nm)	Composite	23552	23852
XL_HDL_PL	Phospholipids in Very Large HDL	mmol/L	Lipoprotein subclasses	Very large HDL (average diameter 14.3 nm)	Non-derived	23553	23853
XL_HDL_C	Cholesterol in Very Large HDL	mmol/L	Lipoprotein subclasses	Very large HDL (average diameter 14.3 nm)	Composite	23554	23854
XL_HDL_CE	Cholesteryl Esters in Very Large HDL	mmol/L	Lipoprotein subclasses	Very large HDL (average diameter 14.3 nm)	Non-derived	23555	23855
XL_HDL_FC	Free Cholesterol in Very Large HDL	mmol/L	Lipoprotein subclasses	Very large HDL (average diameter 14.3 nm)	Non-derived	23556	23856
XL_HDL_TG	Triglycerides in Very Large HDL	mmol/L	Lipoprotein subclasses	Very large HDL (average diameter 14.3 nm)	Non-derived	23557	23857
L_HDL_P	Concentration of Large HDL Particles	mmol/L	Lipoprotein subclasses	Large HDL (average diameter 12.1 nm)	Non-derived	23558	23858
L_HDL_L	Total Lipids in Large HDL	mmol/L	Lipoprotein subclasses	Large HDL (average diameter 12.1 nm)	Composite	23559	23859
L_HDL_PL	Phospholipids in Large HDL	mmol/L	Lipoprotein subclasses	Large HDL (average diameter 12.1 nm)	Non-derived	23560	23860
L_HDL_C	Cholesterol in Large HDL	mmol/L	Lipoprotein subclasses	Large HDL (average diameter 12.1 nm)	Composite	23561	23861
L_HDL_CE	Cholesteryl Esters in Large HDL	mmol/L	Lipoprotein subclasses	Large HDL (average diameter 12.1 nm)	Non-derived	23562	23862
L_HDL_FC	Free Cholesterol in Large HDL	mmol/L	Lipoprotein subclasses	Large HDL (average diameter 12.1 nm)	Non-derived	23563	23863
L_HDL_TG	Triglycerides in Large HDL	mmol/L	Lipoprotein subclasses	Large HDL (average diameter 12.1 nm)	Non-derived	23564	23864

M_HDL_P	Concentration of Medium HDL Particles	mmol/L	Lipoprotein subclasses	Medium HDL (average diameter 10.9 nm)	Non-derived	23565	23865
M_HDL_L	Total Lipids in Medium HDL	mmol/L	Lipoprotein subclasses	Medium HDL (average diameter 10.9 nm)	Composite	23566	23866
M_HDL_PL	Phospholipids in Medium HDL	mmol/L	Lipoprotein subclasses	Medium HDL (average diameter 10.9 nm)	Non-derived	23567	23867
M_HDL_C	Cholesterol in Medium HDL	mmol/L	Lipoprotein subclasses	Medium HDL (average diameter 10.9 nm)	Composite	23568	23868
M_HDL_CE	Cholesteryl Esters in Medium HDL	mmol/L	Lipoprotein subclasses	Medium HDL (average diameter 10.9 nm)	Non-derived	23569	23869
M_HDL_FC	Free Cholesterol in Medium HDL	mmol/L	Lipoprotein subclasses	Medium HDL (average diameter 10.9 nm)	Non-derived	23570	23870
M_HDL_TG	Triglycerides in Medium HDL	mmol/L	Lipoprotein subclasses	Medium HDL (average diameter 10.9 nm)	Non-derived	23571	23871
S_HDL_P	Concentration of Small HDL Particles	mmol/L	Lipoprotein subclasses	Small HDL (average diameter 8.7 nm)	Non-derived	23572	23872
S_HDL_L	Total Lipids in Small HDL	mmol/L	Lipoprotein subclasses	Small HDL (average diameter 8.7 nm)	Composite	23573	23873
S_HDL_PL	Phospholipids in Small HDL	mmol/L	Lipoprotein subclasses	Small HDL (average diameter 8.7 nm)	Non-derived	23574	23874
S_HDL_C	Cholesterol in Small HDL	mmol/L	Lipoprotein subclasses	Small HDL (average diameter 8.7 nm)	Composite	23575	23875
S_HDL_CE	Cholesteryl Esters in Small HDL	mmol/L	Lipoprotein subclasses	Small HDL (average diameter 8.7 nm)	Non-derived	23576	23876
S_HDL_FC	Free Cholesterol in Small HDL	mmol/L	Lipoprotein subclasses	Small HDL (average diameter 8.7 nm)	Non-derived	23577	23877
S_HDL_TG	Triglycerides in Small HDL	mmol/L	Lipoprotein subclasses	Small HDL (average diameter 8.7 nm)	Non-derived	23578	23878
XXL_VLDL_PL_pct	Phospholipids to Total Lipids in Chylomicrons and Extremely Large VLDL percentage	%	Relative lipoprotein lipid concentrations	Chylomicrons and extremely large VLDL ratios	Percentage	23579	23879
XXL_VLDL_C_pct	Cholesterol to Total Lipids in Chylomicrons and Extremely Large VLDL percentage	%	Relative lipoprotein lipid concentrations	Chylomicrons and extremely large VLDL ratios	Percentage	23580	23880
XXL_VLDL_CE_pct	Cholesteryl Esters to Total Lipids in Chylomicrons and Extremely Large VLDL percentage	%	Relative lipoprotein lipid concentrations	Chylomicrons and extremely large VLDL ratios	Percentage	23581	23881
XXL_VLDL_FC_pct	Free Cholesterol to Total Lipids in Chylomicrons and Extremely Large VLDL percentage	%	Relative lipoprotein lipid concentrations	Chylomicrons and extremely large VLDL ratios	Percentage	23582	23882
XXL_VLDL_TG_pct	Triglycerides to Total Lipids in Chylomicrons and Extremely Large VLDL percentage	%	Relative lipoprotein lipid concentrations	Chylomicrons and extremely large VLDL ratios	Percentage	23583	23883
XL_VLDL_PL_pct	Phospholipids to Total Lipids in Very Large VLDL percentage	%	Relative lipoprotein lipid concentrations	Very large VLDL ratios	Percentage	23584	23884
XL_VLDL_C_pct	Cholesterol to Total Lipids in Very Large VLDL percentage	%	Relative lipoprotein lipid concentrations	Very large VLDL ratios	Percentage	23585	23885

XL_VLDL_CE_pct	Cholesteryl Esters to Total Lipids in Very Large VLDL percentage	%	Relative lipoprotein lipid concentrations	Very large VLDL ratios	Percentage	23586	23886
XL_VLDL_FC_pct	Free Cholesterol to Total Lipids in Very Large VLDL percentage	%	Relative lipoprotein lipid concentrations	Very large VLDL ratios	Percentage	23587	23887
XL_VLDL_TG_pct	Triglycerides to Total Lipids in Very Large VLDL percentage	%	Relative lipoprotein lipid concentrations	Very large VLDL ratios	Percentage	23588	23888
L_VLDL_PL_pct	Phospholipids to Total Lipids in Large VLDL percentage	%	Relative lipoprotein lipid concentrations	Large VLDL ratios	Percentage	23589	23889
L_VLDL_C_pct	Cholesterol to Total Lipids in Large VLDL percentage	%	Relative lipoprotein lipid concentrations	Large VLDL ratios	Percentage	23590	23890
L_VLDL_CE_pct	Cholesteryl Esters to Total Lipids in Large VLDL percentage	%	Relative lipoprotein lipid concentrations	Large VLDL ratios	Percentage	23591	23891
L_VLDL_FC_pct	Free Cholesterol to Total Lipids in Large VLDL percentage	%	Relative lipoprotein lipid concentrations	Large VLDL ratios	Percentage	23592	23892
L_VLDL_TG_pct	Triglycerides to Total Lipids in Large VLDL percentage	%	Relative lipoprotein lipid concentrations	Large VLDL ratios	Percentage	23593	23893
M_VLDL_PL_pct	Phospholipids to Total Lipids in Medium VLDL percentage	%	Relative lipoprotein lipid concentrations	Medium VLDL ratios	Percentage	23594	23894
M_VLDL_C_pct	Cholesterol to Total Lipids in Medium VLDL percentage	%	Relative lipoprotein lipid concentrations	Medium VLDL ratios	Percentage	23595	23895
M_VLDL_CE_pct	Cholesteryl Esters to Total Lipids in Medium VLDL percentage	%	Relative lipoprotein lipid concentrations	Medium VLDL ratios	Percentage	23596	23896
M_VLDL_FC_pct	Free Cholesterol to Total Lipids in Medium VLDL percentage	%	Relative lipoprotein lipid concentrations	Medium VLDL ratios	Percentage	23597	23897
M_VLDL_TG_pct	Triglycerides to Total Lipids in Medium VLDL percentage	%	Relative lipoprotein lipid concentrations	Medium VLDL ratios	Percentage	23598	23898
S_VLDL_PL_pct	Phospholipids to Total Lipids in Small VLDL percentage	%	Relative lipoprotein lipid concentrations	Small VLDL ratios	Percentage	23599	23899
S_VLDL_C_pct	Cholesterol to Total Lipids in Small VLDL percentage	%	Relative lipoprotein lipid concentrations	Small VLDL ratios	Percentage	23600	23900
S_VLDL_CE_pct	Cholesteryl Esters to Total Lipids in Small VLDL percentage	%	Relative lipoprotein lipid concentrations	Small VLDL ratios	Percentage	23601	23901
S_VLDL_FC_pct	Free Cholesterol to Total Lipids in Small VLDL percentage	%	Relative lipoprotein lipid concentrations	Small VLDL ratios	Percentage	23602	23902
S_VLDL_TG_pct	Triglycerides to Total Lipids in Small VLDL percentage	%	Relative lipoprotein lipid concentrations	Small VLDL ratios	Percentage	23603	23903
XS_VLDL_PL_pct	Phospholipids to Total Lipids in Very Small VLDL percentage	%	Relative lipoprotein lipid concentrations	Very small VLDL ratios	Percentage	23604	23904
XS_VLDL_C_pct	Cholesterol to Total Lipids in Very Small VLDL percentage	%	Relative lipoprotein lipid concentrations	Very small VLDL ratios	Percentage	23605	23905
XS_VLDL_CE_pct	Cholesteryl Esters to Total Lipids in Very Small VLDL percentage	%	Relative lipoprotein lipid concentrations	Very small VLDL ratios	Percentage	23606	23906

XS_VLDL_FC_pct	Free Cholesterol to Total Lipids in Very Small VLDL percentage	%	Relative lipoprotein lipid concentrations	Very small VLDL ratios	Percentage	23607	23907
XS_VLDL_TG_pct	Triglycerides to Total Lipids in Very Small VLDL percentage	%	Relative lipoprotein lipid concentrations	Very small VLDL ratios	Percentage	23608	23908
IDL_PL_pct	Phospholipids to Total Lipids in IDL percentage	%	Relative lipoprotein lipid concentrations	IDL ratios	Percentage	23609	23909
IDL_C_pct	Cholesterol to Total Lipids in IDL percentage	%	Relative lipoprotein lipid concentrations	IDL ratios	Percentage	23610	23910
IDL_CE_pct	Cholesteryl Esters to Total Lipids in IDL percentage	%	Relative lipoprotein lipid concentrations	IDL ratios	Percentage	23611	23911
IDL_FC_pct	Free Cholesterol to Total Lipids in IDL percentage	%	Relative lipoprotein lipid concentrations	IDL ratios	Percentage	23612	23912
IDL_TG_pct	Triglycerides to Total Lipids in IDL percentage	%	Relative lipoprotein lipid concentrations	IDL ratios	Percentage	23613	23913
L_LDL_PL_pct	Phospholipids to Total Lipids in Large LDL percentage	%	Relative lipoprotein lipid concentrations	Large LDL ratios	Percentage	23614	23914
L_LDL_C_pct	Cholesterol to Total Lipids in Large LDL percentage	%	Relative lipoprotein lipid concentrations	Large LDL ratios	Percentage	23615	23915
L_LDL_CE_pct	Cholesteryl Esters to Total Lipids in Large LDL percentage	%	Relative lipoprotein lipid concentrations	Large LDL ratios	Percentage	23616	23916
L_LDL_FC_pct	Free Cholesterol to Total Lipids in Large LDL percentage	%	Relative lipoprotein lipid concentrations	Large LDL ratios	Percentage	23617	23917
L_LDL_TG_pct	Triglycerides to Total Lipids in Large LDL percentage	%	Relative lipoprotein lipid concentrations	Large LDL ratios	Percentage	23618	23918
M_LDL_PL_pct	Phospholipids to Total Lipids in Medium LDL percentage	%	Relative lipoprotein lipid concentrations	Medium LDL ratios	Percentage	23619	23919
M_LDL_C_pct	Cholesterol to Total Lipids in Medium LDL percentage	%	Relative lipoprotein lipid concentrations	Medium LDL ratios	Percentage	23620	23920
M_LDL_CE_pct	Cholesteryl Esters to Total Lipids in Medium LDL percentage	%	Relative lipoprotein lipid concentrations	Medium LDL ratios	Percentage	23621	23921
M_LDL_FC_pct	Free Cholesterol to Total Lipids in Medium LDL percentage	%	Relative lipoprotein lipid concentrations	Medium LDL ratios	Percentage	23622	23922
M_LDL_TG_pct	Triglycerides to Total Lipids in Medium LDL percentage	%	Relative lipoprotein lipid concentrations	Medium LDL ratios	Percentage	23623	23923
S_LDL_PL_pct	Phospholipids to Total Lipids in Small LDL percentage	%	Relative lipoprotein lipid concentrations	Small LDL ratios	Percentage	23624	23924
S_LDL_C_pct	Cholesterol to Total Lipids in Small LDL percentage	%	Relative lipoprotein lipid concentrations	Small LDL ratios	Percentage	23625	23925
S_LDL_CE_pct	Cholesteryl Esters to Total Lipids in Small LDL percentage	%	Relative lipoprotein lipid concentrations	Small LDL ratios	Percentage	23626	23926
S_LDL_FC_pct	Free Cholesterol to Total Lipids in Small LDL percentage	%	Relative lipoprotein lipid concentrations	Small LDL ratios	Percentage	23627	23927

S_LDL_TG_pct	Triglycerides to Total Lipids in Small LDL percentage	%	Relative lipoprotein lipid concentrations	Small LDL ratios	Percentag e	23628	23928
XL_HDL_PL_pct	Phospholipids to Total Lipids in Very Large HDL percentage	%	Relative lipoprotein lipid concentrations	Very large HDL ratios	Percentag e	23629	23929
XL_HDL_C_pct	Cholesterol to Total Lipids in Very Large HDL percentage	%	Relative lipoprotein lipid concentrations	Very large HDL ratios	Percentag e	23630	23930
XL_HDL_CE_pct	Cholesteryl Esters to Total Lipids in Very Large HDL percentage	%	Relative lipoprotein lipid concentrations	Very large HDL ratios	Percentag e	23631	23931
XL_HDL_FC_pct	Free Cholesterol to Total Lipids in Very Large HDL percentage	%	Relative lipoprotein lipid concentrations	Very large HDL ratios	Percentag e	23632	23932
XL_HDL_TG_pct	Triglycerides to Total Lipids in Very Large HDL percentage	%	Relative lipoprotein lipid concentrations	Very large HDL ratios	Percentag e	23633	23933
L_HDL_PL_pct	Phospholipids to Total Lipids in Large HDL percentage	%	Relative lipoprotein lipid concentrations	Large HDL ratios	Percentag e	23634	23934
L_HDL_C_pct	Cholesterol to Total Lipids in Large HDL percentage	%	Relative lipoprotein lipid concentrations	Large HDL ratios	Percentag e	23635	23935
L_HDL_CE_pct	Cholesteryl Esters to Total Lipids in Large HDL percentage	%	Relative lipoprotein lipid concentrations	Large HDL ratios	Percentag e	23636	23936
L_HDL_FC_pct	Free Cholesterol to Total Lipids in Large HDL percentage	%	Relative lipoprotein lipid concentrations	Large HDL ratios	Percentag e	23637	23937
L_HDL_TG_pct	Triglycerides to Total Lipids in Large HDL percentage	%	Relative lipoprotein lipid concentrations	Large HDL ratios	Percentag e	23638	23938
M_HDL_PL_pct	Phospholipids to Total Lipids in Medium HDL percentage	%	Relative lipoprotein lipid concentrations	Medium HDL ratios	Percentag e	23639	23939
M_HDL_C_pct	Cholesterol to Total Lipids in Medium HDL percentage	%	Relative lipoprotein lipid concentrations	Medium HDL ratios	Percentag e	23640	23940
M_HDL_CE_pct	Cholesteryl Esters to Total Lipids in Medium HDL percentage	%	Relative lipoprotein lipid concentrations	Medium HDL ratios	Percentag e	23641	23941
M_HDL_FC_pct	Free Cholesterol to Total Lipids in Medium HDL percentage	%	Relative lipoprotein lipid concentrations	Medium HDL ratios	Percentag e	23642	23942
M_HDL_TG_pct	Triglycerides to Total Lipids in Medium HDL percentage	%	Relative lipoprotein lipid concentrations	Medium HDL ratios	Percentag e	23643	23943
S_HDL_PL_pct	Phospholipids to Total Lipids in Small HDL percentage	%	Relative lipoprotein lipid concentrations	Small HDL ratios	Percentag e	23644	23944
S_HDL_C_pct	Cholesterol to Total Lipids in Small HDL percentage	%	Relative lipoprotein lipid concentrations	Small HDL ratios	Percentag e	23645	23945
S_HDL_CE_pct	Cholesteryl Esters to Total Lipids in Small HDL percentage	%	Relative lipoprotein lipid concentrations	Small HDL ratios	Percentag e	23646	23946
S_HDL_FC_pct	Free Cholesterol to Total Lipids in Small HDL percentage	%	Relative lipoprotein lipid concentrations	Small HDL ratios	Percentag e	23647	23947
S_HDL_TG_pct	Triglycerides to Total Lipids in Small HDL percentage	%	Relative lipoprotein lipid concentrations	Small HDL ratios	Percentag e	23648	23948

HDL_C_pct	Cholesterol to Total Lipids in HDL percentage	%	Relative lipid concentrations		Percentag e
HDL_CE_pct	Cholesteryl Esters to Total Lipids in HDL percentage	%	Relative lipid concentrations		Percentag e
HDL_CE_pct_C	Cholesteryl Esters to Cholesterol in HDL percentage	%	Relative cholesterol concentrations		Percentag e
HDL_FC_by_CE	Free Cholesterol to Cholesteryl Esters in HDL percentage	ratio	Relative lipid concentrations		Ratio
HDL_FC_pct	Free Cholesterol to Total Lipids in HDL percentage	%	Relative lipid concentrations		Percentag e
HDL_FC_pct_C	Free Cholesterol to Cholesterol in HDL percentage	%	Relative cholesterol concentrations		Percentag e
HDL_PL_pct	Phospholipids to Total Lipids in HDL percentage	%	Relative lipid concentrations		Percentag e
HDL_TG_pct	Triglycerides to Total Lipids in HDL percentage	%	Relative lipid concentrations		Percentag e
IDL_CE_pct_C	Cholesteryl Esters to Cholesterol in IDL percentage	%	Relative lipoprotein cholesterol concentrations	IDL ratios	Percentag e
IDL_FC_by_CE	Free Cholesterol to Cholesteryl Esters in IDL percentage	ratio	Relative lipoprotein lipid concentrations	IDL ratios	Ratio
IDL_FC_pct_C	Free Cholesterol to Cholesterol in IDL percentage	%	Relative lipoprotein cholesterol concentrations	IDL ratios	Percentag e
L_HDL_CE_pct_ C	Cholesteryl Esters to Cholesterol in Large HDL percentage	%	Relative lipoprotein cholesterol concentrations	Large HDL ratios	Percentag e
L_HDL_FC_by_C E	Free Cholesterol to Cholesteryl Esters in Large HDL ratio	ratio	Relative lipoprotein lipid concentrations	Large HDL ratios	Ratio
L_HDL_FC_pct_ C	Free Cholesterol to Cholesterol in Large HDL percentage	%	Relative lipoprotein cholesterol concentrations	Large HDL ratios	Percentag e
L_LDL_CE_pct_C	Cholesteryl Esters to Cholesterol in Large LDL percentage	%	Relative lipoprotein cholesterol concentrations	Large LDL ratios	Percentag e
L_LDL_FC_by_C E	Free Cholesterol to Cholesteryl Esters in Large LDL ratio	ratio	Relative lipoprotein lipid concentrations	Large LDL ratios	Ratio
L_LDL_FC_pct_C	Free Cholesterol to Cholesterol in Large LDL percentage	%	Relative lipoprotein cholesterol concentrations	Large LDL ratios	Percentag e
L_VLDL_CE_pct _C	Cholesteryl Esters to Cholesterol in Large VLDL percentage	%	Relative lipoprotein cholesterol concentrations	Large VLDL ratios	Percentag e
L_VLDL_FC_by_ CE	Free Cholesterol to Cholesteryl Esters in Large VLDL ratio	ratio	Relative lipoprotein lipid concentrations	Large VLDL ratios	Ratio
L_VLDL_FC_pct_ C	Free Cholesterol to Cholesterol in Large VLDL percentage	%	Relative lipoprotein cholesterol concentrations	Large VLDL ratios	Percentag e
LDL_C_pct	Cholesterol to Total Lipids in LDL percentage	%	Relative lipid concentrations		Percentag e

LDL_CE_pct	Cholesteryl Esters to Total Lipids in LDL percentage	%	Relative lipid concentrations		Percentag e
LDL_CE_pct_C	Cholesteryl Esters to Cholesterol in LDL percentage	%	Relative cholesterol concentrations		Percentag e
LDL_FC_by_CE	Free Cholesterol to Cholesteryl Esters in LDL percentage	ratio	Relative lipid concentrations		Ratio
LDL_FC_pct	Free Cholesterol to Total Lipids in LDL percentage	%	Relative lipid concentrations		Percentag e
LDL_FC_pct_C	Free Cholesterol to Cholesterol in LDL percentage	%	Relative cholesterol concentrations		Percentag e
LDL_PL_pct	Phospholipids to Total Lipids in LDL percentage	%	Relative lipid concentrations		Percentag e
LDL_TG_pct	Triglycerides to Total Lipids in LDL percentage	%	Relative lipid concentrations		Percentag e
M_HDL_CE_pct_ C	Cholesteryl Esters to Cholesterol in Medium HDL percentage	%	Relative lipoprotein cholesterol concentrations	Medium HDL ratios	Percentag e
M_HDL_FC_by_ CE	Free Cholesterol to Cholesteryl Esters in Medium HDL ratio	ratio	Relative lipoprotein lipid concentrations	Medium HDL ratios	Ratio
M_HDL_FC_pct_ C	Free Cholesterol to Cholesterol in Medium HDL percentage	%	Relative lipoprotein cholesterol concentrations	Medium HDL ratios	Percentag e
M_LDL_CE_pct_ C	Cholesteryl Esters to Cholesterol in Medium LDL percentage	%	Relative lipoprotein cholesterol concentrations	Medium LDL ratios	Percentag e
M_LDL_FC_by_C E	Free Cholesterol to Cholesteryl Esters in Medium LDL ratio	ratio	Relative lipoprotein lipid concentrations	Medium LDL ratios	Ratio
M_LDL_FC_pct_ C	Free Cholesterol to Cholesterol in Medium LDL percentage	%	Relative lipoprotein cholesterol concentrations	Medium LDL ratios	Percentag e
M_VLDL_CE_pct _C	Cholesteryl Esters to Cholesterol in Medium VLDL percentage	%	Relative lipoprotein cholesterol concentrations	Medium VLDL ratios	Percentag e
M_VLDL_FC_by _CE	Free Cholesterol to Cholesteryl Esters in Medium VLDL ratio	ratio	Relative lipoprotein lipid concentrations	Medium VLDL ratios	Ratio
M_VLDL_FC_pct _C	Free Cholesterol to Cholesterol in Medium VLDL percentage	%	Relative lipoprotein cholesterol concentrations	Medium VLDL ratios	Percentag e
Omega_3_pct_PU FA	Omega-3 Fatty Acids to Polyunsaturated Fatty Acids percentage	%	Fatty acids	Fatty acid ratios	Percentag e
Omega_6_pct_PU FA	Omega-6 Fatty Acids to Polyunsaturated Fatty Acids percentage	%	Fatty acids	Fatty acid ratios	Percentag e
S_HDL_CE_pct_ C	Cholesteryl Esters to Cholesterol in Small HDL percentage	%	Relative lipoprotein cholesterol concentrations	Small HDL ratios	Percentag e
S_HDL_FC_by_C E	Free Cholesterol to Cholesteryl Esters in Small HDL ratio	ratio	Relative lipoprotein lipid concentrations	Small HDL ratios	Ratio
S_HDL_FC_pct_C	Free Cholesterol to Cholesterol in Small HDL percentage	%	Relative lipoprotein cholesterol concentrations	Small HDL ratios	Percentag e

S_LDL_CE_pct_C	Cholesteryl Esters to Cholesterol in Small LDL percentage	%	Relative lipoprotein cholesterol concentrations	Small LDL ratios	Percentag e
S_LDL_FC_by_C E	Free Cholesterol to Cholesteryl Esters in Small LDL ratio	ratio	Relative lipoprotein lipid concentrations	Small LDL ratios	Ratio
S_LDL_FC_pct_C	Free Cholesterol to Cholesterol in Small LDL percentage	%	Relative lipoprotein cholesterol concentrations	Small LDL ratios	Percentag e
S_VLDL_CE_pct_ C	Cholesteryl Esters to Cholesterol in Small VLDL percentage	%	Relative lipoprotein cholesterol concentrations	Small VLDL ratios	Percentag e
S_VLDL_FC_by_ CE	Free Cholesterol to Cholesteryl Esters in Small VLDL ratio	ratio	Relative lipoprotein lipid concentrations	Small VLDL ratios	Ratio
S_VLDL_FC_pct_ C	Free Cholesterol to Cholesterol in Small VLDL percentage	%	Relative lipoprotein cholesterol concentrations	Small VLDL ratios	Percentag e
Total_C_pct	Total Cholesterol to Total Lipids percentage	%	Relative lipid concentrations		Percentag e
Total_CE_pct	Total Esterified Cholesterol to Total Lipids percentage	%	Relative lipid concentrations		Percentag e
Total_CE_pct_C	Total Esterified Cholesterol to Total Cholesterol percentage	%	Relative cholesterol concentrations		Percentag e
Total_FC_by_CE	Total Free Cholesterol to Total Esterified Cholesterol ratio	ratio	Relative lipid concentrations		Ratio
Total_FC_pct	Total Free Cholesterol to Total Lipids percentage	%	Relative lipid concentrations		Percentag e
Total_FC_pct_C	Total Free Cholesterol to Total Cholesterol percentage	%	Relative cholesterol concentrations		Percentag e
Total_PL_pct	Total Phospholipids to Total Lipids percentage	%	Relative lipid concentrations		Percentag e
Total_TG_pct	Total Triglycerides to Total Lipids percentage	%	Relative lipid concentrations		Percentag e
VLDL_C_pct	Cholesterol to Total Lipids in VLDL percentage	%	Relative lipid concentrations		Percentag e
VLDL_CE_pct	Cholesteryl Esters to Total Lipids in VLDL percentage	%	Relative lipid concentrations		Percentag e
VLDL_CE_pct_C	Cholesteryl Esters to Cholesterol in VLDL percentage	%	Relative cholesterol concentrations		Percentag e
VLDL_FC_by_CE	Free Cholesterol to Cholesteryl Esters in VLDL percentage	ratio	Relative lipid concentrations		Ratio
VLDL_FC_pct	Free Cholesterol to Total Lipids in VLDL percentage	%	Relative lipid concentrations		Percentag e
VLDL_FC_pct_C	Free Cholesterol to Cholesterol in VLDL percentage	%	Relative cholesterol concentrations		Percentag e
VLDL_PL_pct	Phospholipids to Total Lipids in VLDL percentage	%	Relative lipid concentrations		Percentag e

VLDL_TG_pct	Triglycerides to Total Lipids in VLDL percentage	%	Relative lipid concentrations		Percentage
XL_HDL_CE_pct_C	Cholesteryl Esters to Cholesterol in Very Large HDL percentage	%	Relative lipoprotein cholesterol concentrations	Very large HDL ratios	Percentage
XL_HDL_FC_by_CE	Free Cholesterol to Cholesteryl Esters in Very Large HDL ratio	ratio	Relative lipoprotein lipid concentrations	Very large HDL ratios	Ratio
XL_HDL_FC_pct_C	Free Cholesterol to Cholesterol in Very Large HDL percentage	%	Relative lipoprotein cholesterol concentrations	Very large HDL ratios	Percentage
XL_VLDL_CE_pct_C	Cholesteryl Esters to Cholesterol in Very Large VLDL percentage	%	Relative lipoprotein cholesterol concentrations	Very large VLDL ratios	Percentage
XL_VLDL_FC_by_CE	Free Cholesterol to Cholesteryl Esters in Very Large VLDL ratio	ratio	Relative lipoprotein lipid concentrations	Very large VLDL ratios	Ratio
XL_VLDL_FC_pct_C	Free Cholesterol to Cholesterol in Very Large VLDL percentage	%	Relative lipoprotein cholesterol concentrations	Very large VLDL ratios	Percentage
XS_VLDL_CE_pct_C	Cholesteryl Esters to Cholesterol in Very Small VLDL percentage	%	Relative lipoprotein cholesterol concentrations	Very small VLDL ratios	Percentage
XS_VLDL_FC_by_CE	Free Cholesterol to Cholesteryl Esters in Very Small VLDL ratio	ratio	Relative lipoprotein lipid concentrations	Very small VLDL ratios	Ratio
XS_VLDL_FC_pct_C	Free Cholesterol to Cholesterol in Very Small VLDL percentage	%	Relative lipoprotein cholesterol concentrations	Very small VLDL ratios	Percentage
XXL_VLDL_CE_pct_C	Cholesteryl Esters to Cholesterol in Chylomicrons and Extremely Large VLDL percentage	%	Relative lipoprotein cholesterol concentrations	Chylomicrons and extremely large VLDL ratios	Percentage
XXL_VLDL_FC_by_CE	Free Cholesterol to Cholesteryl Esters in Chylomicrons and Extremely Large VLDL ratio	ratio	Relative lipoprotein lipid concentrations	Chylomicrons and extremely large VLDL ratios	Ratio
XXL_VLDL_FC_pct_C	Free Cholesterol to Cholesterol in Chylomicrons and Extremely Large VLDL percentage	%	Relative lipoprotein cholesterol concentrations	Chylomicrons and extremely large VLDL ratios	Percentage

Table S5. Statistical power summary (Events Per Variable, EPV) for all CVD models

ICD-10 code	Disease	Case Counts	Predictor Count	EPV
G45	Transient cerebral ischaemic attacks and related syndromes	584	325	1.796923077
G45.9	Transient cerebral ischaemic attack, unspecified	480	325	1.476923077
I05	Rheumatic mitral valve diseases	90	325	0.276923077
I08	Multiple valve diseases	198	325	0.609230769
I10	Essential (primary) hypertension	16814	325	51.73538462
I12	Hypertensive renal disease	322	325	0.990769231
I12.0	Hypertensive renal disease with renal failure	287	325	0.883076923
I20	Angina pectoris	5562	325	17.11384615
I20.0	Unstable angina	1794	325	5.52
I20.8	Other forms of angina pectoris	344	325	1.058461538
I20.9	Angina pectoris, unspecified	4495	325	13.83076923
I21	Acute myocardial infarction	2383	325	7.332307692
I21.0	Acute transmural myocardial infarction of anterior wall	576	325	1.772307692
I21.1	Acute transmural myocardial infarction of inferior wall	740	325	2.276923077
I21.2	Acute transmural myocardial infarction of other sites	97	325	0.298461538
I21.4	Acute subendocardial myocardial infarction	304	325	0.935384615
I21.9	Acute myocardial infarction, unspecified	830	325	2.553846154
I22	Subsequent myocardial infarction	238	325	0.732307692
I22.9	Subsequent myocardial infarction of unspecified site	123	325	0.378461538
I24	Other acute ischaemic heart diseases	190	325	0.584615385
I24.8	Other forms of acute ischaemic heart disease	95	325	0.292307692
I25	Chronic ischaemic heart disease	7005	325	21.55384615
I25.1	Atherosclerotic heart disease	5134	325	15.79692308
I25.2	Old myocardial infarction	1025	325	3.153846154
I25.8	Other forms of chronic ischaemic heart disease	1979	325	6.089230769
I25.9	Chronic ischaemic heart disease, unspecified	2214	325	6.812307692
I26	Pulmonary embolism	671	325	2.064615385
I26.9	Pulmonary embolism without mention of acute cor pulmonale	667	325	2.052307692
I27	Other pulmonary heart diseases	95	325	0.292307692
I31	Other diseases of pericardium	190	325	0.584615385
I31.9	Disease of pericardium, unspecified	109	325	0.335384615
I34	Nonrheumatic mitral valve disorders	468	325	1.44
I34.0	Mitral (valve) insufficiency	388	325	1.193846154
I34.1	Mitral (valve) prolapse	133	325	0.409230769
I35	Nonrheumatic aortic valve disorders	405	325	1.246153846
I35.0	Aortic (valve) stenosis	234	325	0.72
I35.1	Aortic (valve) insufficiency	148	325	0.455384615
I42	Cardiomyopathy	254	325	0.781538462
I42.0	Dilated cardiomyopathy	145	325	0.446153846
I42.9	Cardiomyopathy, unspecified	92	325	0.283076923
I44	Atrioventricular and left bundle-branch block	500	325	1.538461538
I44.0	Atrioventricular block, first degree	97	325	0.298461538
I44.2	Atrioventricular block, complete	97	325	0.298461538
I44.7	Left bundle-branch block, unspecified	225	325	0.692307692
I45	Other conduction disorders	364	325	1.12
I45.1	Other and unspecified right bundle-branch block	213	325	0.655384615
I46	Cardiac arrest	98	325	0.301538462

I47	Paroxysmal tachycardia	819	325	2.52
I47.1	Supraventricular tachycardia	606	325	1.864615385
I47.2	Ventricular tachycardia	189	325	0.581538462
I48	Atrial fibrillation and flutter	2782	325	8.56
I49	Other cardiac arrhythmias	504	325	1.550769231
I49.8	Other specified cardiac arrhythmias	99	325	0.304615385
I49.9	Cardiac arrhythmia, unspecified	197	325	0.606153846
I50	Heart failure	952	325	2.929230769
I50.0	Congestive heart failure	280	325	0.861538462
I50.1	Left ventricular failure	671	325	2.064615385
I50.9	Heart failure, unspecified	139	325	0.427692308
I51	Complications and ill-defined descriptions of heart disease	546	325	1.68
I51.7	Cardiomegaly	363	325	1.116923077
I51.8	Other ill-defined heart diseases	101	325	0.310769231
I60	Subarachnoid haemorrhage	244	325	0.750769231
I60.9	Subarachnoid haemorrhage, unspecified	149	325	0.458461538
I61	Intracerebral haemorrhage	132	325	0.406153846
I61.9	Intracerebral haemorrhage, unspecified	86	325	0.264615385
I63	Cerebral infarction	532	325	1.636923077
I63.9	Cerebral infarction, unspecified	444	325	1.366153846
I64	Stroke, not specified as haemorrhage or infarction	298	325	0.916923077
I65	Occlusion and stenosis of precerebral arteries, not resulting in cerebral infarction	206	325	0.633846154
I65.2	Occlusion and stenosis of carotid artery	195	325	0.6
I67	Other cerebrovascular diseases	315	325	0.969230769
I67.9	Cerebrovascular disease, unspecified	117	325	0.36
I69	Sequelae of cerebrovascular disease	255	325	0.784615385
I69.4	Sequelae of stroke, not specified as haemorrhage or infarction	153	325	0.470769231
I70	Atherosclerosis	245	325	0.753846154
I70.2	Atherosclerosis of arteries of the extremities	150	325	0.461538462
I71	Aortic aneurysm and dissection	185	325	0.569230769
I71.4	Abdominal aortic aneurysm, without mention of rupture	88	325	0.270769231
I73	Other peripheral vascular diseases	804	325	2.473846154
I73.0	Raynaud's syndrome	196	325	0.603076923
I73.9	Peripheral vascular disease, unspecified	606	325	1.864615385
I74	Arterial embolism and thrombosis	203	325	0.624615385
I74.3	Embolism and thrombosis of arteries of the lower extremities	110	325	0.338461538
I77	Other disorders of arteries and arterioles	324	325	0.996923077
I77.1	Stricture of artery	181	325	0.556923077
I78	Diseases of capillaries	89	325	0.273846154
I78.1	Naevus, nonneoplastic	67	325	0.206153846

Table S6. Discrimination performance of machine learning models based on metabolomic and clinical data integration

a. Based on metabolomic data

	**LR**							**XGBoost**							**RF**						
**Disease**	**AUC (95% CI)**	**Acc (95% CI)**	**Sn (95% CI)**	**Sp (95% CI)**	**PPV (95% CI)**	**NPV (95% CI)**	**F1 score (95% CI)**	**AUC (95% CI)**	**Acc (95% CI)**	**Sn (95% CI)**	**Sp (95% CI)**	**PPV (95% CI)**	**NPV (95% CI)**	**F1 score (95% CI)**	**AUC (95% CI)**	**Acc (95% CI)**	**Sn (95% CI)**	**Sp (95% CI)**	**PPV (95% CI)**	**NPV (95% CI)**	**F1 score (95% CI)**
Essential (primary) hypertension	0.7747 (0.7707 - 0.7781)	0.7113 (0.7079 - 0.7150)	0.6874 (0.6629 - 0.7102)	0.7351 (0.7131 - 0.7600)	0.7219 (0.7112 - 0.7343)	0.7016 (0.6927 - 0.7129)	0.7042 (0.6965 - 0.7127)	0.7568 (0.7530 - 0.7609)	0.6939 (0.6904 - 0.6980)	0.6756 (0.6561 - 0.6834)	0.7122 (0.7082 - 0.7364)	0.7013 (0.6968 - 0.7105)	0.6871 (0.6781 - 0.6936)	0.6882 (0.6801 - 0.6931)	0.7532 (0.7493 - 0.7571)	0.6935 (0.6901 - 0.6974)	0.6584 (0.6226 - 0.6712)	0.7286 (0.7192 - 0.7618)	0.7081 (0.7027 - 0.7254)	0.6808 (0.6682 - 0.6876)	0.6823 (0.6687 - 0.6883)
Chronic ischaemic heart disease	0.8792 (0.8751 - 0.8832)	0.8163 (0.8125 - 0.8209)	0.8128 (0.8058 - 0.8433)	0.8198 (0.7918 - 0.8242)	0.8186 (0.8003 - 0.8228)	0.8141 (0.8082 - 0.8358)	0.8157 (0.8116 - 0.8244)	0.8647 (0.8603 - 0.8688)	0.7953 (0.7908 - 0.8005)	0.7973 (0.7693 - 0.8191)	0.7933 (0.7732 - 0.8185)	0.7941 (0.7813 - 0.8117)	0.7965 (0.7788 - 0.8096)	0.7957 (0.7876 - 0.8023)	0.8606 (0.8562 - 0.8648)	0.7974 (0.7931 - 0.8022)	0.7873 (0.7738 - 0.8053)	0.8075 (0.7906 - 0.8163)	0.8035 (0.7923 - 0.8109)	0.7915 (0.7814 - 0.8031)	0.7953 (0.7893 - 0.8016)
Angina pectoris	0.8457 (0.8407 - 0.8517)	0.7846 (0.7802 - 0.7909)	0.8003 (0.7542 - 0.8112)	0.7690 (0.7624 - 0.8179)	0.7760 (0.7710 - 0.8049)	0.7938 (0.7685 - 0.8035)	0.7880 (0.7765 - 0.7949)	0.8318 (0.8264 - 0.8370)	0.7651 (0.7601 - 0.7707)	0.7675 (0.7496 - 0.7842)	0.7626 (0.7518 - 0.7767)	0.7638 (0.7557 - 0.7739)	0.7664 (0.7550 - 0.7787)	0.7657 (0.7584 - 0.7739)	0.8274 (0.8221 - 0.8328)	0.7661 (0.7610 - 0.7722)	0.7478 (0.7327 - 0.7831)	0.7844 (0.7541 - 0.7954)	0.7762 (0.7573 - 0.7858)	0.7567 (0.7468 - 0.7778)	0.7617 (0.7548 - 0.7735)
Atrial fibrillation and flutter	0.7737 (0.7640 - 0.7832)	0.7088 (0.7014 - 0.7195)	0.6909 (0.6027 - 0.7326)	0.7266 (0.6814 - 0.8122)	0.7165 (0.6966 - 0.7671)	0.7015 (0.6686 - 0.7233)	0.7035 (0.6709 - 0.7195)	0.7562 (0.7468 - 0.7654)	0.6921 (0.6848 - 0.7017)	0.6675 (0.5786 - 0.7195)	0.7168 (0.6735 - 0.8066)	0.7021 (0.6802 - 0.7515)	0.6831 (0.6562 - 0.7068)	0.6844 (0.6532 - 0.7023)	0.7335 (0.7239 - 0.7427)	0.6779 (0.6711 - 0.6888)	0.6168 (0.5798 - 0.7025)	0.7389 (0.6618 - 0.7726)	0.7026 (0.6682 - 0.7243)	0.6585 (0.6442 - 0.6915)	0.6569 (0.6405 - 0.6898)
Acute myocardial infarction	0.8980 (0.8925 - 0.9036)	0.8341 (0.8274 - 0.8407)	0.8728 (0.8515 - 0.8890)	0.7953 (0.7766 - 0.8130)	0.8100 (0.7987 - 0.8223)	0.8622 (0.8449 - 0.8782)	0.8403 (0.8321 - 0.8484)	0.8884 (0.8823 - 0.8949)	0.8181 (0.8123 - 0.8271)	0.8183 (0.8062 - 0.8864)	0.8179 (0.7487 - 0.8310)	0.8180 (0.7782 - 0.8282)	0.8182 (0.8091 - 0.8691)	0.8181 (0.8128 - 0.8340)	0.8827 (0.8765 - 0.8889)	0.8212 (0.8154 - 0.8288)	0.8716 (0.8380 - 0.8870)	0.7707 (0.7625 - 0.8030)	0.7917 (0.7838 - 0.8124)	0.8572 (0.8319 - 0.8722)	0.8298 (0.8208 - 0.8379)
Heart failure	0.8583 (0.84	0.7933 (0.78	0.8183 (0.73	0.7683 (0.75	0.7793 (0.76	0.8087 (0.75	0.7983 (0.77	0.8488 (0.83	0.7752 (0.76	0.7794 (0.71	0.7711 (0.69	0.7730 (0.73	0.7776 (0.74	0.7762 (0.75	0.8279 (0.81	0.7662 (0.75	0.8078 (0.73	0.7246 (0.71	0.7458 (0.73	0.7903 (0.74	0.7755 (0.75

	53 - 0.8702)	14 - 0.8058)	06 - 0.8464)	58 - 0.8566)	43 - 0.8356)	75 - 0.8333)	65 - 0.8145)	56 - 0.8607)	28 - 0.7896)	47 - 0.8487)	84 - 0.8351)	71 - 0.8146)	33 - 0.8203)	58 - 0.7949)	57 - 0.8415)	62 - 0.7814)	60 - 0.8312)	25 - 0.7897)	25 - 0.7870)	52 - 0.8121)	31 - 0.7903)
Paroxysmal tachycardia	0.6345 (0.6147 - 0.6523)	0.6058 (0.5920 - 0.6233)	0.4603 (0.3885 - 0.7410)	0.7514 (0.4764 - 0.8195)	0.6493 (0.5756 - 0.6912)	0.5820 (0.5627 - 0.6545)	0.5387 (0.4919 - 0.6583)	0.6407 (0.6209 - 0.6611)	0.6114 (0.5960 - 0.6298)	0.5311 (0.4438 - 0.6019)	0.6917 (0.6239 - 0.7756)	0.6328 (0.6030 - 0.6665)	0.5960 (0.5721 - 0.6237)	0.5775 (0.5295 - 0.6147)	0.6183 (0.5983 - 0.6377)	0.5882 (0.5751 - 0.6078)	0.4457 (0.3893 - 0.6359)	0.7308 (0.5450 - 0.7823)	0.6234 (0.5790 - 0.6539)	0.5687 (0.5495 - 0.6036)	0.5198 (0.4798 - 0.6078)
Other peripheral vascular diseases	0.8019 (0.7870 - 0.8195)	0.7424 (0.7296 - 0.7596)	0.6953 (0.6470 - 0.7657)	0.7895 (0.7361 - 0.8366)	0.7676 (0.7304 - 0.8004)	0.7215 (0.6959 - 0.7596)	0.7296 (0.7078 - 0.7566)	0.7969 (0.7806 - 0.8116)	0.7386 (0.7234 - 0.7534)	0.7015 (0.6473 - 0.7351)	0.7757 (0.7603 - 0.8225)	0.7577 (0.7389 - 0.7906)	0.7221 (0.6931 - 0.7472)	0.7285 (0.7030 - 0.7489)	0.7697 (0.7526 - 0.7860)	0.7124 (0.6995 - 0.7299)	0.7289 (0.6183 - 0.7706)	0.6959 (0.6734 - 0.8001)	0.7056 (0.6859 - 0.7643)	0.7196 (0.6723 - 0.7495)	0.7170 (0.6770 - 0.7396)
Pulmonary embolism	0.6642 (0.6422 - 0.6855)	0.6301 (0.6121 - 0.6500)	0.5827 (0.5359 - 0.6820)	0.6775 (0.5728 - 0.7106)	0.6437 (0.6079 - 0.6693)	0.6188 (0.5945 - 0.6583)	0.6117 (0.5790 - 0.6519)	0.6691 (0.6488 - 0.6899)	0.6304 (0.6153 - 0.6483)	0.7362 (0.6369 - 0.8370)	0.5245 (0.4232 - 0.6179)	0.6076 (0.5826 - 0.6381)	0.6654 (0.6222 - 0.7245)	0.6658 (0.6304 - 0.6957)	0.6192 (0.5985 - 0.6406)	0.5895 (0.5751 - 0.6092)	0.7392 (0.4812 - 0.8249)	0.4398 (0.3457 - 0.6913)	0.5689 (0.5498 - 0.6239)	0.6277 (0.5635 - 0.6737)	0.6429 (0.5331 - 0.6696)
Transient cerebral ischaemic attacks and related syndromes	0.7264 (0.7037 - 0.7478)	0.6817 (0.6651 - 0.7026)	0.6541 (0.5765 - 0.6939)	0.7093 (0.6809 - 0.7840)	0.6923 (0.6747 - 0.7338)	0.6722 (0.6404 - 0.7028)	0.6727 (0.6352 - 0.6999)	0.7346 (0.7139 - 0.7553)	0.6913 (0.6747 - 0.7114)	0.6421 (0.5736 - 0.6813)	0.7406 (0.7204 - 0.8079)	0.7122 (0.6918 - 0.7527)	0.6742 (0.6424 - 0.7033)	0.6754 (0.6425 - 0.7017)	0.7060 (0.6831 - 0.7291)	0.6743 (0.6567 - 0.6966)	0.5873 (0.5341 - 0.6795)	0.7612 (0.6873 - 0.8225)	0.7110 (0.6677 - 0.7440)	0.6485 (0.6226 - 0.6915)	0.6433 (0.6117 - 0.6881)
Complications and ill-defined descriptions of heart disease	0.7358 (0.7112 - 0.7603)	0.7027 (0.6849 - 0.7257)	0.6264 (0.5861 - 0.7074)	0.7791 (0.6896 - 0.7986)	0.7393 (0.6932 - 0.7626)	0.6759 (0.6489 - 0.7141)	0.6782 (0.6509 - 0.7124)	0.7575 (0.7351 - 0.7782)	0.6973 (0.6816 - 0.7211)	0.7161 (0.5349 - 0.7557)	0.6784 (0.6571 - 0.8546)	0.6901 (0.6706 - 0.7913)	0.7050 (0.6415 - 0.7373)	0.7029 (0.6369 - 0.7265)	0.7331 (0.7100 - 0.7555)	0.6937 (0.6768 - 0.7153)	0.6795 (0.6075 - 0.7176)	0.7080 (0.6968 - 0.7662)	0.6994 (0.6813 - 0.7364)	0.6884 (0.6510 - 0.7191)	0.6893 (0.6544 - 0.7149)
Cerebral infarction	0.7989 (0.7790 - 0.8190)	0.7424 (0.7268 - 0.7626)	0.7293 (0.6691 - 0.7860)	0.7554 (0.7248 - 0.8078)	0.7489 (0.7218 - 0.7828)	0.7362 (0.7028 - 0.7773)	0.7390 (0.7125 - 0.7661)	0.8066 (0.7894 - 0.8244)	0.7406 (0.7244 - 0.7601)	0.7782 (0.6804 - 0.8218)	0.7030 (0.6791 - 0.7910)	0.7238 (0.7018 - 0.7743)	0.7602 (0.7071 - 0.7958)	0.7500 (0.7177 - 0.7725)	0.7808 (0.7608 - 0.7998)	0.7176 (0.7024 - 0.7392)	0.7274 (0.6441 - 0.8045)	0.7078 (0.6382 - 0.7732)	0.7134 (0.6772 - 0.7596)	0.7220 (0.6851 - 0.7699)	0.7204 (0.6862 - 0.7503)
Other cardiac arrhythmias	0.6510 (0.6252 -	0.6246 (0.6069 -	0.5853 (0.4148 -	0.6639 (0.6041 -	0.6352 (0.6060 -	0.6155 (0.5786 -	0.6093 (0.5200 -	0.6658 (0.6398 -	0.6304 (0.6141 -	0.5357 (0.4637 -	0.7252 (0.5553 -	0.6609 (0.6024 -	0.6097 (0.5853 -	0.5918 (0.5496 -	0.6345 (0.6085 -	0.6155 (0.5969 -	0.5833 (0.4676 -	0.6476 (0.6076 -	0.6234 (0.6006 -	0.6085 (0.5748 -	0.6027 (0.5465 -

	0.6761)	0.6467)	0.6569)	0.8268)	0.7153)	0.6520)	0.6441)	0.6907)	0.6543)	0.7228)	0.7839)	0.7037)	0.6735)	0.6674)	0.6594)	0.6372)	0.6383)	0.7475)	0.6715)	0.6429)	0.6340)
Atrioventricular and left bundle-branch block	0.6992 (0.6740 - 0.7255)	0.6660 (0.6505 - 0.6925)	0.5260 (0.4928 - 0.7010)	0.8061 (0.6552 - 0.8275)	0.7306 (0.6530 - 0.7612)	0.6297 (0.6107 - 0.6941)	0.6116 (0.5892 - 0.6897)	0.7183 (0.6944 - 0.7445)	0.6815 (0.6623 - 0.7058)	0.5840 (0.5287 - 0.6793)	0.7790 (0.6820 - 0.8162)	0.7255 (0.6745 - 0.7588)	0.6519 (0.6232 - 0.6925)	0.6471 (0.6108 - 0.6881)	0.7014 (0.6752 - 0.7277)	0.6690 (0.6498 - 0.6927)	0.6060 (0.5246 - 0.6890)	0.7319 (0.6757 - 0.8157)	0.6933 (0.6546 - 0.7391)	0.6501 (0.6208 - 0.6929)	0.6467 (0.6080 - 0.6873)
Nonrheumatic mitral valve disorders	0.6826 (0.6560 - 0.7094)	0.6475 (0.6274 - 0.6746)	0.5107 (0.4346 - 0.5720)	0.7843 (0.7177 - 0.8360)	0.7031 (0.6743 - 0.7533)	0.6158 (0.5863 - 0.6496)	0.5916 (0.5411 - 0.6331)	0.6852 (0.6584 - 0.7109)	0.6425 (0.6231 - 0.6674)	0.5855 (0.4978 - 0.6816)	0.6995 (0.6204 - 0.7705)	0.6608 (0.6232 - 0.7053)	0.6279 (0.5972 - 0.6734)	0.6209 (0.5753 - 0.6677)	0.6530 (0.6258 - 0.6774)	0.6173 (0.5995 - 0.6416)	0.5449 (0.4889 - 0.7061)	0.6896 (0.5303 - 0.7294)	0.6371 (0.5883 - 0.6712)	0.6024 (0.5781 - 0.6586)	0.5874 (0.5501 - 0.6557)
Nonrheumatic aortic valve disorders	0.7379 (0.7105 - 0.7653)	0.7070 (0.6851 - 0.7339)	0.6049 (0.5505 - 0.6674)	0.8091 (0.7681 - 0.8394)	0.7601 (0.7257 - 0.7875)	0.6719 (0.6407 - 0.7120)	0.6737 (0.6383 - 0.7119)	0.7515 (0.7263 - 0.7760)	0.6962 (0.6760 - 0.7226)	0.6568 (0.5539 - 0.7904)	0.7357 (0.5985 - 0.8204)	0.7130 (0.6604 - 0.7633)	0.6819 (0.6431 - 0.7385)	0.6838 (0.6389 - 0.7238)	0.7264 (0.7012 - 0.7539)	0.6764 (0.6590 - 0.7027)	0.7407 (0.5235 - 0.7854)	0.6120 (0.6005 - 0.8197)	0.6563 (0.6361 - 0.7573)	0.7024 (0.6237 - 0.7443)	0.6959 (0.6130 - 0.7233)
Other conduction disorders	0.6366 (0.6059 - 0.6666)	0.6146 (0.5930 - 0.6435)	0.5110 (0.4531 - 0.6801)	0.7182 (0.5809 - 0.7755)	0.6445 (0.5847 - 0.6836)	0.5949 (0.5670 - 0.6541)	0.5700 (0.5291 - 0.6505)	0.6604 (0.6306 - 0.6906)	0.6275 (0.6065 - 0.6571)	0.5302 (0.3930 - 0.7003)	0.7249 (0.5663 - 0.8387)	0.6584 (0.6003 - 0.7361)	0.6068 (0.5701 - 0.6589)	0.5874 (0.5040 - 0.6589)	0.6205 (0.5916 - 0.6487)	0.6019 (0.5808 - 0.6295)	0.4945 (0.4104 - 0.6128)	0.7092 (0.5881 - 0.7620)	0.6297 (0.5900 - 0.6761)	0.5839 (0.5525 - 0.6225)	0.5540 (0.5013 - 0.6115)
Other cerebrovascular diseases	0.6923 (0.6544 - 0.7237)	0.6582 (0.6367 - 0.6895)	0.5016 (0.4650 - 0.7367)	0.8148 (0.5867 - 0.8321)	0.7303 (0.6302 - 0.7626)	0.6205 (0.5974 - 0.7024)	0.5947 (0.5671 - 0.6930)	0.7188 (0.6902 - 0.7477)	0.6643 (0.6436 - 0.6938)	0.6921 (0.5062 - 0.7394)	0.6365 (0.6219 - 0.8134)	0.6556 (0.6288 - 0.7465)	0.6739 (0.6113 - 0.7145)	0.6733 (0.5943 - 0.7074)	0.6796 (0.6487 - 0.7105)	0.6359 (0.6143 - 0.6676)	0.5302 (0.3883 - 0.7256)	0.7416 (0.5359 - 0.8709)	0.6723 (0.6039 - 0.7605)	0.6122 (0.5769 - 0.6775)	0.5928 (0.5122 - 0.6698)
Other disorders of arteries and arterioles	0.7629 (0.7333 - 0.7923)	0.7255 (0.7062 - 0.7528)	0.7130 (0.6136 - 0.7577)	0.7381 (0.7092 - 0.8253)	0.7314 (0.7101 - 0.7898)	0.7200 (0.6712 - 0.7614)	0.7220 (0.6803 - 0.7498)	0.7889 (0.7622 - 0.8128)	0.7269 (0.7059 - 0.7526)	0.6883 (0.6435 - 0.8300)	0.7655 (0.6543 - 0.7956)	0.7458 (0.6801 - 0.7748)	0.7106 (0.6805 - 0.7945)	0.7159 (0.6880 - 0.7640)	0.7694 (0.7447 - 0.7924)	0.7188 (0.7012 - 0.7457)	0.7809 (0.6677 - 0.8380)	0.6568 (0.6162 - 0.7692)	0.6947 (0.6664 - 0.7505)	0.7498 (0.6897 - 0.8027)	0.7352 (0.6968 - 0.7655)
Hypertensive renal disease	0.8943 (0.8713 -	0.8428 (0.8232 -	0.8075 (0.7213 -	0.8781 (0.8639 -	0.8688 (0.8488 -	0.8202 (0.7689 -	0.8370 (0.8075 -	0.9194 (0.9010 -	0.8500 (0.8314 -	0.7950 (0.7389 -	0.9050 (0.8045 -	0.8932 (0.8172 -	0.8153 (0.7802 -	0.8413 (0.8169 -	0.8954 (0.8749 -	0.8309 (0.8099 -	0.8168 (0.7588 -	0.8451 (0.7949 -	0.8406 (0.8032 -	0.8218 (0.7811 -	0.8285 (0.8012 -

	0.9169)	0.8636)	0.8495)	0.9624)	0.9477)	0.8549)	0.8620)	0.9371)	0.8747)	0.8817)	0.9428)	0.9331)	0.8741)	0.8701)	0.9145)	0.8532)	0.8700)	0.8895)	0.8756)	0.8638)	0.8544)
Stroke, not specified as haemorrhage or infarction	0.7739 (0.7408 - 0.8045)	0.7357 (0.7124 - 0.7644)	0.6913 (0.6268 - 0.7679)	0.7802 (0.7302 - 0.8139)	0.7587 (0.7172 - 0.7943)	0.7165 (0.6806 - 0.7673)	0.7234 (0.6865 - 0.7632)	0.8009 (0.7734 - 0.8257)	0.7463 (0.7246 - 0.7725)	0.7215 (0.6759 - 0.8249)	0.7712 (0.6873 - 0.8057)	0.7592 (0.7058 - 0.7915)	0.7347 (0.7038 - 0.8013)	0.7399 (0.7121 - 0.7787)	0.7683 (0.7377 - 0.7953)	0.7254 (0.7035 - 0.7557)	0.6376 (0.6014 - 0.7614)	0.8132 (0.7163 - 0.8186)	0.7734 (0.7071 - 0.7921)	0.6917 (0.6649 - 0.7608)	0.6989 (0.6691 - 0.7525)
Cardiomyopathy	0.7421 (0.7083 - 0.7751)	0.6950 (0.6694 - 0.7263)	0.6417 (0.5547 - 0.7879)	0.7483 (0.6175 - 0.8301)	0.7183 (0.6503 - 0.7699)	0.6762 (0.6351 - 0.7515)	0.6779 (0.6301 - 0.7297)	0.7648 (0.7366 - 0.7941)	0.7097 (0.6868 - 0.7426)	0.6929 (0.6008 - 0.7909)	0.7264 (0.6544 - 0.8046)	0.7170 (0.6696 - 0.7711)	0.7029 (0.6614 - 0.7716)	0.7047 (0.6661 - 0.7470)	0.7160 (0.6802 - 0.7497)	0.6759 (0.6497 - 0.7121)	0.5866 (0.4925 - 0.7519)	0.7651 (0.6164 - 0.8340)	0.7141 (0.6405 - 0.7739)	0.6492 (0.6060 - 0.7133)	0.6441 (0.5886 - 0.7083)
Sequelae of cerebrovascular disease	0.7448 (0.7132 - 0.7789)	0.6971 (0.6766 - 0.7300)	0.7216 (0.5247 - 0.7746)	0.6726 (0.6582 - 0.8632)	0.6879 (0.6624 - 0.8024)	0.7072 (0.6367 - 0.7516)	0.7043 (0.6278 - 0.7396)	0.7552 (0.7251 - 0.7831)	0.7037 (0.6827 - 0.7326)	0.7529 (0.6478 - 0.8192)	0.6545 (0.5905 - 0.7421)	0.6855 (0.6516 - 0.7329)	0.7260 (0.6701 - 0.7821)	0.7176 (0.6739 - 0.7505)	0.7266 (0.6957 - 0.7585)	0.6754 (0.6533 - 0.7084)	0.6706 (0.5603 - 0.8140)	0.6802 (0.5546 - 0.7908)	0.6771 (0.6266 - 0.7359)	0.6737 (0.6278 - 0.7586)	0.6738 (0.6258 - 0.7293)
Subarachnoid haemorrhage	0.6002 (0.5652 - 0.6379)	0.5905 (0.5684 - 0.6277)	0.4016 (0.3346 - 0.7188)	0.7794 (0.4831 - 0.8249)	0.6455 (0.5564 - 0.6914)	0.5657 (0.5383 - 0.6529)	0.4952 (0.4433 - 0.6523)	0.5625 (0.5271 - 0.5983)	0.5513 (0.5313 - 0.5893)	0.5574 (0.3158 - 0.9541)	0.5453 (0.1381 - 0.7797)	0.5507 (0.5067 - 0.6354)	0.5520 (0.5167 - 0.7552)	0.5540 (0.4107 - 0.6861)	0.5228 (0.4885 - 0.5584)	0.5348 (0.5121 - 0.5667)	0.8648 (0.1675 - 0.9498)	0.2049 (0.1202 - 0.8952)	0.5210 (0.4923 - 0.6276)	0.6024 (0.5076 - 0.7148)	0.6502 (0.2639 - 0.6809)
Atherosclerosis	0.8104 (0.7813 - 0.8392)	0.7628 (0.7379 - 0.7920)	0.7633 (0.6681 - 0.8139)	0.7624 (0.7454 - 0.8460)	0.7626 (0.7391 - 0.8198)	0.7631 (0.7077 - 0.8099)	0.7629 (0.7204 - 0.7950)	0.8244 (0.7950 - 0.8504)	0.7658 (0.7457 - 0.7922)	0.8408 (0.7039 - 0.8920)	0.6907 (0.6634 - 0.8405)	0.7311 (0.7065 - 0.8063)	0.8127 (0.7299 - 0.8660)	0.7821 (0.7427 - 0.8097)	0.7870 (0.7559 - 0.8162)	0.7401 (0.7159 - 0.7701)	0.7102 (0.6589 - 0.8500)	0.7700 (0.6225 - 0.8008)	0.7554 (0.6839 - 0.7837)	0.7266 (0.6898 - 0.8071)	0.7321 (0.7004 - 0.7757)
Subsequent myocardial infarction	0.8772 (0.8524 - 0.9002)	0.8191 (0.8015 - 0.8453)	0.8403 (0.7701 - 0.9099)	0.7979 (0.7625 - 0.8653)	0.8062 (0.7699 - 0.8519)	0.8333 (0.7831 - 0.8966)	0.8229 (0.7963 - 0.8537)	0.8886 (0.8678 - 0.9078)	0.8242 (0.8072 - 0.8521)	0.8319 (0.7872 - 0.9194)	0.8166 (0.7550 - 0.8539)	0.8193 (0.7691 - 0.8575)	0.8293 (0.7928 - 0.9089)	0.8256 (0.8069 - 0.8583)	0.8662 (0.8439 - 0.8871)	0.8096 (0.7867 - 0.8315)	0.8277 (0.7863 - 0.9159)	0.7915 (0.6951 - 0.8141)	0.7988 (0.7376 - 0.8240)	0.8213 (0.7871 - 0.8943)	0.8130 (0.7882 - 0.8435)
Occlusion and stenosis of precerebral arteries, not	0.7996 (0.7648 -	0.7423 (0.7163 -	0.7184 (0.6295 -	0.7661 (0.6894 -	0.7544 (0.6927 -	0.7313 (0.6826 -	0.7360 (0.6935 -	0.8321 (0.8059 -	0.7707 (0.7445 -	0.7621 (0.7074 -	0.7792 (0.6808 -	0.7754 (0.7100 -	0.7661 (0.7223 -	0.7687 (0.7372 -	0.8111 (0.7813 -	0.7626 (0.7374 -	0.7864 (0.7027 -	0.7389 (0.7041 -	0.7507 (0.7193 -	0.7757 (0.7185 -	0.7681 (0.7308 -

resulting in cerebral infarction	0.8329)	0.7761)	0.8358)	0.8538)	0.8146)	0.8073)	0.7843)	0.8606)	0.8018)	0.8713)	0.8122)	0.8090)	0.8455)	0.8097)	0.8409)	0.7946)	0.8495)	0.8081)	0.8019)	0.8307)	0.8028)
Arterial embolism and thrombosis	0.7633 (0.7280 - 0.7988)	0.6962 (0.6732 - 0.7345)	0.5862 (0.5117 - 0.8430)	0.8063 (0.5569 - 0.8586)	0.7516 (0.6352 - 0.8107)	0.6608 (0.6240 - 0.7901)	0.6587 (0.6134 - 0.7412)	0.7664 (0.7325 - 0.8000)	0.7102 (0.6778 - 0.7459)	0.5813 (0.5240 - 0.7643)	0.8391 (0.6276 - 0.8468)	0.7832 (0.6789 - 0.8139)	0.6671 (0.6265 - 0.7490)	0.6673 (0.6274 - 0.7321)	0.7253 (0.6886 - 0.7618)	0.6834 (0.6573 - 0.7196)	0.6552 (0.5306 - 0.8000)	0.7116 (0.5975 - 0.8230)	0.6944 (0.6312 - 0.7707)	0.6736 (0.6231 - 0.7599)	0.6742 (0.6134 - 0.7305)
Other acute ischaemic heart diseases	0.8413 (0.8060 - 0.8730)	0.7809 (0.7556 - 0.8138)	0.7895 (0.6831 - 0.8549)	0.7722 (0.7491 - 0.8740)	0.7761 (0.7449 - 0.8513)	0.7858 (0.7196 - 0.8457)	0.7827 (0.7398 - 0.8187)	0.8796 (0.8535 - 0.9022)	0.8239 (0.8028 - 0.8491)	0.8789 (0.8032 - 0.9290)	0.7689 (0.7231 - 0.8428)	0.7918 (0.7592 - 0.8405)	0.8640 (0.8096 - 0.9163)	0.8331 (0.8055 - 0.8620)	0.8591 (0.8321 - 0.8843)	0.8174 (0.7945 - 0.8452)	0.8263 (0.7749 - 0.9147)	0.8084 (0.7229 - 0.8424)	0.8118 (0.7518 - 0.8417)	0.8231 (0.7815 - 0.8960)	0.8190 (0.7903 - 0.8527)
Other diseases of pericardium	0.6230 (0.5799 - 0.6638)	0.5991 (0.5747 - 0.6371)	0.7263 (0.3442 - 0.8087)	0.4719 (0.4119 - 0.8400)	0.5790 (0.5463 - 0.7085)	0.6329 (0.5499 - 0.6967)	0.6443 (0.4528 - 0.6870)	0.6275 (0.5852 - 0.6711)	0.6072 (0.5831 - 0.6512)	0.4947 (0.3807 - 0.7290)	0.7198 (0.5122 - 0.8315)	0.6384 (0.5639 - 0.7037)	0.5875 (0.5517 - 0.6746)	0.5575 (0.4842 - 0.6662)	0.6070 (0.5666 - 0.6450)	0.5922 (0.5681 - 0.6318)	0.5316 (0.3891 - 0.7631)	0.6528 (0.4567 - 0.7641)	0.6049 (0.5431 - 0.6753)	0.5822 (0.5404 - 0.6610)	0.5659 (0.4777 - 0.6647)
Multiple valve diseases	0.7161 (0.6718 - 0.7546)	0.6793 (0.6502 - 0.7168)	0.5960 (0.4785 - 0.7088)	0.7627 (0.6912 - 0.8677)	0.7152 (0.6590 - 0.8004)	0.6537 (0.6078 - 0.7224)	0.6502 (0.5832 - 0.7090)	0.7595 (0.7247 - 0.7942)	0.7079 (0.6805 - 0.7429)	0.6919 (0.6010 - 0.7874)	0.7239 (0.6677 - 0.8221)	0.7148 (0.6660 - 0.7619)	0.7015 (0.6505 - 0.7657)	0.7032 (0.6594 - 0.7518)	0.7336 (0.6960 - 0.7664)	0.6835 (0.6516 - 0.7168)	0.6364 (0.5247 - 0.7658)	0.7307 (0.5881 - 0.8229)	0.7027 (0.6408 - 0.7627)	0.6677 (0.6157 - 0.7318)	0.6679 (0.6086 - 0.7192)
Aortic aneurysm and dissection	0.8026 (0.7717 - 0.8309)	0.7389 (0.7149 - 0.7701)	0.7838 (0.6783 - 0.8564)	0.6939 (0.6577 - 0.7868)	0.7192 (0.6751 - 0.7796)	0.7624 (0.6990 - 0.8283)	0.7501 (0.7062 - 0.7857)	0.8106 (0.7801 - 0.8387)	0.7616 (0.7335 - 0.7924)	0.7405 (0.6887 - 0.8400)	0.7827 (0.7029 - 0.7902)	0.7731 (0.7133 - 0.8004)	0.7510 (0.7073 - 0.8233)	0.7565 (0.7227 - 0.7976)	0.7813 (0.7479 - 0.8146)	0.7303 (0.7042 - 0.7690)	0.6595 (0.6094 - 0.8490)	0.8012 (0.6215 - 0.8311)	0.7683 (0.6727 - 0.8052)	0.7017 (0.6652 - 0.8137)	0.7097 (0.6792 - 0.7759)
Intracerebral haemorrhage	0.6541 (0.6027 - 0.6985)	0.6347 (0.5974 - 0.6762)	0.5682 (0.4586 - 0.8208)	0.7012 (0.4118 - 0.7917)	0.6553 (0.5712 - 0.7109)	0.6189 (0.5670 - 0.7258)	0.6087 (0.5378 - 0.6953)	0.6683 (0.6230 - 0.7129)	0.6283 (0.6014 - 0.6781)	0.4621 (0.4196 - 0.8724)	0.7944 (0.4087 - 0.8174)	0.6921 (0.5621 - 0.7460)	0.5963 (0.5636 - 0.7644)	0.5542 (0.5224 - 0.7191)	0.6392 (0.5923 - 0.6801)	0.6112 (0.5827 - 0.6565)	0.4773 (0.4471 - 0.9048)	0.7452 (0.3441 - 0.7491)	0.6519 (0.5414 - 0.6955)	0.5877 (0.5610 - 0.7908)	0.5511 (0.5270 - 0.7149)
Diseases of capillaries	0.5155 (0.4541 -	0.5317 (0.4987 -	0.5169 (0.0610 -	0.5465 (0.2243 -	0.5327 (0.4845 -	0.5308 (0.4859 -	0.5246 (0.1125 -	0.5746 (0.5175 -	0.5802 (0.5476 -	0.6966 (0.4238 -	0.4637 (0.3786 -	0.5650 (0.5144 -	0.6045 (0.5289 -	0.6240 (0.4900 -	0.5347 (0.4769 -	0.5543 (0.5141 -	0.2809 (0.1909 -	0.8276 (0.2362 -	0.6197 (0.4972 -	0.5351 (0.4905 -	0.3866 (0.2861 -

	0.5773)	0.5966)	0.8703)	0.9815)	0.7946)	0.6588)	0.6608)	0.6300)	0.6344)	0.8113)	0.6980)	0.6482)	0.7078)	0.6839)	0.5975)	0.6166)	0.8800)	0.8887)	0.7075)	0.6791)	0.6725)
Cardiac arrest	0.7643 (0.7149 - 0.8104)	0.7174 (0.6862 - 0.7684)	0.6327 (0.5688 - 0.8434)	0.8021 (0.6417 - 0.8250)	0.7617 (0.6608 - 0.8046)	0.6859 (0.6411 - 0.8152)	0.6912 (0.6442 - 0.7734)	0.8113 (0.7673 - 0.8485)	0.7765 (0.7469 - 0.8163)	0.8163 (0.7312 - 0.9231)	0.7366 (0.6717 - 0.8014)	0.7561 (0.7027 - 0.8061)	0.8004 (0.7448 - 0.9062)	0.7850 (0.7441 - 0.8308)	0.7621 (0.7141 - 0.8066)	0.7207 (0.6832 - 0.7676)	0.6633 (0.5900 - 0.8614)	0.7782 (0.5167 - 0.8249)	0.7494 (0.6527 - 0.7938)	0.6980 (0.6467 - 0.8168)	0.7037 (0.6519 - 0.7732)
Other pulmonary heart diseases	0.6169 (0.5535 - 0.6858)	0.6164 (0.5795 - 0.6763)	0.4211 (0.3401 - 0.7283)	0.8117 (0.4716 - 0.8541)	0.6910 (0.5643 - 0.7596)	0.5837 (0.5392 - 0.6883)	0.5233 (0.4482 - 0.6663)	0.6875 (0.6280 - 0.7435)	0.6786 (0.6353 - 0.7246)	0.6842 (0.5862 - 0.7882)	0.6731 (0.6437 - 0.7428)	0.6767 (0.6180 - 0.7275)	0.6806 (0.6074 - 0.7723)	0.6804 (0.6119 - 0.7414)	0.6711 (0.6159 - 0.7231)	0.6417 (0.6046 - 0.6964)	0.6000 (0.4666 - 0.8236)	0.6834 (0.4454 - 0.7994)	0.6546 (0.5722 - 0.7276)	0.6308 (0.5777 - 0.7443)	0.6261 (0.5477 - 0.7138)
Rheumatic mitral valve diseases	0.7052 (0.6404 - 0.7691)	0.6871 (0.6439 - 0.7401)	0.6333 (0.5408 - 0.8462)	0.7409 (0.5522 - 0.7913)	0.7097 (0.6136 - 0.7637)	0.6689 (0.6138 - 0.8059)	0.6693 (0.6068 - 0.7546)	0.6890 (0.6312 - 0.7420)	0.6559 (0.6165 - 0.7141)	0.5444 (0.4704 - 0.8294)	0.7674 (0.4758 - 0.7917)	0.7007 (0.5903 - 0.7539)	0.6275 (0.5757 - 0.7666)	0.6128 (0.5577 - 0.7284)	0.6254 (0.5685 - 0.6809)	0.6081 (0.5708 - 0.6620)	0.6778 (0.3253 - 0.8125)	0.5383 (0.4488 - 0.8646)	0.5948 (0.5386 - 0.7287)	0.6256 (0.5464 - 0.7469)	0.6336 (0.4408 - 0.7041)
Atherosclerotic heart disease	0.8947 (0.8903 - 0.8986)	0.8315 (0.8269 - 0.8366)	0.8444 (0.8223 - 0.8671)	0.8185 (0.7977 - 0.8379)	0.8231 (0.8089 - 0.8374)	0.8402 (0.8242 - 0.8578)	0.8336 (0.8271 - 0.8403)	0.8819 (0.8771 - 0.8865)	0.8127 (0.8075 - 0.8181)	0.8226 (0.8113 - 0.8606)	0.8028 (0.7630 - 0.8121)	0.8066 (0.7835 - 0.8132)	0.8190 (0.8098 - 0.8461)	0.8145 (0.8088 - 0.8231)	0.8779 (0.8734 - 0.8825)	0.8140 (0.8094 - 0.8196)	0.8340 (0.8052 - 0.8505)	0.7940 (0.7747 - 0.8223)	0.8020 (0.7918 - 0.8198)	0.8271 (0.8070 - 0.8390)	0.8177 (0.8091 - 0.8236)
Angina pectoris, unspecified	0.8460 (0.8401 - 0.8522)	0.7853 (0.7803 - 0.7922)	0.7764 (0.7527 - 0.8000)	0.7942 (0.7774 - 0.8136)	0.7904 (0.7779 - 0.8053)	0.7803 (0.7660 - 0.7976)	0.7834 (0.7750 - 0.7931)	0.8320 (0.8260 - 0.8381)	0.7647 (0.7592 - 0.7717)	0.7782 (0.7344 - 0.7944)	0.7512 (0.7383 - 0.7914)	0.7577 (0.7499 - 0.7826)	0.7720 (0.7478 - 0.7836)	0.7678 (0.7552 - 0.7760)	0.8266 (0.8203 - 0.8328)	0.7639 (0.7583 - 0.7706)	0.7562 (0.7438 - 0.7933)	0.7716 (0.7367 - 0.7810)	0.7680 (0.7477 - 0.7747)	0.7599 (0.7507 - 0.7829)	0.7621 (0.7552 - 0.7748)
Chronic ischaemic heart disease, unspecified	0.8456 (0.8370 - 0.8539)	0.7886 (0.7805 - 0.7976)	0.7909 (0.7734 - 0.8164)	0.7863 (0.7691 - 0.7954)	0.7873 (0.7740 - 0.7972)	0.7899 (0.7757 - 0.8100)	0.7891 (0.7790 - 0.8012)	0.8313 (0.8223 - 0.8396)	0.7639 (0.7563 - 0.7735)	0.7737 (0.7215 - 0.8228)	0.7541 (0.7056 - 0.8097)	0.7588 (0.7350 - 0.7900)	0.7692 (0.7429 - 0.8018)	0.7662 (0.7521 - 0.7819)	0.8197 (0.8111 - 0.8287)	0.7584 (0.7516 - 0.7682)	0.7651 (0.7120 - 0.7934)	0.7516 (0.7253 - 0.8043)	0.7549 (0.7397 - 0.7869)	0.7619 (0.7338 - 0.7826)	0.7600 (0.7444 - 0.7718)
Other forms of chronic ischaemic	0.8895 (0.8816 -	0.8279 (0.8209 -	0.8292 (0.8128 -	0.8266 (0.7858 -	0.8270 (0.7989 -	0.8288 (0.8155 -	0.8281 (0.8206 -	0.8820 (0.8753 -	0.8131 (0.8046 -	0.8312 (0.8014 -	0.7950 (0.7785 -	0.8022 (0.7895 -	0.8249 (0.8048 -	0.8164 (0.8054 -	0.8726 (0.8649 -	0.8108 (0.8033 -	0.8332 (0.8091 -	0.7884 (0.7547 -	0.7975 (0.7789 -	0.8254 (0.8076 -	0.8150 (0.8056 -

heart	0.896	0.837	0.874	0.842	0.838	0.864	0.841	0.888	0.821	0.852	0.830	0.822	0.843	0.827	0.879	0.819	0.865	0.806	0.812	0.851	0.825
disease	5)	2)	6)	0)	8)	3)	7)	6)	9)	0)	4)	0)	7)	1)	8)	2)	3)	3)	3)	2)	6)
	0.848	0.788	0.811	0.765	0.775	0.802	0.793	0.840	0.777	0.779	0.774	0.775	0.778	0.777	0.831	0.773	0.787	0.758	0.765	0.781	0.776
	7	5	6	4	7	5	3	3	1	8	4	6	6	7	3	2	6	8	5	3	4
	(0.83	(0.78	(0.75	(0.74	(0.76	(0.76	(0.77	(0.83	(0.76	(0.73	(0.74	(0.75	(0.75	(0.76	(0.82	(0.76	(0.72	(0.72	(0.75	(0.74	(0.75
	92 -	07 -	65 -	47 -	13 -	87 -	84 -	14 -	87 -	09 -	33 -	68 -	18 -	28 -	17 -	45 -	87 -	75 -	06 -	80 -	87 -
Unstable	0.857	0.798	0.838	0.816	0.808	0.824	0.805	0.848	0.787	0.815	0.820	0.804	0.802	0.791	0.840	0.783	0.816	0.815	0.800	0.802	0.789
angina	3)	3)	8)	2)	1)	1)	6)	9)	4)	0)	5)	5)	7)	4)	8)	5)	5)	4)	5)	9)	5)
	0.861	0.794	0.808	0.781	0.786	0.803	0.797	0.861	0.787	0.828	0.745	0.765	0.812	0.795	0.847	0.780	0.824	0.736	0.757	0.807	0.789
	7	9	8	0	9	3	7	6	1	3	9	3	9	5	2	3	4	2	6	4	6
	(0.85	(0.78	(0.75	(0.72	(0.75	(0.77	(0.78	(0.85	(0.77	(0.77	(0.70	(0.74	(0.77	(0.77	(0.83	(0.77	(0.74	(0.72	(0.74	(0.75	(0.76
Old	07 -	57 -	97 -	32 -	18 -	23 -	35 -	17 -	78 -	52 -	99 -	00 -	76 -	98 -	62 -	02 -	49 -	00 -	28 -	87 -	82 -
myocardial	0.872	0.808	0.873	0.824	0.817	0.854	0.818	0.871	0.799	0.877	0.793	0.797	0.854	0.811	0.858	0.793	0.853	0.819	0.805	0.834	0.803
infarction	4)	2)	9)	0)	1)	9)	2)	6)	7)	5)	7)	0)	6)	6)	3)	7)	9)	9)	7)	7)	2)
	0.877	0.822	0.847	0.797	0.806	0.839	0.826	0.879	0.815	0.844	0.787	0.798	0.835	0.821	0.870	0.808	0.836	0.780	0.792	0.826	0.813
	7	0	0	1	7	0	4	4	9	6	2	8	1	0	4	4	1	6	2	5	6
Acute	(0.86	(0.81	(0.82	(0.77	(0.78	(0.81	(0.81	(0.86	(0.80	(0.78	(0.75	(0.77	(0.79	(0.80	(0.85	(0.79	(0.79	(0.76	(0.77	(0.79	(0.79
myocardial	73 -	13 -	24 -	00 -	73 -	73 -	22 -	78 -	45 -	86 -	76 -	99 -	64 -	34 -	89 -	75 -	51 -	18 -	35 -	73 -	78 -
infarction,	0.889	0.834	0.879	0.812	0.820	0.867	0.841	0.889	0.829	0.877	0.837	0.836	0.863	0.834	0.880	0.821	0.867	0.813	0.818	0.853	0.829
unspecified	0)	5)	0)	9)	4)	4)	6)	0)	6)	4)	1)	1)	1)	9)	2)	6)	9)	6)	5)	8)	1)
Acute	0.886	0.828	0.840	0.815	0.820	0.836	0.830	0.883	0.816	0.873	0.760	0.784	0.856	0.826	0.873	0.812	0.856	0.768	0.787	0.842	0.820
transmural	7	0	5	5	0	4	1	3	9	0	7	9	9	6	0	8	8	8	5	9	7
myocardial	(0.87	(0.81	(0.80	(0.79	(0.80	(0.80	(0.81	(0.87	(0.80	(0.80	(0.73	(0.76	(0.80	(0.80	(0.86	(0.80	(0.82	(0.74	(0.76	(0.81	(0.80
infarction	48 -	39 -	50 -	50 -	33 -	78 -	32 -	35 -	56 -	66 -	67 -	80 -	92 -	88 -	16 -	04 -	45 -	11 -	87 -	42 -	61 -
of inferior	0.897	0.841	0.867	0.831	0.836	0.862	0.845	0.892	0.829	0.897	0.823	0.823	0.881	0.840	0.883	0.825	0.885	0.792	0.805	0.870	0.836
wall	4)	7)	0)	4)	5)	6)	9)	9)	5)	3)	6)	6)	0)	0)	6)	8)	9)	7)	2)	6)	7)
	0.863	0.802	0.827	0.777	0.787	0.818	0.806	0.858	0.787	0.758	0.816	0.805	0.771	0.781	0.833	0.776	0.764	0.788	0.782	0.769	0.773
	5	1	1	0	7	0	9	2	5	6	5	2	8	2	0	3	5	0	9	9	6
	(0.84	(0.79	(0.76	(0.75	(0.76	(0.77	(0.78	(0.84	(0.77	(0.73	(0.71	(0.74	(0.75	(0.76	(0.81	(0.76	(0.72	(0.74	(0.75	(0.74	(0.75
Left	75 -	01 -	67 -	01 -	84 -	76 -	86 -	33 -	47 -	52 -	87 -	59 -	29 -	74 -	66 -	35 -	62 -	88 -	32 -	35 -	61 -
ventricular	0.877	0.818	0.862	0.829	0.824	0.849	0.824	0.871	0.803	0.863	0.825	0.820	0.841	0.808	0.849	0.794	0.822	0.815	0.809	0.812	0.797
failure	1)	0)	8)	4)	5)	5)	8)	3)	7)	1)	0)	9)	0)	8)	4)	1)	2)	0)	2)	7)	2)
Pulmonary	0.662	0.626	0.629	0.623	0.626	0.627	0.627	0.668	0.629	0.688	0.571	0.616	0.646	0.650	0.624	0.599	0.688	0.510	0.584	0.620	0.632
embolism	4	8	7	9	1	5	9	5	7	2	3	2	9	2	4	3	2	3	3	7	0
without	(0.64	(0.61	(0.50	(0.56	(0.59	(0.59	(0.57	(0.64	(0.61	(0.60	(0.49	(0.59	(0.61	(0.61	(0.60	(0.58	(0.58	(0.43	(0.56	(0.58	(0.58
mention of	12 -	16 -	15 -	77 -	92 -	59 -	15 -	81 -	40 -	53 -	41 -	16 -	25 -	35 -	39 -	63 -	57 -	15 -	34 -	78 -	69 -
acute cor	0.683	0.648	0.699	0.750	0.668	0.664	0.660	0.687	0.649	0.764	0.645	0.646	0.686	0.681	0.642	0.618	0.763	0.605	0.616	0.661	0.663
pulmonale	0)	2)	6)	6)	7)	5)	9)	9)	5)	1)	7)	9)	3)	4)	8)	8)	9)	4)	2)	9)	9)
	0.605	0.586	0.635	0.538	0.579	0.596	0.605	0.610	0.583	0.567	0.600	0.586	0.581	0.577	0.580	0.562	0.580	0.544	0.560	0.564	0.570
Supraventri	5	7	3	2	1	1	9	6	9	7	2	8	3	1	9	5	9	1	3	9	4
cular	(0.58	(0.57	(0.35	(0.49	(0.55	(0.55	(0.45	(0.58	(0.56	(0.43	(0.41	(0.55	(0.55	(0.50	(0.55	(0.54	(0.33	(0.34	(0.53	(0.53	(0.42
tachycardia	21 -	19 -	44 -	34 -	46 -	65 -	76 -	85 -	97 -	72 -	76 -	13 -	60 -	80 -	95 -	90 -	59 -	47 -	48 -	44 -	99 -

	0.6282)	0.6084)	0.6995)	0.8289)	0.6566)	0.6339)	0.6401)	0.6321)	0.6055)	0.7603)	0.7232)	0.6278)	0.6408)	0.6505)	0.6025)	0.5844)	0.7665)	0.7956)	0.6177)	0.6084)	0.6389)
Peripheral vascular disease, unspecified	0.8604 (0.8447 - 0.8747)	0.8002 (0.7866 - 0.8173)	0.7706 (0.7466 - 0.8420)	0.8298 (0.7631 - 0.8381)	0.8191 (0.7761 - 0.8332)	0.7835 (0.7647 - 0.8335)	0.7941 (0.7808 - 0.8181)	0.8539 (0.8386 - 0.8688)	0.7900 (0.7738 - 0.8069)	0.7607 (0.7301 - 0.8174)	0.8192 (0.7603 - 0.8312)	0.8080 (0.7669 - 0.8218)	0.7739 (0.7487 - 0.8133)	0.7836 (0.7641 - 0.8060)	0.8333 (0.8181 - 0.8486)	0.7657 (0.7508 - 0.7831)	0.7970 (0.7328 - 0.8574)	0.7344 (0.6725 - 0.8106)	0.7500 (0.7175 - 0.7855)	0.7835 (0.7465 - 0.8284)	0.7728 (0.7505 - 0.7946)
Acute transmural myocardial infarction of anterior wall	0.8849 (0.8709 - 0.8984)	0.8290 (0.8146 - 0.8448)	0.8472 (0.8134 - 0.8839)	0.8107 (0.7767 - 0.8398)	0.8174 (0.7969 - 0.8351)	0.8414 (0.8137 - 0.8750)	0.8320 (0.8153 - 0.8502)	0.8870 (0.8735 - 0.8985)	0.8216 (0.8076 - 0.8371)	0.8090 (0.7848 - 0.8950)	0.8341 (0.7554 - 0.8377)	0.8299 (0.7699 - 0.8426)	0.8137 (0.7943 - 0.8793)	0.8193 (0.8045 - 0.8423)	0.8741 (0.8597 - 0.8874)	0.8137 (0.8022 - 0.8309)	0.8576 (0.8063 - 0.9011)	0.7698 (0.7335 - 0.8203)	0.7884 (0.7637 - 0.8229)	0.8439 (0.8070 - 0.8849)	0.8216 (0.8046 - 0.8403)
Transient cerebral ischaemic attack, unspecified	0.7470 (0.7235 - 0.7719)	0.7042 (0.6853 - 0.7270)	0.6354 (0.5917 - 0.7654)	0.7729 (0.6290 - 0.8024)	0.7367 (0.6751 - 0.7642)	0.6795 (0.6541 - 0.7335)	0.6823 (0.6545 - 0.7236)	0.7561 (0.7333 - 0.7779)	0.7172 (0.6975 - 0.7385)	0.6687 (0.6197 - 0.7243)	0.7656 (0.7189 - 0.7974)	0.7405 (0.7098 - 0.7647)	0.6980 (0.6671 - 0.7360)	0.7028 (0.6736 - 0.7335)	0.7338 (0.7099 - 0.7586)	0.6931 (0.6765 - 0.7185)	0.6375 (0.5732 - 0.7573)	0.7486 (0.6393 - 0.8030)	0.7172 (0.6638 - 0.7539)	0.6738 (0.6443 - 0.7311)	0.6750 (0.6432 - 0.7225)
Cerebral infarction, unspecified	0.7908 (0.7669 - 0.8141)	0.7455 (0.7253 - 0.7689)	0.6959 (0.6493 - 0.7852)	0.7950 (0.7083 - 0.8230)	0.7725 (0.7185 - 0.7960)	0.7234 (0.6920 - 0.7722)	0.7322 (0.7038 - 0.7643)	0.8106 (0.7915 - 0.8292)	0.7430 (0.7264 - 0.7647)	0.7207 (0.6643 - 0.8485)	0.7652 (0.6392 - 0.8105)	0.7543 (0.6931 - 0.7879)	0.7326 (0.6999 - 0.8121)	0.7371 (0.7143 - 0.7744)	0.7828 (0.7597 - 0.8035)	0.7185 (0.7019 - 0.7424)	0.6644 (0.6146 - 0.8318)	0.7725 (0.5886 - 0.8162)	0.7449 (0.6651 - 0.7814)	0.6971 (0.6699 - 0.7912)	0.7024 (0.6789 - 0.7553)
Mitral (valve) insufficiency	0.7177 (0.6919 - 0.7427)	0.6677 (0.6463 - 0.6958)	0.5670 (0.4974 - 0.6835)	0.7683 (0.6396 - 0.8321)	0.7099 (0.6544 - 0.7521)	0.6396 (0.6092 - 0.6861)	0.6305 (0.5893 - 0.6775)	0.7102 (0.6824 - 0.7373)	0.6612 (0.6399 - 0.6886)	0.6289 (0.5228 - 0.7003)	0.6936 (0.6726 - 0.8068)	0.6724 (0.6394 - 0.7251)	0.6514 (0.6124 - 0.7022)	0.6499 (0.6025 - 0.6926)	0.6744 (0.6456 - 0.7025)	0.6359 (0.6167 - 0.6629)	0.5851 (0.5301 - 0.7592)	0.6868 (0.5068 - 0.7262)	0.6513 (0.5996 - 0.6868)	0.6234 (0.5966 - 0.6908)	0.6164 (0.5827 - 0.6807)
Cardiomegaly	0.7153 (0.6833 - 0.7448)	0.6760 (0.6528 - 0.7045)	0.5620 (0.4862 - 0.7016)	0.7901 (0.6579 - 0.8361)	0.7280 (0.6625 - 0.7682)	0.6433 (0.6082 - 0.6918)	0.6343 (0.5868 - 0.6903)	0.7603 (0.7337 - 0.7839)	0.7062 (0.6860 - 0.7335)	0.7107 (0.5910 - 0.7979)	0.7017 (0.6293 - 0.8112)	0.7043 (0.6638 - 0.7691)	0.7081 (0.6552 - 0.7658)	0.7075 (0.6597 - 0.7431)	0.7260 (0.6977 - 0.7506)	0.6880 (0.6670 - 0.7114)	0.6997 (0.6108 - 0.7427)	0.6762 (0.6652 - 0.7567)	0.6837 (0.6593 - 0.7289)	0.6925 (0.6455 - 0.7303)	0.6916 (0.6485 - 0.7196)
Other forms of angina pectoris	0.8229 (0.7997 -	0.7598 (0.7436 -	0.7907 (0.7135 -	0.7289 (0.6596 -	0.7447 (0.6995 -	0.7769 (0.7330 -	0.7670 (0.7413 -	0.8419 (0.8227 -	0.7783 (0.7621 -	0.7994 (0.7273 -	0.7572 (0.6750 -	0.7670 (0.7227 -	0.7906 (0.7492 -	0.7829 (0.7591 -	0.8226 (0.8002 -	0.7637 (0.7481 -	0.8343 (0.7198 -	0.6932 (0.6741 -	0.7311 (0.7105 -	0.8071 (0.7318 -	0.7793 (0.7446 -

	0.8452)	0.7855)	0.8788)	0.7979)	0.7920)	0.8478)	0.7998)	0.8603)	0.8017)	0.8832)	0.8266)	0.8161)	0.8592)	0.8126)	0.8434)	0.7877)	0.8704)	0.8019)	0.7974)	0.8442)	0.8020)
Acute subendocardial myocardial infarction	0.8598 (0.8391 - 0.8789)	0.8028 (0.7840 - 0.8259)	0.8158 (0.7801 - 0.8834)	0.7898 (0.7482 - 0.8246)	0.7951 (0.7581 - 0.8200)	0.8109 (0.7793 - 0.8710)	0.8053 (0.7833 - 0.8341)	0.8635 (0.8450 - 0.8797)	0.7945 (0.7750 - 0.8167)	0.8257 (0.7771 - 0.8984)	0.7634 (0.6737 - 0.8070)	0.7773 (0.7294 - 0.8116)	0.8141 (0.7771 - 0.8757)	0.8007 (0.7753 - 0.8262)	0.8458 (0.8251 - 0.8645)	0.7893 (0.7714 - 0.8127)	0.8388 (0.7766 - 0.8957)	0.7398 (0.7013 - 0.7849)	0.7632 (0.7304 - 0.7993)	0.8211 (0.7759 - 0.8739)	0.7992 (0.7747 - 0.8250)
Hypertensive renal disease with renal failure	0.9030 (0.8763 - 0.9267)	0.8501 (0.8307 - 0.8745)	0.8049 (0.7410 - 0.8616)	0.8953 (0.8602 - 0.9391)	0.8849 (0.8500 - 0.9312)	0.8211 (0.7790 - 0.8660)	0.8430 (0.8171 - 0.8706)	0.9217 (0.9021 - 0.9402)	0.8528 (0.8342 - 0.8790)	0.7666 (0.7361 - 0.8870)	0.9390 (0.8280 - 0.9520)	0.9263 (0.8299 - 0.9435)	0.8009 (0.7789 - 0.8847)	0.8389 (0.8200 - 0.8759)	0.8934 (0.8706 - 0.9137)	0.8301 (0.8154 - 0.8576)	0.8571 (0.7534 - 0.9058)	0.8031 (0.7685 - 0.9060)	0.8132 (0.7840 - 0.8966)	0.8490 (0.7826 - 0.8924)	0.8346 (0.8089 - 0.8599)
Congestive heart failure	0.8601 (0.8359 - 0.8842)	0.7968 (0.7745 - 0.8210)	0.7821 (0.7222 - 0.8517)	0.8115 (0.7351 - 0.8509)	0.8058 (0.7514 - 0.8403)	0.7884 (0.7454 - 0.8409)	0.7938 (0.7627 - 0.8241)	0.8478 (0.8240 - 0.8698)	0.7758 (0.7560 - 0.8019)	0.7607 (0.6784 - 0.8500)	0.7909 (0.7175 - 0.8613)	0.7844 (0.7348 - 0.8390)	0.7677 (0.7194 - 0.8327)	0.7724 (0.7387 - 0.8054)	0.8199 (0.7962 - 0.8423)	0.7603 (0.7398 - 0.7865)	0.7607 (0.7075 - 0.8369)	0.7599 (0.7093 - 0.7916)	0.7601 (0.7192 - 0.7923)	0.7605 (0.7224 - 0.8194)	0.7604 (0.7300 - 0.7947)
Aortic (valve) stenosis	0.7730 (0.7424 - 0.8032)	0.7147 (0.6944 - 0.7492)	0.6197 (0.5504 - 0.8361)	0.8097 (0.6090 - 0.8742)	0.7650 (0.6627 - 0.8277)	0.6804 (0.6472 - 0.7950)	0.6847 (0.6514 - 0.7593)	0.7787 (0.7477 - 0.8073)	0.7094 (0.6887 - 0.7437)	0.6838 (0.5845 - 0.8583)	0.7350 (0.5900 - 0.8505)	0.7207 (0.6507 - 0.7914)	0.6992 (0.6614 - 0.8101)	0.7018 (0.6628 - 0.7627)	0.7450 (0.7119 - 0.7800)	0.6995 (0.6764 - 0.7336)	0.6966 (0.6034 - 0.8233)	0.7025 (0.5838 - 0.7788)	0.7007 (0.6457 - 0.7534)	0.6984 (0.6501 - 0.7781)	0.6986 (0.6551 - 0.7486)
Left bundle-branch block, unspecified	0.6833 (0.6479 - 0.7210)	0.6644 (0.6348 - 0.6985)	0.5511 (0.4867 - 0.6696)	0.7777 (0.6550 - 0.8100)	0.7126 (0.6521 - 0.7496)	0.6340 (0.5959 - 0.6833)	0.6215 (0.5704 - 0.6795)	0.7186 (0.6805 - 0.7512)	0.6842 (0.6529 - 0.7147)	0.6222 (0.5518 - 0.7401)	0.7462 (0.6333 - 0.7934)	0.7103 (0.6531 - 0.7461)	0.6639 (0.6202 - 0.7254)	0.6634 (0.6150 - 0.7132)	0.7103 (0.6724 - 0.7458)	0.6887 (0.6620 - 0.7224)	0.6667 (0.5897 - 0.7295)	0.7108 (0.6892 - 0.7599)	0.6974 (0.6628 - 0.7411)	0.6807 (0.6309 - 0.7323)	0.6817 (0.6345 - 0.7245)
Other and unspecified right bundle-branch block	0.6332 (0.5921 - 0.6772)	0.6074 (0.5850 - 0.6504)	0.6667 (0.4076 - 0.7799)	0.5482 (0.4604 - 0.7829)	0.5960 (0.5649 - 0.7019)	0.6219 (0.5574 - 0.6819)	0.6294 (0.5018 - 0.6772)	0.6715 (0.6335 - 0.7083)	0.6302 (0.6041 - 0.6699)	0.5681 (0.4093 - 0.7651)	0.6922 (0.5023 - 0.8255)	0.6486 (0.5874 - 0.7385)	0.6158 (0.5666 - 0.6924)	0.6057 (0.5137 - 0.6722)	0.6394 (0.6012 - 0.6706)	0.6070 (0.5810 - 0.6408)	0.5775 (0.3645 - 0.8298)	0.6366 (0.3705 - 0.8401)	0.6137 (0.5531 - 0.7052)	0.6010 (0.5556 - 0.7049)	0.5950 (0.4794 - 0.6840)
Ventricular tachycardia	0.7161 (0.6733 -	0.6798 (0.6504 -	0.5344 (0.4532 -	0.8253 (0.6480 -	0.7536 (0.6490 -	0.6393 (0.5996 -	0.6253 (0.5700 -	0.7479 (0.7054 -	0.6940 (0.6668 -	0.5873 (0.5000 -	0.8008 (0.6539 -	0.7467 (0.6589 -	0.6599 (0.6193 -	0.6575 (0.6076 -	0.7152 (0.6735 -	0.6909 (0.6597 -	0.5979 (0.5165 -	0.7839 (0.6814 -	0.7345 (0.6654 -	0.6610 (0.6185 -	0.6592 (0.6066 -

	0.7577)	0.7197)	0.7182)	0.8705)	0.8109)	0.7152)	0.7026)	0.7843)	0.7346)	0.7600)	0.8828)	0.8151)	0.7452)	0.7288)	0.7544)	0.7266)	0.7174)	0.8526)	0.7870)	0.7211)	0.7147)
Cardiac arrhythmia, unspecified	0.5868 (0.5408 - 0.6355)	0.5871 (0.5617 - 0.6334)	0.3909 (0.3284 - 0.6405)	0.7833 (0.5844 - 0.8233)	0.6433 (0.5580 - 0.7003)	0.5625 (0.5297 - 0.6396)	0.4863 (0.4339 - 0.6293)	0.6011 (0.5607 - 0.6398)	0.5849 (0.5585 - 0.6213)	0.5939 (0.3220 - 0.8029)	0.5758 (0.3600 - 0.8261)	0.5834 (0.5406 - 0.6682)	0.5864 (0.5326 - 0.6622)	0.5886 (0.4289 - 0.6602)	0.5514 (0.5122 - 0.5912)	0.5596 (0.5304 - 0.5984)	0.5025 (0.3065 - 0.8116)	0.6168 (0.3038 - 0.7951)	0.5673 (0.5168 - 0.6351)	0.5535 (0.5111 - 0.6262)	0.5330 (0.4021 - 0.6465)
Occlusion and stenosis of carotid artery	0.8201 (0.7885 - 0.8502)	0.7689 (0.7433 - 0.7995)	0.7282 (0.6845 - 0.8615)	0.8096 (0.6772 - 0.8234)	0.7927 (0.7196 - 0.8192)	0.7487 (0.7090 - 0.8321)	0.7591 (0.7291 - 0.8047)	0.8413 (0.8153 - 0.8659)	0.7816 (0.7567 - 0.8110)	0.8051 (0.7072 - 0.8652)	0.7580 (0.7008 - 0.8537)	0.7689 (0.7288 - 0.8273)	0.7955 (0.7397 - 0.8529)	0.7866 (0.7483 - 0.8198)	0.8253 (0.7984 - 0.8526)	0.7685 (0.7446 - 0.8010)	0.7692 (0.6961 - 0.8945)	0.7677 (0.6167 - 0.8271)	0.7680 (0.7024 - 0.8184)	0.7689 (0.7193 - 0.8567)	0.7686 (0.7362 - 0.8101)
Raynaud's syndrome	0.6286 (0.5862 - 0.6725)	0.6043 (0.5810 - 0.6455)	0.4541 (0.4081 - 0.7744)	0.7546 (0.4526 - 0.7886)	0.6492 (0.5604 - 0.6937)	0.5802 (0.5553 - 0.6835)	0.5344 (0.4923 - 0.6729)	0.6438 (0.6034 - 0.6835)	0.6159 (0.5889 - 0.6521)	0.6378 (0.3635 - 0.7748)	0.5939 (0.4703 - 0.8554)	0.6110 (0.5670 - 0.7235)	0.6212 (0.5574 - 0.6933)	0.6241 (0.4800 - 0.6829)	0.5880 (0.5452 - 0.6271)	0.5757 (0.5464 - 0.6129)	0.6020 (0.3037 - 0.8307)	0.5494 (0.3123 - 0.8388)	0.5720 (0.5220 - 0.6503)	0.5799 (0.5300 - 0.6709)	0.5866 (0.4085 - 0.6679)
Stricture of artery	0.8220 (0.7889 - 0.8504)	0.7569 (0.7325 - 0.7902)	0.7514 (0.6243 - 0.8649)	0.7624 (0.6452 - 0.8895)	0.7597 (0.6959 - 0.8499)	0.7541 (0.6954 - 0.8371)	0.7555 (0.7113 - 0.7982)	0.8454 (0.8218 - 0.8680)	0.7726 (0.7480 - 0.8018)	0.8122 (0.7277 - 0.9064)	0.7331 (0.6329 - 0.8304)	0.7526 (0.6959 - 0.8050)	0.7960 (0.7382 - 0.8736)	0.7813 (0.7490 - 0.8178)	0.8073 (0.7780 - 0.8337)	0.7435 (0.7197 - 0.7748)	0.8453 (0.6818 - 0.9041)	0.6417 (0.6149 - 0.8107)	0.7023 (0.6643 - 0.7844)	0.8057 (0.7092 - 0.8707)	0.7672 (0.7171 - 0.7988)
Sequelae of stroke, not specified as haemorrhage or infarction	0.7379 (0.6936 - 0.7756)	0.6918 (0.6640 - 0.7316)	0.7843 (0.5768 - 0.8503)	0.5992 (0.5710 - 0.8026)	0.6618 (0.6260 - 0.7536)	0.7353 (0.6370 - 0.8010)	0.7179 (0.6413 - 0.7551)	0.7844 (0.7519 - 0.8156)	0.7379 (0.7152 - 0.7637)	0.9150 (0.7651 - 0.9545)	0.5608 (0.5592 - 0.7262)	0.6757 (0.6421 - 0.7387)	0.8684 (0.7463 - 0.9250)	0.7773 (0.7367 - 0.8042)	0.7476 (0.7138 - 0.7813)	0.7044 (0.6788 - 0.7386)	0.8497 (0.7151 - 0.9098)	0.5592 (0.5161 - 0.6773)	0.6584 (0.6214 - 0.7159)	0.7881 (0.6977 - 0.8608)	0.7419 (0.6889 - 0.7779)
Subarachnoid haemorrhage, unspecified	0.5803 (0.5302 - 0.6343)	0.5896 (0.5554 - 0.6357)	0.5168 (0.3253 - 0.6312)	0.6624 (0.5793 - 0.8427)	0.6048 (0.5553 - 0.6730)	0.5782 (0.5300 - 0.6437)	0.5574 (0.4354 - 0.6281)	0.5072 (0.4631 - 0.5494)	0.5260 (0.4995 - 0.5716)	0.8389 (0.0736 - 0.9578)	0.2132 (0.1094 - 0.9634)	0.5160 (0.4833 - 0.7595)	0.5696 (0.4898 - 0.7309)	0.6390 (0.1325 - 0.6838)	0.5019 (0.4573 - 0.5506)	0.5271 (0.4987 - 0.5733)	0.1745 (0.0580 - 0.6789)	0.8797 (0.3687 - 0.9569)	0.5918 (0.5066 - 0.7136)	0.5159 (0.4826 - 0.5775)	0.2695 (0.1063 - 0.5850)
Atherosclerosis of arteries of the extremities	0.7682 (0.7286 -	0.7111 (0.6825 -	0.7733 (0.5252 -	0.6489 (0.6068 -	0.6878 (0.6490 -	0.7411 (0.6404 -	0.7281 (0.6365 -	0.8233 (0.7885 -	0.7708 (0.7452 -	0.7400 (0.6918 -	0.8015 (0.6778 -	0.7885 (0.7156 -	0.7551 (0.7189 -	0.7635 (0.7332 -	0.7727 (0.7319 -	0.7337 (0.7033 -	0.7267 (0.6478 -	0.7408 (0.6733 -	0.7371 (0.6874 -	0.7305 (0.6812 -	0.7318 (0.6850 -

	0.8103)	0.7525)	0.8379)	0.8792)	0.8319)	0.8085)	0.7674)	0.8590)	0.8082)	0.8803)	0.8594)	0.8282)	0.8595)	0.8168)	0.8137)	0.7726)	0.8189)	0.8158)	0.7852)	0.8050)	0.7829)
Dilated cardiomyopathy	0.7493 (0.7045 - 0.7913)	0.7010 (0.6666 - 0.7432)	0.6828 (0.5637 - 0.7986)	0.7191 (0.6319 - 0.8282)	0.7085 (0.6473 - 0.7776)	0.6939 (0.6348 - 0.7707)	0.6954 (0.6356 - 0.7528)	0.7777 (0.7389 - 0.8157)	0.7169 (0.6924 - 0.7567)	0.8276 (0.5986 - 0.8828)	0.6062 (0.5831 - 0.8446)	0.6776 (0.6466 - 0.7956)	0.7786 (0.6636 - 0.8434)	0.7451 (0.6714 - 0.7811)	0.7270 (0.6850 - 0.7653)	0.6731 (0.6448 - 0.7172)	0.5724 (0.5032 - 0.8403)	0.7739 (0.5273 - 0.8233)	0.7168 (0.6128 - 0.7673)	0.6441 (0.6061 - 0.7730)	0.6365 (0.5930 - 0.7324)
Aortic (valve) insufficiency	0.6613 (0.6132 - 0.7049)	0.6494 (0.6129 - 0.6868)	0.6554 (0.5613 - 0.7634)	0.6433 (0.5125 - 0.6932)	0.6476 (0.5991 - 0.6941)	0.6512 (0.5947 - 0.7158)	0.6515 (0.5939 - 0.7038)	0.6951 (0.6508 - 0.7351)	0.6600 (0.6239 - 0.7016)	0.5405 (0.4114 - 0.6667)	0.7795 (0.7141 - 0.8767)	0.7102 (0.6427 - 0.7860)	0.6291 (0.5793 - 0.6930)	0.6139 (0.5317 - 0.6838)	0.6628 (0.6130 - 0.7111)	0.6330 (0.6030 - 0.6838)	0.5270 (0.3862 - 0.7731)	0.7391 (0.4590 - 0.8554)	0.6688 (0.5891 - 0.7581)	0.6098 (0.5629 - 0.6999)	0.5895 (0.5005 - 0.6770)
Heart failure, unspecified	0.7336 (0.6831 - 0.7814)	0.7022 (0.6641 - 0.7449)	0.5755 (0.4967 - 0.7444)	0.8289 (0.6457 - 0.8541)	0.7709 (0.6697 - 0.8108)	0.6614 (0.6130 - 0.7445)	0.6590 (0.6048 - 0.7296)	0.7962 (0.7593 - 0.8319)	0.7338 (0.7118 - 0.7702)	0.8201 (0.6268 - 0.9173)	0.6475 (0.5573 - 0.8545)	0.6994 (0.6501 - 0.8161)	0.7826 (0.6945 - 0.8838)	0.7550 (0.7008 - 0.7972)	0.7461 (0.7063 - 0.7830)	0.6966 (0.6675 - 0.7376)	0.6835 (0.5691 - 0.8359)	0.7097 (0.5649 - 0.8148)	0.7019 (0.6315 - 0.7690)	0.6915 (0.6382 - 0.7918)	0.6925 (0.6352 - 0.7517)
Cerebrovascular disease, unspecified	0.6966 (0.6473 - 0.7474)	0.6823 (0.6467 - 0.7302)	0.5812 (0.4690 - 0.6871)	0.7834 (0.7334 - 0.8638)	0.7285 (0.6723 - 0.8008)	0.6516 (0.5984 - 0.7251)	0.6466 (0.5738 - 0.7090)	0.7656 (0.7229 - 0.8070)	0.7211 (0.6847 - 0.7685)	0.6239 (0.5391 - 0.7986)	0.8183 (0.6527 - 0.8596)	0.7745 (0.6767 - 0.8234)	0.6851 (0.6348 - 0.7832)	0.6911 (0.6328 - 0.7664)	0.7396 (0.6932 - 0.7839)	0.7112 (0.6719 - 0.7584)	0.6410 (0.5571 - 0.7844)	0.7814 (0.6106 - 0.8249)	0.7457 (0.6642 - 0.7880)	0.6852 (0.6313 - 0.7738)	0.6894 (0.6314 - 0.7536)
Mitral (valve) prolapse	0.5922 (0.5398 - 0.6433)	0.5810 (0.5495 - 0.6304)	0.6015 (0.3418 - 0.7500)	0.5605 (0.4409 - 0.7953)	0.5778 (0.5380 - 0.6817)	0.5845 (0.5228 - 0.6722)	0.5894 (0.4409 - 0.6643)	0.6161 (0.5650 - 0.6644)	0.6030 (0.5731 - 0.6502)	0.4812 (0.3478 - 0.7912)	0.7247 (0.4542 - 0.8249)	0.6361 (0.5505 - 0.7172)	0.5828 (0.5407 - 0.7035)	0.5479 (0.4539 - 0.6799)	0.5973 (0.5493 - 0.6433)	0.5890 (0.5611 - 0.6316)	0.7744 (0.3811 - 0.8561)	0.4035 (0.3484 - 0.8062)	0.5649 (0.5218 - 0.6566)	0.6414 (0.5520 - 0.7342)	0.6533 (0.4799 - 0.6961)
Subsequent myocardial infarction of unspecified site	0.8319 (0.7895 - 0.8726)	0.7919 (0.7615 - 0.8289)	0.7724 (0.7087 - 0.8810)	0.8114 (0.7141 - 0.8360)	0.8037 (0.7365 - 0.8355)	0.7809 (0.7349 - 0.8693)	0.7877 (0.7464 - 0.8355)	0.8681 (0.8333 - 0.9003)	0.8126 (0.7900 - 0.8435)	0.8780 (0.7692 - 0.9528)	0.7472 (0.7020 - 0.8785)	0.7764 (0.7309 - 0.8611)	0.8597 (0.7835 - 0.9357)	0.8241 (0.7897 - 0.8582)	0.8374 (0.7984 - 0.8753)	0.7876 (0.7563 - 0.8276)	0.7480 (0.6891 - 0.8834)	0.8272 (0.6940 - 0.8552)	0.8124 (0.7266 - 0.8428)	0.7665 (0.7198 - 0.8684)	0.7788 (0.7415 - 0.8315)
Disease of pericardium, unspecified	0.6115 (0.5583 -	0.6076 (0.5700 -	0.6055 (0.4587 -	0.6098 (0.4609 -	0.6081 (0.5491 -	0.6072 (0.5495 -	0.6068 (0.5190 -	0.6235 (0.5698 -	0.6061 (0.5704 -	0.5596 (0.4099 -	0.6526 (0.3812 -	0.6170 (0.5488 -	0.5971 (0.5431 -	0.5869 (0.4915 -	0.5951 (0.5449 -	0.5910 (0.5589 -	0.6055 (0.3400 -	0.5766 (0.3731 -	0.5885 (0.5302 -	0.5938 (0.5338 -	0.5969 (0.4413 -

	0.6651)	0.6593)	0.7670)	0.7300)	0.6752)	0.6950)	0.6815)	0.6709)	0.6584)	0.8258)	0.7596)	0.6940)	0.7068)	0.6807)	0.6505)	0.6452)	0.8291)	0.8213)	0.6816)	0.7040)	0.6839)
Embolism and thrombosis of arteries of the lower extremities	0.7176 (0.6668 - 0.7705)	0.6826 (0.6489 - 0.7308)	0.7000 (0.5000 - 0.7870)	0.6652 (0.6303 - 0.8414)	0.6765 (0.6319 - 0.7850)	0.6892 (0.6136 - 0.7613)	0.6880 (0.5966 - 0.7438)	0.8001 (0.7567 - 0.8375)	0.7312 (0.6996 - 0.7774)	0.8455 (0.5841 - 0.9018)	0.6169 (0.6154 - 0.8727)	0.6882 (0.6506 - 0.8278)	0.7997 (0.6629 - 0.8717)	0.7587 (0.6702 - 0.7973)	0.7621 (0.7182 - 0.8028)	0.7104 (0.6803 - 0.7582)	0.8000 (0.5940 - 0.9149)	0.6209 (0.4958 - 0.8239)	0.6785 (0.6268 - 0.7822)	0.7564 (0.6585 - 0.8623)	0.7342 (0.6598 - 0.7826)
Other forms of acute ischaemic heart disease	0.7899 (0.7330 - 0.8439)	0.7377 (0.6995 - 0.7863)	0.7263 (0.5368 - 0.8193)	0.7491 (0.7221 - 0.9056)	0.7433 (0.6953 - 0.8711)	0.7324 (0.6452 - 0.8087)	0.7347 (0.6536 - 0.7901)	0.8709 (0.8309 - 0.9055)	0.8207 (0.7817 - 0.8577)	0.8421 (0.7613 - 0.9177)	0.7992 (0.7521 - 0.8358)	0.8075 (0.7562 - 0.8422)	0.8350 (0.7684 - 0.9107)	0.8244 (0.7760 - 0.8657)	0.8254 (0.7799 - 0.8685)	0.7828 (0.7446 - 0.8232)	0.8000 (0.6699 - 0.8903)	0.7655 (0.6732 - 0.8681)	0.7733 (0.7161 - 0.8446)	0.7929 (0.7115 - 0.8791)	0.7864 (0.7299 - 0.8323)
Other specified cardiac arrhythmias	0.6315 (0.5816 - 0.6846)	0.6341 (0.5925 - 0.6781)	0.7778 (0.5306 - 0.8736)	0.4905 (0.4093 - 0.7480)	0.6042 (0.5503 - 0.6627)	0.6882 (0.5979 - 0.7907)	0.6801 (0.5865 - 0.7317)	0.6083 (0.5543 - 0.6652)	0.5962 (0.5671 - 0.6542)	0.4444 (0.3367 - 0.8470)	0.7480 (0.3690 - 0.8370)	0.6382 (0.5344 - 0.7287)	0.5738 (0.5362 - 0.7266)	0.5240 (0.4456 - 0.6962)	0.6128 (0.5581 - 0.6659)	0.5968 (0.5661 - 0.6514)	0.5152 (0.3659 - 0.8265)	0.6785 (0.4046 - 0.8219)	0.6157 (0.5348 - 0.6994)	0.5832 (0.5405 - 0.7211)	0.5610 (0.4693 - 0.6957)
Naevus, nonneoplastic	0.5000 (0.4269 - 0.5663)	0.5226 (0.4850 - 0.5937)	0.6716 (0.2111 - 1.0000)	0.3735 (0.0527 - 0.8784)	0.5174 (0.4643 - 0.6354)	0.5321 (0.4846 - 1.0000)	0.5845 (0.3085 - 0.7029)	0.5484 (0.4835 - 0.6192)	0.5662 (0.5190 - 0.6346)	0.2985 (0.2192 - 0.9661)	0.8339 (0.1416 - 0.8765)	0.6425 (0.4961 - 0.7314)	0.5431 (0.4895 - 0.8195)	0.4076 (0.3269 - 0.6899)	0.5480 (0.4822 - 0.6177)	0.5590 (0.5178 - 0.6277)	0.2836 (0.1773 - 0.8308)	0.8345 (0.3052 - 0.9123)	0.6314 (0.4927 - 0.7303)	0.5381 (0.4867 - 0.6927)	0.3914 (0.2768 - 0.6777)
Atrioventricular block, complete	0.6631 (0.6066 - 0.7168)	0.6369 (0.6038 - 0.6942)	0.5464 (0.4184 - 0.7911)	0.7275 (0.5108 - 0.8274)	0.6672 (0.5797 - 0.7580)	0.6159 (0.5622 - 0.7304)	0.6008 (0.5186 - 0.7027)	0.7131 (0.6585 - 0.7679)	0.6922 (0.6543 - 0.7484)	0.6392 (0.4900 - 0.7629)	0.7452 (0.6355 - 0.8555)	0.7150 (0.6522 - 0.8086)	0.6738 (0.6008 - 0.7586)	0.6750 (0.5910 - 0.7413)	0.6912 (0.6303 - 0.7492)	0.6828 (0.6443 - 0.7358)	0.6701 (0.5158 - 0.7523)	0.6956 (0.6943 - 0.7942)	0.6876 (0.6484 - 0.7693)	0.6783 (0.6050 - 0.7547)	0.6788 (0.5932 - 0.7364)
Acute transmural myocardial infarction of other sites	0.8275 (0.7771 - 0.8674)	0.7695 (0.7357 - 0.8122)	0.7835 (0.6429 - 0.8953)	0.7554 (0.6469 - 0.8683)	0.7621 (0.7009 - 0.8471)	0.7772 (0.6961 - 0.8694)	0.7727 (0.7128 - 0.8215)	0.8912 (0.8631 - 0.9153)	0.8333 (0.8080 - 0.8681)	0.9175 (0.8105 - 0.9643)	0.7491 (0.7478 - 0.8611)	0.7852 (0.7547 - 0.8589)	0.9008 (0.8120 - 0.9603)	0.8462 (0.8072 - 0.8798)	0.8708 (0.8376 - 0.8981)	0.8205 (0.7928 - 0.8538)	0.8866 (0.7894 - 0.9600)	0.7545 (0.6727 - 0.8391)	0.7831 (0.7266 - 0.8429)	0.8693 (0.7947 - 0.9455)	0.8317 (0.7927 - 0.8688)
Atrioventricular block, first degree	0.6076 (0.5435 -	0.6122 (0.5696 -	0.4742 (0.3534 -	0.7502 (0.5780 -	0.6550 (0.5743 -	0.5879 (0.5326 -	0.5501 (0.4590 -	0.6824 (0.6277 -	0.6424 (0.6071 -	0.5052 (0.4363 -	0.7796 (0.4333 -	0.6963 (0.5713 -	0.6117 (0.5743 -	0.5855 (0.5323 -	0.6416 (0.5861 -	0.6106 (0.5794 -	0.4330 (0.3690 -	0.7882 (0.4134 -	0.6716 (0.5407 -	0.5816 (0.5447 -	0.5265 (0.4801 -

	0.6688)	0.6707)	0.6436)	0.8150)	0.7357)	0.6730)	0.6510)	0.7317)	0.6967)	0.8384)	0.8207)	0.7610)	0.7552)	0.7144)	0.6973)	0.6706)	0.8762)	0.8546)	0.7408)	0.7704)	0.7223)
Other ill-defined heart diseases	0.7388 (0.6800 - 0.7898)	0.6920 (0.6556 - 0.7427)	0.5941 (0.4651 - 0.7958)	0.7898 (0.6468 - 0.8844)	0.7387 (0.6426 - 0.8329)	0.6605 (0.6021 - 0.7663)	0.6585 (0.5789 - 0.7435)	0.8158 (0.7713 - 0.8570)	0.7578 (0.7220 - 0.8062)	0.6634 (0.5946 - 0.8571)	0.8522 (0.6942 - 0.8972)	0.8178 (0.7001 - 0.8662)	0.7168 (0.6719 - 0.8387)	0.7325 (0.6892 - 0.8062)	0.8068 (0.7585 - 0.8499)	0.7507 (0.7123 - 0.7998)	0.6931 (0.5856 - 0.8571)	0.8083 (0.6127 - 0.8838)	0.7834 (0.6811 - 0.8480)	0.7248 (0.6657 - 0.8201)	0.7355 (0.6766 - 0.7931)
Cardiomyopathy, unspecified	0.6655 (0.6110 - 0.7212)	0.6348 (0.5999 - 0.6930)	0.6087 (0.4318 - 0.8556)	0.6610 (0.3831 - 0.8108)	0.6423 (0.5736 - 0.7322)	0.6281 (0.5617 - 0.7535)	0.6250 (0.5357 - 0.7133)	0.7422 (0.6819 - 0.7919)	0.7038 (0.6696 - 0.7472)	0.8478 (0.5795 - 0.9205)	0.5599 (0.5055 - 0.8286)	0.6583 (0.6110 - 0.7821)	0.7863 (0.6547 - 0.8803)	0.7411 (0.6571 - 0.7853)	0.7007 (0.6521 - 0.7490)	0.6793 (0.6444 - 0.7256)	0.8152 (0.6598 - 0.8750)	0.5433 (0.5415 - 0.6863)	0.6409 (0.5943 - 0.7117)	0.7462 (0.6476 - 0.8212)	0.7177 (0.6495 - 0.7635)
Abdominal aortic aneurysm, without mention of rupture	0.7832 (0.7256 - 0.8331)	0.7436 (0.7027 - 0.7885)	0.7159 (0.5930 - 0.8283)	0.7713 (0.7312 - 0.8847)	0.7579 (0.6907 - 0.8442)	0.7308 (0.6663 - 0.8218)	0.7363 (0.6710 - 0.7946)	0.8127 (0.7574 - 0.8635)	0.7777 (0.7354 - 0.8248)	0.7500 (0.6216 - 0.8352)	0.8053 (0.7361 - 0.8982)	0.7939 (0.7475 - 0.8658)	0.7631 (0.6850 - 0.8380)	0.7713 (0.7060 - 0.8269)	0.7864 (0.7316 - 0.8375)	0.7634 (0.7221 - 0.8153)	0.7386 (0.6288 - 0.8290)	0.7882 (0.7517 - 0.8663)	0.7772 (0.7400 - 0.8406)	0.7510 (0.6750 - 0.8337)	0.7574 (0.6948 - 0.8168)
Intracerebral haemorrhage, unspecified	0.6007 (0.5429 - 0.6642)	0.5900 (0.5532 - 0.6522)	0.6395 (0.3292 - 0.9467)	0.5404 (0.1636 - 0.8213)	0.5818 (0.5165 - 0.7081)	0.5999 (0.5300 - 0.7780)	0.6093 (0.4318 - 0.6947)	0.5660 (0.5090 - 0.6194)	0.5820 (0.5471 - 0.6336)	0.6977 (0.6316 - 0.9659)	0.4664 (0.2096 - 0.4826)	0.5666 (0.5016 - 0.6256)	0.6067 (0.5637 - 0.8586)	0.6254 (0.5798 - 0.7184)	0.5127 (0.4604 - 0.5648)	0.5467 (0.5140 - 0.6040)	0.7326 (0.5217 - 0.8368)	0.3609 (0.2934 - 0.5496)	0.5341 (0.4744 - 0.6039)	0.5743 (0.4998 - 0.6988)	0.6177 (0.5115 - 0.6791)

b. Based on metabolome combined with clinical data

	LR							XGBoost							RF						
**Disease**	**AUC (95% CI)**	**Acc (95% CI)**	**Sn (95% CI)**	**Sp (95% CI)**	**PPV (95% CI)**	**NPV (95% CI)**	**F1 score (95% CI)**	**AUC (95% CI)**	**Acc (95% CI)**	**Sn (95% CI)**	**Sp (95% CI)**	**PPV (95% CI)**	**NPV (95% CI)**	**F1 score (95% CI)**	**AUC (95% CI)**	**Acc (95% CI)**	**Sn (95% CI)**	**Sp (95% CI)**	**PPV (95% CI)**	**NPV (95% CI)**	**F1 score (95% CI)**
Essential (primary) hypertension	0.8184 (0.8154 - 0.8215)	0.7464 (0.7435 - 0.7501)	0.7397 (0.7345 - 0.7804)	0.7531 (0.7121 - 0.7570)	0.7497 (0.7300 - 0.7538)	0.7432 (0.7389 - 0.7647)	0.7447 (0.7417 - 0.7557)	0.8067 (0.8032 - 0.8100)	0.7335 (0.7306 - 0.7375)	0.7201 (0.7078 - 0.7746)	0.7469 (0.6924 - 0.7574)	0.7399 (0.7151 - 0.7473)	0.7274 (0.7210 - 0.7556)	0.7299 (0.7250 - 0.7449)	0.8000 (0.7966 - 0.8031)	0.7296 (0.7265 - 0.7332)	0.7523 (0.6987 - 0.7650)	0.7069 (0.6947 - 0.7607)	0.7196 (0.7136 - 0.7448)	0.7405 (0.7154 - 0.7480)	0.7356 (0.7200 - 0.7404)

Chronic ischaemic heart disease	0.9007 (0.8972 - 0.9042)	0.8356 (0.8318 - 0.8400)	0.8555 (0.8389 - 0.8807)	0.8157 (0.7931 - 0.8332)	0.8228 (0.8075 - 0.8346)	0.8495 (0.8366 - 0.8694)	0.8388 (0.8339 - 0.8451)	0.8894 (0.8858 - 0.8926)	0.8195 (0.8154 - 0.8237)	0.8413 (0.8295 - 0.8606)	0.7977 (0.7796 - 0.8086)	0.8062 (0.7932 - 0.8128)	0.8340 (0.8248 - 0.8484)	0.8233 (0.8182 - 0.8288)	0.8818 (0.8781 - 0.8857)	0.8156 (0.8116 - 0.8206)	0.8357 (0.8054 - 0.8515)	0.7955 (0.7820 - 0.8237)	0.8034 (0.7945 - 0.8232)	0.8288 (0.8079 - 0.8409)	0.8192 (0.8118 - 0.8249)
Angina pectoris	0.8719 (0.8671 - 0.8770)	0.8056 (0.8010 - 0.8112)	0.8107 (0.7990 - 0.8394)	0.8004 (0.7746 - 0.8084)	0.8025 (0.7850 - 0.8090)	0.8087 (0.7999 - 0.8295)	0.8065 (0.8009 - 0.8151)	0.8617 (0.8577 - 0.8661)	0.7913 (0.7869 - 0.7967)	0.7925 (0.7821 - 0.8268)	0.7902 (0.7553 - 0.7981)	0.7907 (0.7698 - 0.7972)	0.7920 (0.7843 - 0.8143)	0.7916 (0.7864 - 0.8011)	0.8511 (0.8461 - 0.8559)	0.7872 (0.7827 - 0.7928)	0.7918 (0.7682 - 0.8134)	0.7826 (0.7647 - 0.8071)	0.7846 (0.7721 - 0.7995)	0.7899 (0.7751 - 0.8049)	0.7882 (0.7816 - 0.7964)
Atrial fibrillation and flutter	0.8213 (0.8130 - 0.8288)	0.7502 (0.7422 - 0.7586)	0.7290 (0.7053 - 0.7628)	0.7714 (0.7338 - 0.7929)	0.7613 (0.7420 - 0.7740)	0.7400 (0.7246 - 0.7596)	0.7448 (0.7344 - 0.7574)	0.8141 (0.8056 - 0.8218)	0.7413 (0.7341 - 0.7502)	0.7574 (0.7254 - 0.7841)	0.7253 (0.6976 - 0.7497)	0.7339 (0.7196 - 0.7514)	0.7493 (0.7295 - 0.7680)	0.7454 (0.7332 - 0.7569)	0.7967 (0.7885 - 0.8052)	0.7291 (0.7218 - 0.7379)	0.7243 (0.6717 - 0.7794)	0.7340 (0.6822 - 0.7864)	0.7314 (0.7047 - 0.7611)	0.7269 (0.7024 - 0.7566)	0.7278 (0.7105 - 0.7448)
Acute myocardial infarction	0.9125 (0.9076 - 0.9179)	0.8504 (0.8457 - 0.8575)	0.9119 (0.8630 - 0.9207)	0.7890 (0.7878 - 0.8374)	0.8121 (0.8069 - 0.8434)	0.8995 (0.8577 - 0.9102)	0.8591 (0.8495 - 0.8651)	0.9033 (0.8984 - 0.9079)	0.8357 (0.8299 - 0.8431)	0.8670 (0.8513 - 0.9014)	0.8044 (0.7748 - 0.8150)	0.8159 (0.7958 - 0.8255)	0.8581 (0.8450 - 0.8875)	0.8407 (0.8339 - 0.8509)	0.8946 (0.8887 - 0.9000)	0.8309 (0.8249 - 0.8383)	0.8565 (0.8421 - 0.8937)	0.8052 (0.7751 - 0.8147)	0.8147 (0.7939 - 0.8248)	0.8487 (0.8362 - 0.8812)	0.8351 (0.8285 - 0.8463)
Heart failure	0.8855 (0.8736 - 0.8958)	0.8148 (0.8033 - 0.8275)	0.7878 (0.7619 - 0.8367)	0.8417 (0.8087 - 0.8579)	0.8327 (0.8026 - 0.8491)	0.7987 (0.7782 - 0.8309)	0.8096 (0.7949 - 0.8269)	0.8921 (0.8816 - 0.9014)	0.8178 (0.8066 - 0.8295)	0.8424 (0.8047 - 0.8784)	0.7931 (0.7594 - 0.8253)	0.8029 (0.7815 - 0.8254)	0.8343 (0.8060 - 0.8641)	0.8222 (0.8072 - 0.8369)	0.8680 (0.8576 - 0.8789)	0.8016 (0.7910 - 0.8141)	0.8466 (0.7886 - 0.8843)	0.7566 (0.7174 - 0.8088)	0.7767 (0.7539 - 0.8099)	0.8315 (0.7931 - 0.8629)	0.8102 (0.7947 - 0.8244)
Paroxysmal tachycardia	0.6633 (0.6443 - 0.6833)	0.6339 (0.6200 - 0.6531)	0.5897 (0.4937 - 0.6418)	0.6781 (0.6372 - 0.7780)	0.6469 (0.6228 - 0.6882)	0.6231 (0.5955 - 0.6497)	0.6170 (0.5704 - 0.6478)	0.6844 (0.6651 - 0.7030)	0.6389 (0.6254 - 0.6570)	0.6044 (0.5129 - 0.7285)	0.6735 (0.5560 - 0.7713)	0.6493 (0.6100 - 0.6851)	0.6300 (0.6049 - 0.6789)	0.6260 (0.5817 - 0.6726)	0.6505 (0.6313 - 0.6700)	0.6134 (0.5996 - 0.6312)	0.5470 (0.4677 - 0.7007)	0.6798 (0.5372 - 0.7522)	0.6307 (0.5885 - 0.6625)	0.6001 (0.5773 - 0.6457)	0.5859 (0.5423 - 0.6479)
Other peripheral vascular diseases	0.8267 (0.8106 - 0.8426)	0.7674 (0.7551 - 0.7827)	0.7786 (0.7001 - 0.8097)	0.7563 (0.7486 - 0.8320)	0.7616 (0.7476 - 0.8119)	0.7735 (0.7291 - 0.8003)	0.7700 (0.7441 - 0.7899)	0.8261 (0.8105 - 0.8416)	0.7626 (0.7492 - 0.7802)	0.7338 (0.6955 - 0.8125)	0.7914 (0.7161 - 0.8204)	0.7786 (0.7382 - 0.8036)	0.7483 (0.7206 - 0.7947)	0.7556 (0.7377 - 0.7814)	0.8035 (0.7879 - 0.8184)	0.7454 (0.7320 - 0.7610)	0.7512 (0.7156 - 0.7990)	0.7395 (0.6992 - 0.7605)	0.7425 (0.7196 - 0.7619)	0.7483 (0.7229 - 0.7813)	0.7469 (0.7278 - 0.7698)

Pulmonary embolism	0.6816 (0.6607 - 0.7026)	0.6407 (0.6249 - 0.6614)	0.5604 (0.5169 - 0.7151)	0.7210 (0.5755 - 0.7589)	0.6676 (0.6143 - 0.6940)	0.6212 (0.6020 - 0.6762)	0.6093 (0.5821 - 0.6705)	0.7118 (0.6925 - 0.7303)	0.6611 (0.6470 - 0.6794)	0.6811 (0.6218 - 0.8308)	0.6412 (0.4919 - 0.7123)	0.6550 (0.6114 - 0.6822)	0.6678 (0.6427 - 0.7463)	0.6678 (0.6429 - 0.7171)	0.6854 (0.6654 - 0.7041)	0.6416 (0.6287 - 0.6617)	0.6170 (0.5336 - 0.7555)	0.6661 (0.5247 - 0.7401)	0.6489 (0.6055 - 0.6834)	0.6349 (0.6082 - 0.6909)	0.6325 (0.5938 - 0.6835)
Transient cerebral ischaemic attacks and related syndromes	0.7523 (0.7325 - 0.7722)	0.6958 (0.6815 - 0.7157)	0.7209 (0.6189 - 0.7792)	0.6708 (0.6275 - 0.7755)	0.6865 (0.6596 - 0.7367)	0.7062 (0.6655 - 0.7507)	0.7033 (0.6692 - 0.7311)	0.7752 (0.7567 - 0.7935)	0.7185 (0.7026 - 0.7380)	0.7295 (0.6541 - 0.7869)	0.7075 (0.6623 - 0.7771)	0.7138 (0.6859 - 0.7524)	0.7234 (0.6867 - 0.7621)	0.7215 (0.6912 - 0.7473)	0.7363 (0.7149 - 0.7582)	0.6945 (0.6790 - 0.7160)	0.6764 (0.6022 - 0.7414)	0.7127 (0.6380 - 0.7773)	0.7019 (0.6689 - 0.7444)	0.6877 (0.6530 - 0.7250)	0.6889 (0.6542 - 0.7167)
Complications and ill-defined descriptions of heart disease	0.7577 (0.7355 - 0.7832)	0.7161 (0.6994 - 0.7380)	0.6685 (0.5985 - 0.7220)	0.7636 (0.7377 - 0.8306)	0.7388 (0.7134 - 0.7848)	0.6973 (0.6635 - 0.7318)	0.7019 (0.6704 - 0.7329)	0.7981 (0.7795 - 0.8166)	0.7446 (0.7268 - 0.7629)	0.7106 (0.6672 - 0.7523)	0.7785 (0.7453 - 0.8036)	0.7624 (0.7378 - 0.7836)	0.7290 (0.6977 - 0.7599)	0.7356 (0.7104 - 0.7600)	0.7625 (0.7407 - 0.7844)	0.7115 (0.6960 - 0.7345)	0.6172 (0.5933 - 0.7699)	0.8058 (0.6562 - 0.8155)	0.7607 (0.6843 - 0.7796)	0.6779 (0.6624 - 0.7464)	0.6815 (0.6649 - 0.7363)
Cerebral infarction	0.8098 (0.7900 - 0.8303)	0.7518 (0.7363 - 0.7728)	0.7237 (0.6977 - 0.8299)	0.7799 (0.6891 - 0.7982)	0.7668 (0.7116 - 0.7843)	0.7384 (0.7172 - 0.8039)	0.7446 (0.7269 - 0.7786)	0.8259 (0.8084 - 0.8431)	0.7596 (0.7442 - 0.7786)	0.7801 (0.6790 - 0.8250)	0.7392 (0.7127 - 0.8388)	0.7494 (0.7260 - 0.8092)	0.7707 (0.7201 - 0.8076)	0.7645 (0.7307 - 0.7867)	0.8032 (0.7848 - 0.8213)	0.7335 (0.7183 - 0.7531)	0.7049 (0.6667 - 0.8423)	0.7621 (0.6329 - 0.7939)	0.7476 (0.6831 - 0.7741)	0.7208 (0.6940 - 0.8026)	0.7256 (0.7070 - 0.7654)
Other cardiac arrhythmias	0.6814 (0.6567 - 0.7073)	0.6469 (0.6298 - 0.6719)	0.5456 (0.4787 - 0.6957)	0.7481 (0.6016 - 0.8295)	0.6841 (0.6324 - 0.7335)	0.6221 (0.5954 - 0.6689)	0.6071 (0.5728 - 0.6684)	0.7118 (0.6882 - 0.7332)	0.6661 (0.6484 - 0.6880)	0.6488 (0.5325 - 0.7574)	0.6833 (0.5825 - 0.7921)	0.6720 (0.6302 - 0.7294)	0.6605 (0.6214 - 0.7098)	0.6602 (0.6100 - 0.7010)	0.6787 (0.6545 - 0.7029)	0.6414 (0.6233 - 0.6654)	0.5496 (0.5150 - 0.7518)	0.7331 (0.5407 - 0.7603)	0.6731 (0.6036 - 0.7000)	0.6194 (0.5967 - 0.6932)	0.6051 (0.5815 - 0.6849)
Atrioventricular and left bundle-branch block	0.7345 (0.7110 - 0.7572)	0.6862 (0.6709 - 0.7098)	0.7140 (0.5375 - 0.7485)	0.6583 (0.6542 - 0.8172)	0.6764 (0.6610 - 0.7532)	0.6971 (0.6407 - 0.7306)	0.6947 (0.6314 - 0.7178)	0.7702 (0.7491 - 0.7930)	0.7188 (0.7011 - 0.7406)	0.7060 (0.6255 - 0.7664)	0.7317 (0.6790 - 0.8032)	0.7246 (0.6913 - 0.7651)	0.7134 (0.6801 - 0.7570)	0.7152 (0.6832 - 0.7445)	0.7394 (0.7162 - 0.7641)	0.6922 (0.6772 - 0.7166)	0.6420 (0.5612 - 0.7778)	0.7424 (0.6139 - 0.8098)	0.7136 (0.6563 - 0.7619)	0.6747 (0.6416 - 0.7380)	0.6759 (0.6394 - 0.7207)
Nonrheumatic mitral valve disorders	0.7286 (0.7017 - 0.7522)	0.6857 (0.6649 - 0.7076)	0.6282 (0.5639 - 0.6974)	0.7431 (0.6737 - 0.8077)	0.7098 (0.6731 - 0.7482)	0.6665 (0.6361 - 0.7052)	0.6665 (0.6320 - 0.7013)	0.7493 (0.7267 - 0.7708)	0.6883 (0.6717 - 0.7114)	0.7244 (0.5483 - 0.7744)	0.6522 (0.6291 - 0.8120)	0.6756 (0.6513 - 0.7602)	0.7029 (0.6377 - 0.7425)	0.6992 (0.6325 - 0.7251)	0.7082 (0.6838 - 0.7343)	0.6688 (0.6501 - 0.6926)	0.6560 (0.5507 - 0.7126)	0.6815 (0.6180 - 0.7762)	0.6732 (0.6465 - 0.7235)	0.6646 (0.6249 - 0.7019)	0.6645 (0.6155 - 0.6963)

Nonrheumatic aortic valve disorders	0.7859 (0.7632 - 0.8098)	0.7329 (0.7152 - 0.7589)	0.6395 (0.5995 - 0.7956)	0.8263 (0.6564 - 0.8496)	0.7864 (0.6986 - 0.8171)	0.6962 (0.6704 - 0.7672)	0.7054 (0.6816 - 0.7524)	0.8200 (0.7980 - 0.8390)	0.7449 (0.7272 - 0.7676)	0.7210 (0.6384 - 0.8309)	0.7688 (0.6661 - 0.8411)	0.7572 (0.7034 - 0.8086)	0.7337 (0.6947 - 0.8023)	0.7387 (0.7056 - 0.7762)	0.7756 (0.7520 - 0.7977)	0.7139 (0.6963 - 0.7376)	0.7975 (0.6072 - 0.8320)	0.6303 (0.6260 - 0.8273)	0.6833 (0.6654 - 0.7802)	0.7569 (0.6718 - 0.7938)	0.7360 (0.6796 - 0.7583)
Other conduction disorders	0.6418 (0.6119 - 0.6710)	0.6143 (0.5932 - 0.6431)	0.5027 (0.4439 - 0.7061)	0.7259 (0.5466 - 0.7634)	0.6472 (0.5831 - 0.6872)	0.5935 (0.5643 - 0.6597)	0.5659 (0.5282 - 0.6575)	0.6813 (0.6517 - 0.7090)	0.6491 (0.6282 - 0.6769)	0.5302 (0.4934 - 0.6676)	0.7681 (0.6596 - 0.7738)	0.6957 (0.6362 - 0.7238)	0.6205 (0.5966 - 0.6759)	0.6018 (0.5743 - 0.6692)	0.6475 (0.6163 - 0.6771)	0.6192 (0.5978 - 0.6481)	0.5467 (0.4065 - 0.6593)	0.6917 (0.5815 - 0.8368)	0.6394 (0.5990 - 0.7012)	0.6041 (0.5660 - 0.6475)	0.5894 (0.5180 - 0.6392)
Other cerebrovascular diseases	0.6880 (0.6523 - 0.7231)	0.6629 (0.6396 - 0.6925)	0.6349 (0.4844 - 0.6963)	0.6908 (0.6681 - 0.8210)	0.6725 (0.6393 - 0.7515)	0.6543 (0.6069 - 0.6982)	0.6532 (0.5822 - 0.6934)	0.7366 (0.7065 - 0.7659)	0.6888 (0.6624 - 0.7180)	0.5556 (0.5066 - 0.7397)	0.8220 (0.6299 - 0.8285)	0.7573 (0.6596 - 0.7824)	0.6490 (0.6163 - 0.7164)	0.6409 (0.6004 - 0.7065)	0.6980 (0.6677 - 0.7276)	0.6575 (0.6334 - 0.6850)	0.5841 (0.5014 - 0.7144)	0.7308 (0.5922 - 0.8081)	0.6845 (0.6290 - 0.7326)	0.6373 (0.6022 - 0.6958)	0.6304 (0.5859 - 0.6809)
Other disorders of arteries and arterioles	0.7853 (0.7562 - 0.8122)	0.7418 (0.7188 - 0.7667)	0.7284 (0.6346 - 0.7749)	0.7553 (0.7481 - 0.8332)	0.7485 (0.7268 - 0.7990)	0.7355 (0.6872 - 0.7728)	0.7383 (0.6964 - 0.7697)	0.8314 (0.8079 - 0.8543)	0.7660 (0.7474 - 0.7912)	0.7747 (0.7160 - 0.8663)	0.7574 (0.6829 - 0.8111)	0.7615 (0.7127 - 0.8021)	0.7707 (0.7327 - 0.8435)	0.7680 (0.7429 - 0.8022)	0.8087 (0.7863 - 0.8307)	0.7421 (0.7237 - 0.7660)	0.7531 (0.6776 - 0.8889)	0.7311 (0.5899 - 0.8052)	0.7369 (0.6748 - 0.7881)	0.7475 (0.7033 - 0.8422)	0.7449 (0.7156 - 0.7806)
Hypertensive renal disease	0.8973 (0.8701 - 0.9188)	0.8416 (0.8177 - 0.8659)	0.7453 (0.6963 - 0.8182)	0.9378 (0.8814 - 0.9571)	0.9230 (0.8623 - 0.9467)	0.7864 (0.7543 - 0.8354)	0.8247 (0.7947 - 0.8565)	0.9442 (0.9303 - 0.9594)	0.8903 (0.8710 - 0.9106)	0.8385 (0.8018 - 0.8799)	0.9421 (0.9092 - 0.9472)	0.9355 (0.9094 - 0.9433)	0.8537 (0.8242 - 0.8889)	0.8843 (0.8623 - 0.9066)	0.9388 (0.9223 - 0.9532)	0.8840 (0.8652 - 0.9045)	0.8447 (0.7994 - 0.8875)	0.9234 (0.9063 - 0.9396)	0.9168 (0.9003 - 0.9350)	0.8560 (0.8223 - 0.8937)	0.8793 (0.8554 - 0.9019)
Stroke, not specified as haemorrhage or infarction	0.7810 (0.7512 - 0.8074)	0.7359 (0.7143 - 0.7635)	0.7550 (0.6615 - 0.8216)	0.7168 (0.6443 - 0.7890)	0.7272 (0.6928 - 0.7794)	0.7453 (0.6930 - 0.7974)	0.7409 (0.7014 - 0.7727)	0.8246 (0.8024 - 0.8463)	0.7635 (0.7446 - 0.7902)	0.7315 (0.6976 - 0.8514)	0.7954 (0.6827 - 0.8196)	0.7815 (0.7160 - 0.8043)	0.7477 (0.7184 - 0.8258)	0.7557 (0.7316 - 0.7950)	0.7998 (0.7754 - 0.8240)	0.7421 (0.7217 - 0.7686)	0.6946 (0.6547 - 0.8214)	0.7895 (0.6760 - 0.8169)	0.7675 (0.6997 - 0.7966)	0.7211 (0.6924 - 0.7985)	0.7292 (0.7053 - 0.7723)
Cardiomyopathy	0.7718 (0.7380 - 0.8029)	0.7154 (0.6920 - 0.7477)	0.5984 (0.5333 - 0.8083)	0.8325 (0.6163 - 0.8817)	0.7813 (0.6717 - 0.8349)	0.6746 (0.6432 - 0.7700)	0.6777 (0.6378 - 0.7443)	0.8337 (0.8094 - 0.8579)	0.7686 (0.7452 - 0.7964)	0.7756 (0.7059 - 0.8513)	0.7615 (0.7042 - 0.8217)	0.7648 (0.7234 - 0.8105)	0.7724 (0.7252 - 0.8321)	0.7702 (0.7381 - 0.8050)	0.7901 (0.7629 - 0.8170)	0.7299 (0.7089 - 0.7576)	0.8150 (0.6410 - 0.8631)	0.6449 (0.6191 - 0.8158)	0.6965 (0.6707 - 0.7817)	0.7771 (0.6903 - 0.8234)	0.7511 (0.6978 - 0.7798)

Sequelae of cerebrovascular disease	0.7619 (0.7245 - 0.7933)	0.7118 (0.6873 - 0.7433)	0.6000 (0.5584 - 0.8169)	0.8236 (0.6183 - 0.8318)	0.7728 (0.6623 - 0.7992)	0.6731 (0.6423 - 0.7860)	0.6755 (0.6437 - 0.7571)	0.7836 (0.7550 - 0.8143)	0.7335 (0.7096 - 0.7614)	0.7490 (0.6976 - 0.8284)	0.7180 (0.6512 - 0.7446)	0.7265 (0.6866 - 0.7555)	0.7410 (0.7009 - 0.8013)	0.7376 (0.7057 - 0.7745)	0.7511 (0.7219 - 0.7774)	0.7028 (0.6787 - 0.7327)	0.6745 (0.6314 - 0.8221)	0.7310 (0.5715 - 0.7489)	0.7149 (0.6458 - 0.7442)	0.6919 (0.6573 - 0.7780)	0.6941 (0.6645 - 0.7462)
Subarachnoid haemorrhage	0.5963 (0.5557 - 0.6357)	0.5880 (0.5626 - 0.6280)	0.4098 (0.2920 - 0.6208)	0.7662 (0.5767 - 0.8776)	0.6368 (0.5688 - 0.7206)	0.5649 (0.5335 - 0.6299)	0.4987 (0.4065 - 0.6155)	0.5642 (0.5266 - 0.6015)	0.5564 (0.5320 - 0.5921)	0.3811 (0.1905 - 0.8938)	0.7317 (0.2059 - 0.9001)	0.5869 (0.5127 - 0.6910)	0.5418 (0.5116 - 0.6663)	0.4622 (0.2970 - 0.6678)	0.5180 (0.4822 - 0.5551)	0.5251 (0.5047 - 0.5637)	0.2172 (0.1411 - 0.9646)	0.8330 (0.0799 - 0.9073)	0.5653 (0.4977 - 0.6579)	0.5155 (0.4904 - 0.6845)	0.3138 (0.2276 - 0.6628)
Atherosclerosis	0.8192 (0.7892 - 0.8497)	0.7614 (0.7355 - 0.7928)	0.6612 (0.6148 - 0.8055)	0.8615 (0.7087 - 0.8733)	0.8268 (0.7364 - 0.8492)	0.7177 (0.6828 - 0.7911)	0.7348 (0.7012 - 0.7842)	0.8540 (0.8291 - 0.8776)	0.8002 (0.7783 - 0.8274)	0.7714 (0.7194 - 0.8442)	0.8290 (0.7875 - 0.8566)	0.8186 (0.7770 - 0.8473)	0.7839 (0.7442 - 0.8395)	0.7943 (0.7655 - 0.8274)	0.8203 (0.7926 - 0.8480)	0.7762 (0.7536 - 0.8032)	0.7510 (0.7154 - 0.8361)	0.8014 (0.7571 - 0.8088)	0.7909 (0.7408 - 0.8152)	0.7630 (0.7303 - 0.8270)	0.7704 (0.7441 - 0.8088)
Subsequent myocardial infarction	0.8830 (0.8604 - 0.9021)	0.8186 (0.8005 - 0.8448)	0.8403 (0.7670 - 0.9107)	0.7969 (0.7228 - 0.8585)	0.8054 (0.7622 - 0.8521)	0.8331 (0.7814 - 0.8935)	0.8225 (0.7971 - 0.8517)	0.9050 (0.8888 - 0.9211)	0.8447 (0.8254 - 0.8673)	0.8655 (0.8211 - 0.9218)	0.8239 (0.7736 - 0.8563)	0.8310 (0.7935 - 0.8566)	0.8597 (0.8225 - 0.9116)	0.8479 (0.8227 - 0.8726)	0.8807 (0.8584 - 0.8999)	0.8275 (0.8081 - 0.8519)	0.8487 (0.8070 - 0.9133)	0.8063 (0.7672 - 0.8282)	0.8142 (0.7772 - 0.8387)	0.8420 (0.8058 - 0.9015)	0.8311 (0.8084 - 0.8595)
Occlusion and stenosis of precerebral arteries, not resulting in cerebral infarction	0.8150 (0.7845 - 0.8459)	0.7553 (0.7323 - 0.7881)	0.7864 (0.6814 - 0.8564)	0.7241 (0.6856 - 0.8244)	0.7403 (0.7028 - 0.8061)	0.7722 (0.7089 - 0.8311)	0.7627 (0.7222 - 0.7977)	0.8615 (0.8355 - 0.8851)	0.8032 (0.7827 - 0.8304)	0.8301 (0.7458 - 0.8953)	0.7763 (0.7166 - 0.8496)	0.7877 (0.7506 - 0.8429)	0.8204 (0.7657 - 0.8765)	0.8084 (0.7777 - 0.8386)	0.8431 (0.8175 - 0.8666)	0.7807 (0.7631 - 0.8109)	0.7816 (0.7368 - 0.9279)	0.7798 (0.6546 - 0.8189)	0.7802 (0.7080 - 0.8171)	0.7812 (0.7487 - 0.9003)	0.7809 (0.7565 - 0.8257)
Arterial embolism and thrombosis	0.7392 (0.6964 - 0.7771)	0.6993 (0.6694 - 0.7365)	0.5813 (0.5025 - 0.7500)	0.8173 (0.6630 - 0.8591)	0.7609 (0.6711 - 0.8056)	0.6612 (0.6207 - 0.7343)	0.6591 (0.6044 - 0.7268)	0.7944 (0.7624 - 0.8261)	0.7307 (0.7050 - 0.7675)	0.6601 (0.5867 - 0.8101)	0.8013 (0.6659 - 0.8553)	0.7686 (0.6868 - 0.8165)	0.7022 (0.6597 - 0.7902)	0.7102 (0.6718 - 0.7673)	0.7511 (0.7119 - 0.7860)	0.7142 (0.6875 - 0.7492)	0.6847 (0.6009 - 0.7832)	0.7437 (0.6645 - 0.8102)	0.7276 (0.6819 - 0.7756)	0.7023 (0.6554 - 0.7668)	0.7055 (0.6626 - 0.7522)
Other acute ischaemic	0.8383 (0.8065 -	0.7827 (0.7575 -	0.8158 (0.7048 -	0.7497 (0.6937 -	0.7652 (0.7276 -	0.8027 (0.7355 -	0.7897 (0.7484 -	0.8924 (0.8693 -	0.8364 (0.8163 -	0.9158 (0.8287 -	0.7570 (0.7555 -	0.7903 (0.7677 -	0.8999 (0.8277 -	0.8484 (0.8205 -	0.8767 (0.8536 -	0.8287 (0.8058 -	0.8474 (0.7978 -	0.8099 (0.7528 -	0.8168 (0.7692 -	0.8414 (0.7980 -	0.8318 (0.8046 -

heart diseases	0.8690)	0.8128)	0.8796)	0.8491)	0.8329)	0.8609)	0.8224)	0.9138)	0.8598)	0.9469)	0.8431)	0.8437)	0.9350)	0.8718)	0.8992)	0.8546)	0.9215)	0.8426)	0.8450)	0.9109)	0.8616)
Other diseases of pericardium	0.6312 (0.5918 - 0.6763)	0.6152 (0.5897 - 0.6564)	0.5579 (0.3313 - 0.6871)	0.6725 (0.5268 - 0.8781)	0.6301 (0.5892 - 0.7616)	0.6033 (0.5559 - 0.6624)	0.5918 (0.4582 - 0.6520)	0.6685 (0.6289 - 0.7081)	0.6378 (0.6142 - 0.6772)	0.6842 (0.4530 - 0.7950)	0.5913 (0.5066 - 0.8038)	0.6261 (0.5852 - 0.7239)	0.6519 (0.5833 - 0.7286)	0.6538 (0.5451 - 0.7070)	0.6440 (0.6049 - 0.6806)	0.6226 (0.5956 - 0.6581)	0.6526 (0.5225 - 0.7926)	0.5925 (0.4730 - 0.7160)	0.6156 (0.5717 - 0.6754)	0.6304 (0.5765 - 0.7098)	0.6336 (0.5642 - 0.6928)
Multiple valve diseases	0.7585 (0.7192 - 0.7980)	0.7207 (0.6914 - 0.7595)	0.6061 (0.5414 - 0.7553)	0.8353 (0.6957 - 0.8687)	0.7863 (0.7014 - 0.8199)	0.6795 (0.6425 - 0.7554)	0.6845 (0.6410 - 0.7481)	0.8119 (0.7765 - 0.8437)	0.7478 (0.7196 - 0.7810)	0.7374 (0.6298 - 0.8172)	0.7582 (0.7078 - 0.8607)	0.7531 (0.7127 - 0.8224)	0.7427 (0.6883 - 0.8038)	0.7451 (0.6949 - 0.7885)	0.7707 (0.7370 - 0.8066)	0.7197 (0.6955 - 0.7581)	0.7222 (0.5990 - 0.8341)	0.7172 (0.6224 - 0.8352)	0.7186 (0.6645 - 0.7949)	0.7208 (0.6655 - 0.8031)	0.7204 (0.6703 - 0.7680)
Aortic aneurysm and dissection	0.8317 (0.8011 - 0.8587)	0.7705 (0.7433 - 0.8034)	0.7676 (0.7189 - 0.8830)	0.7734 (0.6385 - 0.7970)	0.7721 (0.7130 - 0.8042)	0.7689 (0.7261 - 0.8546)	0.7698 (0.7392 - 0.8120)	0.8613 (0.8354 - 0.8864)	0.7966 (0.7711 - 0.8256)	0.8324 (0.7444 - 0.8878)	0.7607 (0.7588 - 0.8491)	0.7767 (0.7463 - 0.8334)	0.8195 (0.7587 - 0.8712)	0.8036 (0.7660 - 0.8354)	0.8192 (0.7864 - 0.8491)	0.7577 (0.7383 - 0.7910)	0.8541 (0.6716 - 0.8989)	0.6614 (0.6439 - 0.8396)	0.7161 (0.6880 - 0.8196)	0.8192 (0.7104 - 0.8700)	0.7790 (0.7275 - 0.8049)
Intracerebral haemorrhage	0.6517 (0.6023 - 0.7001)	0.6320 (0.5971 - 0.6738)	0.7121 (0.3916 - 0.8049)	0.5519 (0.5383 - 0.8527)	0.6138 (0.5703 - 0.7387)	0.6572 (0.5731 - 0.7298)	0.6593 (0.5094 - 0.7111)	0.6925 (0.6473 - 0.7418)	0.6536 (0.6242 - 0.7095)	0.7197 (0.4626 - 0.7907)	0.5874 (0.5658 - 0.8282)	0.6356 (0.6032 - 0.7594)	0.6770 (0.5860 - 0.7507)	0.6751 (0.5597 - 0.7204)	0.6555 (0.6064 - 0.7008)	0.6340 (0.6052 - 0.6808)	0.5758 (0.4621 - 0.7402)	0.6923 (0.5663 - 0.7806)	0.6517 (0.5929 - 0.7202)	0.6200 (0.5773 - 0.7064)	0.6114 (0.5428 - 0.6917)
Diseases of capillaries	0.5174 (0.4523 - 0.5787)	0.5469 (0.5077 - 0.6068)	0.3258 (0.1818 - 0.7073)	0.7679 (0.4154 - 0.8636)	0.5840 (0.4992 - 0.6751)	0.5325 (0.4857 - 0.6362)	0.4183 (0.2778 - 0.6284)	0.5736 (0.5121 - 0.6322)	0.5745 (0.5402 - 0.6322)	0.6067 (0.3529 - 0.8261)	0.5422 (0.3815 - 0.7643)	0.5700 (0.5126 - 0.6620)	0.5796 (0.5189 - 0.6914)	0.5878 (0.4412 - 0.6760)	0.5565 (0.4969 - 0.6158)	0.5744 (0.5336 - 0.6316)	0.5393 (0.3972 - 0.7875)	0.6096 (0.3333 - 0.6862)	0.5801 (0.5120 - 0.6510)	0.5696 (0.5174 - 0.6685)	0.5590 (0.4607 - 0.6536)
Cardiac arrest	0.7672 (0.7211 - 0.8117)	0.7075 (0.6772 - 0.7540)	0.7959 (0.5903 - 0.8824)	0.6190 (0.5874 - 0.7889)	0.6763 (0.6310 - 0.7771)	0.7521 (0.6526 - 0.8402)	0.7312 (0.6536 - 0.7777)	0.8458 (0.8054 - 0.8799)	0.8074 (0.7737 - 0.8493)	0.7959 (0.7475 - 0.9246)	0.8189 (0.7238 - 0.8378)	0.8146 (0.7358 - 0.8473)	0.8005 (0.7562 - 0.9117)	0.8052 (0.7697 - 0.8579)	0.8079 (0.7706 - 0.8441)	0.7623 (0.7265 - 0.8089)	0.7755 (0.6706 - 0.8903)	0.7490 (0.6356 - 0.8181)	0.7555 (0.6856 - 0.8124)	0.7694 (0.6981 - 0.8661)	0.7654 (0.7127 - 0.8158)
Other pulmonary heart diseases	0.6357 (0.5727 -	0.6300 (0.5913 -	0.4421 (0.3030 -	0.8179 (0.6488 -	0.7083 (0.6094 -	0.5945 (0.5445 -	0.5444 (0.4373 -	0.7454 (0.6901 -	0.7036 (0.6581 -	0.6000 (0.4821 -	0.8073 (0.5360 -	0.7569 (0.6338 -	0.6687 (0.6047 -	0.6694 (0.5888 -	0.7055 (0.6493 -	0.6613 (0.6234 -	0.5158 (0.4395 -	0.8068 (0.5487 -	0.7275 (0.6001 -	0.6249 (0.5784 -	0.6036 (0.5481 -

	0.7017)	0.6923)	0.6700)	0.9359)	0.8243)	0.6880)	0.6694)	0.7964)	0.7545)	0.8001)	0.8751)	0.8273)	0.7614)	0.7403)	0.7609)	0.7191)	0.8021)	0.8789)	0.7989)	0.7520)	0.7267)
Rheumatic mitral valve diseases	0.7204 (0.6565 - 0.7779)	0.6813 (0.6423 - 0.7371)	0.6333 (0.4197 - 0.7701)	0.7294 (0.5971 - 0.9473)	0.7006 (0.6343 - 0.8754)	0.6655 (0.5935 - 0.7543)	0.6653 (0.5562 - 0.7325)	0.7786 (0.7333 - 0.8226)	0.7181 (0.6853 - 0.7629)	0.8222 (0.6322 - 0.9158)	0.6139 (0.5235 - 0.7694)	0.6805 (0.6284 - 0.7701)	0.7755 (0.6686 - 0.8738)	0.7447 (0.6722 - 0.7917)	0.6972 (0.6446 - 0.7466)	0.6572 (0.6204 - 0.7036)	0.7556 (0.5654 - 0.9208)	0.5588 (0.4082 - 0.7762)	0.6313 (0.5621 - 0.7084)	0.6957 (0.6247 - 0.8469)	0.6879 (0.6144 - 0.7497)
Atherosclerotic heart disease	0.9133 (0.9099 - 0.9165)	0.8494 (0.8457 - 0.8541)	0.8861 (0.8577 - 0.9040)	0.8128 (0.7949 - 0.8410)	0.8256 (0.8147 - 0.8435)	0.8771 (0.8547 - 0.8924)	0.8548 (0.8487 - 0.8600)	0.9025 (0.8991 - 0.9061)	0.8341 (0.8298 - 0.8390)	0.8658 (0.8554 - 0.8793)	0.8024 (0.7945 - 0.8083)	0.8142 (0.8072 - 0.8202)	0.8567 (0.8478 - 0.8686)	0.8392 (0.8340 - 0.8454)	0.8951 (0.8908 - 0.8989)	0.8289 (0.8249 - 0.8342)	0.8422 (0.8189 - 0.8808)	0.8155 (0.7791 - 0.8388)	0.8203 (0.7971 - 0.8366)	0.8379 (0.8214 - 0.8687)	0.8311 (0.8246 - 0.8389)
Angina pectoris, unspecified	0.8747 (0.8701 - 0.8798)	0.8079 (0.8033 - 0.8138)	0.8167 (0.8049 - 0.8504)	0.7991 (0.7690 - 0.8091)	0.8026 (0.7832 - 0.8093)	0.8134 (0.8040 - 0.8390)	0.8096 (0.8040 - 0.8195)	0.8656 (0.8608 - 0.8703)	0.7949 (0.7898 - 0.8011)	0.7956 (0.7855 - 0.8326)	0.7943 (0.7596 - 0.7996)	0.7945 (0.7734 - 0.8007)	0.7953 (0.7862 - 0.8211)	0.7950 (0.7897 - 0.8065)	0.8515 (0.8462 - 0.8571)	0.7879 (0.7822 - 0.7943)	0.7880 (0.7719 - 0.8178)	0.7879 (0.7574 - 0.8018)	0.7879 (0.7704 - 0.7974)	0.7880 (0.7765 - 0.8076)	0.7880 (0.7805 - 0.7971)
Chronic ischaemic heart disease, unspecified	0.8742 (0.8668 - 0.8811)	0.8085 (0.8011 - 0.8177)	0.8311 (0.7923 - 0.8504)	0.7858 (0.7673 - 0.8259)	0.7951 (0.7829 - 0.8206)	0.8231 (0.7971 - 0.8402)	0.8127 (0.8013 - 0.8222)	0.8675 (0.8607 - 0.8744)	0.7986 (0.7918 - 0.8068)	0.8433 (0.8086 - 0.8607)	0.7539 (0.7392 - 0.7868)	0.7741 (0.7640 - 0.7939)	0.8279 (0.8026 - 0.8436)	0.8072 (0.7963 - 0.8158)	0.8503 (0.8429 - 0.8577)	0.7857 (0.7780 - 0.7943)	0.8148 (0.7712 - 0.8293)	0.7565 (0.7529 - 0.7986)	0.7699 (0.7627 - 0.7938)	0.8034 (0.7752 - 0.8169)	0.7917 (0.7777 - 0.8012)
Other forms of chronic ischaemic heart disease	0.9111 (0.9053 - 0.9171)	0.8492 (0.8421 - 0.8571)	0.8575 (0.8411 - 0.8875)	0.8409 (0.8114 - 0.8502)	0.8435 (0.8223 - 0.8527)	0.8551 (0.8411 - 0.8795)	0.8504 (0.8422 - 0.8596)	0.9032 (0.8977 - 0.9086)	0.8326 (0.8264 - 0.8406)	0.8813 (0.8333 - 0.8982)	0.7840 (0.7715 - 0.8380)	0.8031 (0.7935 - 0.8365)	0.8685 (0.8336 - 0.8849)	0.8404 (0.8295 - 0.8485)	0.8903 (0.8840 - 0.8967)	0.8269 (0.8200 - 0.8353)	0.8535 (0.8341 - 0.8888)	0.8003 (0.7651 - 0.8217)	0.8104 (0.7901 - 0.8226)	0.8452 (0.8294 - 0.8749)	0.8314 (0.8235 - 0.8426)
Unstable angina	0.8632 (0.8548 - 0.8713)	0.8013 (0.7930 - 0.8107)	0.8149 (0.7759 - 0.8369)	0.7876 (0.7719 - 0.8210)	0.7933 (0.7804 - 0.8164)	0.8097 (0.7834 - 0.8281)	0.8040 (0.7906 - 0.8147)	0.8609 (0.8526 - 0.8684)	0.7937 (0.7852 - 0.8035)	0.8077 (0.7930 - 0.8497)	0.7797 (0.7459 - 0.7856)	0.7857 (0.7621 - 0.7943)	0.8021 (0.7887 - 0.8325)	0.7965 (0.7865 - 0.8102)	0.8461 (0.8375 - 0.8550)	0.7888 (0.7810 - 0.7987)	0.7993 (0.7803 - 0.8429)	0.7783 (0.7405 - 0.7912)	0.7828 (0.7575 - 0.7948)	0.7950 (0.7792 - 0.8265)	0.7910 (0.7815 - 0.8041)
Old myocardial infarction	0.8813 (0.8720 -	0.8174 (0.8076 -	0.8361 (0.8032 -	0.7987 (0.7568 -	0.8059 (0.7780 -	0.8297 (0.8076 -	0.8207 (0.8071 -	0.8856 (0.8772 -	0.8169 (0.8066 -	0.8468 (0.8258 -	0.7869 (0.7587 -	0.7989 (0.7840 -	0.8371 (0.8172 -	0.8222 (0.8095 -	0.8664 (0.8560 -	0.8045 (0.7941 -	0.8517 (0.8000 -	0.7573 (0.7305 -	0.7782 (0.7603 -	0.8362 (0.7983 -	0.8133 (0.7964 -

	0.8915)	0.8296)	0.8786)	0.8288)	0.8256)	0.8660)	0.8358)	0.8941)	0.8277)	0.8738)	0.7954)	0.8112)	0.8616)	0.8355)	0.8767)	0.8162)	0.8807)	0.8033)	0.8095)	0.8615)	0.8270)
Acute myocardial infarction, unspecified	0.8890 (0.8783 - 0.8993)	0.8246 (0.8155 - 0.8384)	0.8494 (0.8208 - 0.9232)	0.7999 (0.7256 - 0.8282)	0.8093 (0.7684 - 0.8294)	0.8416 (0.8209 - 0.9041)	0.8289 (0.8171 - 0.8475)	0.8946 (0.8858 - 0.9039)	0.8298 (0.8197 - 0.8437)	0.8494 (0.8136 - 0.8974)	0.8101 (0.7613 - 0.8387)	0.8173 (0.7876 - 0.8394)	0.8432 (0.8160 - 0.8822)	0.8330 (0.8193 - 0.8491)	0.8804 (0.8698 - 0.8905)	0.8203 (0.8097 - 0.8326)	0.8614 (0.8300 - 0.9029)	0.7792 (0.7403 - 0.8095)	0.7960 (0.7696 - 0.8138)	0.8490 (0.8235 - 0.8843)	0.8274 (0.8135 - 0.8422)
Acute transmural myocardial infarction of inferior wall	0.8990 (0.8885 - 0.9087)	0.8398 (0.8283 - 0.8524)	0.8797 (0.8414 - 0.9085)	0.7999 (0.7781 - 0.8277)	0.8147 (0.7970 - 0.8362)	0.8693 (0.8395 - 0.8979)	0.8459 (0.8308 - 0.8603)	0.8989 (0.8909 - 0.9083)	0.8325 (0.8230 - 0.8452)	0.9000 (0.8475 - 0.9202)	0.7650 (0.7606 - 0.8173)	0.7930 (0.7820 - 0.8276)	0.8844 (0.8413 - 0.9068)	0.8431 (0.8287 - 0.8553)	0.8844 (0.8743 - 0.8942)	0.8220 (0.8111 - 0.8353)	0.8527 (0.8358 - 0.9136)	0.7914 (0.7284 - 0.7956)	0.8034 (0.7655 - 0.8149)	0.8431 (0.8264 - 0.8970)	0.8273 (0.8166 - 0.8437)
Left ventricular failure	0.8866 (0.8730 - 0.8991)	0.8186 (0.8059 - 0.8333)	0.8256 (0.7894 - 0.8571)	0.8115 (0.7867 - 0.8255)	0.8141 (0.7951 - 0.8315)	0.8231 (0.7940 - 0.8527)	0.8198 (0.8025 - 0.8370)	0.8928 (0.8813 - 0.9044)	0.8247 (0.8123 - 0.8412)	0.8376 (0.8048 - 0.8763)	0.8118 (0.7713 - 0.8383)	0.8166 (0.7881 - 0.8380)	0.8333 (0.8061 - 0.8665)	0.8269 (0.8104 - 0.8455)	0.8680 (0.8535 - 0.8803)	0.8062 (0.7920 - 0.8202)	0.8361 (0.7876 - 0.8663)	0.7764 (0.7647 - 0.8278)	0.7890 (0.7731 - 0.8199)	0.8257 (0.7900 - 0.8564)	0.8118 (0.7925 - 0.8286)
Pulmonary embolism without mention of acute cor pulmonale	0.6729 (0.6516 - 0.6930)	0.6365 (0.6196 - 0.6546)	0.5952 (0.5120 - 0.6501)	0.6778 (0.6450 - 0.7546)	0.6488 (0.6241 - 0.6853)	0.6261 (0.5978 - 0.6568)	0.6208 (0.5780 - 0.6524)	0.7144 (0.6946 - 0.7325)	0.6667 (0.6535 - 0.6857)	0.7541 (0.6233 - 0.7806)	0.5794 (0.5757 - 0.7018)	0.6419 (0.6260 - 0.6868)	0.7021 (0.6487 - 0.7283)	0.6935 (0.6469 - 0.7104)	0.6824 (0.6630 - 0.7016)	0.6440 (0.6276 - 0.6632)	0.6912 (0.5973 - 0.7319)	0.5968 (0.5778 - 0.6744)	0.6315 (0.6105 - 0.6664)	0.6590 (0.6212 - 0.6902)	0.6600 (0.6224 - 0.6851)
Supraventricular tachycardia	0.6290 (0.6063 - 0.6529)	0.6034 (0.5888 - 0.6276)	0.4686 (0.4414 - 0.7331)	0.7381 (0.4695 - 0.7534)	0.6415 (0.5731 - 0.6666)	0.5814 (0.5630 - 0.6547)	0.5416 (0.5209 - 0.6570)	0.6490 (0.6259 - 0.6700)	0.6205 (0.6041 - 0.6411)	0.6007 (0.5496 - 0.6875)	0.6404 (0.5649 - 0.6866)	0.6255 (0.5945 - 0.6526)	0.6159 (0.5894 - 0.6547)	0.6128 (0.5841 - 0.6530)	0.6112 (0.5880 - 0.6337)	0.5835 (0.5698 - 0.6063)	0.6023 (0.3779 - 0.7487)	0.5648 (0.4265 - 0.7940)	0.5805 (0.5502 - 0.6357)	0.5868 (0.5547 - 0.6411)	0.5912 (0.4788 - 0.6501)
Peripheral vascular disease, unspecified	0.8833 (0.8678 - 0.8979)	0.8219 (0.8091 - 0.8371)	0.8482 (0.8032 - 0.8981)	0.7956 (0.7515 - 0.8378)	0.8058 (0.7731 - 0.8342)	0.8398 (0.8057 - 0.8820)	0.8264 (0.8094 - 0.8441)	0.8854 (0.8719 - 0.8975)	0.8155 (0.8024 - 0.8301)	0.8482 (0.8049 - 0.8894)	0.7829 (0.7440 - 0.8263)	0.7962 (0.7713 - 0.8250)	0.8376 (0.8040 - 0.8760)	0.8214 (0.8055 - 0.8391)	0.8588 (0.8446 - 0.8715)	0.7962 (0.7839 - 0.8116)	0.8284 (0.7833 - 0.8593)	0.7639 (0.7336 - 0.7955)	0.7782 (0.7605 - 0.8044)	0.8166 (0.7818 - 0.8455)	0.8025 (0.7848 - 0.8198)
Acute transmural myocardial	0.8970 (0.8831 -	0.8375 (0.8265 -	0.8889 (0.8377 -	0.7862 (0.7652 -	0.8061 (0.7879 -	0.8762 (0.8354 -	0.8455 (0.8300 -	0.9033 (0.8929 -	0.8459 (0.8347 -	0.9045 (0.8772 -	0.7873 (0.7769 -	0.8096 (0.7952 -	0.8918 (0.8647 -	0.8544 (0.8400 -	0.8902 (0.8772 -	0.8295 (0.8156 -	0.8611 (0.8167 -	0.7978 (0.7533 -	0.8098 (0.7816 -	0.8517 (0.8184 -	0.8347 (0.8176 -

infarction of anterior wall	0.9086)	0.8513)	0.9201)	0.8303)	0.8383)	0.9074)	0.8606)	0.9133)	0.8593)	0.9304)	0.8001)	0.8268)	0.9207)	0.8695)	0.9018)	0.8448)	0.9044)	0.8371)	0.8371)	0.8898)	0.8522)
Transient cerebral ischaemic attack, unspecified	0.7673 (0.7447 - 0.7881)	0.7120 (0.6955 - 0.7338)	0.6896 (0.6041 - 0.7892)	0.7345 (0.6391 - 0.8126)	0.7220 (0.6796 - 0.7781)	0.7029 (0.6632 - 0.7568)	0.7054 (0.6712 - 0.7412)	0.7933 (0.7738 - 0.8122)	0.7381 (0.7231 - 0.7594)	0.7854 (0.6774 - 0.8165)	0.6908 (0.6862 - 0.7983)	0.7175 (0.7009 - 0.7780)	0.7630 (0.7088 - 0.7935)	0.7499 (0.7138 - 0.7713)	0.7580 (0.7324 - 0.7798)	0.7202 (0.7007 - 0.7414)	0.6667 (0.6183 - 0.7289)	0.7737 (0.7326 - 0.7894)	0.7465 (0.7144 - 0.7688)	0.6989 (0.6689 - 0.7385)	0.7043 (0.6738 - 0.7353)
Cerebral infarction, unspecified	0.8074 (0.7851 - 0.8285)	0.7543 (0.7360 - 0.7777)	0.7410 (0.6611 - 0.7918)	0.7676 (0.7460 - 0.8319)	0.7613 (0.7375 - 0.8071)	0.7477 (0.7078 - 0.7894)	0.7510 (0.7171 - 0.7807)	0.8279 (0.8087 - 0.8466)	0.7612 (0.7459 - 0.7817)	0.7635 (0.7061 - 0.8663)	0.7589 (0.6595 - 0.8332)	0.7600 (0.7096 - 0.8003)	0.7624 (0.7298 - 0.8343)	0.7618 (0.7407 - 0.7945)	0.8083 (0.7895 - 0.8267)	0.7449 (0.7244 - 0.7676)	0.7140 (0.6693 - 0.7803)	0.7758 (0.7164 - 0.7974)	0.7611 (0.7176 - 0.7848)	0.7306 (0.6981 - 0.7751)	0.7368 (0.7088 - 0.7667)
Mitral (valve) insufficiency	0.7524 (0.7280 - 0.7758)	0.6912 (0.6719 - 0.7180)	0.6314 (0.5515 - 0.7902)	0.7509 (0.5798 - 0.8231)	0.7171 (0.6471 - 0.7702)	0.6708 (0.6349 - 0.7441)	0.6715 (0.6314 - 0.7211)	0.7680 (0.7437 - 0.7920)	0.6992 (0.6826 - 0.7277)	0.7113 (0.5414 - 0.7872)	0.6871 (0.6186 - 0.8572)	0.6945 (0.6604 - 0.7953)	0.7042 (0.6401 - 0.7524)	0.7028 (0.6382 - 0.7366)	0.7249 (0.7002 - 0.7496)	0.6739 (0.6560 - 0.6991)	0.6649 (0.5696 - 0.7670)	0.6830 (0.5873 - 0.7651)	0.6771 (0.6392 - 0.7237)	0.6709 (0.6312 - 0.7245)	0.6710 (0.6279 - 0.7134)
Cardiomegaly	0.7530 (0.7267 - 0.7816)	0.6971 (0.6804 - 0.7240)	0.6556 (0.5287 - 0.8172)	0.7385 (0.5929 - 0.8661)	0.7149 (0.6521 - 0.8021)	0.6820 (0.6404 - 0.7638)	0.6840 (0.6340 - 0.7377)	0.8068 (0.7844 - 0.8266)	0.7392 (0.7173 - 0.7639)	0.7163 (0.6250 - 0.8220)	0.7622 (0.6588 - 0.8346)	0.7507 (0.6955 - 0.8062)	0.7287 (0.6824 - 0.7940)	0.7331 (0.6947 - 0.7672)	0.7678 (0.7431 - 0.7897)	0.7152 (0.6954 - 0.7401)	0.6942 (0.6361 - 0.7899)	0.7362 (0.6432 - 0.7796)	0.7247 (0.6795 - 0.7576)	0.7065 (0.6727 - 0.7607)	0.7091 (0.6784 - 0.7481)
Other forms of angina pectoris	0.8345 (0.8129 - 0.8564)	0.7745 (0.7558 - 0.7963)	0.7994 (0.7084 - 0.8560)	0.7497 (0.7010 - 0.8364)	0.7615 (0.7317 - 0.8155)	0.7889 (0.7386 - 0.8376)	0.7800 (0.7502 - 0.8072)	0.8619 (0.8441 - 0.8788)	0.8007 (0.7873 - 0.8220)	0.8634 (0.7857 - 0.9174)	0.7380 (0.7045 - 0.8239)	0.7672 (0.7398 - 0.8162)	0.8438 (0.7879 - 0.8983)	0.8124 (0.7894 - 0.8349)	0.8430 (0.8234 - 0.8611)	0.7862 (0.7677 - 0.8077)	0.8285 (0.7657 - 0.8784)	0.7439 (0.7075 - 0.8085)	0.7639 (0.7378 - 0.7999)	0.8126 (0.7710 - 0.8561)	0.7949 (0.7699 - 0.8187)
Acute subendocardial myocardial infarction	0.8629 (0.8437 - 0.8814)	0.8048 (0.7875 - 0.8267)	0.8421 (0.7762 - 0.8860)	0.7674 (0.7538 - 0.8203)	0.7836 (0.7614 - 0.8243)	0.8294 (0.7798 - 0.8714)	0.8118 (0.7879 - 0.8370)	0.8757 (0.8593 - 0.8906)	0.8070 (0.7908 - 0.8271)	0.9013 (0.7836 - 0.9355)	0.7127 (0.6914 - 0.8320)	0.7583 (0.7352 - 0.8267)	0.8784 (0.7946 - 0.9193)	0.8236 (0.7958 - 0.8441)	0.8557 (0.8361 - 0.8749)	0.7996 (0.7799 - 0.8213)	0.8520 (0.7763 - 0.8914)	0.7473 (0.7204 - 0.8090)	0.7713 (0.7472 - 0.8162)	0.8347 (0.7784 - 0.8763)	0.8096 (0.7810 - 0.8333)
Hypertensive renal disease	0.9100 (0.8841 -	0.8641 (0.8401 -	0.7805 (0.7376 -	0.9478 (0.9068 -	0.9373 (0.8959 -	0.8120 (0.7795 -	0.8517 (0.8221 -	0.9535 (0.9377 -	0.9051 (0.8875 -	0.8676 (0.8350 -	0.9427 (0.9184 -	0.9380 (0.9097 -	0.8768 (0.8467 -	0.9014 (0.8821 -	0.9424 (0.9238 -	0.8942 (0.8745 -	0.8502 (0.8103 -	0.9381 (0.8786 -	0.9322 (0.8806 -	0.8623 (0.8292 -	0.8893 (0.8669 -

with renal failure	0.9310)	0.8885)	0.8386)	0.9525)	0.9452)	0.8563)	0.8811)	0.9674)	0.9265)	0.9161)	0.9559)	0.9531)	0.9174)	0.9248)	0.9586)	0.9158)	0.9000)	0.9481)	0.9461)	0.9058)	0.9136)
Congestive heart failure	0.8936 (0.8692 - 0.9155)	0.8305 (0.8111 - 0.8589)	0.8357 (0.7573 - 0.8874)	0.8254 (0.7968 - 0.8966)	0.8271 (0.7971 - 0.8861)	0.8340 (0.7791 - 0.8801)	0.8314 (0.8032 - 0.8590)	0.9132 (0.8953 - 0.9291)	0.8433 (0.8243 - 0.8646)	0.8607 (0.8118 - 0.9101)	0.8258 (0.7589 - 0.8609)	0.8317 (0.8000 - 0.8601)	0.8557 (0.8139 - 0.8991)	0.8459 (0.8223 - 0.8701)	0.8934 (0.8765 - 0.9112)	0.8281 (0.8112 - 0.8479)	0.8821 (0.7946 - 0.9216)	0.7741 (0.7643 - 0.8684)	0.7961 (0.7738 - 0.8562)	0.8679 (0.8066 - 0.9112)	0.8369 (0.8141 - 0.8583)
Aortic (valve) stenosis	0.8233 (0.7915 - 0.8503)	0.7633 (0.7383 - 0.7942)	0.7436 (0.6650 - 0.8644)	0.7829 (0.6691 - 0.8435)	0.7740 (0.7039 - 0.8273)	0.7533 (0.7098 - 0.8350)	0.7585 (0.7259 - 0.8024)	0.8452 (0.8216 - 0.8681)	0.7664 (0.7494 - 0.7969)	0.8632 (0.7043 - 0.9143)	0.6695 (0.6450 - 0.8373)	0.7232 (0.6990 - 0.8189)	0.8304 (0.7335 - 0.8833)	0.7870 (0.7467 - 0.8122)	0.7902 (0.7596 - 0.8211)	0.7381 (0.7157 - 0.7684)	0.7991 (0.6597 - 0.8595)	0.6770 (0.6281 - 0.8096)	0.7121 (0.6795 - 0.7824)	0.7712 (0.6923 - 0.8292)	0.7531 (0.7049 - 0.7861)
Left bundle-branch block, unspecified	0.7114 (0.6716 - 0.7464)	0.6694 (0.6453 - 0.7050)	0.5867 (0.5159 - 0.8132)	0.7521 (0.5708 - 0.8135)	0.7030 (0.6164 - 0.7542)	0.6453 (0.6096 - 0.7601)	0.6396 (0.5989 - 0.7308)	0.7702 (0.7395 - 0.8003)	0.7189 (0.6984 - 0.7520)	0.7022 (0.6165 - 0.8174)	0.7355 (0.6247 - 0.8239)	0.7264 (0.6734 - 0.7846)	0.7118 (0.6669 - 0.7877)	0.7141 (0.6766 - 0.7618)	0.7445 (0.7111 - 0.7774)	0.7003 (0.6753 - 0.7339)	0.6444 (0.5635 - 0.7729)	0.7562 (0.6213 - 0.8181)	0.7255 (0.6700 - 0.7713)	0.6802 (0.6369 - 0.7429)	0.6826 (0.6351 - 0.7325)
Other and unspecified right bundle-branch block	0.6525 (0.6144 - 0.6884)	0.6264 (0.6000 - 0.6620)	0.6291 (0.4703 - 0.7559)	0.6237 (0.5186 - 0.7816)	0.6257 (0.5827 - 0.6893)	0.6271 (0.5783 - 0.6933)	0.6274 (0.5481 - 0.6852)	0.6990 (0.6641 - 0.7346)	0.6553 (0.6292 - 0.6898)	0.6995 (0.4855 - 0.7867)	0.6110 (0.5576 - 0.8279)	0.6426 (0.6002 - 0.7263)	0.6704 (0.6089 - 0.7380)	0.6699 (0.5848 - 0.7160)	0.6672 (0.6313 - 0.7015)	0.6356 (0.6096 - 0.6635)	0.7840 (0.4581 - 0.8334)	0.4871 (0.4850 - 0.8018)	0.6045 (0.5711 - 0.7046)	0.6928 (0.5871 - 0.7581)	0.6827 (0.5509 - 0.7134)
Ventricular tachycardia	0.7277 (0.6870 - 0.7697)	0.6913 (0.6647 - 0.7305)	0.6561 (0.4687 - 0.7419)	0.7265 (0.6595 - 0.8897)	0.7058 (0.6632 - 0.8322)	0.6787 (0.6115 - 0.7373)	0.6800 (0.5922 - 0.7254)	0.7875 (0.7505 - 0.8199)	0.7332 (0.7057 - 0.7688)	0.7196 (0.6373 - 0.8000)	0.7468 (0.7028 - 0.8018)	0.7397 (0.6976 - 0.7901)	0.7270 (0.6751 - 0.7925)	0.7295 (0.6869 - 0.7718)	0.7555 (0.7174 - 0.7932)	0.7130 (0.6838 - 0.7516)	0.6667 (0.5418 - 0.7513)	0.7593 (0.6980 - 0.8676)	0.7347 (0.6923 - 0.8173)	0.6949 (0.6388 - 0.7577)	0.6990 (0.6401 - 0.7466)
Cardiac arrhythmia, unspecified	0.6020 (0.5545 - 0.6423)	0.5872 (0.5646 - 0.6296)	0.3909 (0.3133 - 0.7455)	0.7836 (0.4508 - 0.8622)	0.6437 (0.5495 - 0.7015)	0.5626 (0.5369 - 0.6608)	0.4864 (0.4273 - 0.6608)	0.6561 (0.6158 - 0.6970)	0.6298 (0.6027 - 0.6657)	0.5482 (0.4270 - 0.7634)	0.7114 (0.5285 - 0.8164)	0.6551 (0.5851 - 0.7153)	0.6116 (0.5685 - 0.7036)	0.5969 (0.5289 - 0.6875)	0.6032 (0.5630 - 0.6442)	0.5904 (0.5620 - 0.6292)	0.5178 (0.3366 - 0.7292)	0.6630 (0.4468 - 0.8609)	0.6058 (0.5488 - 0.6892)	0.5789 (0.5390 - 0.6470)	0.5583 (0.4523 - 0.6435)
Occlusion and stenosis of carotid artery	0.8339 (0.8019 -	0.7760 (0.7497 -	0.7385 (0.6650 -	0.8135 (0.6968 -	0.7984 (0.7240 -	0.7567 (0.7083 -	0.7673 (0.7303 -	0.8659 (0.8397 -	0.8083 (0.7853 -	0.8256 (0.7540 -	0.7910 (0.7102 -	0.7980 (0.7568 -	0.8194 (0.7683 -	0.8116 (0.7797 -	0.8458 (0.8204 -	0.7890 (0.7663 -	0.8051 (0.7317 -	0.7728 (0.6853 -	0.7799 (0.7325 -	0.7986 (0.7482 -	0.7923 (0.7624 -

	0.8657)	0.8112)	0.8590)	0.8591)	0.8412)	0.8395)	0.8100)	0.8902)	0.8385)	0.8986)	0.8552)	0.8420)	0.8839)	0.8446)	0.8718)	0.8189)	0.8883)	0.8387)	0.8324)	0.8654)	0.8270)
Raynaud's syndrome	0.6711 (0.6315 - 0.7087)	0.6318 (0.6064 - 0.6729)	0.6429 (0.4103 - 0.7762)	0.6208 (0.5041 - 0.8574)	0.6290 (0.5936 - 0.7480)	0.6348 (0.5737 - 0.7012)	0.6359 (0.5240 - 0.6909)	0.7032 (0.6667 - 0.7412)	0.6578 (0.6337 - 0.6945)	0.6837 (0.5236 - 0.8277)	0.6319 (0.5078 - 0.7722)	0.6500 (0.6000 - 0.7202)	0.6664 (0.6126 - 0.7590)	0.6664 (0.5937 - 0.7243)	0.6428 (0.6024 - 0.6788)	0.6132 (0.5841 - 0.6482)	0.6480 (0.4336 - 0.8599)	0.5784 (0.3600 - 0.7825)	0.6058 (0.5515 - 0.6768)	0.6216 (0.5680 - 0.7313)	0.6262 (0.5252 - 0.6938)
Stricture of artery	0.8450 (0.8119 - 0.8755)	0.7846 (0.7591 - 0.8186)	0.7624 (0.7005 - 0.8548)	0.8067 (0.7408 - 0.8696)	0.7978 (0.7441 - 0.8432)	0.7725 (0.7299 - 0.8397)	0.7797 (0.7486 - 0.8217)	0.8836 (0.8634 - 0.9033)	0.8125 (0.7922 - 0.8429)	0.8122 (0.7680 - 0.9050)	0.8128 (0.7235 - 0.8450)	0.8126 (0.7581 - 0.8433)	0.8123 (0.7769 - 0.8896)	0.8124 (0.7873 - 0.8507)	0.8473 (0.8226 - 0.8698)	0.7771 (0.7535 - 0.8075)	0.8177 (0.7500 - 0.9390)	0.7365 (0.6312 - 0.7957)	0.7563 (0.6917 - 0.7960)	0.8016 (0.7522 - 0.9132)	0.7858 (0.7571 - 0.8232)
Sequelae of stroke, not specified as haemorrhage or infarction	0.7513 (0.7091 - 0.7912)	0.7023 (0.6650 - 0.7430)	0.5882 (0.5069 - 0.7421)	0.8164 (0.6319 - 0.8540)	0.7622 (0.6716 - 0.8033)	0.6647 (0.6158 - 0.7340)	0.6640 (0.6020 - 0.7271)	0.8229 (0.7941 - 0.8509)	0.7576 (0.7364 - 0.7929)	0.7582 (0.7295 - 0.9440)	0.7571 (0.5907 - 0.7911)	0.7574 (0.6664 - 0.7937)	0.7579 (0.7336 - 0.9187)	0.7578 (0.7402 - 0.8143)	0.7618 (0.7275 - 0.7973)	0.7233 (0.6950 - 0.7550)	0.8366 (0.7771 - 0.9211)	0.6099 (0.5417 - 0.6383)	0.6820 (0.6359 - 0.7189)	0.7887 (0.7331 - 0.8778)	0.7514 (0.7140 - 0.7899)
Subarachnoid haemorrhage, unspecified	0.5842 (0.5315 - 0.6343)	0.5874 (0.5576 - 0.6348)	0.5705 (0.3428 - 0.6579)	0.6042 (0.5824 - 0.8216)	0.5904 (0.5545 - 0.6876)	0.5845 (0.5286 - 0.6527)	0.5803 (0.4427 - 0.6381)	0.5119 (0.4653 - 0.5616)	0.5268 (0.4999 - 0.5728)	0.0738 (0.0519 - 0.8658)	0.9798 (0.2306 - 0.9866)	0.7848 (0.4951 - 0.8469)	0.5141 (0.4821 - 0.6277)	0.1350 (0.0970 - 0.6576)	0.5091 (0.4606 - 0.5543)	0.5237 (0.4951 - 0.5693)	0.3289 (0.0577 - 0.7875)	0.7186 (0.2769 - 0.9746)	0.5389 (0.4850 - 0.6950)	0.5171 (0.4794 - 0.6027)	0.4085 (0.1053 - 0.6416)
Atherosclerosis of arteries of the extremities	0.7849 (0.7426 - 0.8265)	0.7359 (0.7083 - 0.7761)	0.6600 (0.6131 - 0.8176)	0.8117 (0.6709 - 0.8304)	0.7780 (0.6915 - 0.8095)	0.7048 (0.6701 - 0.7975)	0.7142 (0.6799 - 0.7796)	0.8457 (0.8114 - 0.8794)	0.7964 (0.7714 - 0.8348)	0.7800 (0.6846 - 0.8704)	0.8127 (0.7605 - 0.8867)	0.8064 (0.7596 - 0.8716)	0.7870 (0.7261 - 0.8571)	0.7930 (0.7530 - 0.8381)	0.8191 (0.7857 - 0.8546)	0.7702 (0.7446 - 0.8088)	0.7533 (0.6842 - 0.9070)	0.7871 (0.6366 - 0.8418)	0.7796 (0.6937 - 0.8296)	0.7614 (0.7156 - 0.8801)	0.7663 (0.7319 - 0.8152)
Dilated cardiomyopathy	0.7900 (0.7464 - 0.8275)	0.7209 (0.6912 - 0.7653)	0.6345 (0.5113 - 0.8378)	0.8073 (0.6374 - 0.9275)	0.7670 (0.6643 - 0.8750)	0.6883 (0.6432 - 0.8079)	0.6945 (0.6347 - 0.7694)	0.8518 (0.8224 - 0.8784)	0.7830 (0.7596 - 0.8135)	0.8828 (0.7447 - 0.9452)	0.6831 (0.6242 - 0.8303)	0.7359 (0.6964 - 0.8125)	0.8535 (0.7602 - 0.9221)	0.8027 (0.7627 - 0.8339)	0.7891 (0.7531 - 0.8225)	0.7384 (0.7101 - 0.7767)	0.7241 (0.6478 - 0.8400)	0.7527 (0.6726 - 0.8235)	0.7455 (0.6870 - 0.7904)	0.7318 (0.6818 - 0.8174)	0.7346 (0.6956 - 0.7866)
Aortic (valve)	0.7008 (0.65	0.6623 (0.63	0.5203 (0.40	0.8044 (0.61	0.7267 (0.62	0.6264 (0.58	0.6064 (0.52	0.7867 (0.75	0.7273 (0.69	0.7162 (0.55	0.7383 (0.69	0.7324 (0.69	0.7224 (0.65	0.7242 (0.65	0.7318 (0.68	0.6857 (0.65	0.6554 (0.55	0.7160 (0.47	0.6977 (0.61	0.6751 (0.63	0.6759 (0.62

insufficiency	13 - 0.7519)	35 - 0.7138)	14 - 0.7080)	96 - 0.8909)	80 - 0.8228)	14 - 0.7051)	80 - 0.6926)	18 - 0.8231)	98 - 0.7696)	86 - 0.7901)	38 - 0.8760)	71 - 0.8338)	54 - 0.7846)	91 - 0.7699)	76 - 0.7723)	65 - 0.7262)	72 - 0.8657)	08 - 0.8454)	68 - 0.7568)	08 - 0.8011)	73 - 0.7460)
Heart failure, unspecified	0.7830 (0.7414 - 0.8255)	0.7243 (0.6951 - 0.7694)	0.6259 (0.5294 - 0.8074)	0.8228 (0.6439 - 0.9065)	0.7794 (0.6863 - 0.8606)	0.6874 (0.6381 - 0.7935)	0.6942 (0.6424 - 0.7678)	0.8566 (0.8246 - 0.8881)	0.7815 (0.7531 - 0.8242)	0.7482 (0.6601 - 0.8786)	0.8149 (0.7217 - 0.8855)	0.8017 (0.7224 - 0.8663)	0.7639 (0.7143 - 0.8636)	0.7740 (0.7316 - 0.8243)	0.8202 (0.7869 - 0.8518)	0.7504 (0.7216 - 0.7896)	0.7770 (0.6522 - 0.8857)	0.7238 (0.6022 - 0.8331)	0.7378 (0.6780 - 0.8149)	0.7645 (0.6939 - 0.8516)	0.7569 (0.7050 - 0.8002)
Cerebrovascular disease, unspecified	0.7036 (0.6479 - 0.7593)	0.6736 (0.6367 - 0.7211)	0.5726 (0.4049 - 0.7913)	0.7745 (0.5541 - 0.9309)	0.7175 (0.6338 - 0.8448)	0.6444 (0.5828 - 0.7328)	0.6369 (0.5404 - 0.7205)	0.8013 (0.7551 - 0.8391)	0.7466 (0.7116 - 0.7922)	0.7094 (0.5937 - 0.8253)	0.7837 (0.6907 - 0.8762)	0.7664 (0.6995 - 0.8391)	0.7295 (0.6681 - 0.8186)	0.7368 (0.6795 - 0.7935)	0.7584 (0.7099 - 0.8051)	0.7213 (0.6817 - 0.7703)	0.5983 (0.5284 - 0.8077)	0.8443 (0.6329 - 0.8555)	0.7935 (0.6635 - 0.8268)	0.6776 (0.6292 - 0.7831)	0.6822 (0.6259 - 0.7567)
Mitral (valve) prolapse	0.6292 (0.5716 - 0.6813)	0.6176 (0.5836 - 0.6666)	0.3985 (0.3138 - 0.6793)	0.8368 (0.5257 - 0.8909)	0.7094 (0.5821 - 0.7769)	0.5818 (0.5417 - 0.6694)	0.5103 (0.4398 - 0.6459)	0.6932 (0.6432 - 0.7401)	0.6592 (0.6284 - 0.7041)	0.5789 (0.4532 - 0.7853)	0.7396 (0.5991 - 0.8449)	0.6897 (0.6129 - 0.7571)	0.6372 (0.5907 - 0.7412)	0.6295 (0.5604 - 0.7223)	0.6627 (0.6172 - 0.7036)	0.6337 (0.6030 - 0.6739)	0.6541 (0.4318 - 0.7814)	0.6133 (0.4932 - 0.8241)	0.6285 (0.5808 - 0.7191)	0.6394 (0.5727 - 0.7165)	0.6410 (0.5270 - 0.6985)
Subsequent myocardial infarction of unspecified site	0.8446 (0.8102 - 0.8811)	0.7986 (0.7720 - 0.8373)	0.7561 (0.7120 - 0.9182)	0.8411 (0.7043 - 0.8726)	0.8264 (0.7233 - 0.8586)	0.7752 (0.7373 - 0.8980)	0.7897 (0.7593 - 0.8430)	0.8942 (0.8667 - 0.9175)	0.8404 (0.8135 - 0.8688)	0.8943 (0.8261 - 0.9436)	0.7864 (0.7708 - 0.8308)	0.8072 (0.7758 - 0.8455)	0.8815 (0.8237 - 0.9326)	0.8485 (0.8153 - 0.8798)	0.8585 (0.8241 - 0.8896)	0.8017 (0.7747 - 0.8403)	0.7886 (0.7292 - 0.9097)	0.8149 (0.7224 - 0.8456)	0.8099 (0.7401 - 0.8443)	0.7940 (0.7460 - 0.8917)	0.7991 (0.7666 - 0.8458)
Disease of pericardium, unspecified	0.6170 (0.5633 - 0.6744)	0.6145 (0.5768 - 0.6676)	0.4954 (0.4375 - 0.7228)	0.7336 (0.5492 - 0.7381)	0.6503 (0.5640 - 0.7008)	0.5925 (0.5443 - 0.6897)	0.5624 (0.5127 - 0.6719)	0.6671 (0.6186 - 0.7153)	0.6385 (0.6017 - 0.6829)	0.7523 (0.5041 - 0.8801)	0.5246 (0.3356 - 0.7359)	0.6128 (0.5606 - 0.7043)	0.6793 (0.5847 - 0.7927)	0.6754 (0.5709 - 0.7266)	0.6199 (0.5665 - 0.6688)	0.5988 (0.5679 - 0.6508)	0.4128 (0.3393 - 0.8109)	0.7849 (0.4253 - 0.8278)	0.6574 (0.5461 - 0.7194)	0.5720 (0.5349 - 0.7162)	0.5072 (0.4471 - 0.6853)
Embolism and thrombosis of arteries of the lower extremities	0.7628 (0.7140 - 0.8134)	0.7021 (0.6734 - 0.7578)	0.6545 (0.4959 - 0.8559)	0.7496 (0.5697 - 0.8924)	0.7233 (0.6312 - 0.8445)	0.6845 (0.6246 - 0.8102)	0.6872 (0.6169 - 0.7635)	0.8446 (0.8097 - 0.8758)	0.7784 (0.7462 - 0.8194)	0.7455 (0.6818 - 0.8922)	0.8114 (0.7040 - 0.8524)	0.7981 (0.7164 - 0.8357)	0.7612 (0.7157 - 0.8675)	0.7709 (0.7315 - 0.8255)	0.7982 (0.7549 - 0.8364)	0.7612 (0.7299 - 0.7973)	0.8364 (0.7022 - 0.9135)	0.6860 (0.6424 - 0.8133)	0.7271 (0.6823 - 0.7945)	0.8074 (0.7232 - 0.8896)	0.7779 (0.7262 - 0.8196)

Other forms of acute ischaemic heart disease	0.7878 (0.7316 - 0.8392)	0.7449 (0.7054 - 0.7985)	0.6000 (0.5333 - 0.8370)	0.8897 (0.6794 - 0.9064)	0.8447 (0.6880 - 0.8771)	0.6899 (0.6449 - 0.8117)	0.7016 (0.6515 - 0.7864)	0.8857 (0.8524 - 0.9165)	0.8243 (0.7933 - 0.8609)	0.8211 (0.7565 - 0.9425)	0.8276 (0.7106 - 0.8599)	0.8265 (0.7418 - 0.8617)	0.8222 (0.7677 - 0.9311)	0.8238 (0.7876 - 0.8667)	0.8450 (0.8004 - 0.8880)	0.8028 (0.7736 - 0.8447)	0.8632 (0.7292 - 0.9250)	0.7424 (0.7308 - 0.8588)	0.7702 (0.7313 - 0.8486)	0.8444 (0.7515 - 0.9099)	0.8140 (0.7673 - 0.8565)
Other specified cardiac arrhythmias	0.6197 (0.5622 - 0.6822)	0.6147 (0.5758 - 0.6732)	0.5758 (0.4556 - 0.7159)	0.6536 (0.5096 - 0.7242)	0.6244 (0.5646 - 0.6957)	0.6064 (0.5458 - 0.6927)	0.5991 (0.5194 - 0.6770)	0.6942 (0.6366 - 0.7465)	0.6702 (0.6317 - 0.7192)	0.6667 (0.4951 - 0.8021)	0.6737 (0.5531 - 0.8335)	0.6714 (0.6086 - 0.7687)	0.6690 (0.5986 - 0.7606)	0.6690 (0.5817 - 0.7318)	0.6746 (0.6176 - 0.7278)	0.6435 (0.6046 - 0.6966)	0.5657 (0.4285 - 0.8061)	0.7213 (0.4922 - 0.8540)	0.6700 (0.5815 - 0.7604)	0.6242 (0.5717 - 0.7445)	0.6134 (0.5274 - 0.7168)
Naevus, nonneoplastic	0.5004 (0.4328 - 0.5686)	0.5318 (0.4893 - 0.5990)	0.9254 (0.3173 - 1.0000)	0.1383 (0.0719 - 0.7671)	0.5178 (0.4636 - 0.6292)	0.6495 (0.5032 - 1.0000)	0.6640 (0.4050 - 0.7202)	0.5411 (0.4747 - 0.6108)	0.5516 (0.5107 - 0.6268)	0.3433 (0.2409 - 1.0000)	0.7599 (0.0684 - 0.8523)	0.5884 (0.4863 - 0.7040)	0.5364 (0.4932 - 1.0000)	0.4336 (0.3487 - 0.7047)	0.5490 (0.4817 - 0.6157)	0.5608 (0.5152 - 0.6266)	0.6119 (0.2386 - 0.9032)	0.5096 (0.2503 - 0.8649)	0.5552 (0.4841 - 0.6662)	0.5677 (0.5078 - 0.7424)	0.5822 (0.3432 - 0.7011)
Atrioventricular block, complete	0.7500 (0.7043 - 0.7932)	0.7037 (0.6765 - 0.7495)	0.8144 (0.6320 - 0.9174)	0.5929 (0.5126 - 0.7544)	0.6668 (0.6178 - 0.7576)	0.7616 (0.6636 - 0.8710)	0.7332 (0.6728 - 0.7845)	0.8024 (0.7554 - 0.8456)	0.7455 (0.7089 - 0.7948)	0.7113 (0.5882 - 0.8819)	0.7797 (0.6069 - 0.8794)	0.7636 (0.6692 - 0.8392)	0.7298 (0.6611 - 0.8495)	0.7365 (0.6763 - 0.7959)	0.7743 (0.7254 - 0.8182)	0.7500 (0.7101 - 0.7945)	0.7629 (0.6667 - 0.8511)	0.7371 (0.6730 - 0.7707)	0.7437 (0.6899 - 0.7936)	0.7566 (0.6853 - 0.8410)	0.7532 (0.6937 - 0.8038)
Acute transmural myocardial infarction of other sites	0.8287 (0.7854 - 0.8703)	0.7897 (0.7537 - 0.8271)	0.8041 (0.7315 - 0.9101)	0.7752 (0.6979 - 0.8169)	0.7815 (0.7169 - 0.8178)	0.7983 (0.7373 - 0.8944)	0.7927 (0.7462 - 0.8375)	0.9073 (0.8836 - 0.9274)	0.8436 (0.8134 - 0.8792)	0.8454 (0.7857 - 0.9451)	0.8418 (0.7339 - 0.8798)	0.8424 (0.7698 - 0.8803)	0.8448 (0.7956 - 0.9388)	0.8439 (0.8130 - 0.8845)	0.8835 (0.8507 - 0.9118)	0.8386 (0.8145 - 0.8719)	0.9278 (0.8191 - 0.9677)	0.7493 (0.7482 - 0.8441)	0.7873 (0.7603 - 0.8548)	0.9121 (0.8234 - 0.9604)	0.8518 (0.8131 - 0.8840)
Atrioventricular block, first degree	0.6483 (0.5821 - 0.7103)	0.6287 (0.5902 - 0.6878)	0.5567 (0.3077 - 0.7851)	0.7006 (0.4944 - 0.9206)	0.6503 (0.5741 - 0.8143)	0.6125 (0.5532 - 0.7133)	0.5999 (0.4448 - 0.6993)	0.7577 (0.7112 - 0.8023)	0.7072 (0.6759 - 0.7529)	0.8247 (0.6315 - 0.8980)	0.5896 (0.5207 - 0.7814)	0.6677 (0.6237 - 0.7603)	0.7709 (0.6640 - 0.8552)	0.7380 (0.6714 - 0.7833)	0.6792 (0.6204 - 0.7361)	0.6551 (0.6151 - 0.7067)	0.6289 (0.4118 - 0.7471)	0.6814 (0.5768 - 0.8505)	0.6637 (0.6036 - 0.7781)	0.6474 (0.5694 - 0.7335)	0.6458 (0.5268 - 0.7110)
Other ill-defined heart diseases	0.7375 (0.6860 - 0.7894)	0.6971 (0.6601 - 0.7552)	0.5446 (0.4737 - 0.7583)	0.8496 (0.6728 - 0.8758)	0.7836 (0.6701 - 0.8302)	0.6510 (0.6033 - 0.7555)	0.6426 (0.5857 - 0.7404)	0.8253 (0.7784 - 0.8695)	0.7711 (0.7361 - 0.8201)	0.6832 (0.6199 - 0.8495)	0.8590 (0.7232 - 0.8900)	0.8289 (0.7222 - 0.8662)	0.7306 (0.6843 - 0.8330)	0.7490 (0.7052 - 0.8188)	0.7854 (0.7323 - 0.8331)	0.7561 (0.7098 - 0.8059)	0.6238 (0.5368 - 0.7355)	0.8884 (0.8723 - 0.8903)	0.8483 (0.7902 - 0.8738)	0.7025 (0.6423 - 0.7823)	0.7189 (0.6497 - 0.7844)

Cardiomyopathy, unspecified	0.6902 (0.6253 - 0.7506)	0.6649 (0.6263 - 0.7216)	0.5978 (0.4022 - 0.7648)	0.7319 (0.6046 - 0.8854)	0.6904 (0.6257 - 0.8205)	0.6454 (0.5736 - 0.7456)	0.6408 (0.5282 - 0.7177)	0.8087 (0.7698 - 0.8518)	0.7590 (0.7274 - 0.7991)	0.8370 (0.6981 - 0.9231)	0.6811 (0.5992 - 0.8216)	0.7241 (0.6702 - 0.8028)	0.8069 (0.7224 - 0.8995)	0.7764 (0.7258 - 0.8200)	0.7650 (0.7154 - 0.8076)	0.7316 (0.6912 - 0.7690)	0.8261 (0.6905 - 0.8967)	0.6371 (0.6352 - 0.7652)	0.6948 (0.6410 - 0.7596)	0.7856 (0.6956 - 0.8671)	0.7548 (0.6904 - 0.7974)
Abdominal aortic aneurysm, without mention of rupture	0.8645 (0.8259 - 0.9052)	0.8030 (0.7648 - 0.8544)	0.7386 (0.6666 - 0.9000)	0.8673 (0.6844 - 0.8907)	0.8477 (0.7271 - 0.8796)	0.7684 (0.7148 - 0.8827)	0.7894 (0.7413 - 0.8512)	0.8921 (0.8512 - 0.9234)	0.8283 (0.7962 - 0.8639)	0.8864 (0.7700 - 0.9457)	0.7702 (0.7517 - 0.8818)	0.7941 (0.7578 - 0.8648)	0.8714 (0.7854 - 0.9336)	0.8377 (0.7964 - 0.8752)	0.8368 (0.7872 - 0.8814)	0.7850 (0.7492 - 0.8366)	0.8182 (0.6249 - 0.8830)	0.7519 (0.7508 - 0.9042)	0.7673 (0.7309 - 0.8902)	0.8053 (0.6994 - 0.8714)	0.7919 (0.7207 - 0.8367)
Intracerebral haemorrhage, unspecified	0.6855 (0.6253 - 0.7402)	0.6482 (0.6169 - 0.7064)	0.6512 (0.4390 - 0.8646)	0.6452 (0.4954 - 0.8323)	0.6473 (0.5792 - 0.7661)	0.6491 (0.5806 - 0.7920)	0.6492 (0.5450 - 0.7339)	0.6427 (0.5901 - 0.6963)	0.6122 (0.5824 - 0.6699)	0.8140 (0.5319 - 0.9579)	0.4105 (0.2240 - 0.7086)	0.5800 (0.5296 - 0.6840)	0.6881 (0.5711 - 0.8642)	0.6773 (0.5696 - 0.7311)	0.5645 (0.5127 - 0.6154)	0.5822 (0.5454 - 0.6367)	0.8140 (0.4698 - 0.8934)	0.3505 (0.2838 - 0.7067)	0.5562 (0.5054 - 0.6359)	0.6533 (0.5457 - 0.7700)	0.6608 (0.5310 - 0.7128)

Table S7. internal geographic hold-out validation of XGBoost model for CVD class-level Discrimination

a. Based on metabolomic data

Disease	AUC (95%CI)	Acc (95%CI)	Sn (95%CI)	Sp (95%CI)	PPV (95%CI)	NPV (95%CI)	F1 score (95%CI)
Essential (primary) hypertension	0.7694 (0.7552 - 0.7840)	0.7057 (0.6950 - 0.7210)	0.7013 (0.6167 - 0.7680)	0.7100 (0.6398 - 0.7890)	0.7075 (0.6789 - 0.7538)	0.7039 (0.6708 - 0.7438)	0.7044 (0.6733 - 0.7303)
Chronic ischaemic heart disease	0.8842 (0.8723 - 0.8950)	0.8110 (0.7991 - 0.8256)	0.7994 (0.7740 - 0.8851)	0.8226 (0.7464 - 0.8379)	0.8184 (0.7670 - 0.8357)	0.8039 (0.7833 - 0.8707)	0.8088 (0.7954 - 0.8328)
Angina pectoris	0.8452 (0.8288 - 0.8612)	0.7754 (0.7594 - 0.7937)	0.8012 (0.7305 - 0.8549)	0.7497 (0.7010 - 0.8178)	0.7619 (0.7347 - 0.8063)	0.7904 (0.7477 - 0.8336)	0.7811 (0.7573 - 0.8046)
Atrial fibrillation and flutter	0.7269 (0.6936 - 0.7608)	0.6719 (0.6482 - 0.7066)	0.5514 (0.4596 - 0.7511)	0.7924 (0.6222 - 0.8796)	0.7265 (0.6360 - 0.7986)	0.6385 (0.6045 - 0.7212)	0.6270 (0.5755 - 0.7110)
Acute myocardial infarction	0.8759 (0.8548 - 0.8948)	0.8111 (0.7895 - 0.8385)	0.7847 (0.7489 - 0.8772)	0.8376 (0.7639 - 0.8476)	0.8285 (0.7769 - 0.8467)	0.7955 (0.7674 - 0.8691)	0.8060 (0.7814 - 0.8426)
Heart failure	0.8343 (0.7938 - 0.8696)	0.7919 (0.7622 - 0.8306)	0.7752 (0.7050 - 0.8730)	0.8085 (0.7352 - 0.8492)	0.8019 (0.7419 - 0.8469)	0.7824 (0.7327 - 0.8637)	0.7883 (0.7495 - 0.8343)
Paroxysmal tachycardia	0.5766 (0.5131 - 0.6370)	0.5760 (0.5438 - 0.6387)	0.7065 (0.2688 - 0.7975)	0.4456 (0.4413 - 0.8482)	0.5603 (0.5191 - 0.7287)	0.6029 (0.5144 - 0.6958)	0.6250 (0.3776 - 0.6822)
Complications and ill-defined descriptions of heart disease	0.6960 (0.6268 - 0.7588)	0.6587 (0.6135 - 0.7221)	0.7647 (0.4444 - 0.8718)	0.5526 (0.5047 - 0.8805)	0.6309 (0.5692 - 0.7784)	0.7014 (0.5900 - 0.8160)	0.6914 (0.5490 - 0.7559)
Other cardiac arrhythmias	0.5849 (0.5221 - 0.6535)	0.5767 (0.5468 - 0.6451)	0.9118 (0.3506 - 0.9737)	0.2417 (0.1619 - 0.8273)	0.5460 (0.5046 - 0.6786)	0.7326 (0.5350 - 0.8998)	0.6830 (0.4595 - 0.7225)
Cerebral infarction	0.7748 (0.7108 - 0.8347)	0.7394 (0.6931 - 0.8006)	0.7015 (0.5535 - 0.8261)	0.7772 (0.6849 - 0.8746)	0.7590 (0.6861 - 0.8425)	0.7225 (0.6436 - 0.8290)	0.7291 (0.6441 - 0.7995)
Other peripheral vascular diseases	0.7444 (0.6801 - 0.8048)	0.7105 (0.6602 - 0.7665)	0.6970 (0.5263 - 0.8136)	0.7240 (0.6737 - 0.8437)	0.7163 (0.6436 - 0.8036)	0.7049 (0.6146 - 0.7999)	0.7065 (0.6152 - 0.7741)
Pulmonary embolism	0.6464 (0.5897 - 0.6954)	0.6409 (0.5952 - 0.6912)	0.8571 (0.6154 - 0.9683)	0.4247 (0.3083 - 0.6923)	0.5984 (0.5319 - 0.6682)	0.7483 (0.6274 - 0.9217)	0.7048 (0.6147 - 0.7613)
Transient cerebral ischaemic attacks and related syndromes	0.6776 (0.6024 - 0.7548)	0.6809 (0.6231 - 0.7469)	0.5833 (0.4261 - 0.7193)	0.7785 (0.6811 - 0.9067)	0.7247 (0.6487 - 0.8248)	0.6514 (0.5761 - 0.7465)	0.6464 (0.5370 - 0.7304)
Atrioventricular and left bundle-branch block	0.6988 (0.6161 - 0.7750)	0.6802 (0.6277 - 0.7564)	0.5962 (0.4510 - 0.8298)	0.7642 (0.5295 - 0.8781)	0.7166 (0.6026 - 0.8159)	0.6543 (0.5839 - 0.7976)	0.6508 (0.5596 - 0.7560)
Nonrheumatic aortic valve disorders	0.7577 (0.6985 - 0.8113)	0.7019 (0.6723 - 0.7656)	0.9400 (0.5806 - 1.0000)	0.4637 (0.4352 - 0.8484)	0.6367 (0.5742 - 0.8153)	0.8854 (0.6487 - 1.0000)	0.7592 (0.6624 - 0.8062)

b. Based on metabolome combined with clinical data

Disease	AUC (95%CI)	Acc (95%CI)	Sn (95%CI)	Sp (95%CI)	PPV (95%CI)	NPV (95%CI)	F1 score (95%CI)
Essential (primary) hypertension	0.8157 (0.8034 - 0.8273)	0.7459 (0.7334 - 0.7585)	0.7536 (0.7139 - 0.8154)	0.7382 (0.6722 - 0.7699)	0.7422 (0.7114 - 0.7587)	0.7498 (0.7273 - 0.7855)	0.7479 (0.7305 - 0.7680)
Chronic ischaemic heart disease	0.8992 (0.8889 - 0.9094)	0.8309 (0.8206 - 0.8453)	0.8663 (0.8296 - 0.9146)	0.7956 (0.7472 - 0.8349)	0.8091 (0.7810 - 0.8342)	0.8561 (0.8289 - 0.9009)	0.8367 (0.8236 - 0.8538)
Angina pectoris	0.8718 (0.8582 - 0.8849)	0.8009 (0.7878 - 0.8190)	0.8316 (0.7715 - 0.8779)	0.7702 (0.7347 - 0.8346)	0.7835 (0.7601 - 0.8227)	0.8206 (0.7837 - 0.8628)	0.8069 (0.7875 - 0.8285)
Atrial fibrillation and flutter	0.8046 (0.7770 - 0.8318)	0.7320 (0.7109 - 0.7612)	0.8272 (0.6316 - 0.8948)	0.6368 (0.5878 - 0.8361)	0.6949 (0.6622 - 0.8001)	0.7865 (0.6873 - 0.8562)	0.7553 (0.6976 - 0.7885)
Acute myocardial infarction	0.8920 (0.8710 - 0.9091)	0.8189 (0.7991 - 0.8464)	0.8182 (0.7684 - 0.9415)	0.8196 (0.6958 - 0.8496)	0.8194 (0.7442 - 0.8513)	0.8184 (0.7807 - 0.9240)	0.8188 (0.7967 - 0.8513)
Heart failure	0.8515 (0.8148 - 0.8843)	0.8019 (0.7716 - 0.8408)	0.7984 (0.7368 - 0.8953)	0.8053 (0.7492 - 0.8354)	0.8040 (0.7528 - 0.8379)	0.7998 (0.7532 - 0.8854)	0.8012 (0.7633 - 0.8485)
Paroxysmal tachycardia	0.6082 (0.5527 - 0.6608)	0.5934 (0.5587 - 0.6472)	0.6304 (0.2600 - 0.8902)	0.5564 (0.2599 - 0.9041)	0.5870 (0.5205 - 0.7447)	0.6009 (0.5381 - 0.7493)	0.6079 (0.3893 - 0.6964)
Complications and ill-defined descriptions of heart disease	0.7145 (0.6454 - 0.7832)	0.6597 (0.6238 - 0.7329)	0.5294 (0.3971 - 0.9296)	0.7900 (0.3820 - 0.8811)	0.7160 (0.5721 - 0.8264)	0.6267 (0.5806 - 0.8655)	0.6087 (0.5210 - 0.7548)
Other cardiac arrhythmias	0.6199 (0.5536 - 0.6836)	0.5960 (0.5583 - 0.6640)	0.4853 (0.3696 - 0.9571)	0.7067 (0.2640 - 0.8335)	0.6233 (0.5151 - 0.7203)	0.5786 (0.5386 - 0.8599)	0.5457 (0.4739 - 0.7293)
Cerebral infarction	0.8175 (0.7635 - 0.8699)	0.7744 (0.7270 - 0.8298)	0.7612 (0.6140 - 0.8611)	0.7876 (0.7412 - 0.9025)	0.7818 (0.7339 - 0.8729)	0.7673 (0.6752 - 0.8679)	0.7714 (0.6950 - 0.8318)
Other peripheral vascular diseases	0.7585 (0.6934 - 0.8224)	0.7115 (0.6664 - 0.7705)	0.7727 (0.5455 - 0.8750)	0.6502 (0.5886 - 0.8771)	0.6884 (0.6329 - 0.8186)	0.7410 (0.6323 - 0.8479)	0.7281 (0.6239 - 0.7877)
Pulmonary embolism	0.6553 (0.5996 - 0.7034)	0.6535 (0.6119 - 0.7035)	0.8730 (0.6973 - 1.0000)	0.4340 (0.3011 - 0.6278)	0.6067 (0.5380 - 0.6709)	0.7736 (0.6708 - 1.0000)	0.7159 (0.6583 - 0.7731)
Transient cerebral ischaemic attacks and related syndromes	0.7049 (0.6302 - 0.7744)	0.6893 (0.6404 - 0.7564)	0.7000 (0.4443 - 0.8071)	0.6786 (0.6743 - 0.8800)	0.6854 (0.6242 - 0.8354)	0.6935 (0.5924 - 0.7898)	0.6926 (0.5638 - 0.7673)
Atrioventricular and left bundle-branch block	0.7211 (0.6363 - 0.7935)	0.7070 (0.6563 - 0.7822)	0.6154 (0.4680 - 0.7963)	0.7987 (0.6765 - 0.8622)	0.7535 (0.6513 - 0.8325)	0.6750 (0.5984 - 0.8016)	0.6775 (0.5770 - 0.7795)
Nonrheumatic aortic valve disorders	0.8020 (0.7477 - 0.8520)	0.7309 (0.6911 - 0.7973)	0.7200 (0.6031 - 0.9623)	0.7419 (0.5418 - 0.8387)	0.7361 (0.6181 - 0.8192)	0.7260 (0.6586 - 0.9334)	0.7280 (0.6741 - 0.8159)

Table S8. internal geographic hold-out validation of XGBoost model for CVD subclass-level

a. Based on metabolomic data

Disease	AUC (95%CI)	Acc (95%CI)	Sn (95%CI)	Sp (95%CI)	PPV (95%CI)	NPV (95%CI)	F1 score (95%CI)
Atherosclerotic heart disease	0.8960 (0.8844 - 0.9059)	0.8189 (0.8075 - 0.8362)	0.8250 (0.8016 - 0.9172)	0.8128 (0.7227 - 0.8391)	0.8150 (0.7622 - 0.8335)	0.8228 (0.8042 - 0.9007)	0.8200 (0.8096 - 0.8468)
Angina pectoris, unspecified	0.8463 (0.8271 - 0.8638)	0.7774 (0.7610 - 0.7995)	0.7678 (0.7123 - 0.8685)	0.7870 (0.7030 - 0.8388)	0.7828 (0.7274 - 0.8210)	0.7722 (0.7393 - 0.8473)	0.7752 (0.7549 - 0.8082)
Chronic ischaemic heart disease, unspecified	0.8554 (0.8317 - 0.8749)	0.7821 (0.7620 - 0.8100)	0.8205 (0.7130 - 0.8805)	0.7438 (0.6960 - 0.8442)	0.7620 (0.7313 - 0.8333)	0.8056 (0.7365 - 0.8579)	0.7902 (0.7556 - 0.8191)
Old myocardial infarction	0.8562 (0.8317 - 0.8811)	0.7916 (0.7695 - 0.8239)	0.7966 (0.7312 - 0.9035)	0.7865 (0.6961 - 0.8474)	0.7887 (0.7204 - 0.8353)	0.7945 (0.7502 - 0.8855)	0.7926 (0.7651 - 0.8340)
Unstable angina	0.8188 (0.7882 - 0.8499)	0.7545 (0.7246 - 0.7888)	0.7561 (0.6503 - 0.8217)	0.7530 (0.7282 - 0.8299)	0.7538 (0.7199 - 0.8152)	0.7553 (0.6938 - 0.8149)	0.7549 (0.7059 - 0.7963)
Left ventricular failure	0.8471 (0.8103 - 0.8812)	0.7862 (0.7582 - 0.8292)	0.7429 (0.6909 - 0.9252)	0.8295 (0.6243 - 0.8615)	0.8133 (0.7058 - 0.8508)	0.7634 (0.7233 - 0.9020)	0.7765 (0.7459 - 0.8367)
Acute transmural myocardial infarction of inferior wall	0.8470 (0.8089 - 0.8865)	0.7782 (0.7368 - 0.8315)	0.8000 (0.6621 - 0.9366)	0.7564 (0.6418 - 0.8717)	0.7666 (0.6828 - 0.8543)	0.7909 (0.7078 - 0.9135)	0.7829 (0.7218 - 0.8409)
Acute myocardial infarction, unspecified	0.8436 (0.8003 - 0.8803)	0.7810 (0.7450 - 0.8343)	0.7619 (0.6956 - 0.9741)	0.8001 (0.5683 - 0.8684)	0.7921 (0.6678 - 0.8487)	0.7707 (0.7191 - 0.9553)	0.7767 (0.7364 - 0.8429)
Supraventricular tachycardia	0.5735 (0.5058 - 0.6384)	0.5886 (0.5442 - 0.6476)	0.8254 (0.4374 - 0.9107)	0.3519 (0.3100 - 0.7129)	0.5601 (0.4954 - 0.6604)	0.6683 (0.5368 - 0.8166)	0.6674 (0.4913 - 0.7258)
Acute transmural myocardial infarction of anterior wall	0.9012 (0.8713 - 0.9261)	0.8494 (0.8184 - 0.8861)	0.9206 (0.8200 - 0.9710)	0.7782 (0.7741 - 0.8416)	0.8058 (0.7709 - 0.8636)	0.9074 (0.8221 - 0.9633)	0.8594 (0.8141 - 0.8972)
Pulmonary embolism without mention of acute cor pulmonale	0.6358 (0.5684 - 0.6970)	0.6300 (0.5824 - 0.6907)	0.6333 (0.5581 - 0.9181)	0.6267 (0.4010 - 0.6683)	0.6291 (0.5284 - 0.7001)	0.6309 (0.5675 - 0.8319)	0.6312 (0.5678 - 0.7397)
Cerebral infarction, unspecified	0.7442 (0.6693 - 0.8128)	0.7097 (0.6511 - 0.7782)	0.6250 (0.4815 - 0.8114)	0.7944 (0.6535 - 0.8915)	0.7525 (0.6542 - 0.8359)	0.6793 (0.5970 - 0.8077)	0.6828 (0.5925 - 0.7805)
Cardiomegaly	0.7066 (0.6383 - 0.7665)	0.6793 (0.6336 - 0.7400)	0.7222 (0.5640 - 0.9388)	0.6365 (0.4212 - 0.7868)	0.6652 (0.5681 - 0.7422)	0.6962 (0.6159 - 0.8835)	0.6925 (0.6179 - 0.7761)
Transient cerebral ischaemic attack, unspecified	0.7054 (0.6290 - 0.7758)	0.6913 (0.6345 - 0.7566)	0.6604 (0.5091 - 0.7967)	0.7222 (0.6263 - 0.8489)	0.7039 (0.6235 - 0.7760)	0.6801 (0.5939 - 0.7955)	0.6814 (0.5731 - 0.7651)
Peripheral vascular disease, unspecified	0.7686 (0.7031 - 0.8314)	0.7184 (0.6670 - 0.7898)	0.6346 (0.5435 - 0.8947)	0.8021 (0.5088 - 0.8560)	0.7623 (0.6244 - 0.8333)	0.6870 (0.6212 - 0.8462)	0.6926 (0.6320 - 0.7904)

b. Based on metabolome combined with clinical data

Disease	AUC (95%CI)	Acc (95%CI)	Sn (95%CI)	Sp (95%CI)	PPV (95%CI)	NPV (95%CI)	F1 score (95%CI)
Atherosclerotic heart disease	0.9092 (0.8990 - 0.9184)	0.8436 (0.8297 - 0.8577)	0.8808 (0.8490 - 0.9080)	0.8064 (0.7923 - 0.8259)	0.8198 (0.8032 - 0.8386)	0.8712 (0.8411 - 0.8993)	0.8492 (0.8318 - 0.8646)
Angina pectoris, unspecified	0.8741 (0.8577 - 0.8888)	0.8105 (0.7927 - 0.8318)	0.8047 (0.7795 - 0.8857)	0.8163 (0.7434 - 0.8223)	0.8142 (0.7626 - 0.8302)	0.8070 (0.7828 - 0.8698)	0.8094 (0.7915 - 0.8369)
Chronic ischaemic heart disease, unspecified	0.8829 (0.8644 - 0.9011)	0.8142 (0.7978 - 0.8398)	0.8718 (0.7778 - 0.9224)	0.7567 (0.7203 - 0.8540)	0.7818 (0.7538 - 0.8457)	0.8551 (0.7832 - 0.9041)	0.8243 (0.7986 - 0.8521)
Old myocardial infarction	0.8721 (0.8462 - 0.8947)	0.8141 (0.7884 - 0.8434)	0.8192 (0.7729 - 0.8988)	0.8089 (0.7422 - 0.8220)	0.8109 (0.7609 - 0.8362)	0.8173 (0.7737 - 0.8877)	0.8150 (0.7855 - 0.8504)
Unstable angina	0.8464 (0.8189 - 0.8715)	0.7706 (0.7455 - 0.8049)	0.7378 (0.6752 - 0.9070)	0.8034 (0.6598 - 0.8574)	0.7896 (0.6989 - 0.8343)	0.7539 (0.7154 - 0.8773)	0.7628 (0.7310 - 0.8161)
Left ventricular failure	0.8526 (0.8148 - 0.8896)	0.7979 (0.7647 - 0.8382)	0.7810 (0.7129 - 0.9027)	0.8148 (0.7104 - 0.8492)	0.8083 (0.7261 - 0.8422)	0.7881 (0.7396 - 0.8843)	0.7944 (0.7543 - 0.8438)
Acute transmural myocardial infarction of inferior wall	0.8637 (0.8297 - 0.8979)	0.7858 (0.7557 - 0.8439)	0.8000 (0.6825 - 1.0000)	0.7716 (0.5151 - 0.8710)	0.7779 (0.6749 - 0.8683)	0.7941 (0.7192 - 1.0000)	0.7888 (0.7478 - 0.8499)
Acute myocardial infarction, unspecified	0.8627 (0.8209 - 0.9014)	0.7965 (0.7625 - 0.8535)	0.7778 (0.7115 - 0.9672)	0.8152 (0.6495 - 0.8783)	0.8080 (0.6892 - 0.8654)	0.7858 (0.7404 - 0.9543)	0.7926 (0.7548 - 0.8599)
Supraventricular tachycardia	0.5973 (0.5217 - 0.6671)	0.5982 (0.5506 - 0.6602)	0.7143 (0.2461 - 0.8406)	0.4821 (0.4387 - 0.9191)	0.5797 (0.5154 - 0.7800)	0.6279 (0.5277 - 0.7574)	0.6400 (0.3691 - 0.7129)
Acute transmural myocardial infarction of anterior wall	0.9110 (0.8866 - 0.9345)	0.8597 (0.8285 - 0.8949)	0.9206 (0.8333 - 0.9839)	0.7988 (0.7932 - 0.8800)	0.8206 (0.7747 - 0.8790)	0.9096 (0.8335 - 0.9806)	0.8678 (0.8230 - 0.9058)
Pulmonary embolism without mention of acute cor pulmonale	0.6534 (0.5932 - 0.7137)	0.6350 (0.5975 - 0.6968)	0.7000 (0.4918 - 0.9400)	0.5701 (0.3742 - 0.7945)	0.6195 (0.5328 - 0.7094)	0.6552 (0.5855 - 0.8824)	0.6573 (0.5721 - 0.7532)
Cerebral infarction, unspecified	0.7740 (0.7106 - 0.8387)	0.7447 (0.6991 - 0.8070)	0.6964 (0.5733 - 0.8393)	0.7930 (0.7485 - 0.8692)	0.7708 (0.6960 - 0.8399)	0.7232 (0.6444 - 0.8412)	0.7318 (0.6549 - 0.8104)
Cardiomegaly	0.7032 (0.6375 - 0.7689)	0.6792 (0.6337 - 0.7347)	0.8333 (0.5438 - 0.9445)	0.5251 (0.4454 - 0.8224)	0.6370 (0.5623 - 0.7663)	0.7591 (0.6392 - 0.9047)	0.7220 (0.6253 - 0.7885)
Transient cerebral ischaemic attack, unspecified	0.7239 (0.6463 - 0.7939)	0.6862 (0.6309 - 0.7582)	0.6981 (0.4284 - 0.8269)	0.6742 (0.6167 - 0.8982)	0.6818 (0.6218 - 0.8299)	0.6907 (0.5801 - 0.8064)	0.6899 (0.5494 - 0.7649)
Peripheral vascular disease, unspecified	0.7983 (0.7299 - 0.8585)	0.7571 (0.7023 - 0.8165)	0.7885 (0.6274 - 0.9153)	0.7257 (0.6164 - 0.8594)	0.7419 (0.6644 - 0.8272)	0.7743 (0.6699 - 0.8935)	0.7645 (0.6850 - 0.8337)

Table S9a. Discrimination performance of XGBoost using selected metabolites for CVD class-level

Disease	AUC (95%CI)	Acc (95%CI)	Sn (95%CI)	Sp (95%CI)	PPV (95%CI)	NPV (95%CI)	F1 score (95%CI)
Essential (primary) hypertension	0.7371 (0.7331 - 0.7411)	0.6793 (0.6759 - 0.6833)	0.6232 (0.6024 - 0.6640)	0.7353 (0.6930 - 0.7558)	0.7019 (0.6844 - 0.7124)	0.6612 (0.6541 - 0.6750)	0.6602 (0.6524 - 0.6753)
Chronic ischaemic heart disease	0.8573 (0.8533 - 0.8620)	0.7863 (0.7825 - 0.7918)	0.8287 (0.7560 - 0.8366)	0.7439 (0.7426 - 0.8158)	0.7639 (0.7607 - 0.8063)	0.8128 (0.7694 - 0.8203)	0.7950 (0.7786 - 0.7995)
Angina pectoris	0.8224 (0.8165 - 0.8273)	0.7562 (0.7510 - 0.7620)	0.7388 (0.7259 - 0.7715)	0.7737 (0.7414 - 0.7823)	0.7655 (0.7480 - 0.7735)	0.7476 (0.7384 - 0.7654)	0.7519 (0.7453 - 0.7619)
Atrial fibrillation and flutter	0.7347 (0.7252 - 0.7438)	0.6748 (0.6674 - 0.6849)	0.6121 (0.5558 - 0.6819)	0.7374 (0.6692 - 0.7887)	0.6998 (0.6720 - 0.7324)	0.6553 (0.6363 - 0.6797)	0.6531 (0.6273 - 0.6796)
Acute myocardial infarction	0.8823 (0.8763 - 0.8883)	0.8124 (0.8058 - 0.8205)	0.8359 (0.8105 - 0.8834)	0.7890 (0.7429 - 0.8124)	0.7984 (0.7710 - 0.8137)	0.8278 (0.8106 - 0.8651)	0.8167 (0.8081 - 0.8284)
Heart failure	0.8401 (0.8274 - 0.8532)	0.7691 (0.7580 - 0.7838)	0.7426 (0.7122 - 0.8119)	0.7955 (0.7291 - 0.8200)	0.7841 (0.7469 - 0.8011)	0.7556 (0.7365 - 0.8008)	0.7628 (0.7483 - 0.7873)
Paroxysmal tachycardia	0.6299 (0.6090 - 0.6499)	0.6026 (0.5870 - 0.6227)	0.4579 (0.4059 - 0.6428)	0.7474 (0.5611 - 0.7956)	0.6444 (0.5864 - 0.6758)	0.5796 (0.5601 - 0.6221)	0.5354 (0.4998 - 0.6236)
Other peripheral vascular diseases	0.7783 (0.7614 - 0.7963)	0.7203 (0.7074 - 0.7392)	0.6667 (0.6091 - 0.7543)	0.7738 (0.7076 - 0.8219)	0.7467 (0.7044 - 0.7819)	0.6989 (0.6718 - 0.7434)	0.7044 (0.6796 - 0.7367)
Pulmonary embolism	0.6392 (0.6180 - 0.6597)	0.6045 (0.5901 - 0.6259)	0.6498 (0.5087 - 0.7531)	0.5593 (0.4715 - 0.7031)	0.5959 (0.5712 - 0.6394)	0.6149 (0.5777 - 0.6626)	0.6216 (0.5612 - 0.6638)
Transient cerebral ischaemic attacks and related syndromes	0.7366 (0.7165 - 0.7589)	0.6851 (0.6693 - 0.7081)	0.6558 (0.5832 - 0.7617)	0.7145 (0.5932 - 0.7732)	0.6967 (0.6579 - 0.7359)	0.6749 (0.6420 - 0.7237)	0.6756 (0.6421 - 0.7123)
Complications and ill-defined descriptions of heart disease	0.7552 (0.7328 - 0.7774)	0.7053 (0.6862 - 0.7269)	0.6117 (0.5637 - 0.6798)	0.7989 (0.7323 - 0.8433)	0.7526 (0.7091 - 0.7868)	0.6729 (0.6446 - 0.7092)	0.6749 (0.6468 - 0.7098)
Cerebral infarction	0.7941 (0.7742 - 0.8124)	0.7335 (0.7179 - 0.7541)	0.7481 (0.6678 - 0.8068)	0.7188 (0.6715 - 0.8084)	0.7268 (0.6994 - 0.7735)	0.7405 (0.7002 - 0.7836)	0.7373 (0.7089 - 0.7635)
Other cardiac arrhythmias	0.6701 (0.6445 - 0.6952)	0.6312 (0.6147 - 0.6573)	0.4821 (0.4548 - 0.7268)	0.7802 (0.5631 - 0.7892)	0.6869 (0.6016 - 0.7102)	0.6011 (0.5837 - 0.6746)	0.5666 (0.5425 - 0.6694)
Atrioventricular and left bundle-branch block	0.7191 (0.6959 - 0.7419)	0.6754 (0.6562 - 0.7011)	0.6120 (0.4939 - 0.6599)	0.7388 (0.7072 - 0.8367)	0.7009 (0.6780 - 0.7710)	0.6557 (0.6161 - 0.6870)	0.6534 (0.5940 - 0.6855)
Nonrheumatic mitral valve disorders	0.6757 (0.6505 - 0.7012)	0.6357 (0.6173 - 0.6614)	0.5705 (0.4352 - 0.7534)	0.7008 (0.5139 - 0.8357)	0.6560 (0.6081 - 0.7272)	0.6200 (0.5852 - 0.6791)	0.6103 (0.5377 - 0.6732)
Nonrheumatic aortic valve disorders	0.7537 (0.7312 - 0.7772)	0.6939 (0.6780 - 0.7199)	0.6691 (0.5807 - 0.7881)	0.7186 (0.6296 - 0.8124)	0.7040 (0.6587 - 0.7645)	0.6847 (0.6458 - 0.7521)	0.6861 (0.6493 - 0.7289)
Other conduction disorders	0.6550 (0.6266 - 0.6839)	0.6307 (0.6079 - 0.6596)	0.4863 (0.4316 - 0.6372)	0.7752 (0.6465 - 0.7932)	0.6839 (0.6145 - 0.7192)	0.6014 (0.5720 - 0.6498)	0.5684 (0.5274 - 0.6392)
Other cerebrovascular diseases	0.7124 (0.6847 - 0.7404)	0.6634 (0.6427 - 0.6916)	0.7079 (0.5705 - 0.8056)	0.6188 (0.5439 - 0.7395)	0.6500 (0.6155 - 0.7105)	0.6794 (0.6237 - 0.7370)	0.6777 (0.6203 - 0.7136)
Other disorders of arteries and arterioles	0.7881 (0.7620 - 0.8115)	0.7237 (0.7057 - 0.7494)	0.7377 (0.6039 - 0.8488)	0.7097 (0.6179 - 0.8548)	0.7176 (0.6697 - 0.7996)	0.7301 (0.6745 - 0.8096)	0.7275 (0.6833 - 0.7679)

Hypertensive renal disease	0.9078 (0.8886 - 0.9271)	0.8370 (0.8171 - 0.8621)	0.7950 (0.7236 - 0.8515)	0.8790 (0.8502 - 0.9465)	0.8679 (0.8355 - 0.9312)	0.8109 (0.7683 - 0.8560)	0.8299 (0.8030 - 0.8586)
Stroke, not specified as haemorrhage or infarction	0.8092 (0.7832 - 0.8326)	0.7543 (0.7325 - 0.7792)	0.7819 (0.7090 - 0.8234)	0.7267 (0.7253 - 0.7903)	0.7410 (0.7176 - 0.7819)	0.7691 (0.7203 - 0.8098)	0.7609 (0.7249 - 0.7878)
Cardiomyopathy	0.7596 (0.7286 - 0.7890)	0.7048 (0.6833 - 0.7357)	0.6378 (0.6000 - 0.8548)	0.7718 (0.5756 - 0.8040)	0.7365 (0.6444 - 0.7676)	0.6806 (0.6531 - 0.8043)	0.6836 (0.6600 - 0.7551)
Sequelae of cerebrovascular disease	0.7411 (0.7091 - 0.7691)	0.6989 (0.6766 - 0.7268)	0.7882 (0.6260 - 0.8397)	0.6095 (0.5932 - 0.7684)	0.6687 (0.6381 - 0.7375)	0.7421 (0.6644 - 0.7956)	0.7236 (0.6660 - 0.7562)
Subarachnoid haemorrhage	0.5078 (0.4706 - 0.5431)	0.5166 (0.4986 - 0.5544)	0.6475 (0.1149 - 0.9957)	0.3856 (0.0503 - 0.9245)	0.5131 (0.4874 - 0.6189)	0.5225 (0.4944 - 0.9258)	0.5726 (0.1925 - 0.6830)
Atherosclerosis	0.8217 (0.7941 - 0.8468)	0.7609 (0.7419 - 0.7890)	0.7592 (0.6856 - 0.8550)	0.7626 (0.6797 - 0.8341)	0.7618 (0.7127 - 0.8139)	0.7600 (0.7189 - 0.8314)	0.7605 (0.7289 - 0.7989)
Subsequent myocardial infarction	0.8923 (0.8721 - 0.9101)	0.8297 (0.8115 - 0.8558)	0.8571 (0.7739 - 0.9250)	0.8022 (0.7379 - 0.8675)	0.8125 (0.7682 - 0.8680)	0.8488 (0.7898 - 0.9086)	0.8342 (0.8068 - 0.8611)
Occlusion and stenosis of precerebral arteries, not resulting in cerebral infarction	0.8355 (0.8079 - 0.8604)	0.7741 (0.7512 - 0.7999)	0.8398 (0.7383 - 0.8940)	0.7083 (0.6710 - 0.8078)	0.7422 (0.7080 - 0.8027)	0.8156 (0.7495 - 0.8734)	0.7880 (0.7543 - 0.8189)
Arterial embolism and thrombosis	0.7468 (0.7123 - 0.7812)	0.7007 (0.6750 - 0.7354)	0.6502 (0.5815 - 0.8000)	0.7511 (0.6304 - 0.8124)	0.7232 (0.6558 - 0.7669)	0.6823 (0.6453 - 0.7662)	0.6848 (0.6467 - 0.7469)
Other acute ischaemic heart diseases	0.8774 (0.8522 - 0.9021)	0.8230 (0.8032 - 0.8488)	0.8895 (0.7988 - 0.9399)	0.7564 (0.7108 - 0.8454)	0.7850 (0.7531 - 0.8433)	0.8725 (0.8049 - 0.9275)	0.8340 (0.8059 - 0.8602)
Other diseases of pericardium	0.6339 (0.5885 - 0.6764)	0.6095 (0.5839 - 0.6483)	0.6579 (0.3172 - 0.7464)	0.5611 (0.4841 - 0.8821)	0.5998 (0.5671 - 0.7556)	0.6212 (0.5491 - 0.6829)	0.6275 (0.4375 - 0.6778)
Multiple valve diseases	0.7516 (0.7189 - 0.7833)	0.7052 (0.6815 - 0.7411)	0.7323 (0.5943 - 0.7989)	0.6780 (0.6417 - 0.7955)	0.6946 (0.6635 - 0.7705)	0.7170 (0.6533 - 0.7709)	0.7130 (0.6518 - 0.7498)
Aortic aneurysm and dissection	0.8177 (0.7859 - 0.8460)	0.7511 (0.7230 - 0.7861)	0.7730 (0.6428 - 0.8306)	0.7293 (0.7274 - 0.8388)	0.7406 (0.7087 - 0.8150)	0.7626 (0.6892 - 0.8137)	0.7565 (0.7026 - 0.7909)
Intracerebral haemorrhage	0.6760 (0.6315 - 0.7196)	0.6500 (0.6126 - 0.6957)	0.5758 (0.4772 - 0.7280)	0.7242 (0.6310 - 0.7779)	0.6761 (0.6059 - 0.7330)	0.6306 (0.5771 - 0.7172)	0.6219 (0.5558 - 0.6917)
Diseases of capillaries	0.5564 (0.4992 - 0.6129)	0.5626 (0.5292 - 0.6202)	0.8652 (0.2571 - 0.9348)	0.2599 (0.2411 - 0.8640)	0.5390 (0.4956 - 0.6974)	0.6585 (0.5148 - 0.7937)	0.6642 (0.3615 - 0.7192)
Cardiac arrest	0.8150 (0.7739 - 0.8563)	0.7934 (0.7607 - 0.8313)	0.8673 (0.7809 - 0.9333)	0.7195 (0.7175 - 0.7676)	0.7557 (0.7113 - 0.8001)	0.8443 (0.7703 - 0.9103)	0.8077 (0.7580 - 0.8491)
Other pulmonary heart diseases	0.6956 (0.6349 - 0.7479)	0.6569 (0.6188 - 0.7139)	0.5474 (0.3655 - 0.8021)	0.7665 (0.5302 - 0.9022)	0.7010 (0.5968 - 0.8356)	0.6287 (0.5681 - 0.7416)	0.6147 (0.4979 - 0.7167)
Rheumatic mitral valve diseases	0.6538 (0.5955 - 0.7128)	0.6437 (0.5999 - 0.6940)	0.5667 (0.4624 - 0.7326)	0.7206 (0.6052 - 0.8043)	0.6698 (0.5916 - 0.7333)	0.6245 (0.5650 - 0.7143)	0.6139 (0.5337 - 0.6963)

Table S9b. internal geographic hold-out validation

Disease	AUC (95%CI)	Acc (95%CI)	Sn (95%CI)	Sp (95%CI)	PPV (95%CI)	NPV (95%CI)	F1 score (95%CI)

Essential (primary) hypertension	0.7494 (0.7340 - 0.7643)	0.6951 (0.6813 - 0.7091)	0.6421 (0.5844 - 0.6809)	0.7481 (0.7037 - 0.7952)	0.7182 (0.6981 - 0.7493)	0.6764 (0.6516 - 0.6988)	0.6780 (0.6523 - 0.6991)
Chronic ischaemic heart disease	0.8762 (0.8644 - 0.8879)	0.8031 (0.7918 - 0.8189)	0.8395 (0.7903 - 0.9023)	0.7666 (0.7003 - 0.8107)	0.7825 (0.7485 - 0.8150)	0.8269 (0.7913 - 0.8790)	0.8100 (0.7938 - 0.8308)
Angina pectoris	0.8329 (0.8139 - 0.8515)	0.7641 (0.7495 - 0.7853)	0.7972 (0.7155 - 0.8401)	0.7311 (0.7142 - 0.8106)	0.7478 (0.7258 - 0.7960)	0.7828 (0.7338 - 0.8226)	0.7717 (0.7451 - 0.7928)
Atrial fibrillation and flutter	0.7311 (0.6973 - 0.7674)	0.6782 (0.6552 - 0.7142)	0.5638 (0.5130 - 0.8091)	0.7926 (0.5648 - 0.8256)	0.7311 (0.6250 - 0.7717)	0.6450 (0.6122 - 0.7492)	0.6366 (0.6000 - 0.7204)
Acute myocardial infarction	0.8739 (0.8539 - 0.8937)	0.7992 (0.7798 - 0.8276)	0.8756 (0.7363 - 0.9246)	0.7228 (0.6586 - 0.8741)	0.7595 (0.7286 - 0.8517)	0.8532 (0.7629 - 0.9071)	0.8134 (0.7781 - 0.8402)
Heart failure	0.8346 (0.7965 - 0.8659)	0.7771 (0.7459 - 0.8121)	0.8295 (0.6690 - 0.8865)	0.7248 (0.7204 - 0.8692)	0.7508 (0.7190 - 0.8445)	0.8095 (0.7139 - 0.8672)	0.7882 (0.7292 - 0.8234)
Paroxysmal tachycardia	0.5777 (0.5160 - 0.6399)	0.5813 (0.5471 - 0.6399)	0.5870 (0.3103 - 0.7097)	0.5756 (0.4965 - 0.8292)	0.5803 (0.5317 - 0.6920)	0.5822 (0.5153 - 0.6721)	0.5836 (0.4155 - 0.6530)
Complications and ill-defined descriptions of heart disease	0.6916 (0.6338 - 0.7458)	0.6519 (0.6157 - 0.7155)	0.6471 (0.4374 - 0.9335)	0.6568 (0.3465 - 0.8473)	0.6534 (0.5539 - 0.7871)	0.6505 (0.5796 - 0.8641)	0.6502 (0.5442 - 0.7507)
Other cardiac arrhythmias	0.6129 (0.5509 - 0.6758)	0.6069 (0.5662 - 0.6601)	0.8824 (0.4091 - 0.9524)	0.3314 (0.3244 - 0.7797)	0.5689 (0.5162 - 0.6975)	0.7380 (0.5535 - 0.8636)	0.6918 (0.4996 - 0.7420)
Cerebral infarction	0.8023 (0.7426 - 0.8582)	0.7650 (0.7200 - 0.8205)	0.7015 (0.6282 - 0.8677)	0.8285 (0.6913 - 0.8607)	0.8036 (0.7107 - 0.8454)	0.7351 (0.6803 - 0.8610)	0.7491 (0.6884 - 0.8249)
Other peripheral vascular diseases	0.7119 (0.6437 - 0.7786)	0.6679 (0.6295 - 0.7390)	0.6970 (0.4246 - 0.9063)	0.6389 (0.4336 - 0.8839)	0.6587 (0.5850 - 0.8227)	0.6783 (0.5817 - 0.8371)	0.6773 (0.5405 - 0.7552)
Pulmonary embolism	0.6201 (0.5506 - 0.6832)	0.6124 (0.5646 - 0.6766)	0.6825 (0.4054 - 0.9384)	0.5422 (0.2580 - 0.8103)	0.5985 (0.5265 - 0.6902)	0.6307 (0.5553 - 0.8169)	0.6378 (0.4973 - 0.7224)
Transient cerebral ischaemic attacks and related syndromes	0.6942 (0.6194 - 0.7643)	0.6613 (0.6198 - 0.7354)	0.5167 (0.3703 - 0.8001)	0.8059 (0.5611 - 0.9172)	0.7269 (0.5927 - 0.8441)	0.6251 (0.5666 - 0.7584)	0.6040 (0.5050 - 0.7348)
Atrioventricular and left bundle-branch block	0.6627 (0.5697 - 0.7505)	0.6548 (0.6048 - 0.7303)	0.5000 (0.3620 - 0.8136)	0.8095 (0.5707 - 0.9126)	0.7241 (0.5889 - 0.8184)	0.6182 (0.5518 - 0.7798)	0.5916 (0.4832 - 0.7389)
Nonrheumatic aortic valve disorders	0.7455 (0.6870 - 0.7975)	0.7012 (0.6551 - 0.7588)	0.8400 (0.6303 - 0.9584)	0.5623 (0.4309 - 0.7445)	0.6574 (0.5832 - 0.7535)	0.7785 (0.6496 - 0.9331)	0.7376 (0.6517 - 0.7989)

Table S10a. Discrimination performance of XGBoost using selected metabolites for CVD subclass-level

Disease	AUC (95%CI)	Acc (95%CI)	Sn (95%CI)	Sp (95%CI)	PPV (95%CI)	NPV (95%CI)	F1 score (95%CI)
Atherosclerotic heart disease	0.8740 (0.8692 - 0.8783)	0.8027 (0.7977 - 0.8084)	0.8239 (0.8099 - 0.8686)	0.7814 (0.7340 - 0.7944)	0.7903 (0.7658 - 0.7993)	0.8161 (0.8047 - 0.8477)	0.8068 (0.8006 - 0.8153)
Angina pectoris, unspecified	0.8231 (0.8168 - 0.8292)	0.7566 (0.7507 - 0.7635)	0.7691 (0.7313 - 0.7857)	0.7440 (0.7333 - 0.7804)	0.7503 (0.7420 - 0.7718)	0.7631 (0.7410 - 0.7758)	0.7596 (0.7475 - 0.7683)
Chronic ischaemic heart disease, unspecified	0.8221 (0.8122 - 0.8311)	0.7565 (0.7484 - 0.7662)	0.7520 (0.7224 - 0.7895)	0.7610 (0.7314 - 0.7925)	0.7588 (0.7384 - 0.7758)	0.7542 (0.7379 - 0.7794)	0.7554 (0.7430 - 0.7699)
Other forms of chronic ischaemic heart disease	0.8754 (0.8680 - 0.8824)	0.8071 (0.7993 - 0.8166)	0.8060 (0.7888 - 0.8453)	0.8082 (0.7742 - 0.8189)	0.8077 (0.7830 - 0.8185)	0.8064 (0.7932 - 0.8348)	0.8069 (0.7987 - 0.8203)
Unstable angina	0.8307 (0.8209 - 0.8403)	0.7706 (0.7622 - 0.7811)	0.7899 (0.7363 - 0.8098)	0.7514 (0.7384 - 0.8034)	0.7606 (0.7503 - 0.7923)	0.7814 (0.7493 - 0.7979)	0.7749 (0.7589 - 0.7868)
Old myocardial infarction	0.8578 (0.8474 - 0.8677)	0.7842 (0.7743 - 0.7976)	0.8322 (0.7521 - 0.8651)	0.7363 (0.7050 - 0.8206)	0.7594 (0.7403 - 0.8074)	0.8144 (0.7645 - 0.8428)	0.7941 (0.7732 - 0.8074)
Acute myocardial infarction, unspecified	0.8728 (0.8609 - 0.8836)	0.8100 (0.7979 - 0.8218)	0.8434 (0.8031 - 0.8765)	0.7766 (0.7540 - 0.8132)	0.7906 (0.7713 - 0.8107)	0.8322 (0.8025 - 0.8612)	0.8161 (0.7998 - 0.8307)
Acute transmural myocardial infarction of inferior wall	0.8774 (0.8672 - 0.8885)	0.8102 (0.8007 - 0.8263)	0.8459 (0.8169 - 0.9138)	0.7745 (0.7049 - 0.8119)	0.7896 (0.7540 - 0.8137)	0.8341 (0.8138 - 0.8935)	0.8168 (0.8054 - 0.8371)
Left ventricular failure	0.8508 (0.8335 - 0.8661)	0.7824 (0.7678 - 0.7983)	0.8286 (0.7231 - 0.8549)	0.7362 (0.7325 - 0.8515)	0.7585 (0.7443 - 0.8255)	0.8112 (0.7526 - 0.8388)	0.7920 (0.7641 - 0.8085)
Pulmonary embolism without mention of acute cor pulmonale	0.6437 (0.6225 - 0.6637)	0.6082 (0.5919 - 0.6275)	0.7256 (0.5048 - 0.7672)	0.4907 (0.4718 - 0.7014)	0.5876 (0.5682 - 0.6431)	0.6414 (0.5814 - 0.6788)	0.6493 (0.5590 - 0.6723)
Supraventricular tachycardia	0.5989 (0.5758 - 0.6227)	0.5733 (0.5613 - 0.5961)	0.5050 (0.3713 - 0.7738)	0.6417 (0.3794 - 0.7716)	0.5849 (0.5434 - 0.6336)	0.5645 (0.5443 - 0.6375)	0.5420 (0.4612 - 0.6539)
Peripheral vascular disease, unspecified	0.8402 (0.8252 - 0.8563)	0.7757 (0.7621 - 0.7919)	0.8152 (0.7307 - 0.8436)	0.7362 (0.7293 - 0.8159)	0.7555 (0.7400 - 0.8040)	0.7993 (0.7472 - 0.8269)	0.7842 (0.7574 - 0.8023)
Acute transmural myocardial infarction of anterior wall	0.8849 (0.8729 - 0.8960)	0.8170 (0.8060 - 0.8333)	0.8420 (0.7973 - 0.9081)	0.7920 (0.7322 - 0.8403)	0.8019 (0.7629 - 0.8335)	0.8337 (0.8024 - 0.8888)	0.8215 (0.8059 - 0.8416)
Transient cerebral ischaemic attack, unspecified	0.7491 (0.7260 - 0.7715)	0.7013 (0.6838 - 0.7249)	0.6396 (0.5848 - 0.7598)	0.7630 (0.6529 - 0.8059)	0.7296 (0.6760 - 0.7659)	0.6792 (0.6523 - 0.7382)	0.6816 (0.6534 - 0.7283)
Cerebral infarction, unspecified	0.8005 (0.7809 - 0.8216)	0.7383 (0.7196 - 0.7589)	0.7658 (0.7108 - 0.8115)	0.7108 (0.6799 - 0.7393)	0.7258 (0.6999 - 0.7528)	0.7521 (0.7134 - 0.7949)	0.7453 (0.7134 - 0.7698)
Mitral (valve) insufficiency	0.7016 (0.6748 - 0.7284)	0.6579 (0.6370 - 0.6860)	0.5464 (0.5040 - 0.6782)	0.7694 (0.6662 - 0.7973)	0.7032 (0.6434 - 0.7321)	0.6291 (0.6003 - 0.6833)	0.6150 (0.5829 - 0.6765)
Cardiomegaly	0.7585 (0.7319 - 0.7838)	0.7074 (0.6882 - 0.7333)	0.7218 (0.5959 - 0.7710)	0.6929 (0.6485 - 0.8175)	0.7015 (0.6758 - 0.7730)	0.7135 (0.6579 - 0.7541)	0.7115 (0.6650 - 0.7397)
Other forms of angina pectoris	0.8330 (0.8121 - 0.8531)	0.7733 (0.7564 - 0.7962)	0.8256 (0.7175 - 0.8865)	0.7210 (0.6468 - 0.8208)	0.7474 (0.7134 - 0.8120)	0.8052 (0.7392 - 0.8564)	0.7845 (0.7522 - 0.8094)
Acute subendocardial myocardial infarction	0.8624 (0.8452 - 0.8797)	0.7930 (0.7770 - 0.8174)	0.8125 (0.7820 - 0.8986)	0.7735 (0.7034 - 0.8021)	0.7820 (0.7386 - 0.8061)	0.8049 (0.7781 - 0.8747)	0.7969 (0.7781 - 0.8295)

Hypertensive renal disease with renal failure	0.9125 (0.8904 - 0.9310)	0.8490 (0.8304 - 0.8735)	0.8293 (0.7276 - 0.8826)	0.8687 (0.8236 - 0.9477)	0.8633 (0.8301 - 0.9373)	0.8357 (0.7720 - 0.8812)	0.8460 (0.8158 - 0.8716)
Congestive heart failure	0.8375 (0.8145 - 0.8603)	0.7651 (0.7451 - 0.7924)	0.6964 (0.6514 - 0.9007)	0.8338 (0.6213 - 0.8546)	0.8074 (0.6945 - 0.8347)	0.7331 (0.7002 - 0.8648)	0.7478 (0.7225 - 0.7978)
Aortic (valve) stenosis	0.7854 (0.7597 - 0.8123)	0.7184 (0.7009 - 0.7515)	0.6795 (0.6495 - 0.8908)	0.7574 (0.5505 - 0.7848)	0.7369 (0.6495 - 0.7684)	0.7026 (0.6813 - 0.8446)	0.7070 (0.6897 - 0.7776)
Left bundle-branch block, unspecified	0.7223 (0.6890 - 0.7554)	0.6770 (0.6519 - 0.7117)	0.6356 (0.5593 - 0.8235)	0.7185 (0.5595 - 0.7878)	0.6930 (0.6262 - 0.7398)	0.6635 (0.6265 - 0.7670)	0.6630 (0.6223 - 0.7307)
Other and unspecified right bundle-branch block	0.6542 (0.6175 - 0.6919)	0.6238 (0.5988 - 0.6639)	0.5305 (0.3395 - 0.6181)	0.7171 (0.6891 - 0.8821)	0.6522 (0.6113 - 0.7776)	0.6044 (0.5578 - 0.6576)	0.5851 (0.4638 - 0.6454)
Ventricular tachycardia	0.7555 (0.7160 - 0.7948)	0.7157 (0.6887 - 0.7528)	0.6984 (0.6178 - 0.8034)	0.7330 (0.6474 - 0.7763)	0.7234 (0.6699 - 0.7681)	0.7085 (0.6610 - 0.7793)	0.7107 (0.6622 - 0.7599)
Cardiac arrhythmia, unspecified	0.6263 (0.5814 - 0.6665)	0.6142 (0.5873 - 0.6565)	0.4619 (0.3439 - 0.6554)	0.7664 (0.6020 - 0.8489)	0.6641 (0.5943 - 0.7480)	0.5875 (0.5495 - 0.6540)	0.5449 (0.4580 - 0.6424)
Occlusion and stenosis of carotid artery	0.8428 (0.8151 - 0.8694)	0.7822 (0.7589 - 0.8149)	0.7949 (0.7336 - 0.8932)	0.7696 (0.6822 - 0.8051)	0.7753 (0.7164 - 0.8137)	0.7896 (0.7459 - 0.8694)	0.7850 (0.7529 - 0.8239)
Raynaud's syndrome	0.6262 (0.5905 - 0.6622)	0.6072 (0.5797 - 0.6407)	0.7296 (0.5454 - 0.9046)	0.4848 (0.2676 - 0.6693)	0.5861 (0.5424 - 0.6334)	0.6420 (0.5797 - 0.7433)	0.6500 (0.5758 - 0.7004)
Stricture of artery	0.8370 (0.8114 - 0.8615)	0.7693 (0.7424 - 0.7984)	0.8122 (0.7273 - 0.8865)	0.7264 (0.6632 - 0.7935)	0.7480 (0.7098 - 0.7948)	0.7945 (0.7351 - 0.8651)	0.7788 (0.7393 - 0.8137)
Sequelae of stroke, not specified as haemorrhage or infarction	0.7777 (0.7442 - 0.8085)	0.7323 (0.7070 - 0.7642)	0.8562 (0.6956 - 0.9205)	0.6084 (0.5684 - 0.7697)	0.6861 (0.6470 - 0.7558)	0.8088 (0.7159 - 0.8863)	0.7618 (0.7124 - 0.7983)
Subarachnoid haemorrhage, unspecified	0.5068 (0.4612 - 0.5550)	0.5219 (0.5004 - 0.5722)	0.4497 (0.0391 - 0.9704)	0.5941 (0.0611 - 0.9822)	0.5256 (0.4892 - 0.7597)	0.5191 (0.4861 - 0.6907)	0.4847 (0.0742 - 0.6700)
Atherosclerosis of arteries of the extremities	0.8174 (0.7783 - 0.8533)	0.7687 (0.7376 - 0.8083)	0.6867 (0.6364 - 0.8358)	0.8508 (0.7011 - 0.8674)	0.8215 (0.7340 - 0.8469)	0.7308 (0.6914 - 0.8207)	0.7481 (0.7119 - 0.8077)
Dilated cardiomyopathy	0.7809 (0.7441 - 0.8210)	0.7276 (0.6976 - 0.7675)	0.7241 (0.6058 - 0.8088)	0.7310 (0.6753 - 0.8149)	0.7292 (0.6851 - 0.7931)	0.7260 (0.6631 - 0.7858)	0.7266 (0.6715 - 0.7749)
Aortic (valve) insufficiency	0.6886 (0.6445 - 0.7302)	0.6522 (0.6191 - 0.6985)	0.5473 (0.4431 - 0.7482)	0.7571 (0.5812 - 0.8338)	0.6926 (0.6085 - 0.7655)	0.6258 (0.5785 - 0.7090)	0.6114 (0.5463 - 0.6900)
Heart failure, unspecified	0.7919 (0.7515 - 0.8286)	0.7222 (0.6942 - 0.7658)	0.7338 (0.5774 - 0.8532)	0.7105 (0.6039 - 0.8508)	0.7171 (0.6603 - 0.8136)	0.7275 (0.6551 - 0.8206)	0.7254 (0.6586 - 0.7769)
Cerebrovascular disease, unspecified	0.7638 (0.7145 - 0.8080)	0.7252 (0.6918 - 0.7694)	0.7350 (0.6090 - 0.8254)	0.7153 (0.6558 - 0.8117)	0.7208 (0.6749 - 0.7972)	0.7297 (0.6572 - 0.8097)	0.7279 (0.6670 - 0.7782)
Mitral (valve) prolapse	0.6413 (0.6015 - 0.6820)	0.6245 (0.5978 - 0.6678)	0.7068 (0.5151 - 0.8547)	0.5422 (0.3986 - 0.7191)	0.6069 (0.5619 - 0.6824)	0.6490 (0.5864 - 0.7622)	0.6530 (0.5744 - 0.7155)
Subsequent myocardial infarction of unspecified site	0.8734 (0.8377 - 0.9044)	0.8032 (0.7775 - 0.8430)	0.7480 (0.7027 - 0.9508)	0.8585 (0.6711 - 0.8849)	0.8409 (0.7133 - 0.8743)	0.7731 (0.7361 - 0.9325)	0.7917 (0.7677 - 0.8479)
Disease of pericardium, unspecified	0.6156 (0.5630 - 0.6638)	0.6021 (0.5671 - 0.6495)	0.6972 (0.3301 - 0.8164)	0.5070 (0.4171 - 0.8548)	0.5858 (0.5320 - 0.7078)	0.6261 (0.5444 - 0.7305)	0.6367 (0.4434 - 0.6990)
Embolism and thrombosis of arteries of the lower extremities	0.7831 (0.7428 - 0.8227)	0.7412 (0.7100 - 0.7793)	0.8273 (0.6929 - 0.8990)	0.6552 (0.6283 - 0.7694)	0.7058 (0.6660 - 0.7711)	0.7914 (0.7080 - 0.8684)	0.7617 (0.7082 - 0.8027)

Other forms of acute ischaemic heart disease	0.8773 (0.8385 - 0.9126)	0.8330 (0.8002 - 0.8699)	0.8526 (0.7961 - 0.9348)	0.8134 (0.7875 - 0.8339)	0.8204 (0.7666 - 0.8489)	0.8466 (0.7896 - 0.9280)	0.8362 (0.7975 - 0.8775)
Other specified cardiac arrhythmias	0.6238 (0.5682 - 0.6830)	0.6199 (0.5786 - 0.6727)	0.6061 (0.4643 - 0.8047)	0.6338 (0.4232 - 0.7198)	0.6233 (0.5561 - 0.6887)	0.6167 (0.5523 - 0.7154)	0.6146 (0.5320 - 0.6934)
Naevus, nonneoplastic	0.5577 (0.4866 - 0.6250)	0.5611 (0.5170 - 0.6333)	0.4179 (0.2464 - 0.9719)	0.7043 (0.1053 - 0.8679)	0.5856 (0.4908 - 0.6838)	0.5475 (0.5011 - 0.8298)	0.4878 (0.3549 - 0.6880)
Atrioventricular block, complete	0.7218 (0.6574 - 0.7764)	0.6869 (0.6554 - 0.7431)	0.7732 (0.4583 - 0.8597)	0.6006 (0.5304 - 0.8950)	0.6594 (0.6160 - 0.8376)	0.7259 (0.6001 - 0.8102)	0.7118 (0.5793 - 0.7634)
Acute transmural myocardial infarction of other sites	0.8892 (0.8646 - 0.9128)	0.8344 (0.8062 - 0.8694)	0.8454 (0.8000 - 0.9560)	0.8235 (0.7226 - 0.8474)	0.8273 (0.7475 - 0.8564)	0.8419 (0.7998 - 0.9461)	0.8362 (0.8024 - 0.8764)
Atrioventricular block, first degree	0.7138 (0.6579 - 0.7645)	0.6868 (0.6456 - 0.7400)	0.6598 (0.4999 - 0.7551)	0.7138 (0.6954 - 0.8275)	0.6974 (0.6476 - 0.7839)	0.6772 (0.6044 - 0.7523)	0.6781 (0.5838 - 0.7422)
Other ill-defined heart diseases	0.8185 (0.7736 - 0.8618)	0.7850 (0.7468 - 0.8263)	0.7624 (0.6638 - 0.8404)	0.8077 (0.8059 - 0.9068)	0.7986 (0.7595 - 0.8634)	0.7727 (0.7055 - 0.8446)	0.7801 (0.7212 - 0.8275)
Cardiomyopathy, unspecified	0.7256 (0.6741 - 0.7750)	0.6895 (0.6519 - 0.7431)	0.5761 (0.5126 - 0.8027)	0.8029 (0.5824 - 0.8196)	0.7451 (0.6352 - 0.7872)	0.6545 (0.6064 - 0.7706)	0.6498 (0.5957 - 0.7426)
Abdominal aortic aneurysm, without mention of rupture	0.8323 (0.7777 - 0.8824)	0.7926 (0.7571 - 0.8450)	0.7614 (0.6624 - 0.8691)	0.8238 (0.7554 - 0.8942)	0.8120 (0.7568 - 0.8749)	0.7754 (0.7100 - 0.8678)	0.7859 (0.7353 - 0.8457)
Intracerebral haemorrhage, unspecified	0.5993 (0.5454 - 0.6564)	0.5790 (0.5502 - 0.6413)	0.8721 (0.3788 - 1.0000)	0.2860 (0.1641 - 0.8222)	0.5498 (0.5038 - 0.6893)	0.6910 (0.5372 - 1.0000)	0.6744 (0.4801 - 0.7316)

Table S10b. Internal geographic hold-out validation

Disease	AUC (95%CI)	Acc (95%CI)	Sn (95%CI)	Sp (95%CI)	PPV (95%CI)	NPV (95%CI)	F1 score (95%CI)
Atherosclerotic heart disease	0.8861 (0.8749 - 0.8971)	0.8098 (0.7962 - 0.8264)	0.8385 (0.8143 - 0.9120)	0.7812 (0.7119 - 0.7988)	0.7930 (0.7467 - 0.8088)	0.8286 (0.8064 - 0.8943)	0.8151 (0.8003 - 0.8366)
Angina pectoris, unspecified	0.8347 (0.8136 - 0.8537)	0.7691 (0.7515 - 0.7925)	0.7731 (0.7180 - 0.8679)	0.7650 (0.6790 - 0.8140)	0.7669 (0.7160 - 0.8019)	0.7712 (0.7371 - 0.8402)	0.7700 (0.7455 - 0.8014)
Chronic ischaemic heart disease, unspecified	0.8490 (0.8255 - 0.8715)	0.7806 (0.7584 - 0.8077)	0.8034 (0.7352 - 0.8651)	0.7578 (0.7337 - 0.8240)	0.7684 (0.7313 - 0.8081)	0.7940 (0.7468 - 0.8514)	0.7855 (0.7531 - 0.8175)
Old myocardial infarction	0.8500 (0.8227 - 0.8752)	0.7802 (0.7572 - 0.8130)	0.8192 (0.7104 - 0.8921)	0.7412 (0.6818 - 0.8534)	0.7599 (0.7207 - 0.8324)	0.8039 (0.7359 - 0.8692)	0.7885 (0.7495 - 0.8251)
Unstable angina	0.8196 (0.7858 - 0.8504)	0.7499 (0.7245 - 0.7874)	0.7805 (0.6232 - 0.8427)	0.7193 (0.6776 - 0.8809)	0.7355 (0.7027 - 0.8392)	0.7662 (0.6858 - 0.8207)	0.7573 (0.7026 - 0.7959)
Left ventricular failure	0.8517 (0.8204 - 0.8819)	0.7810 (0.7546 - 0.8213)	0.8095 (0.6964 - 0.9314)	0.7524 (0.6478 - 0.8911)	0.7658 (0.6978 - 0.8611)	0.7980 (0.7356 - 0.9121)	0.7871 (0.7523 - 0.8391)
Acute transmural myocardial infarction of inferior wall	0.8433 (0.7971 - 0.8828)	0.7767 (0.7360 - 0.8309)	0.7538 (0.6621 - 0.9385)	0.7995 (0.5509 - 0.8453)	0.7899 (0.6780 - 0.8447)	0.7646 (0.6998 - 0.9171)	0.7714 (0.7181 - 0.8367)
Acute myocardial infarction, unspecified	0.8597 (0.8204 - 0.8947)	0.7957 (0.7599 - 0.8506)	0.7460 (0.7000 - 0.9465)	0.8455 (0.6257 - 0.8508)	0.8284 (0.7019 - 0.8612)	0.7690 (0.7273 - 0.9377)	0.7851 (0.7483 - 0.8587)

Supraventricular tachycardia	0.5518 (0.4775 - 0.6284)	0.5601 (0.5174 - 0.6293)	0.3810 (0.1272 - 0.8621)	0.7393 (0.2521 - 0.9548)	0.5937 (0.5058 - 0.8039)	0.5443 (0.4875 - 0.6850)	0.4641 (0.2123 - 0.6706)
Acute transmural myocardial infarction of anterior wall	0.8931 (0.8596 - 0.9209)	0.8194 (0.7852 - 0.8708)	0.7778 (0.7162 - 0.9605)	0.8610 (0.7190 - 0.9072)	0.8484 (0.7297 - 0.8970)	0.7949 (0.7499 - 0.9481)	0.8116 (0.7750 - 0.8748)
Pulmonary embolism without mention of acute cor pulmonale	0.6462 (0.5772 - 0.7118)	0.6284 (0.5863 - 0.6940)	0.7333 (0.4090 - 0.9201)	0.5234 (0.2784 - 0.8273)	0.6061 (0.5386 - 0.7258)	0.6625 (0.5606 - 0.8400)	0.6637 (0.5080 - 0.7412)
Cerebral infarction, unspecified	0.7683 (0.6948 - 0.8342)	0.7405 (0.6835 - 0.8054)	0.6607 (0.5428 - 0.8491)	0.8203 (0.5819 - 0.8705)	0.7861 (0.6724 - 0.8344)	0.7074 (0.6299 - 0.8317)	0.7180 (0.6289 - 0.8029)
Cardiomegaly	0.6905 (0.6305 - 0.7497)	0.6386 (0.6080 - 0.7117)	0.7037 (0.4464 - 0.9556)	0.5736 (0.3392 - 0.8368)	0.6227 (0.5472 - 0.7673)	0.6594 (0.5820 - 0.9009)	0.6607 (0.5432 - 0.7559)
Transient cerebral ischaemic attack, unspecified	0.6624 (0.5792 - 0.7416)	0.6549 (0.5927 - 0.7246)	0.5660 (0.3447 - 0.7925)	0.7437 (0.5640 - 0.9385)	0.6883 (0.5811 - 0.8386)	0.6315 (0.5439 - 0.7607)	0.6212 (0.4709 - 0.7291)
Peripheral vascular disease, unspecified	0.7878 (0.7243 - 0.8425)	0.7312 (0.6823 - 0.7987)	0.6923 (0.5818 - 0.9455)	0.7701 (0.5194 - 0.8651)	0.7507 (0.6168 - 0.8232)	0.7145 (0.6405 - 0.9255)	0.7203 (0.6544 - 0.8066)
